# DNA Barcoding and the Associated PhylAphidB@se Website for the Identification of European Aphids (Insecta: Hemiptera: Aphididae)

**DOI:** 10.1371/journal.pone.0097620

**Published:** 2014-06-04

**Authors:** Armelle Coeur d’acier, Astrid Cruaud, Emmanuelle Artige, Gwenaëlle Genson, Anne-Laure Clamens, Eric Pierre, Sylvie Hudaverdian, Jean-Christophe Simon, Emmanuelle Jousselin, Jean-Yves Rasplus

**Affiliations:** 1 INRA, UMR 1062 CBGP (Centre de Biologie pour la Gestion des Populations), Montferrier-sur-Lez, France; 2 INRA, UMR IGEPP (Institute of Genetics, Environnement and Plant Protection), Le Rheu, France; Institut National de la Recherche Agronomique (INRA), France

## Abstract

Aphids constitute a diverse group of plant-feeding insects and are among the most important crop pests in temperate regions. Their morphological identification is time-consuming and requires specific knowledge, training and skills that may take years to acquire. We assessed the advantages and limits of DNA barcoding with the standard *COI* barcode fragment for the identification of European aphids. We constructed a large reference dataset of barcodes from 1020 specimens belonging to 274 species and 87 genera sampled throughout Europe and set up a database-driven website allowing species identification from query sequences.

**Results:**

In this unbiased sampling of the taxonomic diversity of European aphids, intraspecific divergence ranged from 0.0% to 3.9%, with a mean value of 0.29%, whereas mean congeneric divergence was 6.4%, ranging from 0.0% to 15%. Neighbor-joining analysis generated a tree in which most species clustered in distinct genetic units. Most of the species with undifferentiated or overlapping barcodes belonged to the genus *Aphis* or, to a lesser extent, the genera *Brachycaudus, Dysaphis and Macrosiphum.* The taxa involved were always morphologically similar or closely related and belonged to species groups known to present taxonomic difficulties.

**Conclusions:**

These data confirm that *COI* barcoding is a useful identification tool for aphids. Barcode identification is straightforward and reliable for 80% of species, including some difficult to distinguish on the basis of morphological characters alone. Unsurprisingly, barcodes often failed to distinguish between species from groups for which classical taxonomy has also reached its limits, leading to endless revisions and discussions about species and subspecies definitions. In such cases, the development of an effective procedure for the accurate identification of aphid specimens continues to pose a difficult challenge.

## Introduction

Aphids (Hemiptera: Aphididae) constitute a diverse group (about 4800 species [1]) of plant-feeding insects. They occur mostly in temperate regions and European aphids account for one third of the world’s fauna, with approximately 1400 species [2]. The intricate life cycles of aphids and their close association with their host plants, polyphenism and ability to reproduce both asexually and sexually make these insects interesting systems for studying many issues in evolution and ecology [3], but they also make species identification challenging.

Furthermore, aphids are among the most serious agricultural pests of temperate regions [4]. In addition to causing direct damage by feeding on phloem, they also act as vectors of many plant viruses [5],[6]. Aphids are small insects that are often transported around the globe, constituting an invasive threat to native and cultivated plants [2],[7]. The Aphididae is the insect family containing the largest number of invasive alien species introduced into Europe [8]. Aphids can cause very severe direct and indirect damage to crops. For example, introductions of *Aphis glycines* Matsumura, 1917, *Toxoptera citricidus* (Kirkaldy, 1907) and *Diuraphis noxia* (Kurdjumov, 1913) into North America have resulted in crop losses amounting to millions of dollars [7].

The reliable identification, to species level, of all developmental stages of aphids is critical for improvements in border controls and biomonitoring and for the success of integrated pest management strategies. However, the routine morphological identification of aphids is time-consuming and requires specific knowledge, training and skills that may take years to acquire. The accurate identification of aphids is difficult, because many species are morphologically similar and complexes of ecologically contrasting taxa frequently occur [9],[10],[11],[12]. Morphological identification is also hampered by the high level of intraspecific variation. Indeed, the range of continuous morphological variation is probably wider in aphids than in any other insect group [13]. The presence of different morphs on different host plants and at different periods of the year further complicates species identification [1]. Finally, for several genera (e.g. *Aphis, Dysaphis*), identification on the basis of morphological characters alone is often impossible and a knowledge of host-plant association is required for accurate species identification [14],[15]. For the genus *Aphis*, no taxonomist has yet succeeded in writing a comprehensive dichotomous morphological key that effectively separates all the species of a local fauna [15]. In this genus, some species can be identified on the basis of one easily distinguishable morphological character, but many are grouped within morphological entities known as “species groups”. These “species groups”, which have no taxonomic validity, bring together species that are difficult to tell apart morphologically [16]. In practice, there are two ways to identify these difficult taxa: i) the use of morphological characters to identify the “species group” to which the specimen belongs, followed by the use of host-plant association criteria to define the nominal species, ii) initial identification of the host plant, followed by the checking of morphological criteria against a list of associated aphid species (if available) to identify the specimen [15],[17]. With this approach, only specimens for which an accurate host-plant association is available can be correctly identified. The morphological identification of winged morphs is reliable for only a fraction of the specimens caught in traps [15]. Furthermore, correct identification requires the taxonomist to have expertise in both entomology and botany.

The development of a reliable molecular tool based on sound taxonomic knowledge would therefore facilitate aphid identification by non-specialists (i.e. non-taxonomists) using aphids as model systems for their studies. This tool would also be useful for biomonitoring programs (such as that based on suction trap networks operating in Europe, see EXAMINE http://www.rothamsted.ac.uk/examine), for which the fast and accurate identification of large numbers of aphid individuals is required and in which winged morphs are captured.

DNA barcoding with the 5′-terminal fragment of the mitochondrial cytochrome C oxidase subunit 1 gene (*COI*) [18] has proved to be an effective standardized approach for the characterization of diverse organisms [19], including insects [20]. Most DNA barcoding-based studies in aphids have involved comparisons of small numbers of economically important species [21],[22],[23],[24]. Only a few recent studies have included relatively large numbers of aphid species. Wang *et al.* [25] focused on subtribe Aphidina, a difficult group, and two studies have demonstrated the utility of DNA barcoding for the identification of specimens from the large regional aphid fauna of North America [1],[26] and Korea [27]. However, the accuracy of DNA barcoding for the identification of European aphids has never before been assessed.

Here (i) we present the first European aphid barcode database including a large number of species (274), (ii) we discuss the usefulness, accuracy and limitations of this database for identifying European aphids and (iii) we introduce a database-driven website including taxonomic and biological data and images and allowing the identification of species through BLAST sequence comparisons with a query sequence.

## Materials and Methods

### Ethics Statement

No permission was required for sampling at the sites studied. This study involved no endangered or protected species.

### Taxonomic Sampling

Specimens were collected between 1997 and 2008. They were killed and preserved in 70% ethanol, at 2°C. The DNA extraction process was destructive, so we selected vouchers from other specimens from the same colony (i.e. sampled on the same host plant at the same time). Voucher specimens were mounted on microscope slides and deposited in the Aphididae collection of the Center for Biology and Management of Populations (CBGP) at Montferrier-sur-Lez, France. Specimens were identified to species level by the first author. Taxonomy and nomenclature were as described by Remaudière and Remaudière [28], Nieto Nafria *et al.* [29], Eastop and Blackman [30] and Favret [31]. For nine samples, identification to species level was not possible, although the morphological characters of these specimens clearly indicated that they belonged to different species. In these cases, species names were replaced by “sp.”, followed by the sample code.

Comprehensive lists of all the specimens included in the study, with voucher numbers, sampling and taxonomic data, are provided in Supporting Information Table S1 and Table S2 and are available in the ACEA project in BOLD (http://www.barcodinglife.org).

### DNA Extraction, Amplification and Sequencing

DNA was isolated from single individuals with the Qiagen DNeasy or ZyGem extraction kit, according to the standard protocol recommended by the manufacturer. DNA was recovered in 50 µl of purified H_2_O. The cytochrome c oxidase I gene was amplified with either LepF (5′-ATTCAACCAATCATAAAGATATTGG-3′) (forward) and LepR (5′-TAAACTTCTGGATGTCCAAAAAATCA-3′) (reverse) [32] primers or with LCO1490 (5′- GGTCAACAAATCATAAAGATATTGG-3′) (forward) [33] and a degenerate reverse primer HCO2198-puc (5′-TAAACTTCWGGRTGWCCAAARAATC-3′) [34] if amplification with the first primer pair failed. The 25 µl PCR mixtures contained 1 X Qiagen® enzyme buffer (containing 1.5 mM MgCl_2_), 1 unit of *Taq* polymerase, 17.5 pmol of each primer, 25 nM of each dNTP and 2 µl of DNA extract. Samples were subjected to initial denaturation at 94°C for 3 minutes, followed by 30 cycles of 30 s at 94°C, 1 minute at 48°C and 1 minute at 72°C, before a final elongation for 10 minutes at 72°C.

PCR products were purified by treatment with exonuclease I and phosphatase and sequenced directly with the Big Dye Terminator V3.1 kit (Applied Biosystems) and an ABI3730XL sequencer at Genoscope, Evry, France. Contigs were assembled from forward and reverse reads and corrected with GENEIOUS V3.7 sequence editing software [35].

The same software was used to align the sequences, and the alignment was translated into an amino-acid sequence with MEGA ve.5 software [36], which was used to detect frameshift mutations and premature stop codons potentially indicative of the presence of pseudogenes.

We tried to obtain complete sequences (658 bp) with no ambiguous nucleotides for any specimen, to establish a valuable reference database. We therefore repeated PCR and sequencing for all sequences that were incomplete or contained ambiguous base pairs. All sequences were deposited in GenBank (KF638720 to KF639739) and are also available from the PhylAphidB@se website (http://aphiddb.supagro.inra.fr) and from BOLD (http://www.barcodinglife.org).

### Data Analyses

We first evaluated the extent to which our database was representative of the known European fauna [37]. We then compared the number of haplotypes obtained with the number of specimens sequenced per species. We also constructed frequency histograms of pairwise genetic distance values at three levels: between specimens from the same species (intraspecific), between species from the same genus (congeneric) and between species from different genera (intergeneric). The distribution of pairwise distances and associated statistical values may be biased by the uneven sampling of different taxa (e.g. intensive sampling of a few species and the overrepresentation of a few haplotypes), so we repeated our analyses, taking into account a maximum of two specimens per haplotype for each species, which is equivalent to considering haplotype diversity instead of haplotype frequency.

For each species, we also plotted maximum within-species divergence (Max-WSD i.e. maximum intraspecific divergence) against minimum between-species divergence (Min-BSD, i.e. minimum interspecific divergence) to detect incidences of misleading barcode-based assignment (Max-WSD≥Min-BSD). Pairwise nucleotide sequence divergences were calculated with a Kimura two-parameter model of base substitution [38], using the “pairwise-deletion” option. This distance is commonly used in DNA barcoding studies, making it possible to compare our results with those of many other published studies, including previous studies on aphids. The R (v.2.15.0) packages ape 3.0 [39] and Spider 1.1–2 [40] were used for all analyses and for the creation of graphical illustrations.

Finally, neighbor-joining (NJ) trees were reconstructed on the basis of the same evolutionary model, to provide a graphical representation of the phenetic distance matrix. We performed a bootstrap test of node support, with 500 replications, with MEGA version 5 [36]. Trees were edited with TreeDyn (v.198.3) software [41].

### Database and Website

A database was constructed with BioloMICS Software (www.Bio-Aware.com) [42], to manage all arthropod specimens, including aphids, hosted by the Center for the Biology and Management of Populations (CBGP, France). This database includes taxonomic and collection information, the DNA sequences available for each specimen, photographs and host-plant associations, when relevant. The BioloMICS Net Module was used to create the PhylAphid database (PhylAphid@base, available from http://aphiddb.supagro.inra.fr) dedicated to aphid specimens. The pairwise sequence alignment function embedded in BioloMICS Software is implemented in PhylAphid@base as an identification tool. The Fauna Europaea [37] and Aphid Species Files V.5.0 [31] were used as references for aphid species names. The ISO 3166 standard published by the International Organization for Standardization (ISO) was chosen as the reference for geographic information. ISO 3166-1 defines codes for the names of countries, dependent territories and special areas of geographical interest. ISO 3166-2 defines codes for identifying the principal subdivisions (e.g., provinces or states) of all countries represented in ISO 3166-1. Plant nomenclature was as in *The Plant List* V.1 [43].

## Results

### Representativeness of the Dataset

**Figure 1:**
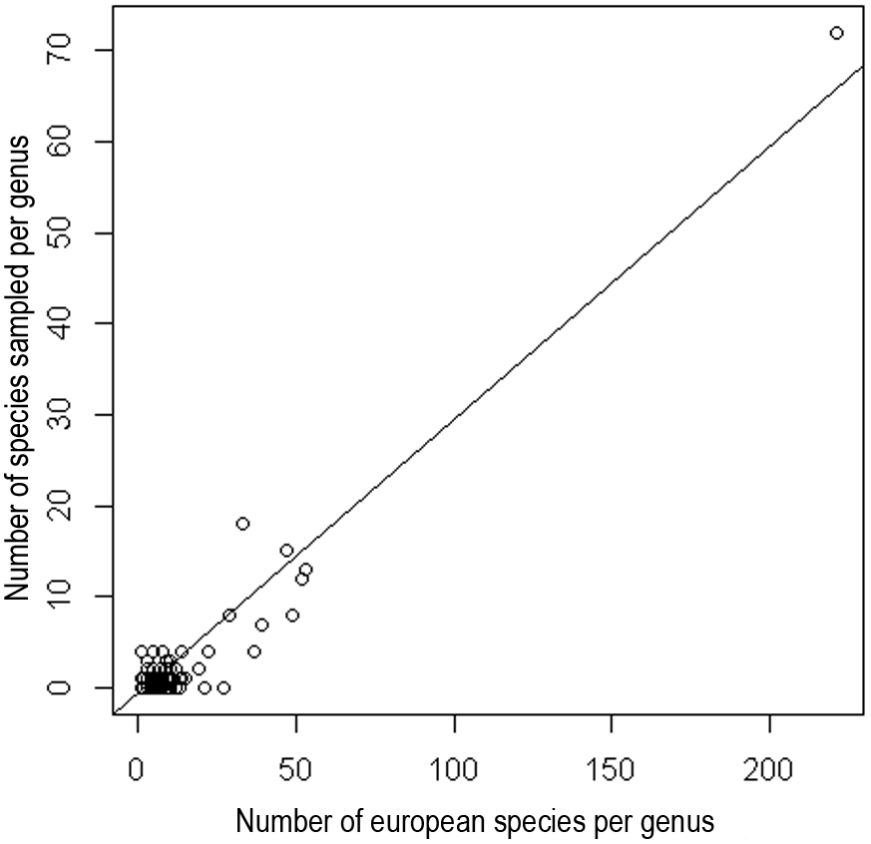
Taxonomic representativeness of our dataset. Linear regression of the number of species per genus sampled in this study against the number of known species per genus in Europe.

Our complete dataset included 1020 samples, from 274 species (20% of all European species), 87 genera (38.5% of the genera present in Europe) and 11 subfamilies (Table 1). All European subfamilies with more than five species were represented, with the exception of Saltusaphidinae. The number of species sampled per genus was significantly correlated with the number of species from the aphid genus concerned known to be present in Europe (Figure 1; R^2^ = 0.9562, t = 48.9198, df = 224, *p*-value<0.001). Our dataset may therefore be considered to correspond to an unbiased sample of the taxonomic diversity of European aphids. *Aphis*, the genus with the largest number of species in Europe, was slightly oversampled, but only five genera containing more than 10 European species were not represented in our dataset: *Microsiphum* (10 European species), *Xerobion* (12), *Eulachnus* (13), *Coloradoa* (21) and *Schizaphis* (27). Most specimens were sampled in France (730), Greece (148) and Italy (112). A few were collected in the United Kingdom (24) and Serbia (6) (Table S1).

**Table 1:** Representativeness of the sampling analyzed in our study: Numbers of genera and species included in our dataset, known in Europe and occurring worldwide are reported for each aphid subfamily.

	N taxa in dataset*		N taxa in Europe		N taxa worldwide	
Subfamilies	Genera	Species	Genera	Species	Genera	Species
Anoeciinae	1	1	1	14	1	24
Aphidinae	51	198	124	946	337	2860
Calaphidinae	15	23	29	92	91	356
Chaitophorinae	3	14	7	62	11	178
Drepanosiphinae	1	2	3	7	5	37
Eriosomatinae	8	10	24	84	60	369
Greenideinae	1	1	1	1	16	173
Lachninae	4	19	12	98	18	397
Phyllaphidinae	1	1	2	2	2	14
Pterocommatinae	1	3	4	14	5	57
Thelaxinae	1	2	2	6	4	19
**Subfamilies not sampled**						
Hormaphidinae	0	0	3	5	41	197
Israelaphidinae	0	0	1	4	1	4
Lizerinae	0	0	1	1	3	34
Mindarinae	0	0	1	2	1	9
Neophyllaphidinae	0	0	1	1	1	12
Phloeomyzinae	0	0	1	1	1	2
Phyllaphidinae	0	0	2	2	2	14
Saltusaphidinae	0	0	10	33	12	71

*A full list of the materials analyzed and associated data are available in Supporting Information Table S1 and Table S2. Classification is as for Remaudière and Remaudière [28] and Nieto Nafria *et al.* [29]. European data were provided by Fauna Europea (http://www.faunaeur.org/) [37], and world data were provided by Foottit *et al.* [1].

After several rounds of PCR amplification and sequencing of *COI*, only four of the 1020 barcodes still contained ambiguous bases at either the 5′- (specimens ACOE1772, ACOE1982, ACOE1007) or 3′- (specimen ACOE1586) end. As these specimens were singletons, their incompleteness had little impact on the analysis and we left them in the dataset. Alignment was straightforward, due to a lack of sequence length variation and an absence of stop codons and frameshifts, suggesting that our dataset contained no NUMts.

**Figure 2:**
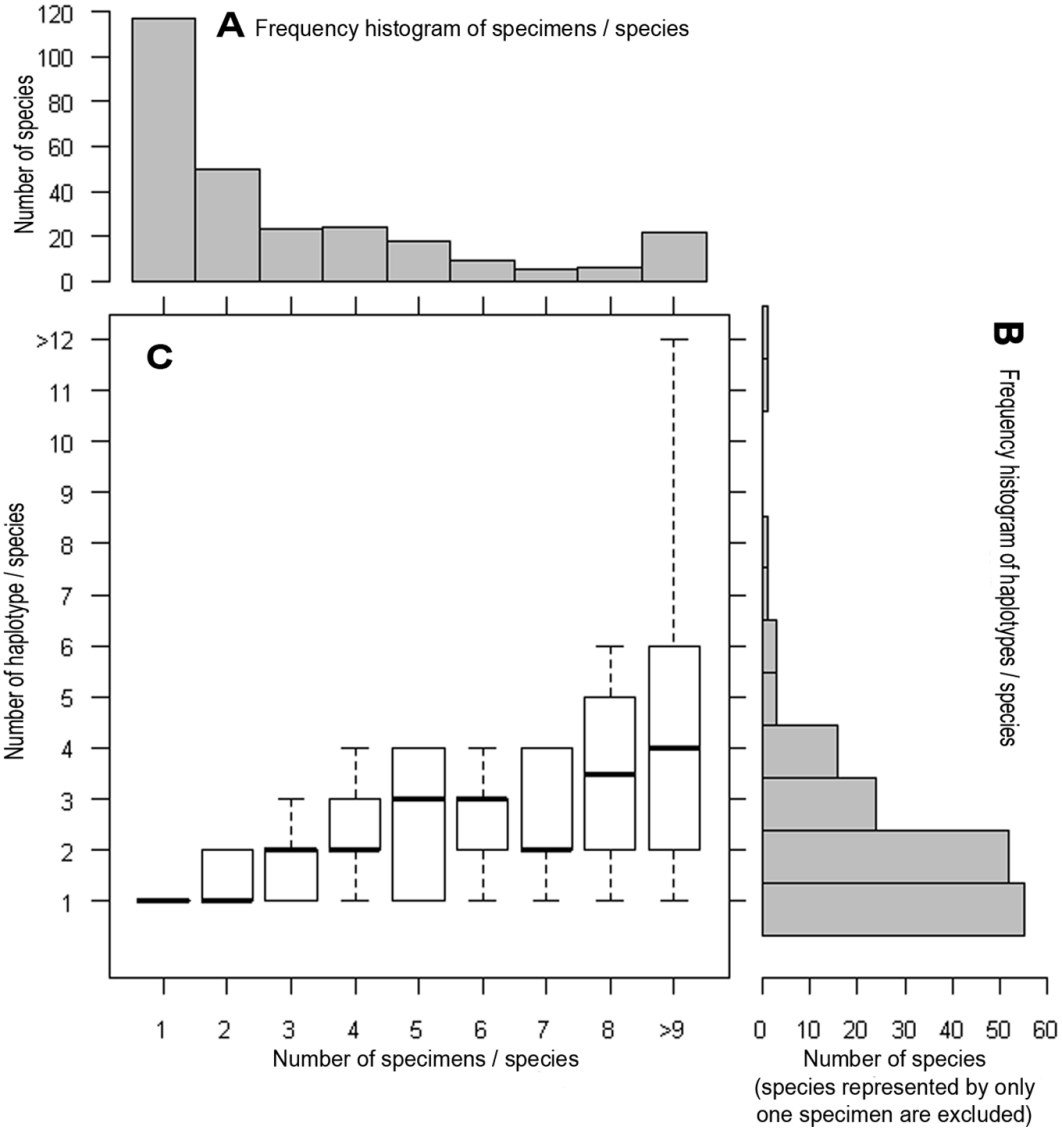
Intraspecific representativeness of our dataset. Frequency histograms of specimen numbers (A), number of haplotypes per species (B) and changes in the number of haplotypes with respect to the number of specimens sampled per species (C; box and whisker plot with the bottom and top of the boxes representing the 25th and 75th percentiles, respectively, bands near the middle of the boxes representing the medians and the ends of the whiskers representing the 10th and 90th percentiles).

We obtained a mean of 3.7 barcode sequences per species, with 58% of the species represented by at least two barcodes and 40% represented by at least three barcodes (Figure 2 A, Table S3). Three species were densely sampled: *Aphis fabae* (96 specimens), *Brachycaudus helichrysi* (42) and *Aphis craccivora* (23) (Table S3).

**Figure 3:**
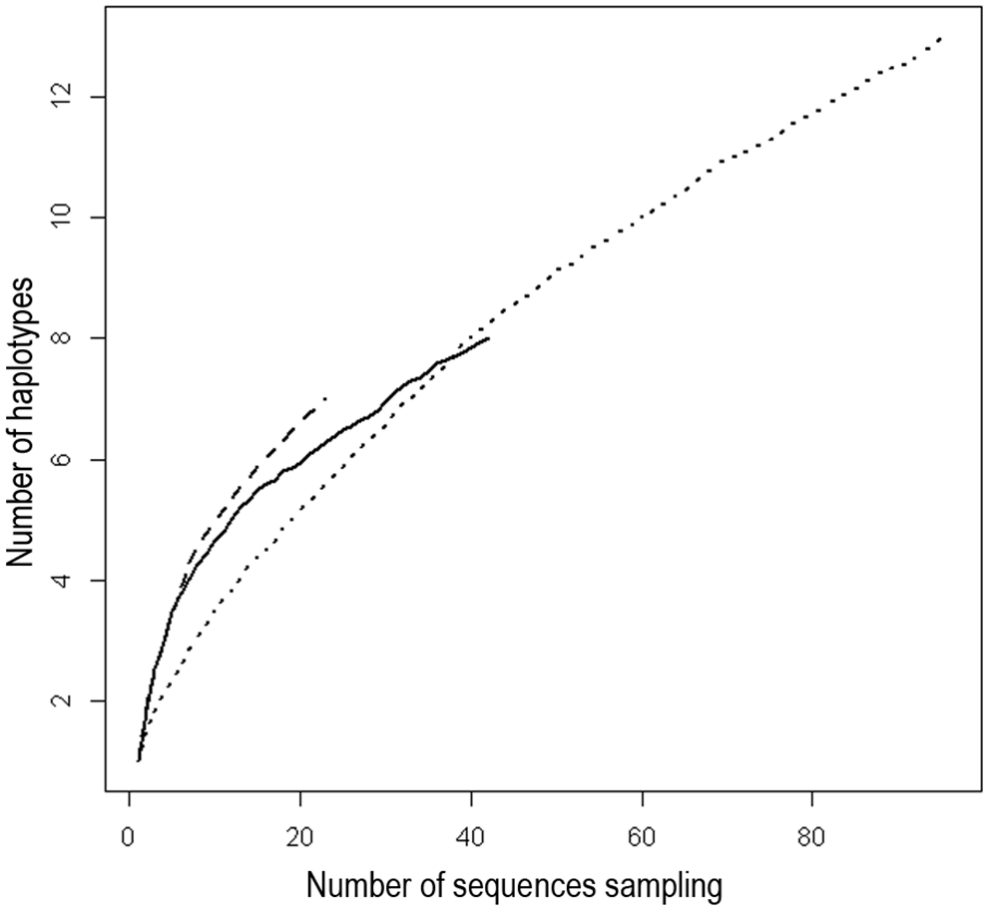
Haplotype accumulation curves. The curves represent the mean number of haplotypes accumulated through random permutations (subsampling of sequences) for *Aphis fabae* (dotted line), *A. craccivora* (dashed line) and *Brachycaudus helichrysi* (solid line).

We found 457 haplotypes among the 1020 barcodes. Some species had the same haplotype and a given haplotype could be common to two to eight species (see species with a min-BSD of 0 in Table S3). If we excluded species represented by a single specimen, the number of haplotypes per species ranged from 1 to 13 (Figure 2 B, Table S3), with a mean of 2.3 haplotypes per species. This mean number increased with the number of specimens sampled (Figure 2 C), although haplotype accumulation curves never reached the asymptote, even for the three most heavily sampled species (Figure 3). Haplotype numbers increased rapidly with the number of specimens sampled per species (R = 0.7149, t = 12.7304, DF = 155, *p*-value = ***), but they increased less rapidly with mean and maximum intraspecific distances (R = 0.36, t = 4.78, df = 155, *p*-value = *** and 0.51, t = 7.45, df = 155, *p*-value = *** respectively). Thus, greater intraspecific sampling results in greater haplotype diversity but has no major effect on intraspecific genetic distances.

### Intra- and Interspecific Divergences

**Figure 4:**
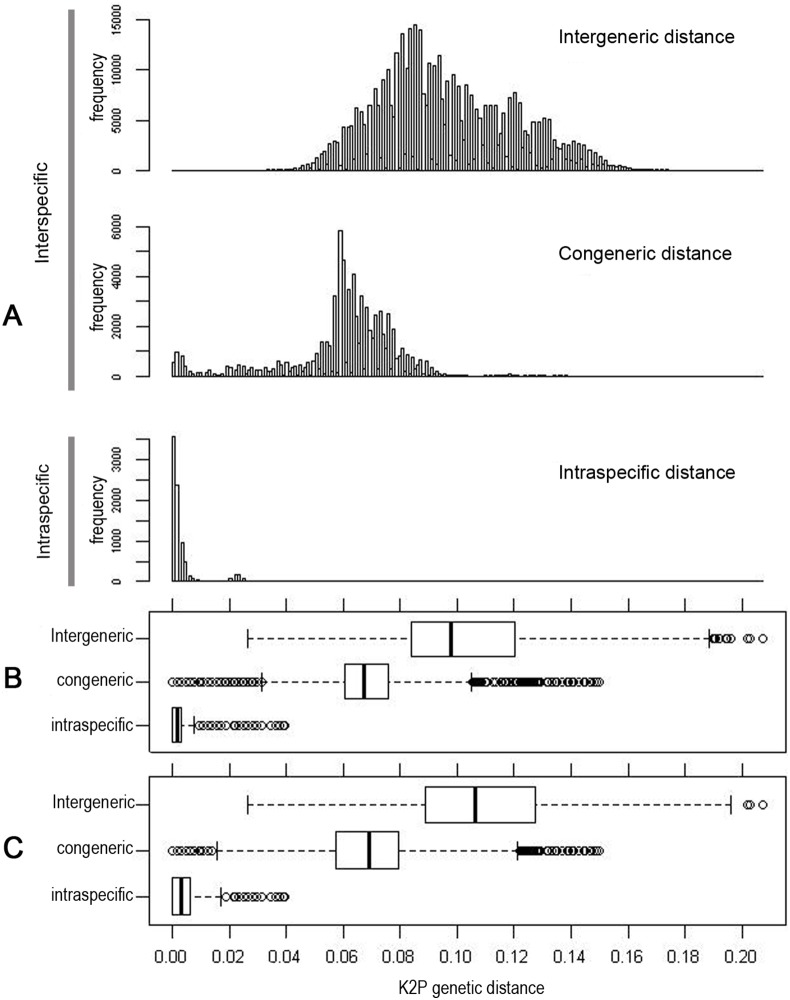
Distribution of pairwise K2P distances among 1020 specimens of aphids, based on *COI* sequences. Graphs A and B include all pairwise comparisons, graph C includes each pair of haplotypes only once. On the box and whisker plots in B and C, the bottom and top of the boxes represent the 25th and 75th percentiles, respectively, the bands near the middle of the boxes represent the median, the ends of the whiskers represent the 2.5^th^ and 97.5^th^ percentiles and dots represent the outliers beyond 95% of the distribution.

Frequency histograms of pairwise genetic distances (Figure 4 A) showed that there were (i) no gaps between congeneric and intergeneric distances, (ii) a gap between intraspecific and intergeneric distances, (iii) a slight overlap between intraspecific and congeneric distances.

Intraspecific divergences (8205 pairwise comparisons) ranged from 0.0% to 3.9%, with a mean value of 0.29%, a median of 0.15% and a 95% confidence interval (CI) of 0.0–0.7% (Figure 4 B).

The average divergence in the 515571 interspecific comparisons was 9.5% (median = 9.2%, ranged = 0.0% to 20.7%). These comparisons included 73473 congeneric pairwise comparisons with a mean divergence of 6.4% (median = 6.7% range = 0.0% to 15% with a 95% CI = 3.1–10.5% (Figure 4 B)). The remaining 442098 intergeneric pairwise comparisons had a mean divergence of 9.8% (range = 2.6% to 20.7% with a 95% CI = 2.6 to 18.9% (Figure 4 B)). Exclusion of the outliers from the pairwise distance distribution (dots on Figure 4 B) resulted in a gap between intraspecific and interspecific (intergeneric + congeneric) genetic distances, with no overlap of their distribution curves between 0.7% and 2.6%.

If we considered a maximum of two specimens per haplotype, the mean intraspecific divergence (421 pairwise comparisons) was 0.45% (median = 0.3% with a 95% CI of 0.0–1.7% (Figure 4 C)) and the mean interspecific divergence (87594 comparisons) was 10.37% (median = 10.27% with a CI of 0.46–20.3%). The mean congeneric divergence was 6.5% (median = 6.9% with a 95% CI of 1.5–12.1% for 8756 pairwise comparisons) and the mean intergeneric divergence was 10.8% (median = 10.6% with a 95% CI of 2.6 to 19.6% for 78838 pairwise comparisons).

Following the exclusion of outlier values (dots on Figure 4 C), the intraspecific divergence distribution overlapped with the congeneric divergence distribution between 1.5% and 1.7%, whereas the gap between intraspecific and intergeneric divergence remained.

**Figure 5:**
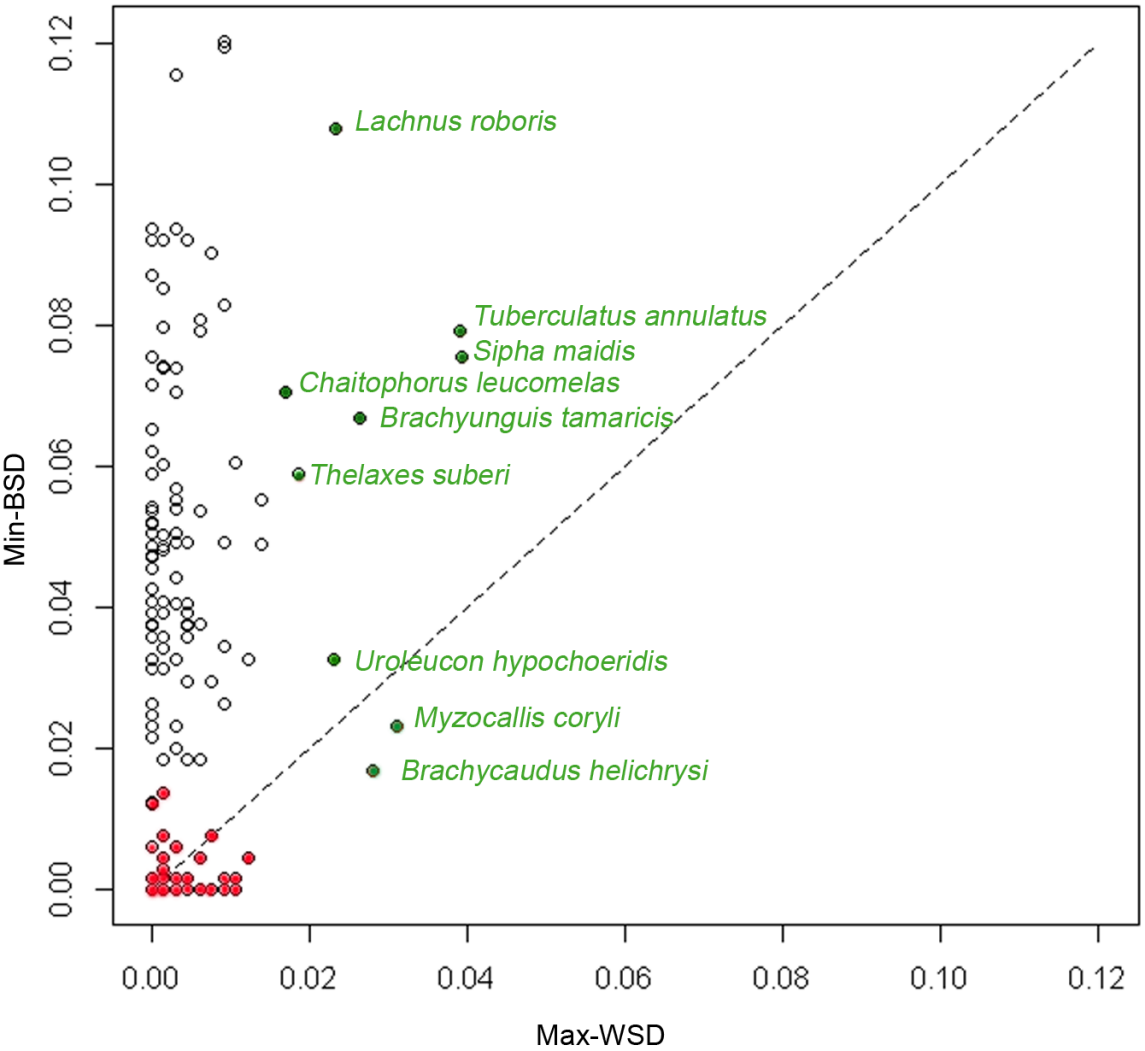
Patterns of *COI* divergence for 155 species represented by at least two individuals. For each nominal species, minimum between-species divergence (Min-BSD) is plotted against maximum within-species divergence (Max-WSD). Points above the diagonal correspond to cases in which species identification is straightforward. Colored dots represent nominal species detected as outliers in the species divergence distribution. Green dots represent the species with high levels of intraspecific divergence; red dots represent species with exceptionally low levels of interspecific genetic divergence. Distances are calculated with a K2P model of base substitution.

The outliers in the intraspecific divergence distribution, with exceptionally high intraspecific divergences, included nine species: *Tuberculatus annulatus, Myzocallis coryli, Brachycaudus helichrysi, Chaitophorus leucomelas, Sipha maydis, Lachnus roboris, Thelaxes suberi, Brachyunguis tamaricis* and *Uroleucon hypochoeridis* (Figure 5). The outliers in the congeneric divergence distribution with exceptionally low levels of interspecific divergence included 73 species (species with Min-BSD <1.5% in Table S3).

### Species Identification through the Exploration of Genetic Variation within and between Species

Species assignment was correct for 85% of the 274 species included in our dataset. Species represented by a single specimen (*n* = 119) had distinct haplotypes, and those represented by more than one specimen (155) had a Max-WSD value that was smaller than min-BSD (Figure 5, Table S3). NJ analysis generated a tree (Figure S4) in which most species formed distinct genetic units; 77.8% of the species represented by several specimens were recovered as monophyletic units, 95% of which were supported by a bootstrap value (BP) >80%.

A misleading barcode-based assignment to a particular species could occurs when the maximum sequence divergence among individuals belonging to one species (max-WSD) equals or exceeds the minimum sequence divergence with another species (min-BSD) (Hajibabaei *et al.,* 2006). In our dataset, this situation was encountered for 41 species (dots below the diagonal on Figure 5, species shown in bold in Table S3). Two of these species, *Brachycaudus helichrysi* and *Myzocallis coryli* (green dots in Figure 5) were previously identified as species with exceptionally high levels of intraspecific divergence. In the NJ tree (Figure 6.4), specimens of *B. helichrysi* were segregated into two well supported clades (BP = 100) (containing 16 and 26 specimens, respectively). *B. helichrysi* was rendered paraphyletic by one specimen of *B. spiraeae* (Figure 6.4) branching with a high BP value (88) as a sister group to one of the clades. The high degree of intraspecific divergence observed for *Myzocallis coryli* (Figure 6.1) was due to a single specimen, which diverged strongly from the other representatives of the species. Species paraphyly was due to a single specimen of *Myzocallis carpini* branching within one clade of *M. coryli* with a low BP value (BP<50).

**Figure 6:**
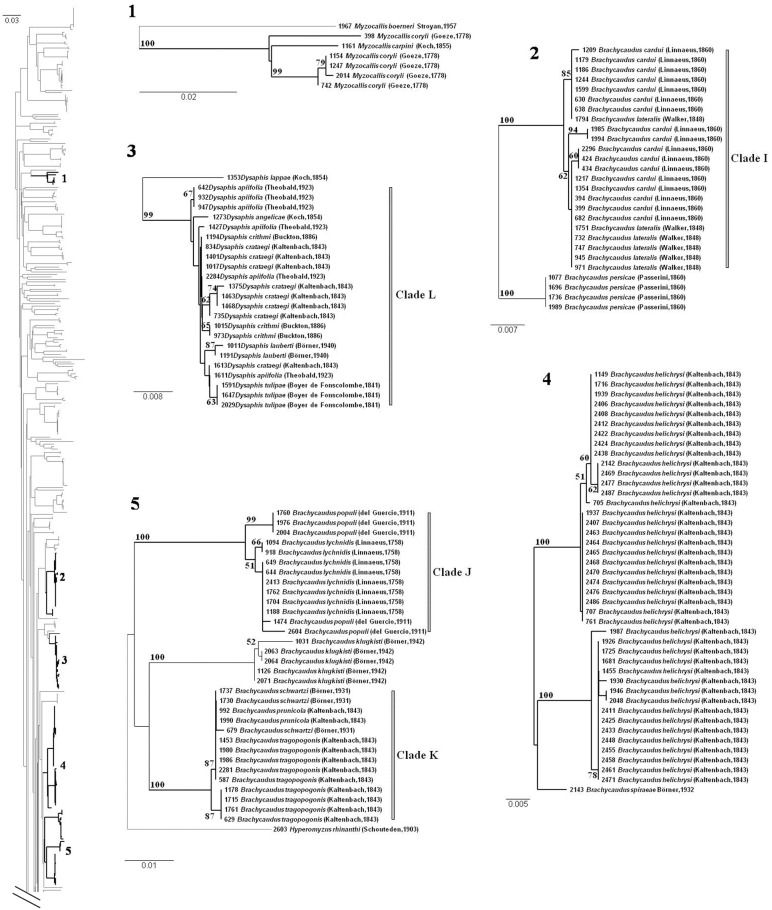
Focus on some problematic clades for barcode assignment. See Figure S4 for the complete NJ tree. Identification numbers of each clade are reported on the tree silhouette. Bootstrap support values >50 are provided. Note that the scale of genetic K2P divergence differs between subtrees.

**Figure 7:**
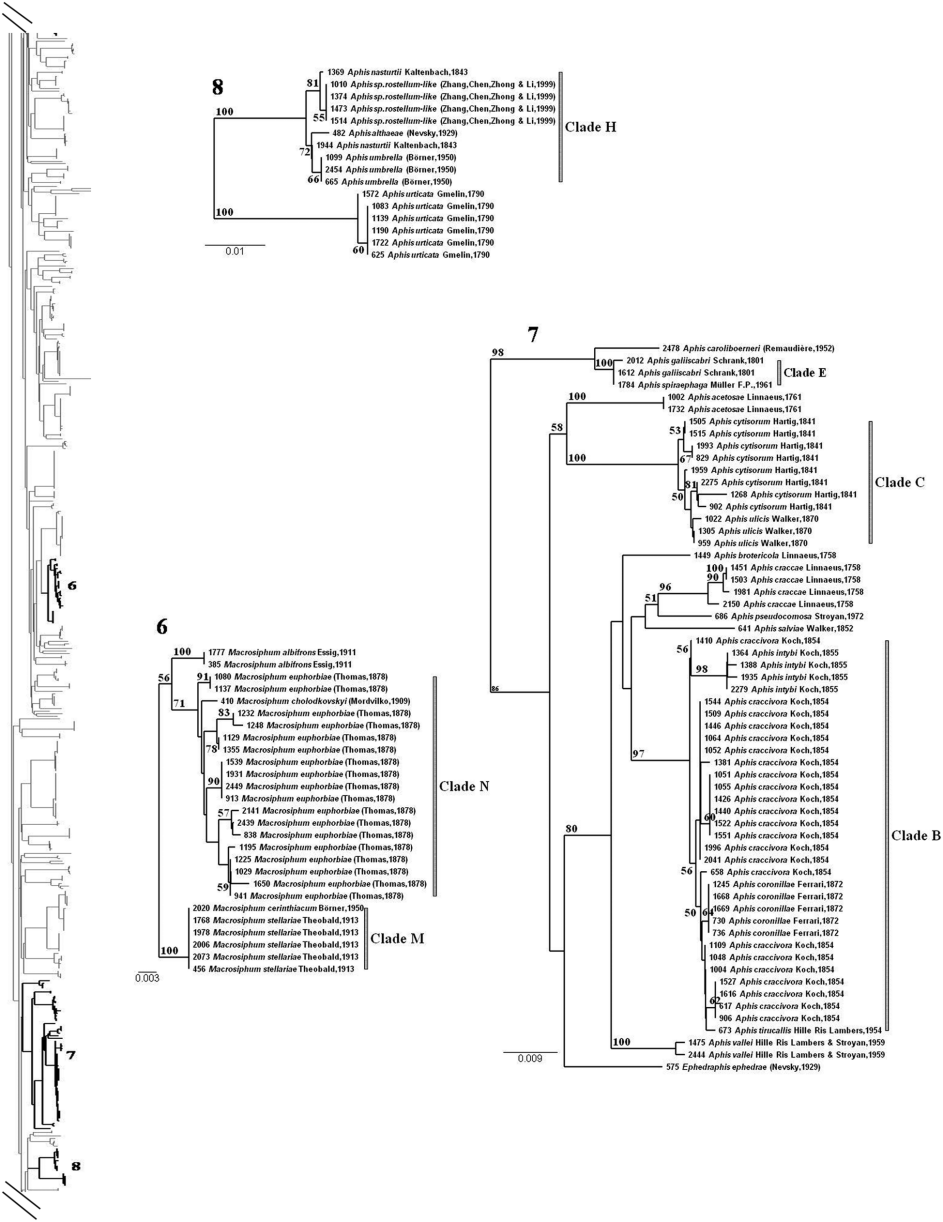
Focus on some problematic clades for barcode assignment (following on from Figure 6). See Figure S1 for the complete NJ tree. The identification numbers of each clade are reported on the tree silhouette. Bootstrap support values >50 are indicated. The scale of genetic K2P divergence differs between subtrees.

**Figure 8:**
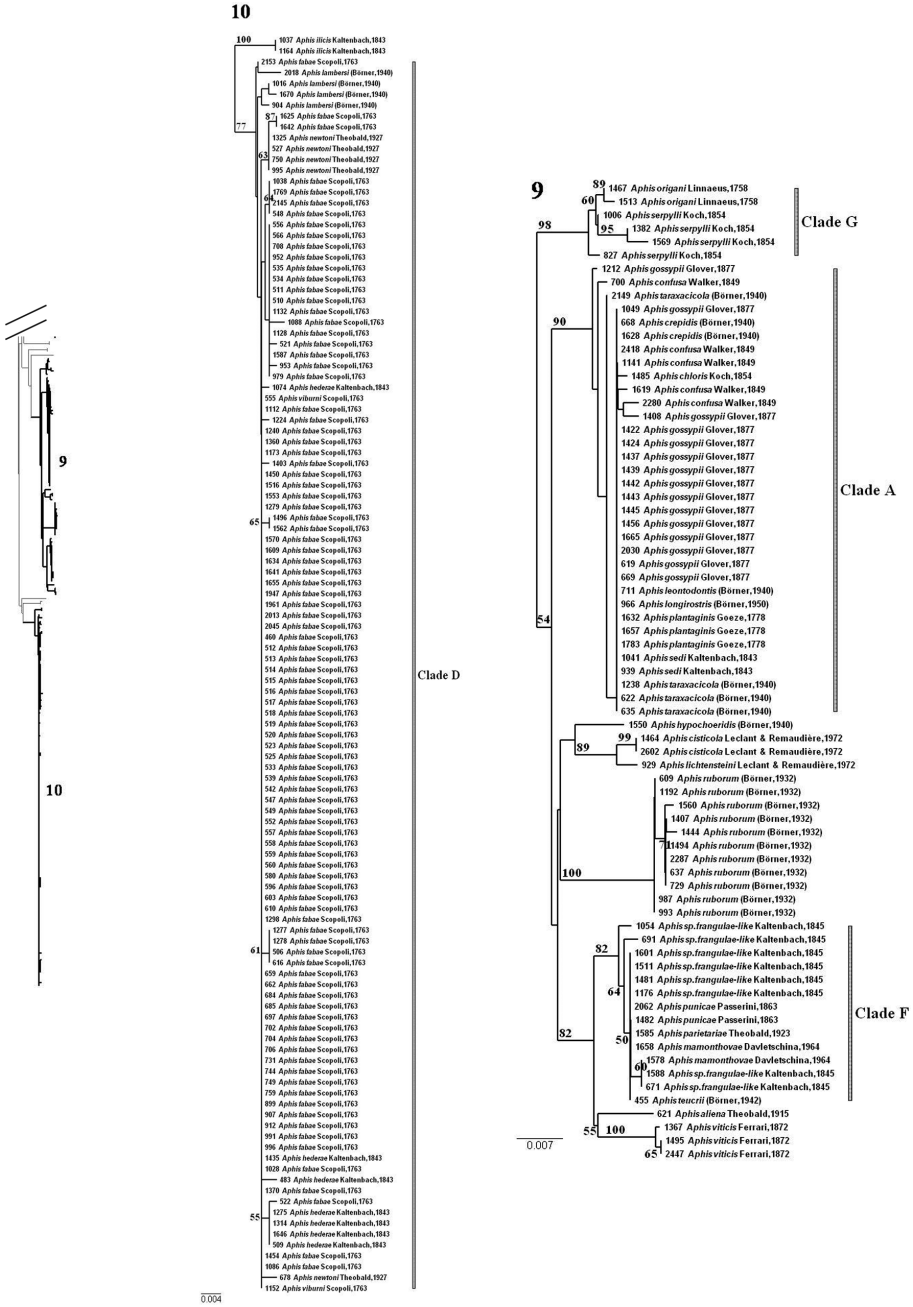
Focus on some problematic clades for barcode assignment (following on from Figure 7). See Figure S1 for the complete NJ tree. The identification numbers of each clade are reported on the tree silhouette. Bootstrap support values >50 are indicated. The scale of genetic K2P divergence differs between subtrees.

The other 39 species, with a max-WSD≥min-BSD (Table S3, red dots in Figure 5), had previously been identified as species with exceptionally low levels of interspecific genetic divergence and low levels of intraspecific divergence, but within the normal distribution for aphid species. Most of these species with undifferentiated or overlapping barcodes belonged to the genus *Aphis* (26 species), or, to a lesser extent, the genera *Brachycaudus* (*n* = 7), *Dysaphis* (*n* = 3) and *Macrosiphum* (*n* = 3). Twenty-eight of these species had a haplotype in common with another species (see Min-BSD value = 0 in Table S3), always from the same genus. In the NJ tree, these 39 species belonged to 14 clades encompassing a total of 50 species (Table 2) and were characterized by short internal branches, low levels of internal node resolution and, except for one species, high BP values (BP>80) (Figure 6.2 to Figure 6.3 and Figure 6.5; Figure 7, Figure 8). The min-BSD values within each clade (mean: 0–0.69%, range: 0–1.23%) were within the range of intraspecific divergence for aphids and, for each clade (except for cluster N), these values were clearly below the min-BSD with the sister species of the clade (Table 2).

**Table 2:** Clusters of nominal species poorly discriminated by *COI* barcodes.

Clade	Species (number of specimens)	Min-BSD (%)		BP-Value	Min-BSD (%) with closest species
		Range	Mean		
A	*A. chloris(1)*, *Aphis confusa (5)*,*A. crepidis (2)*, *A. gossypii (15)*,*A. leontodontis (1)*, *A. longiristris (1)*,*A. plantaginis (3)*, *A. sedi (2)*,*A. taraxacicola (4)*	0–0.61	0.09	90	1.70
B	*A. coronillae (5), Aphis craccivora (23)*, [*A. intybi* (4)], *A. tirucallis (1)*	0.15–1.23	0.64	97	2
C	*Aphis cytisorum (8)*, *A. ulicis (3)*	0.15–0.76	0.32	100	3.61
D	*Aphis fabae (96)*, *A. hederae* (7)*A. lambersi (4), A. newtoni (5)*, *A. viburni (2)*	0–1.07	0.27	77	1.23
E	Aphis galiiscabri (2), Aphis spiraephaga (1)	0–0.15	0.1	100	1.38
F	*Aphis mamonthovae (2)*,*A. parietariae (1)*, *A. punicae (2)*,*A. frangulae-like (8)*, *A. teucrii (1)*	0–0.46	0.11	82	1.23
G	*Aphis serpylli (4)*, [*A. origani (2)*]	0.15–1.07	0.61	98	1.70
H	*Aphis althaeae (1), A. nasturtii (2),* *A. sp.rostellum-like (4), A. umbrella (3)*	0.15–0.76	0.41	100	5.2
I	*Brachycaudus cardui (17)*,*B. lateralis (6)*	0–0.77	0.22	100	3.6
J	*Brachycaudus lychnidis (8)*,*B. populi (5)*	0.15–0.92	0.69	100	4
K	*Brachycaudus prunicola (2)*,*B.tragopogonis (9)*, *B. schwartzi (3)*	0–0.46	0.15	100	2.96
L	*Dysaphis angelicae (1), D. apiifolia (6)*, *D. crataegi (8)*, *D. crithmi (3)*,[*D. lauberti (2)*], *D. tulipae (3)*	0–1.07	0.41	99	2.64
M	*Macrosiphum cerinthiacum (1)*,M. stellariae (5)	0–0	0	100	0.92
N	*Macrosiphum euphorbiae (18)*,*M. cholodkovskyi (1)*	0.46–0.92	0.65	71	0.92

Means and ranges of genetic distances between the species included in each cluster (BSD) and between the cluster and its closest relative are reported. Bootstrap support (BP) values for each cluster are given. Square brackets indicate monophyletic species in the NJ tree.

More than half these clades (8/14) consisted exclusively of species from the genus *Aphis*. The clade with the highest species richness (A) included nine species (Table 2, Figure 8.9). Eight of these species – *Aphis crepidis*, *A. confusa*, *A. leontodontis*, *A. longisrostrata*, *A. plantaginis*, *A. sedi*, *A. taraxacicola* and *A. gossypii* – had the same haplotype. The single specimen of *Aphis chloris* was nested within this clade. It did not share a haplotype with any other species of the clade, but there was less of a difference between its barcode and those of some other species of the clade (Min-BSD = 0.15%) than between *A. gossypii* haplotypes (Max-WSD = 0.61). Clade A was closely related to another *Aphis* clade including five species (cluster F, Table 2, Figure 8.9): *Aphis mamonthovae*, *A. parietariae*, *A. punicae*, *A. frangulae-like* and *A. teucrii*, all with some identical barcodes in common. A similar pattern was observed in cluster D (Table 2, Figure 8.10), for *Aphis fabae, A. hederae* and *A. viburni*. Two other species – *A. lambersi* and *A. newtoni* – were nested within this clade. Most specimens of each of these species formed a clade, but all included a specimen with a slightly divergent haplotype (ACOE2018 for *A. lambersi* and ACOE678 for *A. newtoni*) more closely related to *A. fabae* than to any other conspecific specimen. *Aphis galiiscabri* and *A. spiraephaga* also included specimens with the same haplotype, clustering in clade E (Table 2, Figure 7.7). In all the other four *Aphis* clades (Table 2, Figure 7.7, 7.8 and Figure 8.9., Clade B–C, G–H), there were four polyphyletic species: *A. craccivora* (in Clade B), *A. cytisorum* (in Clade C), *A. serpylli* (in Clade G) and *A. nasturtii* (in Clade H). Monophyly of the remaining species was generally poorly supported (BP<80%), with the exception of *Aphis intybi* (Clade B) and *A. origani* (Clade G). *Aphis intybi* had a relatively high min-BSD value for an *Aphis* species (0.61). Its placement in clade B resulted from a single specimen of *Aphis craccivora* (ACOE1410) branching at its root. The two specimens of *A. origani* were nested within *A. serpylli* specimens.

Some *Brachycaudus* species also displayed very little, if any interspecific divergence. They were grouped into three clades (I, J, K, on Figure 6.2 and Figure 6.5, Table 2). Barcode sequences did not distinguish *B. cardui* from *B. lateralis* (Clade I), *B. lychnidis* from *B. populi* (Clade J) and *B. tragopogonis*, *B. schwartzi* and *B. prunicola* (Clade K) from one another.

More than half (6/11) the *Dysaphis* species included in our dataset were grouped into a single clade displaying little differentiation (Clade L Table 2, Figure 6.3). Three of these species – *D. apiifolia*, *D. crataegi* and *D. crithmi* – were represented by specimens sharing one haplotype. The single specimen of *D. angelicae* was nested within these polyphyletic species, like *D. tulipae* and *D. lauberti* specimens, although these two species formed monophyletic groups that were either highly (BP = 87 for *D. lauberti*) or poorly (BP = 63 for *D. tulipae)* supported. *Macrosiphum cerinthiacum* and *M. stellariae* could not be distinguished by barcoding, because they share a common haplotype (Clade M, Figure 7.6). Finally, the single specimen of *Macrosiphum cholodkovskyi* was nested within the clade including all haplotypes of *M. euphorbiae*, making this species paraphyletic (Clade N, Table 2, Figure 7.6). Some haplotypes of *M. euphorbiae* diverged considerably from the others (Max-WSD = 1.23, Table S3) and the genetic distance between clade N and its closest relatives (specimens from *Macrosiphum albifrons*) remained low (min-BSD = 0.92, Table 2).

### Database and Website Use

Data for the specimens included in this study have been uploaded to the PhylAphidB@se database and can be accessed online via the following website: http://aphiddb.supagro.inra.fr/.

PhylAphidB@ase queries can be carried out easily with basic or advanced search tools. Detailed information about the records (species, specimens, geography, pictures, taxonomy, molecular data, etc.) are automatically displayed. The locations at which specimens were collected can be visualized with Google Earth maps.

The PhylAphidB@se pairwise sequence alignment tool allows users to run an algorithm similar to Blastn, to align unknown *COI* DNA sequences with the reference sequence in the database. Several pairwise alignment parameters can be modified by the user (e.g. minimum similarity, minimum overlap). The results can be presented as a list of blast hits of decreasing similarity, or as a phenetic tree (several algorithms are available e.g. UPGMA, neighbor-joining). Detailed information about the reference specimens can be obtained by clicking on their IDs either in the list of blast hits or on the leaf of the tree.

By using these online tools (Blast and/or tree reconstruction), users can assign a species name to an unknown *COI* sequence.

## Discussion

DNA barcoding aims to identify species, as accurately as, and faster than a taxonomist. It requires the use of an appropriate DNA marker with an adequate rate of evolution, and the availability of a reference dataset representative of the taxonomic diversity of the group studied. We present here the first large barcoding dataset for European aphids, providing records for 1020 individuals from 274 species. We show that this dataset corresponds to an unbiased sample of the taxonomic diversity of European aphids and provides a useful tool for species identification, at least as useful as an aphid taxonomist, who would not conduct thorough and time-consuming comprehensive studies on each problematic taxon.

### *COI* Variation and its Use for Barcoding in Aphids

The intraspecific and interspecific *COI* divergences obtained for our dataset were of a similar order of magnitude to those for the North American and Korean aphid fauna [1],[27]. However, the mean intraspecific divergence (0.29%, range: 0–3.9%) was slightly higher than the values obtained for the North American (mean: 0.201%, SE 0.004) and Korean (mean: 0.05%; range: 0.00–1.00%) fauna, possibly reflecting differences in the magnitude of sampling efforts rather than differences between the fauna. Indeed, the number of specimens per species was higher in our dataset (1020 specimens/274 species; ratio: 3.72) than for the North American (690/335; ratio: 2.06) and Korean (249/154; ratio: 1.61) datasets. This increase in intraspecific divergence with the number of specimens sampled per species has already been highlighted by several studies [44],[45], although exceptions have been reported [46]. Even for highly sampled species (96 specimens of *Aphis fabae*), haplotype accumulation curves never reached an asymptote. This is consistent with the results of Zhang *et al.* [47] for neotropical butterflies, showing that a sample size of 32 to 618 specimens per species was required to unravel most of the genetic diversity (80%) in simulated cases, and that a sample size of 9.5–216.6 was required for the actual species they were studying. However, increasing levels of genetic diversity does not necessarily affect deeply intraspecific divergence values if haplotypes differ at only a few autapomorphic positions, as appears to be the case in our dataset. If we increased our intraspecific sampling effort, the mean intraspecific divergence would probably increase a little, but would probably remain low.

Aphids are at the lower end of the range of intraspecific divergence found in insect species (0 to 7.64%) [20]. Our values are very close to those recorded for other well studied phytophagous, species-rich groups and families, such as Hesperidae (mean intraspecific K2P divergence 0.17%), Sphingidae (0.43%) and Saturniidae (0.46%) [32].

Interspecific divergences for European congeneric species (mean 6.4%, range 0–15%) were intermediate between those for the Korean fauna (mean 5.84% range 0–14.04%) [27] and for the North American fauna (mean 7.25%, range 0.46–13.1%) [1]. These values are again at the lower end of the distribution of interspecific divergences obtained for congeneric species of insects (the means of 95% of which fall between 2.47 and 21% [20]), and approach those obtained for lepidopteran families (Hesperidae 4.58%, Sphingidae 4.41% Saturniidae 6.02%) [32].

The interspecific divergence distribution overlaps the intraspecific divergence distribution, resulting in the absence of a perfect gap between the two, making it impossible to define a species distance threshold. However, we detected an “imperfect gap” in the distribution (between 0.7% and 2.6%) in our dataset. This made it possible to define an optimal threshold minimizing assignment errors, between these values. The usefulness of this gap is debatable, but its presence, by contrast to the continuous distribution observed for congeneric and intergeneric divergences, suggests that levels of *COI* variation can be used for species delimitation, but not for genus delimitation. This may be due to the rate of evolution of *COI* and/or the fact that species delimitations are more consistent than the definitions of genera in aphids.

### Problematic Species for which Further Taxonomic Studies are Required

The high levels of intraspecific divergences displayed by some nominal species (*Brachycaudus helichrysi, Brachyunguis tamaricis, Chaitophorus leucomelas, Lachnus roboris, Myzocallis coryli, Sipha maydis, Thelaxes suberi, Tuberculatus annulatus, Uroleucon hypochoeridis*) may reflect geographical or biological history (i.e. merged phylogeographic variants or retained ancestral polymorphism) or the existence of sibling taxa that have not yet been described. Even in a group for which extensive taxonomic studies have been carried out, such as aphids [48], there are probably undescribed species and DNA barcoding, allowing the rapid detection of deep intraspecific barcode divergences, may facilitate the choice of interesting species for future taxonomic works [19]. The presence of several sibling taxa has already been suggested for some species displaying large intraspecific divergences in our study. Recent studies on *Brachycaudus helichrysi* with several mitochondrial, nuclear and *Buchnera* symbiont genes and microsatellite markers have highlighted the existence of two specific taxa that have not yet been formally described [12], [49]. The presence of several sibling species within *Lachnus roboris* and *Chaitophorus leucomelas* has also been discussed before. Hille Ris Lambers [50] grouped together several *Lachnus* from various *Quercus* species under the name *L. roboris,* considering morphological variation to be environmentally induced. However, other authors [51], [52] have suggested that *L. roboris* may be a complex of species associated with different host plants and with different karyotypes. One of these species, *L. iliciphilus* (del Guercio, 1909) is considered to be valid [53], although it differs from *L. roboris* mostly in terms of its size, and further confirmation is required [51], [52]. Some of our specimens may belong to this species. Indeed, in the absence of diagnostic morphological or ecological characters, we have adopted a “lumping” approach, grouping our specimens together under the name *L. roboris*. *Chaitophorus leucomelas* is a species with a large geographic distribution that presents different numbers of chromosomes according to its origin. This suggests that there may be sibling species within this taxon [51], [52]. Our results confirm that further investigations, including morphological and genetic studies, are required for these species. However, if we exclude *Brachycaudus helichrysi* and *Myzocallis coryli*, both of which are paraphyletic on our NJ trees, the use of DNA barcodes leads to the correct assignation of query sequences to current species names.

### Disentangling Species Groups in Aphid: Barcoding and Morphology are Subject to the Same Limitations

Overall, 77.8% of the species represented by multiple specimens clustered into distinct clades on the NJ trees for *COI*. These clades were separated from their nearest neighbors, indicating that specimen assignation to species by DNA barcoding should be correct. About 19% of the nominal species appeared to be polyphyletic or paraphyletic with respect to other recognized species (including *B. helichrysi* and *M. coryli,* as previously discussed). Situations in which the distances between congeneric species are extremely small are problematic. We detected 14 polyphyletic clades of nominal species poorly discriminated by *COI* barcodes. These clades included a total of 50 aphid species belonging to four (*Aphis*, *Dysaphis*, *Brachycaudus*, and *Macrosiphum*) of the 87 genera represented in our dataset. These genera are known to contain taxonomically problematic species groups formed by species that are morphologically very similar. A detailed taxonomic discussion of each of these species groups and the match between taxonomic divisions and our DNA barcoding data is provided in the supplementary material (Text S1). In summary, eight of the 14 problematic clades cluster specimens from the genus *Aphis* and almost half the *Aphis* species appear to be problematic for identification to the species level by barcoding. This is not surprising given the findings of taxonomic studies on *Aphis*. *Aphis* is the largest aphid genus [54] and contains several of the most damaging aphid pests. It is also the genus most recalcitrant to any comprehensive taxonomic treatment [15]. Most species can easily be classified into species groups forming morphologically well-defined entities, but many of the species within these groups are difficult to tell apart morphologically and identification keys remain ambiguous and are mostly based on host-plant associations [55]. In a few cases, DNA barcode sequences are useful for differentiating between species that are often confused because of their morphological similarities, such as *Aphis pomi* and *A. spiraecola*. Our findings confirm previous reports [21],[56] that specimens from these two distinct clades are separated from each other by considerable *COI* gene divergence. However, barcodes mostly display the same limitations as morphological characters and cannot differentiate between species that are difficult to identify by traditional approaches. Four major morphological *Aphis* species groups have been reported in recent European studies: [14],[15] *frangulae*-like, black backed aphid ( = *A. craccivora* group), black aphid ( = *A. fabae* group) and *nasturtii*-like aphids. Most of the specimens from the species belonging to one of these groups were recovered in one of the problematic clades highlighted in our study. The genus *Brachycaudus* has been the subject of recent molecular phylogenetic studies [57],[58], based on several genes, including the *COI* barcode fragment. Our results confirm the taxonomic issues identified in these papers. Three clades of poorly discriminated *Brachycaudus* species are found, each displaying some haplotype diversity. However, the observed structure does not match morphological species delineation. In the supplementary material (Text S1), we present a short historical review highlighting the difficulty, within each of these species groups, encountered in the delimitation of taxa, specification of their taxonomic rank and the description of their biological features. The *Dysaphis* (*Dysaphis*) subgenus is traditionally divided into several clearly defined species groups, together with a number of isolated species of uncertain taxonomic position [55]. Only one of these groups, the *D. crataegi* group, is represented by several species in our dataset. Unsurprisingly, all these species were grouped together to form a clade with species displaying an overlapping barcode. This group has been studied in detail in Western Europe, first by Börner [59] and then by Stroyan [55],[60],[61]. It remains a matter of debate whether these taxa should be treated as species or subspecies of *D. crataegi*: this classification is somewhat arbitrary, as it is not based on valid biological criteria [51]. The last genus containing poorly discriminated species is *Macrosiphum*. Four of the seven species present in our sample form two pairs of species, *M. cerinthiacum/stellariae* and *M. cholodkovskyi/euphorbiae*. With the exception of the little studied *M. cerinthiacum*, these species have been recognized as belonging to the morphologically similar *M. euphorbiae* species group [62],[63].

There are several possible explanations for the overlapping barcodes in the 14 clades. First, some of these clades may represent recently diverged taxa. These may be relatively young species in which the *COI* sequence has not yet accumulated mutations. These aphid species groups may have undergone recent adaptive radiation [64]. Two evolutionary scenarios have often been put forward. In the first, an ancestral polyphagous species is thought to have colonized herbaceous plants during their diversification, leading to rapid and extensive speciation through a gradual restriction of host range [65],[66] (but see [67] for an alternative scenario in *Brachycaudus*). This rapid diversification is probably still underway [68], potentially accounting for the homogeneity of these species groups. A non-exclusive second scenario would involve recent speciation through host shifts, with populations colonizing a new plant species and diverging from their population of origin [69]. Such cases of recent speciation accompanied by very small number of *COI* mutations and/or incomplete lineage sorting represent the ultimate limit for barcoding, as they result in non-monophyletic clades. In such cases, it has generally been suggested that more extensive sequence data would improve resolution [46]. Other genes have been tested for aphid barcoding or phylogeny [70],[71]. The use of more variable DNA fragments from the endosymbiotic bacterium *Buchnera aphidicola* currently seems to be a promising way to resolve the problematic cases encountered with *COI* barcoding [72]. However, within *Brachycaudus*, the use of highly variable *Buchnera* DNA fragments has been shown to result in the same conclusions for species delimitation as *COI* barcoding [58]. Even more variable markers, such as those used for population studies, including microsatellites, might be useful for studying relationships between taxa within these species groups [73]. However, they are too variable and too specific for use as a routine identification tool.

Alternatively, the lack of correspondence between sequence variants and existing Linnean binomials may reflect failings of the procedures used for species delimitation in traditional taxonomy or an inconsistent application of the species concept [45],[74]. Imperfect taxonomy can cause non-monophyly when different morphotypes or ecotypes are inappropriately recognized as species. The species concept in aphids has been the subject of considerable debate [62],[75],[76],[77],[78]. Information about life cycle, host specificity and morphology are essential for the delimitation of aphid species [75]. Host plant association is a major driver of reproductive isolation and speciation in aphids [9],[62],[79],[80],[81],[82]. The ecological species concept has thus been intensively used in some species-rich genera, such as *Aphis* and *Dysaphis*. Due to the considerable overlap in morphological characters, all attempts to correlate morphology and host-plant association in the black aphid species group [15] have been unsuccessful. Multivariate morphometric methods have facilitated morphological separation in some cases (e.g. the *Brachycaudus prunicola* species group [83] or the *D. crataegi* species group [60]). However, Shaposhnikov [84] reported that within a single clone of *Dysaphis foeniculus* (Theobald), the allometry of some parts of the aphid body may change in response to different host-plant associations. This led him to conclude that new species have probably been described erroneously. Intensive host-plant transfers have also been conducted in the *Aphis* [85],[86],[87],[88],[89], *Dysaphis* [90],[91],[92], *Macrosiphum* [63],[93],[94] and *Brachycaudus* [95],[96] species groups. Conflicting results have been obtained between different investigations, suggesting that host plant associations may be inconsistent over both time and space. These inconsistencies are probably intrinsic features of the structure of these species complexes, rather than reflecting experimental shortcomings [15].

DNA barcode database users must accept that species definitions are established on the basis of traditional taxonomy, which may be imperfect. It must, therefore, be borne in mind that many formal species are not monophyletic. In addition, due to morphological homogeneity, incorrect species identification may occur more frequently in some species, contributing to the high frequency of polyphyletic species. Misidentifications with the use of GenBank as a barcode database have been reported [97], but the rate of misidentification in the construction of barcode databases has never been evaluated. The use of barcode databases built in collaboration with a taxonomist decreases the risk of misidentification, although mistakes may still occur, particularly for challenging taxonomical groups. All these factors increase the error rates for barcode-based identification and it is thus the traditional way of delimiting and describing species that requires re-evaluation. In this context, trying to identify the perfect gene for barcoding may be pointless. Furthermore, even if aphid taxonomists are, by necessity, also “amateur” botanists, they are not specialists in plant systematics. In situations in which the identification of the aphid is dependent on correct host-plant identification, the frequency of misidentification may be increased further. Even the most recognized aphid taxonomists acknowledge that there has been confusion between species in the past (e.g. [98]). Taxonomists can make identification mistakes that can be traced back with voucher specimens. In some cases, the lack of morphological characters for diagnosis make aphid vouchers useless for future identification. We therefore suggest the establishment of a host-plant herbarium linked to the aphid voucher specimens, to allow the checking of aphid species identification, when issues are highlighted.

Finally, species may share haplotypes due to mitochondrial introgression. These species may lie in the indeterminate zone between differentiated populations and distinct species [99] or formed species that are losing their genetic identity due to secondary contact and hybridization. Most of the aphid species sharing *COI* barcodes hybridize at least occasionally and can produce fertile hybrid offspring. This has been demonstrated experimentally for black aphids [86],[88],[100],[101], *frangulae*-like aphids [102], the *Brachycaudus prunicola* species group [95], the *Dysaphis devecta* species group [103] and the *Macrosiphum euphorbiae* group [63],[104]. These hybrids, which were obtained experimentally, are frequently considered not to occur in natural conditions due to prezygotic (mating on different host plants, phenology shifts etc.) or postzygotic (hybrid sterility, hybrid weakness or F2 breakdown) barriers [62]. However, the co-existence of potential “parental” taxa on shared host plants may help to remove some of these constraints in natural conditions [15]. Natural hybridization may break down isolation and delay the divergence of species within aphid species groups [60].

## Conclusion

Our study contributes to the assembly of a DNA barcode library for the world aphid fauna. The addition of our dataset to those from North America [1] and Korea [27] results in the coverage of only 15% of the described species with published barcodes, this percentage being only slightly increased by the inclusion of recent taxonomic studies (i.e. [25],[105]). More efforts are therefore required for the barcoding of this group of economically important families and model systems for evolutionary biologists.

The geographic scale of the available samples and the relatively well known taxonomy of this group of insects make aphids ideal for the testing of several issues relating to DNA barcoding, such as the impact of geography or taxon coverage on the accuracy of species assignment.

The data presented here confirm that *COI* barcodes are a potentially useful tool for aphid identification. This approach simplifies identification for 80% of the species, including some species that are difficult to identify on the basis of morphological characters only. However, our work also highlights identification difficulties in *Aphis, Brachycaudus*, *Dysaphis* and *Macrosiphum,* genera including a large number of pest species. This may be the stumbling block for the actual use of the aphid barcoding tool, particularly in agricultural management programs, which are likely to be the principal users of this tool. These problematic groups of species have been studied by taxonomists for a very long time. Barcoding cannot replace a comprehensive taxonomic analysis. Detailed genetic, morphological and ecological investigations are required to define species boundaries, and this is the job of taxonomists. However, systematics studies take much longer than barcoding [106]. Such long-term work is incompatible with the urgency of societal demands for a powerful, user-friendly identification tool. One possible pragmatic solution would be to mimic the procedure used by aphid systematics specialists: assigning specimens to a group of species, returning their names with information about the host plants of the different nominal species included in the group, and then allowing the user of the system to identify the specimen on the basis of the available information.

This procedure, together with the different assignment methods, will require evaluation in future studies before the use of aphid barcoding databases as accurate identification tools for applications in pest management and plant quarantine.

## Supporting Information

Figure S1 **Neighbor-joining tree.** Neighbor-joining tree (K2P model) obtained from the analysis of the 1020 COI sequences from the 274 species of European aphid included in the study. Bootstrap values >50 are indicated at nodes.
(PDF)

Table S1 **Data collection.** Data collected for the 1020 specimens used in the study.
(DOCX)

Table S2 **Identification and taxonomic data.** Identification and taxonomic data for the 1020 specimens used in the study.
(DOCX)

Table S3 **List of species included in the study.** List of species included in the study, with a summary of the results obtained in barcoding analysis.
(DOCX)

Text S1 **Detailed discussion on aphid species groups.** Detailed discussion and references for each aphid species groups encountered in the study and the match to our DNA barcoding data.
(DOCX)
