# Supplementary material for: DNA Barcoding and the Associated PhylAphidB@se Website for the Identification of European Aphids (Insecta: Hemiptera: Aphididae)
Source: PLoS One. 2014 Jun 4;9(6):e97620. doi: 10.1371/journal.pone.0097620 (PMC4045754; doi:10.1371/journal.pone.0097620)
Supplement: Table S1 — Data collection. Data collected for the 1020 specimens used in the study. (DOCX) [file pone.0097620.s002.docx]

| Voucher ID | Country | Province | Region | City | Lat._degres | Long._degres | Date collected | Collector | Host-plant familly | Host-plant species |
| --- | --- | --- | --- | --- | --- | --- | --- | --- | --- | --- |
| ACOE365 | France | Midi-Pyrénées | Lot | Payrac | 44,7957 | 1,469278 | 19/06/1998 | Coeur d'Acier | Apiaceae | Daucus carota L. |
| ACOE366 | France | Midi-Pyrénées | Lot | Payrac | 44,7957 | 1,469278 | 19/06/1998 | Coeur d'Acier | Compositae | Sonchus arvensis L. |
| ACOE372 | France | Midi-Pyrénées | Lot | Payrac | 44,7957 | 1,469278 | 22/06/1998 | Coeur d'Acier | Rosaceae | Prunus persica (L.) Batsch |
| ACOE383 | France | Midi-Pyrénées | Lot | Payrac | 44,7957 | 1,469278 | 28/06/1998 | Coeur d'Acier |  |  |
| ACOE384 | France | Haute-Normandie | Seine-Maritime | Criquetot-sur-Ouville | 49,684369 | 0,849392 | 28/06/1998 | Coeur d'Acier | Juglandaceae | Juglans regia L. |
| ACOE385 | France | Haute-Normandie | Seine-Maritime | Criquetot-sur-Ouville | 49,684369 | 0,849392 | 28/06/1998 | Coeur d'Acier | Fabaceae | Lupinus sp. L. |
| ACOE386 | France | Haute-Normandie | Seine-Maritime | Criquetot-sur-Ouville | 49,684369 | 0,849392 | 28/06/1998 | Coeur d'Acier |  |  |
| ACOE387 | France | Haute-Normandie | Seine-Maritime | Criquetot-sur-Ouville | 49,684369 | 0,849392 | 28/06/1998 | Coeur d'Acier | Tiliaceae | Tilia sp. L. |
| ACOE388 | France | Haute-Normandie | Seine-Maritime | La Fontelaye | 49,689899 | 0,952678 | 29/06/1998 | Coeur d'Acier | Apiaceae | Heracleum sphondylium L. |
| ACOE389 | France | Haute-Normandie | Seine-Maritime | La Fontelaye | 49,689899 | 0,952678 | 29/06/1998 | Coeur d'Acier | Apiaceae | Heracleum sphondylium L. |
| ACOE391 | France | Haute-Normandie | Seine-Maritime | La Fontelaye | 49,689899 | 0,952678 | 29/06/1998 | Coeur d'Acier | Ulmaceae | Ulmus minor Miller |
| ACOE392 | France | Haute-Normandie | Seine-Maritime | La Fontelaye | 49,689899 | 0,952678 | 29/06/1998 | Coeur d'Acier | Fabaceae | Vicia sepium L. |
| ACOE393 | France | Haute-Normandie | Seine-Maritime | La Fontelaye | 49,689899 | 0,952678 | 29/06/1998 | Coeur d'Acier | Rosaceae | Rubus sp. L. |
| ACOE394 | France | Haute-Normandie | Seine-Maritime | La Fontelaye | 49,689899 | 0,952678 | 29/06/1998 | Coeur d'Acier | Asteraceae | Arctium sp. L. |
| ACOE395 | France | Haute-Normandie | Seine-Maritime | La Fontelaye | 49,689899 | 0,952678 | 29/06/1998 | Coeur d'Acier | Oenotheraceae | Epilobium sp. L. |
| ACOE398 | France | Haute-Normandie | Seine-Maritime | Sainte-Austreberthe | 49,597202 | 0,976078 | 30/06/1998 | Coeur d'Acier | Corylaceae | Corylus avellana L. |
| ACOE399 | France | Haute-Normandie | Seine-Maritime | Saint-Wandrille-Rancon | 49,527897 | 0,768178 | 01/07/1998 | Coeur d'Acier | Asteraceae | Senecio jacobaea L. |
| ACOE402 | France | Haute-Normandie | Seine-Maritime | Saint-Wandrille-Rancon | 49,527897 | 0,768178 | 01/07/1998 | Coeur d'Acier | Rosaceae | Crataegus sp. L. |
| ACOE403 | France | Haute-Normandie | Seine-Maritime | Saint-Wandrille-Rancon | 49,527897 | 0,768178 | 01/07/1998 | Coeur d'Acier | Betulaceae | Betula sp. L. |
| ACOE409 | France | Haute-Normandie | Seine-Maritime | Saint-Wandrille-Rancon | 49,527897 | 0,768178 | 01/07/1998 | Coeur d'Acier | Apiaceae | Apiaceae sp. Lindl. |
| ACOE410 | France | Haute-Normandie | Eure | Saint-Samson-de-La-Roque | 49,427097 | 0,429778 | 02/07/1998 | Coeur d'Acier | Rosaceae | Filipendula ulmaria (L.) Maxim. |
| ACOE411 | France | Haute-Normandie | Eure | Saint-Samson-de-La-Roque | 49,427097 | 0,429778 | 02/07/1998 | Coeur d'Acier | Lythraceae | Lythrum sp. L. |
| ACOE412 | France | Haute-Normandie | Eure | Saint-Samson-de-La-Roque | 49,427097 | 0,429778 | 02/07/1998 | Coeur d'Acier | Lythraceae | Lythrum salicaria L. |
| ACOE413 | France | Haute-Normandie | Eure | Saint-Samson-de-La-Roque | 49,427097 | 0,429778 | 02/07/1998 | Coeur d'Acier | Fagaceae | Quercus robur L. |
| ACOE416 | France | Haute-Normandie | Seine-Maritime | Le Trait | 49,481998 | 0,808678 | 06/07/1998 | Coeur d'Acier | Oenotheraceae | Epilobium hirsutum L. |
| ACOE418 | France | Haute-Normandie | Seine-Maritime | Le Trait | 49,481998 | 0,808678 | 06/07/1998 | Coeur d'Acier | Rosaceae | Filipendula ulmaria (L.) Maxim. |
| ACOE424 | France | Languedoc-Roussillon | Pyrénées-Orientales | Err | 42,439499 | 2,034478 | 16/07/1998 | Coeur d'Acier | Asteraceae | Senecio pyrenaicus L. |
| ACOE425 | France | Languedoc-Roussillon | Pyrénées-Orientales | Err | 42,439499 | 2,034478 | 16/07/1998 | Coeur d'Acier | Asteraceae | Tanacetum vulgare L. |
| ACOE428 | France | Languedoc-Roussillon | Pyrénées-Orientales | Err | 42,439499 | 2,034478 | 16/07/1998 | Coeur d'Acier | Salicaceae | Populus sp. L. |
| ACOE430 | France | Provence-Alpes-Côte- d'Azur | Alpes-Maritimes | Saint-Etienne-de-Tinee | 44,227778 | 6,948333 | 22/07/1998 | Coeur d'Acier | Asteraceae | Artemisia absinthium L. |
| ACOE432 | Italy | Piemonte (it) |  | Borgo San Dalmazzo | 44,201944 | 7,149722 | 23/07/1998 | Coeur d'Acier | Scrophulariaceae | Verbascum thapsus L. |
| ACOE433 | Italy | Piemonte (it) |  | Borgo San Dalmazzo | 44,201944 | 7,149722 | 23/07/1998 | Coeur d'Acier | Salicaceae | Salix caprea L. |
| ACOE434 | Italy | Piemonte (it) |  | Borgo San Dalmazzo | 44,201944 | 7,149722 | 23/07/1998 | Coeur d'Acier | Asteraceae | Chardon sp. |
| ACOE437 | Italy | Piemonte (it) |  | Borgo San Dalmazzo | 44,201944 | 7,149722 | 24/07/1998 | Coeur d'Acier | Asteraceae | Asteraceae sp. |
| ACOE438 | France | Provence-Alpes-Côte- d'Azur | Alpes-Maritimes | Entraunes | 44,189098 | 6,747778 | 24/07/1998 | Coeur d'Acier | Apiaceae | Apiaceae sp. Lindl. |
| ACOE439 | France | Provence-Alpes-Côte- d'Azur | Alpes-Maritimes | Entraunes | 44,189098 | 6,747778 | 24/07/1998 | Coeur d'Acier | Scrophulariaceae | Verbascum chaixii Vill. |
| ACOE442 | France | Languedoc-Roussillon | Gard | Breau-et-Salagosse | 44,054156 | 3,547389 | 31/07/1998 | Coeur d'Acier | Oenotheraceae | Epilobium sp. L. |
| ACOE443 | France | Languedoc-Roussillon | Gard | Breau-et-Salagosse | 44,054156 | 3,547389 | 31/07/1998 | Coeur d'Acier | Oenotheraceae | Epilobium sp. L. |
| ACOE445 | France | Languedoc-Roussillon | Gard | Breau-et-Salagosse | 44,054156 | 3,547389 | 31/07/1998 | Coeur d'Acier | Pinaceae | Larix sp. P. Miller |
| ACOE446 | France | Languedoc-Roussillon | Gard | Breau-et-Salagosse | 44,054156 | 3,547389 | 31/07/1998 | Coeur d'Acier | Pinaceae | Abies alba MILLER |
| ACOE448 | France | Languedoc-Roussillon | Gard | Breau-et-Salagosse | 44,054156 | 3,547389 | 31/07/1998 | Coeur d'Acier | Poaceae | Poaceae sp. Barnhart |
| ACOE449 | France | Languedoc-Roussillon | Gard | Breau-et-Salagosse | 44,054156 | 3,547389 | 31/07/1998 | Coeur d'Acier | Asteraceae | Hypochoeris sp. L. |
| ACOE450 | France | Languedoc-Roussillon | Gard | Valleraugue | 44,092655 | 3,54708 | 31/07/1998 | Coeur d'Acier | Asteraceae | Centaurea sp. L. |
| ACOE453 | France | Languedoc-Roussillon | Gard | Valleraugue | 44,10301 | 3,541267 | 31/07/1998 | Coeur d'Acier | Juncaceae | Luzula sp. A. P. de Candolle |
| ACOE454 | France | Languedoc-Roussillon | Gard | Valleraugue | 44,10301 | 3,541267 | 31/07/1998 | Coeur d'Acier | Asteraceae | Asteraceae sp. |
| ACOE455 | France | Languedoc-Roussillon | Gard | Valleraugue | 44,114704 | 3,55003 | 31/07/1998 | Coeur d'Acier | Labiatae | Teucrium sp. L. |
| ACOE456 | France | Languedoc-Roussillon | Gard | Valleraugue | 44,114704 | 3,55003 | 31/07/1998 | Coeur d'Acier |  |  |
| ACOE458 | France | Languedoc-Roussillon | Gard | Valleraugue | 44,114704 | 3,55003 | 31/07/1998 | Coeur d'Acier | Oenotheraceae | Epilobium angustifolium L. |
| ACOE459 | France | Languedoc-Roussillon | Gard | Le Vigan | 44,010454 | 3,605851 | 31/07/1998 | Coeur d'Acier | Anacardiaceae | Pistacia sp. L. |
| ACOE460 | France | Languedoc-Roussillon | Gard | Le Vigan | 44,010454 | 3,605851 | 01/07/1998 | Coeur d'Acier | Apiaceae | Foeniculum vulgare Miller |
| ACOE461 | France | Languedoc-Roussillon | Gard | Mandagout | 44,01779 | 3,619861 | 01/07/1998 | Coeur d'Acier | Scrophulariaceae | Verbascum sp. L. |
| ACOE462 | France | Languedoc-Roussillon | Gard | Mandagout | 44,01779 | 3,619861 | 01/07/1998 | Coeur d'Acier | Apiaceae | Pastinaca sativa (Req. ex Godron) Celak. |
| ACOE463 | France | Languedoc-Roussillon | Gard | Mandagout | 44,01779 | 3,619861 | 01/07/1998 | Coeur d'Acier | Pinaceae | Pinus sylvestris L. |
| ACOE464 | France | Provence-Alpes-Côte- d'Azur | Bouches-du-Rhône | Saint-Martin-de-Crau | 43,644264 | 4,699836 | 15/10/1998 | Coeur d'Acier | Asteraceae | Picris echioides L. |
| ACOE465 | France | Provence-Alpes-Côte- d'Azur | Bouches-du-Rhône | Saint-Martin-de-Crau | 43,644264 | 4,699836 | 15/10/1998 | Coeur d'Acier | Asteraceae | Sonchus sp. L. |
| ACOE466 | France | Provence-Alpes-Côte- d'Azur | Bouches-du-Rhône | Saint-Martin-de-Crau | 43,644264 | 4,699836 | 15/10/1998 | Coeur d'Acier | Asteraceae | Artemisia sp. L. |
| ACOE467 | France | Provence-Alpes-Côte- d'Azur | Bouches-du-Rhône | Saint-Martin-de-Crau | 43,644264 | 4,699836 | 15/10/1998 | Coeur d'Acier | Asteraceae | Centaurea sp. L. |
| ACOE470 | France | Provence-Alpes-Côte- d'Azur | Bouches-du-Rhône | Saint-Martin-de-Crau | 43,644264 | 4,699836 | 15/10/1998 | Coeur d'Acier | Brassicaceae | Brassicaceae sp. Burnett |
| ACOE475 | France | Provence-Alpes-Côte- d'Azur | Bouches-du-Rhône | Saint-Martin-de-Crau | 43,644264 | 4,699836 | 15/10/1998 | Meusnier | Apiaceae | Foeniculum vulgare Miller |
| ACOE477 | France | Provence-Alpes-Côte- d'Azur | Bouches-du-Rhône | Arles | 43,639262 | 4,601163 | 15/10/1998 | Coeur d'Acier | Plantaginaceae | Plantago lanceolata L. |
| ACOE482 | France | Provence-Alpes-Côte- d'Azur | Bouches-du-Rhône | Arles | 43,639262 | 4,601163 | 15/10/1998 | Coeur d'Acier | Malvaceae | Althaea officinalis L. |
| ACOE483 | France | Languedoc-Roussillon | Hérault | Montpellier | 43,61717 | 3,858032 | 04/04/1997 | Coeur d'Acier | Araliaceae | Hedera helix L. |
| ACOE495 | France | Haute-Normandie | Seine-Maritime | Criquetot-sur-Ouville | 49,684369 | 0,849392 | 05/05/1997 | Coeur d'Acier | Caprifoliaceae | Viburnum sp. L. |
| ACOE504 | France | Haute-Normandie | Seine-Maritime | Maulevrier-Sainte-Gertrude | 49,552169 | 0,74342 | 15/05/1997 | Coeur d'Acier | Caprifoliaceae | Viburnum lantana L. |
| ACOE506 | France | Haute-Normandie | Seine-Maritime | Duclair | 49,484374 | 0,923799 | 16/05/1997 | Coeur d'Acier | Polygonaceae | Rumex sp. L. |
| ACOE507 | France | Haute-Normandie | Seine-Maritime | Doudeville | 49,719601 | 0,783478 | 16/05/1997 | Coeur d'Acier | Caprifoliaceae | Sambucus nigra L. |
| ACOE509 | France | Haute-Normandie | Seine-Maritime | Criquetot-sur-Ouville | 49,684369 | 0,849392 | 17/05/1997 | Coeur d'Acier | Araliaceae | Hedera helix L. |
| ACOE510 | France | Languedoc-Roussillon | Gard | Saint-Andre-de-Majencoules | 44,0055 | 3,653119 | 23/05/1997 | Coeur d'Acier | Scrophulariaceae | Digitalis purpurea L. |
| ACOE511 | France | Languedoc-Roussillon | Gard | Saint-Andre-de-Majencoules | 44,0055 | 3,653119 | 23/05/1997 | Coeur d'Acier | Scrophulariaceae | Digitalis purpurea L. |
| ACOE512 | France | Languedoc-Roussillon | Gard | Mandagout | 44,01779 | 3,619861 | 29/05/1997 | Coeur d'Acier | Papaveraceae | Papaver rhoeas L. |
| ACOE513 | France | Languedoc-Roussillon | Gard | Mandagout | 44,01779 | 3,619861 | 29/05/1997 | Coeur d'Acier | Apiaceae | Tordylium maximum L. |
| ACOE514 | France | Languedoc-Roussillon | Gard | Mandagout | 44,01779 | 3,619861 | 29/05/1997 | Coeur d'Acier | Asteraceae | Arctium sp. L. |
| ACOE515 | France | Languedoc-Roussillon | Gard | Mandagout | 44,01779 | 3,619861 | 29/05/1997 | Coeur d'Acier | Polygonaceae | Rumex sp. L. |
| ACOE516 | France | Languedoc-Roussillon | Gard | Mandagout | 44,01779 | 3,619861 | 29/05/1997 | Coeur d'Acier | Ranunculaceae | Ranunculus acris L. |
| ACOE517 | France | Languedoc-Roussillon | Gard | Mandagout | 44,01779 | 3,619861 | 29/05/1997 | Coeur d'Acier | Apiaceae | Anthriscus sylvestris (L.) Hoffm. |
| ACOE518 | France | Languedoc-Roussillon | Gard | Le Vigan | 43,991277 | 3,603702 | 29/05/1997 | Coeur d'Acier | Rubiaceae | Galium mollugo L. |
| ACOE519 | France | Languedoc-Roussillon | Gard | Le Vigan | 43,991277 | 3,603702 | 29/05/1997 | Coeur d'Acier | Rubiaceae | Galium aparine L. |
| ACOE520 | France | Languedoc-Roussillon | Gard | Le Vigan | 43,991277 | 3,603702 | 29/05/1997 | Coeur d'Acier | Apiaceae | Foeniculum vulgare Miller |
| ACOE521 | France | Languedoc-Roussillon | Gard | Le Vigan | 43,991277 | 3,603702 | 29/05/1997 | Coeur d'Acier | Chenopodiaceae | Chenopodium album L. |
| ACOE522 | France | Languedoc-Roussillon | Gard | Le Vigan | 44,008524 | 3,609313 | 29/05/1997 | Coeur d'Acier | Polygonaceae | Rumex pulcher L. |
| ACOE523 | France | Languedoc-Roussillon | Gard | Le Vigan | 43,991277 | 3,603702 | 29/05/1997 | Coeur d'Acier | Apiaceae | Bupleurum praealtum L. |
| ACOE525 | France | Languedoc-Roussillon | Hérault | Lattes | 43,552562 | 3,903463 | 09/06/1997 | Coeur d'Acier | Apiaceae | Torilis arvensis (Hudson) Link |
| ACOE527 | France | Languedoc-Roussillon | Hérault | Lattes | 43,552562 | 3,903463 | 09/06/1997 | Coeur d'Acier | Iridaceae | Iris sp. L. |
| ACOE533 | France | Languedoc-Roussillon | Pyrénées-Orientales | Nohedes | 42,6231 | 2,288278 | 25/06/1997 | Coeur d'Acier | Melanthiaceae | Veratrum album L. |
| ACOE534 | France | Languedoc-Roussillon | Pyrénées-Orientales | Nohedes | 42,6231 | 2,288278 | 25/06/1997 | Coeur d'Acier | Asteraceae | Cirsium eriophorum (L.) Scop. |
| ACOE535 | France | Languedoc-Roussillon | Pyrénées-Orientales | Nohedes | 42,6231 | 2,288278 | 25/06/1997 | Coeur d'Acier | Asteraceae | Cirsium eriophorum (L.) Scop. |
| ACOE539 | France | Languedoc-Roussillon | Pyrénées-Orientales | Nohedes | 42,6231 | 2,288278 | 27/06/1997 | Coeur d'Acier | Melanthiaceae | Veratrum album L. |
| ACOE540 | France | Haute-Normandie | Seine-Maritime | Criquetot-sur-Ouville | 49,684369 | 0,849392 | 30/06/1997 | Coeur d'Acier | Polygonaceae | Rumex sp. L. |
| ACOE542 | France | Haute-Normandie | Seine-Maritime | Criquetot-sur-Ouville | 49,684369 | 0,849392 | 02/07/1997 | Coeur d'Acier | Asteraceae | Cirsium arvense (L.) Scop. |
| ACOE547 | France | Haute-Normandie | Seine-Maritime | Criquetot-sur-Ouville | 49,684369 | 0,849392 | 02/07/1997 | Coeur d'Acier | Asteraceae | Cirsium arvense (L.) Scop. |
| ACOE548 | France | Haute-Normandie | Seine-Maritime | Criquetot-sur-Ouville | 49,684369 | 0,849392 | 02/07/1997 | Coeur d'Acier | Asteraceae | Cirsium arvense (L.) Scop. |
| ACOE549 | France | Haute-Normandie | Seine-Maritime | Criquetot-sur-Ouville | 49,684369 | 0,849392 | 02/07/1997 | Coeur d'Acier | Rubiaceae | Galium aparine L. |
| ACOE552 | France | Haute-Normandie | Seine-Maritime | Maulevrier-Sainte-Gertrude | 49,552169 | 0,74342 | 04/07/1997 | Coeur d'Acier | Celastraceae | Evonymus europaeus Hort. |
| ACOE555 | France | Haute-Normandie | Seine-Maritime | Maulevrier-Sainte-Gertrude | 49,552169 | 0,74342 | 05/07/1997 | Coeur d'Acier | Caprifoliaceae | Viburnum opulus L. |
| ACOE556 | France | Haute-Normandie | Seine-Maritime | Maulevrier-Sainte-Gertrude | 49,552169 | 0,74342 | 05/07/1997 | Coeur d'Acier | Celastraceae | Evonymus europaeus Hort. |
| ACOE557 | France | Haute-Normandie | Seine-Maritime | Maulevrier-Sainte-Gertrude | 49,552169 | 0,74342 | 05/07/1997 | Coeur d'Acier | Celastraceae | Evonymus europaeus Hort. |
| ACOE558 | France | Haute-Normandie | Seine-Maritime | Henouville | 49,480202 | 0,956278 | 07/07/1997 | Coeur d'Acier | Papaveraceae | Papaver rhoeas L. |
| ACOE559 | France | Haute-Normandie | Seine-Maritime | Sainte-Austreberthe | 49,583271 | 0,98367 | 07/07/1997 | Coeur d'Acier | Polygonaceae | Rheum rhaponticum L. |
| ACOE560 | France | Haute-Normandie | Seine-Maritime | Sainte-Austreberthe | 49,583271 | 0,98367 | 07/07/1997 | Coeur d'Acier | Umbelliferae | Levisticum officinale KOCH |
| ACOE565 | France | Midi-Pyrénées | Ariège | Mijanes | 42,736667 | 2,005278 | 15/07/1998 | Coeur d'Acier | Melanthiaceae | Veratrum sp. L. |
| ACOE566 | Italy | Piemonte (it) |  | Borgo San Dalmazzo | 44,201944 | 7,149722 | 23/07/1998 | Coeur d'Acier | Scrophulariaceae | Digitalis lutea L. |
| ACOE567 | Italy | Piemonte (it) |  | Borgo San Dalmazzo | 44,201944 | 7,149722 | 23/07/1998 | Coeur d'Acier | Compositae | Senecio nemorensis L. |
| ACOE570 | France | Haute-Normandie | Seine-Maritime | Criquetot-sur-Ouville | 49,684369 | 0,849392 | 04/07/1998 | Coeur d'Acier | Aceraceae | Acer sp. L. |
| ACOE571 | France | Île-de-France | Yvelines | Versailles | 48,806099 | 2,136178 | 04/05/1998 | Coeur d'Acier | Labiatae | Mentha sp. L. |
| ACOE572 | France | Languedoc-Roussillon | Hérault | Pérols | 43,55641 | 4,020544 | 13/05/1999 | Coeur d'Acier | Ranunculaceae | Clematis vitalba L. |
| ACOE573 | France | Languedoc-Roussillon | Hérault | Pérols | 43,55641 | 4,020544 | 13/05/1999 | Coeur d'Acier | Asteraceae | Helichrysum stoechas (L.) Moench |
| ACOE574 | France | Languedoc-Roussillon | Hérault | Pérols | 43,55641 | 4,020544 | 13/05/1999 | Coeur d'Acier |  |  |
| ACOE575 | France | Languedoc-Roussillon | Hérault | Pérols | 43,55641 | 4,020544 | 13/05/1999 | Coeur d'Acier | Ephedraceae | Ephedra distachya L. |
| ACOE576 | France | Languedoc-Roussillon | Hérault | Pérols | 43,55641 | 4,020544 | 13/05/1999 | Coeur d'Acier | Poaceae | Arundo donax L. |
| ACOE577 | France | Languedoc-Roussillon | Gard | Vissec | 43,893 | 3,5005 | 15/05/1999 | Coeur d'Acier | Cornaceae | Cornus sanguinea L. |
| ACOE578 | France | Languedoc-Roussillon | Gard | Vissec | 43,893 | 3,5005 | 15/05/1999 | Coeur d'Acier | Rosaceae | Sanguisorba minor Scop. |
| ACOE579 | France | Languedoc-Roussillon | Gard | Vissec | 43,893 | 3,5005 | 15/05/1999 | Coeur d'Acier | Rosaceae | Sanguisorba minor Scop. |
| ACOE580 | France | Languedoc-Roussillon | Gard | Vissec | 43,893 | 3,5005 | 15/05/1999 | Coeur d'Acier | Apiaceae | Apiaceae sp. Lindl. |
| ACOE581 | France | Languedoc-Roussillon | Gard | Vissec | 43,893 | 3,5005 | 15/05/1999 | Coeur d'Acier | Salicaceae | Populus nigra L. |
| ACOE582 | France | Languedoc-Roussillon | Gard | Vissec | 43,893 | 3,5005 | 15/05/1999 | Coeur d'Acier | Salicaceae | Populus nigra L. |
| ACOE583 | France | Languedoc-Roussillon | Gard | Vissec | 43,893 | 3,5005 | 15/05/1999 | Coeur d'Acier | Fabaceae | Medicago sp. L. |
| ACOE585 | France | Languedoc-Roussillon | Gard | Vissec | 43,893 | 3,5005 | 15/05/1999 | Coeur d'Acier | Aceraceae | Acer monspessulanum L. |
| ACOE587 | France | Languedoc-Roussillon | Gard | Vissec | 43,893 | 3,5005 | 15/05/1999 | Coeur d'Acier | Asteraceae | Tragopogon pratensis L. |
| ACOE588 | France | Languedoc-Roussillon | Hérault | Pérols | 43,55641 | 4,020544 | 26/05/1999 | Coeur d'Acier | Asteraceae | Centaurea aspera L. |
| ACOE589 | France | Languedoc-Roussillon | Hérault | Pérols | 43,55641 | 4,020544 | 26/05/1999 | Coeur d'Acier | Asteraceae | Centaurea aspera L. |
| ACOE590 | France | Languedoc-Roussillon | Hérault | Pérols | 43,55641 | 4,020544 | 26/05/1999 | Coeur d'Acier | Asteraceae | Centaurea aspera L. |
| ACOE592 | France | Languedoc-Roussillon | Hérault | Pérols | 43,55641 | 4,020544 | 26/05/1999 | Coeur d'Acier | Tamaricaceae | Tamarix sp. L. |
| ACOE593 | France | Midi-Pyrénées | Lot | Rocamadour | 44,803882 | 1,625382 | 31/05/1999 | Coeur d'Acier | Aceraceae | Acer monspessulanum L. |
| ACOE595 | France | Midi-Pyrénées | Lot | Rocamadour | 44,803882 | 1,625382 | 31/05/1999 | Coeur d'Acier | Rosaceae | Prunus mahaleb L. |
| ACOE596 | France | Midi-Pyrénées | Lot | Rocamadour | 44,803882 | 1,625382 | 31/05/1999 | Coeur d'Acier | Apiaceae | Apiaceae sp. Lindl. |
| ACOE599 | France | Midi-Pyrénées | Lot | Rocamadour | 44,803882 | 1,625382 | 31/05/1999 | Coeur d'Acier | Polygonaceae | Polygonum sp. L. |
| ACOE601 | France | Midi-Pyrénées | Lot | Rocamadour | 44,803882 | 1,625382 | 31/05/1999 | Coeur d'Acier | Asteraceae | Sonchus sp. L. |
| ACOE602 | France | Midi-Pyrénées | Lot | Rocamadour | 44,803882 | 1,625382 | 31/05/1999 | Coeur d'Acier | Asteraceae | Sonchus sp. L. |
| ACOE603 | France | Midi-Pyrénées | Lot | Rocamadour | 44,803882 | 1,625382 | 31/05/1999 | Coeur d'Acier | Rubiaceae | Galium aparine L. |
| ACOE604 | France | Midi-Pyrénées | Lot | Rocamadour | 44,803882 | 1,625382 | 31/05/1999 | Coeur d'Acier | Rosaceae | Spiraea sp. L. |
| ACOE605 | France | Midi-Pyrénées | Lot | Rocamadour | 44,803882 | 1,625382 | 31/05/1999 | Coeur d'Acier | Papaveraceae | Papaver rhoeas L. |
| ACOE607 | France | Midi-Pyrénées | Lot | Rocamadour | 44,803882 | 1,625382 | 31/05/1999 | Coeur d'Acier | Urticaceae | Urtica sp. L. |
| ACOE609 | France | Midi-Pyrénées | Lot | Rocamadour | 44,803882 | 1,625382 | 31/05/1999 | Coeur d'Acier | Rosaceae | Rubus fruticosus L. |
| ACOE610 | France | Midi-Pyrénées | Lot | Rocamadour | 44,803882 | 1,625382 | 31/05/1999 | Coeur d'Acier | Orchidaceae | Himantoglossum hircinum (L.) Sprengel |
| ACOE611 | France | Midi-Pyrénées | Lot | Rocamadour | 44,803882 | 1,625382 | 31/05/1999 | Coeur d'Acier | Rosaceae | Rosa canina L. |
| ACOE612 | France | Midi-Pyrénées | Lot | Rocamadour | 44,803882 | 1,625382 | 31/05/1999 | Coeur d'Acier | Fabaceae | Vicia sp. L. |
| ACOE613 | France | Aquitaine | Dordogne | Peyrillac-et-Millac | 44,889297 | 1,406278 | 02/06/1999 | Coeur d'Acier | Asteraceae | Artemisia sp. L. |
| ACOE614 | France | Midi-Pyrénées | Lot | Rocamadour | 44,803882 | 1,625382 | 31/05/1999 | Coeur d'Acier | Asteraceae | Cichorium intybus L. |
| ACOE615 | France | Aquitaine | Dordogne | Coly | 44,8875 | 1,286029 | 01/06/1999 | Coeur d'Acier | Rosaceae | Sorbus torminalis (L.) Crantz |
| ACOE616 | France | Aquitaine | Dordogne | Coly | 44,8875 | 1,286029 | 01/06/1999 | Coeur d'Acier | Celastraceae | Evonymus europaeus L. |
| ACOE617 | France | Aquitaine | Dordogne | Coly | 44,8875 | 1,286029 | 01/06/1999 | Coeur d'Acier | Leguminosae | Melilotus sp. P. Miller |
| ACOE618 | France | Aquitaine | Dordogne | Coly | 44,8875 | 1,286029 | 01/06/1999 | Coeur d'Acier | Asteraceae | Centaurea sp. L. |
| ACOE619 | France | Aquitaine | Dordogne | Coly | 44,8875 | 1,286029 | 01/06/1999 | Coeur d'Acier |  |  |
| ACOE620 | France | Aquitaine | Dordogne | Coly | 44,8875 | 1,286029 | 01/06/1999 | Coeur d'Acier | Campanulaceae | Campanula trachelium L. |
| ACOE621 | France | Aquitaine | Dordogne | Coly | 44,8875 | 1,286029 | 01/06/1999 | Coeur d'Acier | Labiatae | Teucrium scorodonia L. |
| ACOE622 | France | Aquitaine | Dordogne | Coly | 44,8875 | 1,286029 | 01/06/1999 | Coeur d'Acier | Asteraceae | Taraxacum officinale Weber |
| ACOE623 | France | Aquitaine | Dordogne | Coly | 44,8875 | 1,286029 | 01/06/1999 | Coeur d'Acier | Asteraceae | Asteraceae sp. |
| ACOE625 | France | Aquitaine | Dordogne | Coly | 44,8875 | 1,286029 | 01/06/1999 | Coeur d'Acier | Urticaceae | Urtica sp. L. |
| ACOE627 | France | Aquitaine | Dordogne | Coly | 44,8875 | 1,286029 | 01/06/1999 | Coeur d'Acier | Dipsacaceae | Knautia arvensis (L.) Coulter |
| ACOE629 | France | Aquitaine | Dordogne | Coly | 44,8875 | 1,286029 | 01/06/1999 | Coeur d'Acier | Asteraceae | Tragopogon pratensis L. |
| ACOE630 | France | Aquitaine | Dordogne | Coly | 44,8875 | 1,286029 | 01/06/1999 | Coeur d'Acier | Asteraceae | Senecio jacobaea L. |
| ACOE631 | France | Aquitaine | Dordogne | Coly | 44,8875 | 1,286029 | 01/06/1999 | Coeur d'Acier | Asteraceae | Asteraceae sp. |
| ACOE632 | France | Aquitaine | Dordogne | Coly | 44,8875 | 1,286029 | 01/06/1999 | Coeur d'Acier | Salicaceae | Salix sp. L. |
| ACOE633 | France | Aquitaine | Dordogne | Coly | 44,8875 | 1,286029 | 01/06/1999 | Coeur d'Acier | Pinaceae | Picea abies (L.) Karsten |
| ACOE635 | France | Aquitaine | Dordogne | Coly | 45,08657 | 1,268822 | 01/06/1999 | Coeur d'Acier | Asteraceae | Taraxacum officinale Weber |
| ACOE636 | France | Aquitaine | Dordogne | Coly | 45,08657 | 1,268822 | 01/06/1999 | Coeur d'Acier | Poaceae | Phragmites australis (Cav.) Steudel |
| ACOE637 | France | Aquitaine | Dordogne | Coly | 45,08657 | 1,268822 | 01/06/1999 | Coeur d'Acier | Rosaceae | Rubus sp. L. |
| ACOE638 | France | Aquitaine | Dordogne | Coly | 45,08657 | 1,268822 | 01/06/1999 | Coeur d'Acier | Asteraceae | Carduus sp. L. |
| ACOE639 | France | Aquitaine | Dordogne | Coly | 45,08657 | 1,268822 | 01/06/1999 | Coeur d'Acier |  |  |
| ACOE640 | France | Aquitaine | Dordogne | Coly | 45,08657 | 1,268822 | 01/06/1999 | Coeur d'Acier | Cupressaceae | Juniperus sp. L. |
| ACOE641 | France | Aquitaine | Dordogne | Coly | 45,08657 | 1,268822 | 01/06/1999 | Coeur d'Acier | Labiatae | Salvia pratensis L. |
| ACOE642 | France | Aquitaine | Dordogne | Coly | 45,08657 | 1,268822 | 01/06/1999 | Coeur d'Acier | Apiaceae | Eryngium campestre L. |
| ACOE644 | France | Aquitaine | Dordogne | Peyrillac-et-Millac | 44,889297 | 1,406278 | 02/06/1999 | Coeur d'Acier | Caryophyllaceae | Silene sp. L. |
| ACOE645 | France | Aquitaine | Dordogne | Peyrillac-et-Millac | 44,889297 | 1,406278 | 02/06/1999 | Coeur d'Acier | Apiaceae | Pastinaca sativa L. |
| ACOE646 | France | Aquitaine | Dordogne | Peyrillac-et-Millac | 44,889297 | 1,406278 | 02/06/1999 | Coeur d'Acier | Rosaceae | Prunus sp. L. |
| ACOE648 | France | Aquitaine | Dordogne | Peyrillac-et-Millac | 44,889297 | 1,406278 | 02/06/1999 | Coeur d'Acier | Asteraceae | Artemisia sp. L. |
| ACOE649 | France | Aquitaine | Dordogne | Saint-Julien-de-Lampon | 44,8605 | 1,362178 | 02/06/1999 | Coeur d'Acier | Caryophyllaceae | Silene sp. L. |
| ACOE651 | France | Aquitaine | Dordogne | Saint-Julien-de-Lampon | 44,8605 | 1,362178 | 02/06/1999 | Coeur d'Acier | Asteraceae | Hypochaeris radicata L. |
| ACOE654 | France | Aquitaine | Dordogne | Saint-Julien-de-Lampon | 44,8605 | 1,362178 | 02/06/1999 | Coeur d'Acier | Salicaceae | Populus nigra L. |
| ACOE655 | France | Aquitaine | Dordogne | Saint-Julien-de-Lampon | 44,8605 | 1,362178 | 02/06/1999 | Coeur d'Acier | Salicaceae | Populus nigra L. |
| ACOE656 | France | Aquitaine | Dordogne | Saint-Julien-de-Lampon | 44,8605 | 1,362178 | 02/06/1999 | Coeur d'Acier | Salicaceae | Populus nigra L. |
| ACOE658 | France | Aquitaine | Dordogne | Saint-Julien-de-Lampon | 44,8605 | 1,362178 | 02/06/1999 | Coeur d'Acier | Fabaceae | Trifolium sp. L. |
| ACOE659 | France | Aquitaine | Dordogne | Sainte-Mondane | 44,847 | 1,342729 | 02/06/1999 | Coeur d'Acier | Asteraceae | Arctium sp. L. |
| ACOE661 | France | Midi-Pyrénées | Lot | Payrac | 44,7957 | 1,469278 | 02/06/1999 | Coeur d'Acier | Conifere | Conifère sp. |
| ACOE662 | France | Aquitaine | Dordogne | Saint-Vincent-de-Cosse | 44,8407 | 1,121509 | 02/06/1999 | Coeur d'Acier | Polygonaceae | Polygonaceae sp. Juss. |
| ACOE665 | France | Aquitaine | Dordogne | Saint-Vincent-de-Cosse | 44,8407 | 1,121509 | 03/06/1999 | Coeur d'Acier | Malvaceae | Malva sp. L. |
| ACOE666 | France | Aquitaine | Dordogne | Saint-Vincent-de-Cosse | 44,8407 | 1,121509 | 03/06/1999 | Coeur d'Acier | Caprifoliaceae | Sambucus nigra L. |
| ACOE668 | France | Aquitaine | Dordogne | Saint-Vincent-de-Cosse | 44,8407 | 1,121509 | 03/06/1999 | Coeur d'Acier | Asteraceae | Crepis sp. L. |
| ACOE669 | France | Aquitaine | Dordogne | Saint-Vincent-de-Cosse | 44,8407 | 1,121509 | 03/06/1999 | Coeur d'Acier | Dipsacaceae | Dipsacus sp. L. |
| ACOE671 | France | Aquitaine | Dordogne | Saint-Vincent-de-Cosse | 44,8407 | 1,121509 | 03/06/1999 | Coeur d'Acier |  |  |
| ACOE673 | France | Aquitaine | Dordogne | Saint-Vincent-de-Cosse | 44,8407 | 1,121509 | 03/06/1999 | Coeur d'Acier | Euphorbiaceae | Euphorbia sp. L. |
| ACOE674 | France | Aquitaine | Dordogne | Saint-Vincent-de-Cosse | 44,8407 | 1,121509 | 03/06/1999 | Coeur d'Acier | Salicaceae | Populus nigra L. |
| ACOE675 | France | Aquitaine | Dordogne | Saint-Vincent-de-Cosse | 44,8407 | 1,121509 | 03/06/1999 | Coeur d'Acier | Juglandaceae | Juglans regia L. |
| ACOE677 | France | Aquitaine | Dordogne | Saint-Vincent-de-Cosse | 44,8407 | 1,121509 | 03/06/1999 | Coeur d'Acier | Rosaceae | Crataegus oxyacantha L., nom. rej. |
| ACOE678 | France | Aquitaine | Dordogne | Saint-Vincent-de-Cosse | 44,8407 | 1,121509 | 03/06/1999 | Coeur d'Acier | Iridaceae | Iris sp. L. |
| ACOE679 | France | Aquitaine | Dordogne | Saint-Vincent-de-Cosse | 44,8407 | 1,121509 | 03/06/1999 | Coeur d'Acier | Rosaceae | Prunus sp. L. |
| ACOE682 | France | Aquitaine | Dordogne | Saint-Vincent-de-Cosse | 44,8407 | 1,121509 | 03/06/1999 | Coeur d'Acier | Asteraceae | Senecio vulgaris L. |
| ACOE683 | France | Aquitaine | Dordogne | Saint-Vincent-de-Cosse | 44,8407 | 1,121509 | 03/06/1999 | Coeur d'Acier | Valerianaceae | Centranthus ruber (L.) DC. |
| ACOE684 | France | Aquitaine | Dordogne | Beynac-et-Cazenac | 44,856 | 1,124929 | 03/06/1999 | Coeur d'Acier | Asteraceae | Cirsium sp. P. Miller |
| ACOE685 | France | Aquitaine | Dordogne | Beynac-et-Cazenac | 44,856 | 1,124929 | 03/06/1999 | Coeur d'Acier | Asteraceae | Arctium sp. L. |
| ACOE686 | France | Aquitaine | Dordogne | Beynac-et-Cazenac | 44,856 | 1,124929 | 03/06/1999 | Coeur d'Acier | Fabaceae | Lathyrus sp. L. |
| ACOE687 | France | Aquitaine | Dordogne | Beynac-et-Cazenac | 44,856 | 1,124929 | 03/06/1999 | Coeur d'Acier | Asteraceae | Achillea sp. L. |
| ACOE688 | France | Aquitaine | Dordogne | Beynac-et-Cazenac | 44,856 | 1,124929 | 03/06/1999 | Coeur d'Acier | Asteraceae | Achillea sp. L. |
| ACOE691 | France | Aquitaine | Dordogne | Saint-Vincent-de-Cosse | 44,8407 | 1,121509 | 03/06/1999 | Coeur d'Acier |  |  |
| ACOE692 | France | Aquitaine | Dordogne | Peyrillac-et-Millac | 44,889297 | 1,406278 | 02/06/1999 | Coeur d'Acier | Asteraceae | Artemisia sp. L. |
| ACOE693 | France | Alsace | Haut-Rhin | Le Bonhomme | 48,154974 | 7,081758 | 22/06/1999 | Coeur d'Acier | Aceraceae | Acer pseudoplatanus L. |
| ACOE694 | France | Alsace | Haut-Rhin | Le Bonhomme | 48,154974 | 7,081758 | 22/06/1999 | Coeur d'Acier |  |  |
| ACOE695 | France | Alsace | Haut-Rhin | Le Bonhomme | 48,154974 | 7,081758 | 22/06/1999 | Coeur d'Acier | Dipsacaceae | Dipsacaceae sp. Juss. |
| ACOE696 | France | Alsace | Haut-Rhin | Le Bonhomme | 48,154974 | 7,081758 | 22/06/1999 | Coeur d'Acier | Oenotheraceae | Epilobium sp. L. |
| ACOE697 | France | Alsace | Haut-Rhin | Le Bonhomme | 48,154974 | 7,081758 | 22/06/1999 | Coeur d'Acier |  |  |
| ACOE700 | France | Alsace | Haut-Rhin | Le Bonhomme | 48,154974 | 7,081758 | 22/06/1999 | Coeur d'Acier | Dipsacaceae | Dipsacaceae sp. Juss. |
| ACOE701 | France | Alsace | Haut-Rhin | Le Bonhomme | 48,154974 | 7,081758 | 22/06/1999 | Coeur d'Acier | Asteraceae | Achillea sp. L. |
| ACOE702 | France | Alsace | Haut-Rhin | Le Bonhomme | 48,154974 | 7,081758 | 22/06/1999 | Coeur d'Acier | Apiaceae | Apiaceae sp. Lindl. |
| ACOE704 | France | Alsace | Haut-Rhin | Le Bonhomme | 48,154974 | 7,081758 | 22/06/1999 | Coeur d'Acier | Polygonaceae | Rumex sp. L. |
| ACOE705 | France | Lorraine | Vosges | Xonrupt Longemer | 48,04191 | 7,011391 | 22/06/1999 | Coeur d'Acier | Asteraceae | Achillea macrophylla L. |
| ACOE706 | France | Lorraine | Vosges | Xonrupt Longemer | 48,04191 | 7,011391 | 22/06/1999 | Coeur d'Acier | Asteraceae | Achillea macrophylla L. |
| ACOE707 | France | Lorraine | Vosges | Xonrupt Longemer | 48,04191 | 7,011391 | 22/06/1999 | Coeur d'Acier | Asteraceae | Saussurea alpina (L.) DC. |
| ACOE708 | France | Lorraine | Vosges | Xonrupt Longemer | 48,04191 | 7,011391 | 22/06/1999 | Coeur d'Acier |  |  |
| ACOE711 | France | Lorraine | Vosges | Xonrupt Longemer | 48,04191 | 7,011391 | 22/06/1999 | Coeur d'Acier | Asteraceae | Leontodon pyrenaicus (Mérat) Finch & P.D. Sell |
| ACOE713 | France | Alsace | Haut-Rhin | Kaysersberg | 48,138298 | 7,262578 | 22/06/1999 | Coeur d'Acier | Caprifoliaceae | Sambucus nigra L. |
| ACOE718 | France | Alsace | Haut-Rhin | Kaysersberg | 48,138298 | 7,262578 | 22/06/1999 | Coeur d'Acier |  |  |
| ACOE720 | France | Alsace | Haut-Rhin | Kaysersberg | 48,138298 | 7,262578 | 22/06/1999 | Coeur d'Acier | Rosaceae | Rosa sp. L. |
| ACOE721 | France | Alsace | Haut-Rhin | Kaysersberg | 48,138298 | 7,262578 | 22/06/1999 | Coeur d'Acier | Rosaceae | Spiraea sp. L. |
| ACOE722 | France | Alsace | Haut-Rhin | Kaysersberg | 48,157688 | 7,217606 | 23/06/1999 | Coeur d'Acier | Asteraceae | Sonchus sp. L. |
| ACOE724 | France | Alsace | Haut-Rhin | Kaysersberg | 48,157688 | 7,217606 | 23/06/1999 | Coeur d'Acier | Asteraceae | Lapsana sp. L. |
| ACOE726 | France | Alsace | Haut-Rhin | Kaysersberg | 48,157688 | 7,217606 | 23/06/1999 | Coeur d'Acier | Oenotheraceae | Epilobium sp. L. |
| ACOE729 | France | Alsace | Haut-Rhin | Kaysersberg | 48,157688 | 7,217606 | 23/06/1999 | Coeur d'Acier | Rosaceae | Rubus sp. L. |
| ACOE730 | France | Alsace | Haut-Rhin | Kaysersberg | 48,157688 | 7,217606 | 23/06/1999 | Coeur d'Acier | Fabaceae | Trifolium sp. L. |
| ACOE731 | France | Alsace | Haut-Rhin | Kaysersberg | 48,157688 | 7,217606 | 23/06/1999 | Coeur d'Acier | Rubiaceae | Galium aparine L. |
| ACOE732 | France | Alsace | Haut-Rhin | Kaysersberg | 48,157688 | 7,217606 | 23/06/1999 | Coeur d'Acier | Asteraceae | Achillea millefolium L. |
| ACOE733 | France | Alsace | Haut-Rhin | Kaysersberg | 48,157688 | 7,217606 | 23/06/1999 | Coeur d'Acier | Asteraceae | Sonchus sp. L. |
| ACOE734 | France | Alsace | Haut-Rhin | Kaysersberg | 48,157688 | 7,217606 | 23/06/1999 | Coeur d'Acier | Apiaceae | Apiaceae sp. Lindl. |
| ACOE735 | France | Alsace | Haut-Rhin | Kaysersberg | 48,157688 | 7,217606 | 23/06/1999 | Coeur d'Acier | Apiaceae | Apiaceae sp. Lindl. |
| ACOE736 | France | Alsace | Haut-Rhin | Kaysersberg | 48,157688 | 7,217606 | 23/06/1999 | Coeur d'Acier | Fabaceae | Trifolium sp. L. |
| ACOE739 | France | Alsace | Haut-Rhin | Kaysersberg | 48,157688 | 7,217606 | 23/06/1999 | Coeur d'Acier | Asteraceae | Asteraceae sp. |
| ACOE741 | France | Alsace | Haut-Rhin | Kaysersberg | 48,157688 | 7,217606 | 23/06/1999 | Coeur d'Acier | Polygonaceae | Rumex sp. L. |
| ACOE742 | France | Alsace | Haut-Rhin | Kaysersberg | 48,157688 | 7,217606 | 23/06/1999 | Coeur d'Acier | Corylaceae | Corylus avellana L. |
| ACOE744 | France | Alsace | Haut-Rhin | Le Bonhomme | 48,1716 | 7,113178 | 23/06/1999 | Coeur d'Acier | Polygonaceae | Rumex sp. L. |
| ACOE747 | France | Alsace | Haut-Rhin | Lapoutroie | 48,170351 | 7,095343 | 23/06/1999 | Coeur d'Acier | Asteraceae | Asteraceae sp. |
| ACOE748 | France | Alsace | Bas-Rhin | Selestat | 48,2607 | 7,447078 | 24/06/1999 | Coeur d'Acier | Poaceae | Phragmites australis (Cav.) Steudel |
| ACOE749 | France | Alsace | Bas-Rhin | Selestat | 48,2607 | 7,447078 | 24/06/1999 | Coeur d'Acier | Orchidaceae | Orchidaceae sp. Juss. |
| ACOE750 | France | Alsace | Bas-Rhin | Selestat | 48,2607 | 7,447078 | 24/06/1999 | Coeur d'Acier | Iridaceae | Iris sp. L. |
| ACOE752 | France | Alsace | Bas-Rhin | Selestat | 48,2607 | 7,447078 | 24/06/1999 | Coeur d'Acier | Dipsacaceae | Dipsacus fullonum L. |
| ACOE755 | France | Alsace | Bas-Rhin | Selestat | 48,2607 | 7,447078 | 24/06/1999 | Coeur d'Acier | Fagaceae | Quercus sp. L. |
| ACOE756 | France | Alsace | Bas-Rhin | Selestat | 48,2607 | 7,447078 | 24/06/1999 | Coeur d'Acier | Betulaceae | Alnus sp. P. Miller |
| ACOE758 | France | Alsace | Haut-Rhin | Le Bonhomme | 48,19387 | 7,113003 | 24/06/1999 | Coeur d'Acier | Pinaceae | Picea abies (L.) Karsten |
| ACOE759 | France | Alsace | Haut-Rhin | Le Bonhomme | 48,19387 | 7,113003 | 24/06/1999 | Coeur d'Acier | Apiaceae | Apiaceae sp. Lindl. |
| ACOE761 | France | Lorraine | Vosges | Xonrupt Longemer | 48,04191 | 7,011391 | 24/06/1999 | Coeur d'Acier | Asteraceae | Solidago glomerata Michx. |
| ACOE762 | France | Franche-Comté | Jura | Audelange | 47,147283 | 5,581167 | 25/06/1999 | Coeur d'Acier | Corylaceae | Corylus avellana L. |
| ACOE765 | France | Languedoc-Roussillon | Gard | Aigues-Mortes | 43,527778 | 4,1775 | 12/06/1999 | Gompel | Salicaceae | Populus nigra L. |
| ACOE819 | France | Languedoc-Roussillon | Hérault | Saint-Bauzille-de-La-Sylve | 43,615803 | 3,547378 | 08/04/2000 | Coeur d'Acier | Pinaceae | Pinus sp. L. |
| ACOE824 | France | Languedoc-Roussillon | Hérault | Saint-Guilhem-le-Desert | 43,7337 | 3,550078 | 08/04/2000 | Coeur d'Acier | Fabaceae | Trifolium sp. L. |
| ACOE827 | France | Languedoc-Roussillon | Hérault | Saint-Clement-de-Riviere | 43,685101 | 3,850678 | 15/03/2000 | Yvon | Labiatae | Thymus vulgaris L. |
| ACOE828 | France | Languedoc-Roussillon | Hérault | Montpellier | 43,616192 | 3,859809 | 13/04/2000 | Coeur d'Acier | Rhamnaceae | Frangula alnus Miller |
| ACOE829 | France | Languedoc-Roussillon | Gard | Anduze | 44,055 | 3,986578 | 14/04/2000 | Coeur d'Acier | Fabaceae | Spartium junceum L. |
| ACOE830 | France | Languedoc-Roussillon | Gard | Anduze | 44,055 | 3,986578 | 14/04/2000 | Coeur d'Acier | Asteraceae | Asteraceae sp. |
| ACOE831 | France | Languedoc-Roussillon | Gard | Anduze | 44,055 | 3,986578 | 14/04/2000 | Coeur d'Acier | Rosaceae | Rubus sp. L. |
| ACOE832 | France | Languedoc-Roussillon | Gard | Anduze | 44,055 | 3,986578 | 14/04/2000 | Coeur d'Acier | Asteraceae | Asteraceae sp. |
| ACOE834 | France | Languedoc-Roussillon | Aude | Lagrasse | 43,091103 | 2,620378 | 19/04/2000 | Coeur d'Acier | Apiaceae | Aegopodium podagraria L. |
| ACOE838 | France | Languedoc-Roussillon | Aude | Lagrasse | 43,091103 | 2,620378 | 19/04/2000 | Coeur d'Acier | Asteraceae | Sonchus sp. L. |
| ACOE855 | France | Aquitaine | Landes | Ondres | 43,561802 | -1,449422 | 07/06/2000 | Coeur d'Acier | Oenotheraceae | Oenothera sp. L. |
| ACOE875 | France | Aquitaine | Landes | Orx | 43,603203 | -1,368422 | 08/06/2000 | Coeur d'Acier | Salicaceae | Salix sp. L. |
| ACOE899 | France | Languedoc-Roussillon | Pyrénées-Orientales | Le Barcares | 42,7869 | 3,036178 | 21/06/2000 | Coeur d'Acier | Apiaceae | Eryngium campestre L. |
| ACOE902 | France | Languedoc-Roussillon | Pyrénées-Orientales | Le Barcares | 42,7869 | 3,036178 | 22/06/2000 | Coeur d'Acier | Fabaceae | Spartium junceum L. |
| ACOE903 | France | Languedoc-Roussillon | Pyrénées-Orientales | Le Barcares | 42,7869 | 3,036178 | 22/06/2000 | Coeur d'Acier | Salicaceae | Populus nigra L. |
| ACOE904 | France | Languedoc-Roussillon | Pyrénées-Orientales | Le Barcares | 42,7869 | 3,036178 | 22/06/2000 | Coeur d'Acier | Apiaceae | Daucus carota L. |
| ACOE905 | France | Languedoc-Roussillon | Pyrénées-Orientales | Le Barcares | 42,7869 | 3,036178 | 22/06/2000 | Coeur d'Acier | Asteraceae | Asteraceae sp. |
| ACOE906 | France | Languedoc-Roussillon | Pyrénées-Orientales | Le Barcares | 42,7869 | 3,036178 | 22/06/2000 | Coeur d'Acier | Fabaceae | Medicago littoralis Rohde ex Loisel. |
| ACOE907 | France | Languedoc-Roussillon | Pyrénées-Orientales | Le Barcares | 42,7869 | 3,036178 | 22/06/2000 | Coeur d'Acier | Crassulaceae | Crassulaceae sp. D. C. |
| ACOE908 | France | Languedoc-Roussillon | Pyrénées-Orientales | Le Barcares | 42,7869 | 3,036178 | 23/06/2000 | Coeur d'Acier | Salicaceae | Populus alba L. |
| ACOE910 | France | Languedoc-Roussillon | Pyrénées-Orientales | Le Barcares | 42,7869 | 3,036178 | 23/06/2000 | Coeur d'Acier | Asteraceae | Sonchus sp. L. |
| ACOE911 | France | Languedoc-Roussillon | Pyrénées-Orientales | Le Barcares | 42,7869 | 3,036178 | 23/06/2000 | Coeur d'Acier | Valerianaceae | Centranthus sp. A.P. de Candolle |
| ACOE912 | France | Languedoc-Roussillon | Pyrénées-Orientales | Rodes | 42,658199 | 2,562778 | 23/06/2000 | Coeur d'Acier | Apiaceae | Apiaceae sp. Lindl. |
| ACOE913 | France | Languedoc-Roussillon | Pyrénées-Orientales | Rodes | 42,658199 | 2,562778 | 23/06/2000 | Coeur d'Acier | Solanaceae | Solanum sp. L. |
| ACOE917 | France | Languedoc-Roussillon | Pyrénées-Orientales | Rodes | 42,658199 | 2,562778 | 25/06/2000 | Coeur d'Acier | Poaceae | Arundo sp. L. |
| ACOE918 | France | Languedoc-Roussillon | Pyrénées-Orientales | Rodes | 42,658199 | 2,562778 | 25/06/2000 | Coeur d'Acier | Caryophyllaceae | Silene sp. L. |
| ACOE922 | France | Languedoc-Roussillon | Pyrénées-Orientales | Rodes | 42,658199 | 2,562778 | 25/06/2000 | Coeur d'Acier | Asteraceae | Carthamus lanatus L. |
| ACOE923 | France | Languedoc-Roussillon | Pyrénées-Orientales | Rodes | 42,658199 | 2,562778 | 25/06/2000 | Coeur d'Acier | Salicaceae | Populus canadensis Moench |
| ACOE924 | France | Languedoc-Roussillon | Pyrénées-Orientales | Rodes | 42,658199 | 2,562778 | 25/06/2000 | Coeur d'Acier | Rosaceae | Prunus avium L. |
| ACOE926 | France | Languedoc-Roussillon | Pyrénées-Orientales | Rodes | 42,658199 | 2,562778 | 25/06/2000 | Coeur d'Acier | Asteraceae | Sonchus oleraceus L. |
| ACOE929 | France | Languedoc-Roussillon | Pyrénées-Orientales | Port-Vendres | 42,5205 | 3,105478 | 26/06/2000 | Coeur d'Acier | Cistaceae | Cistus monspeliensis L. |
| ACOE932 | France | Languedoc-Roussillon | Pyrénées-Orientales | Port-Vendres | 42,5205 | 3,105478 | 26/06/2000 | Coeur d'Acier | Apiaceae | Eryngium campestre L. |
| ACOE935 | France | Languedoc-Roussillon | Pyrénées-Orientales | Salses-le-Chateau | 42,834602 | 2,920978 | 27/06/2000 | Coeur d'Acier | Asteraceae | Asteraceae sp. |
| ACOE936 | France | Languedoc-Roussillon | Pyrénées-Orientales | Codalet | 42,6078 | 2,417878 | 28/06/2000 | Coeur d'Acier | Oenotheraceae | Oenothera sp. L. |
| ACOE939 | France | Languedoc-Roussillon | Pyrénées-Orientales | Codalet | 42,6078 | 2,417878 | 28/06/2000 | Coeur d'Acier | Crassulaceae | Sedum sp. |
| ACOE941 | France | Poitou-Charentes | Charente-Maritime | Chatelaillon-Plage | 46,050543 | -1,068575 | 24/07/2000 | Coeur d'Acier | Papaveraceae | Glaucium flavum Crantz |
| ACOE943 | France | Poitou-Charentes | Charente-Maritime | Chatelaillon-Plage | 46,050543 | -1,068575 | 24/07/2000 | Coeur d'Acier | Poaceae | Phragmites australis (Cav.) Steudel |
| ACOE944 | France | Poitou-Charentes | Charente-Maritime | Chatelaillon-Plage | 46,050543 | -1,068575 | 24/07/2000 | Coeur d'Acier | Fabaceae | Melilotus officinalis Lam. |
| ACOE945 | France | Bretagne | Morbihan | Locmariaquer | 47,556716 | -2,947754 | 25/07/2000 | Coeur d'Acier | Asteraceae | Senecio jacobaea L. |
| ACOE946 | France | Bretagne | Morbihan | Locmariaquer | 47,556716 | -2,947754 | 25/07/2000 | Coeur d'Acier | Asteraceae | Sonchus sp. L. |
| ACOE947 | France | Bretagne | Morbihan | Locmariaquer | 47,556716 | -2,947754 | 25/07/2000 | Coeur d'Acier | Apiaceae | Eryngium campestre L. |
| ACOE948 | France | Bretagne | Morbihan | Locmariaquer | 47,556716 | -2,947754 | 25/07/2000 | Coeur d'Acier | Salicaceae | Populus alba L. |
| ACOE952 | France | Bretagne | Morbihan | Locmariaquer | 47,556716 | -2,947754 | 25/07/2000 | Coeur d'Acier | Chenopodiaceae | Chenopodiaceae sp. Vent. |
| ACOE953 | France | Bretagne | Morbihan | Locmariaquer | 47,556716 | -2,947754 | 25/07/2000 | Coeur d'Acier | Chenopodiaceae | Beta vulgaris (L.) Arcangeli |
| ACOE955 | France | Bretagne | Morbihan | Plouharnel | 47,598301 | -3,112622 | 26/07/2000 | Coeur d'Acier | Salicaceae | Populus alba L. |
| ACOE956 | France | Bretagne | Morbihan | Plouharnel | 47,598301 | -3,112622 | 26/07/2000 | Coeur d'Acier | Salicaceae | Populus alba L. |
| ACOE957 | France | Bretagne | Morbihan | Saint-Pierre-Quiberon | 47,65227 | -3,133015 | 26/07/2000 | Coeur d'Acier | Oenotheraceae | Oenothera sp. L. |
| ACOE959 | France | Bretagne | Morbihan | Saint-Pierre-Quiberon | 47,65227 | -3,133015 | 26/07/2000 | Coeur d'Acier | Fabaceae | Ulex europaeus L. |
| ACOE960 | France | Bretagne | Morbihan | Saint-Pierre-Quiberon | 47,65227 | -3,133015 | 26/07/2000 | Coeur d'Acier | Oenotheraceae | Epilobium ciliatum Rafin. |
| ACOE962 | France | Bretagne | Morbihan | Saint-Pierre-Quiberon | 47,65227 | -3,133015 | 26/07/2000 | Coeur d'Acier | Rosaceae | Rubus sp. L. |
| ACOE963 | France | Bretagne | Morbihan | Saint-Pierre-Quiberon | 47,65227 | -3,133015 | 26/07/2000 | Coeur d'Acier | Oenotheraceae | Oenothera sp. L. |
| ACOE966 | France | Bretagne | Morbihan | Quiberon | 47,506596 | -3,149481 | 26/07/2000 | Coeur d'Acier | Plantaginaceae | Plantago coronopus L. |
| ACOE967 | France | Bretagne | Morbihan | Quiberon | 47,506596 | -3,149481 | 26/07/2000 | Coeur d'Acier | Asteraceae | Sonchus sp. L. |
| ACOE969 | France | Bretagne | Morbihan | Quiberon | 47,506596 | -3,149481 | 26/07/2000 | Coeur d'Acier | Asteraceae | Hypochoeris sp. L. |
| ACOE970 | France | Bretagne | Finistère | Tregunc | 47,80415 | -3,851273 | 27/07/2000 | Coeur d'Acier | Chenopodiaceae | Atriplex littoralis L. |
| ACOE971 | France | Bretagne | Finistère | Tregunc | 47,80415 | -3,851273 | 27/07/2000 | Coeur d'Acier | Asteraceae | Senecio jacobaea L. |
| ACOE973 | France | Bretagne | Finistère | Tregunc | 47,80415 | -3,851273 | 27/07/2000 | Coeur d'Acier | Apiaceae | Crithmum maritimum L. |
| ACOE974 | France | Bretagne | Finistère | Fouesnant | 47,888463 | -3,98064 | 27/07/2000 | Coeur d'Acier | Apiaceae | Pastinaca sativa (Req. ex Godron) Celak. |
| ACOE975 | France | Bretagne | Finistère | Fouesnant | 47,888463 | -3,98064 | 27/07/2000 | Coeur d'Acier | Compositae | Sonchus arvensis L. |
| ACOE977 | France | Bretagne | Finistère | Fouesnant | 47,888463 | -3,98064 | 27/07/2000 | Coeur d'Acier | Chenopodiaceae | Atriplex sp. L. |
| ACOE979 | France | Bretagne | Finistère | Fouesnant | 47,888463 | -3,98064 | 27/07/2000 | Coeur d'Acier | Rubiaceae | Galium aparine L. |
| ACOE980 | France | Bretagne | Finistère | Fouesnant | 47,888463 | -3,98064 | 27/07/2000 | Coeur d'Acier | Salicaceae | Populus canadensis Moench |
| ACOE984 | France | Bretagne | Finistère | Fouesnant | 47,847346 | -4,039867 | 28/07/2000 | Coeur d'Acier | Rosaceae | Malus domestica Borckh. |
| ACOE987 | France | Bretagne | Finistère | Fouesnant | 47,847346 | -4,039867 | 28/07/2000 | Coeur d'Acier | Rosaceae | Rubus sp. L. |
| ACOE988 | France | Bretagne | Finistère | Fouesnant | 47,847346 | -4,039867 | 28/07/2000 | Coeur d'Acier | Rosaceae | Sanguisorba minor Scop. |
| ACOE991 | France | Bretagne | Finistère | Fouesnant | 47,847346 | -4,039867 | 28/07/2000 | Coeur d'Acier | Asteraceae | Chardon sp. |
| ACOE992 | France | Bretagne | Finistère | Fouesnant | 47,847346 | -4,039867 | 28/07/2000 | Coeur d'Acier | Rosaceae | Prunus spinosa L. |
| ACOE993 | France | Bretagne | Finistère | Fouesnant | 47,847346 | -4,039867 | 28/07/2000 | Coeur d'Acier | Rosaceae | Rubus sp. L. |
| ACOE994 | France | Bretagne | Finistère | Fouesnant | 47,847346 | -4,039867 | 28/07/2000 | Coeur d'Acier | Fagaceae | Quercus robur L. |
| ACOE995 | France | Bretagne | Finistère | Fouesnant | 47,847346 | -4,039867 | 28/07/2000 | Coeur d'Acier | Iridaceae | Iris sp. L. |
| ACOE996 | France | Bretagne | Finistère | Fouesnant | 47,847346 | -4,039867 | 28/07/2000 | Coeur d'Acier | Solanaceae | Solanum sp. L. |
| ACOE997 | France | Bretagne | Finistère | Fouesnant | 47,847346 | -4,039867 | 28/07/2000 | Coeur d'Acier | Polygonaceae | Rumex sp. L. |
| ACOE998 | France | Bretagne | Finistère | Fouesnant | 47,847346 | -4,039867 | 28/07/2000 | Coeur d'Acier | Compositae | Leontodon autumnalis L. |
| ACOE999 | France | Bretagne | Finistère | Fouesnant | 47,847346 | -4,039867 | 28/07/2000 | Coeur d'Acier | Oenotheraceae | Epilobium ciliatum Rafin. |
| ACOE1001 | France | Bretagne | Finistère | Pont-l'Abbe | 47,520057 | -4,112779 | 28/07/2000 | Coeur d'Acier | Fagaceae | Quercus robur L. |
| ACOE1002 | France | Bretagne | Finistère | Pont-l'Abbe | 47,520057 | -4,112779 | 28/07/2000 | Coeur d'Acier | Polygonaceae | Rumex sp. L. |
| ACOE1003 | France | Bretagne | Finistère | Pont-l'Abbe | 47,520057 | -4,112779 | 28/07/2000 | Coeur d'Acier | Poaceae | Phragmites australis (Cav.) Steudel |
| ACOE1004 | France | Bretagne | Finistère | Pont-l'Abbe | 47,520057 | -4,112779 | 28/07/2000 | Coeur d'Acier | Fabaceae | Vicia sativa L. |
| ACOE1005 | France | Bretagne | Finistère | Pont-l'Abbe | 47,860694 | -4,184226 | 28/07/2000 | Coeur d'Acier | Salicaceae | Salix acuminata Miller |
| ACOE1006 | France | Bretagne | Finistère | Plomeur | 47,837638 | -4,353441 | 29/07/2000 | Coeur d'Acier | Labiatae | Thymus vulgaris L. |
| ACOE1007 | France | Bretagne | Finistère | Plomeur | 47,837638 | -4,353441 | 29/07/2000 | Coeur d'Acier | Rubiaceae | Galium arenarium Loisel. |
| ACOE1009 | France | Bretagne | Finistère | Plomeur | 47,837638 | -4,353441 | 29/07/2000 | Coeur d'Acier | Apiaceae | Pastinaca sativa L. |
| ACOE1010 | France | Bretagne | Finistère | Treogat | 47,900124 | -4,367651 | 30/07/2000 | Coeur d'Acier | Malvaceae | Lavatera cretica L. |
| ACOE1011 | France | Bretagne | Finistère | Treogat | 47,89235 | -4,353171 | 30/07/2000 | Coeur d'Acier | Apiaceae | Heracleum sp. L. |
| ACOE1012 | France | Bretagne | Finistère | Treogat | 47,89235 | -4,353171 | 30/07/2000 | Coeur d'Acier | Poaceae | Phragmites australis (Cav.) Steudel |
| ACOE1014 | France | Bretagne | Finistère | Plozevet | 47,986191 | -4,464225 | 30/07/2000 | Coeur d'Acier | Asteraceae | Picris echioides L. |
| ACOE1015 | France | Bretagne | Finistère | Plouhinec | 47,986191 | -4,464225 | 30/07/2000 | Coeur d'Acier | Apiaceae | Crithmum maritimum L. |
| ACOE1016 | France | Bretagne | Finistère | Plouhinec | 47,986191 | -4,464225 | 30/07/2000 | Coeur d'Acier | Apiaceae | Daucus carota L. |
| ACOE1017 | France | Bretagne | Finistère | Plouhinec | 47,986191 | -4,464225 | 30/07/2000 | Coeur d'Acier | Apiaceae | Daucus carota L. |
| ACOE1018 | France | Bretagne | Finistère | Plouhinec | 47,986191 | -4,464225 | 30/07/2000 | Coeur d'Acier | Asteraceae | Centaurea nigra L. |
| ACOE1020 | France | Bretagne | Finistère | Plouhinec | 47,986191 | -4,464225 | 30/07/2000 | Coeur d'Acier | Fabaceae | Trifolium pratense L. |
| ACOE1022 | France | Bretagne | Finistère | Plogoff | 48,024812 | -4,642031 | 31/07/2000 | Coeur d'Acier | Fabaceae | Ulex europaeus L. |
| ACOE1028 | France | Bretagne | Finistère | Plogoff | 48,036602 | -4,665123 | 31/07/2000 | Coeur d'Acier | Valerianaceae | Centranthus ruber (L.) DC. |
| ACOE1029 | France | Bretagne | Finistère | Plogoff | 48,036602 | -4,665123 | 31/07/2000 | Coeur d'Acier | Valerianaceae | Centranthus ruber (L.) DC. |
| ACOE1031 | France | Bretagne | Finistère | Locronan | 48,043979 | -4,133242 | 01/08/2000 | Coeur d'Acier | Caryophyllaceae | Silene sp. L. |
| ACOE1033 | France | Bretagne | Finistère | Locronan | 48,043979 | -4,133242 | 01/08/2000 | Coeur d'Acier | Asteraceae | Asteraceae sp. |
| ACOE1036 | France | Bretagne | Finistère | Locronan | 48,043979 | -4,133242 | 01/08/2000 | Coeur d'Acier | Rosaceae | Rubus sp. L. |
| ACOE1037 | France | Bretagne | Finistère | Locronan | 48,097801 | -4,207022 | 01/08/2000 | Coeur d'Acier | Aquifoliaceae | Ilex aquifolium L. |
| ACOE1038 | France | Bretagne | Finistère | Locronan | 48,097801 | -4,207022 | 01/08/2000 | Coeur d'Acier | Caprifoliaceae | Viburnum sp. L. |
| ACOE1041 | France | Bretagne | Finistère | Locronan | 48,097801 | -4,207022 | 01/08/2000 | Coeur d'Acier | Crassulaceae | Sedum sp. |
| ACOE1044 | France | Languedoc-Roussillon | Hérault | Fontanes | 43,794899 | 3,913678 | 21/08/2000 | Meusnier | Brassicaceae | Brassica sp. L. |
| ACOE1045 | France | Languedoc-Roussillon | Hérault | Fontanes | 43,794899 | 3,913678 | 21/08/2000 | Meusnier | Apocynaceae | Nerium oleander L. |
| ACOE1046 | France | Languedoc-Roussillon | Hérault | Fontanes | 43,794899 | 3,913678 | 21/08/2000 | Meusnier | Pinaceae | Pinus sp. L. |
| ACOE1047 | France | Languedoc-Roussillon | Pyrénées-Orientales | Le Barcares | 42,7869 | 3,036178 | 30/10/2000 | Coeur d'Acier | Asteraceae | Asteraceae sp. |
| ACOE1048 | France | Languedoc-Roussillon | Pyrénées-Orientales | Le Barcares | 42,7869 | 3,036178 | 30/10/2000 | Coeur d'Acier | Portulacaceae | Portulaca oleracea L. |
| ACOE1049 | France | Languedoc-Roussillon | Pyrénées-Orientales | Le Barcares | 42,7869 | 3,036178 | 30/10/2000 | Coeur d'Acier | Scrophulariaceae | Veronica sp. L. |
| ACOE1051 | France | Languedoc-Roussillon | Pyrénées-Orientales | Le Barcares | 42,7869 | 3,036178 | 30/10/2000 | Coeur d'Acier | Amaranthaceae | Amaranthus sp. L. |
| ACOE1052 | France | Languedoc-Roussillon | Pyrénées-Orientales | Le Barcares | 42,7869 | 3,036178 | 30/10/2000 | Coeur d'Acier | Fabaceae | Medicago sp. L. |
| ACOE1053 | France | Languedoc-Roussillon | Pyrénées-Orientales | Le Barcares | 42,7869 | 3,036178 | 30/10/2000 | Coeur d'Acier | Asteraceae | Asteraceae sp. |
| ACOE1054 | France | Languedoc-Roussillon | Pyrénées-Orientales | Le Barcares | 42,7869 | 3,036178 | 30/10/2000 | Coeur d'Acier | Asteraceae | Asteraceae sp. |
| ACOE1055 | France | Languedoc-Roussillon | Pyrénées-Orientales | Le Barcares | 42,7869 | 3,036178 | 30/10/2000 | Coeur d'Acier | Chenopodiaceae | Salsola kali L. |
| ACOE1056 | France | Languedoc-Roussillon | Pyrénées-Orientales | Le Barcares | 42,7869 | 3,036178 | 30/10/2000 | Coeur d'Acier | Papaveraceae | Glaucium flavum Crantz |
| ACOE1057 | France | Languedoc-Roussillon | Pyrénées-Orientales | Le Barcares | 42,7869 | 3,036178 | 30/10/2000 | Coeur d'Acier | Apiaceae | Echinophora spinosa L. |
| ACOE1058 | France | Languedoc-Roussillon | Pyrénées-Orientales | Le Barcares | 42,7869 | 3,036178 | 30/10/2000 | Coeur d'Acier | Salicaceae | Populus alba L. |
| ACOE1059 | France | Languedoc-Roussillon | Pyrénées-Orientales | Le Barcares | 42,7869 | 3,036178 | 30/10/2000 | Coeur d'Acier | Asteraceae | Centaurea aspera L. |
| ACOE1060 | France | Languedoc-Roussillon | Pyrénées-Orientales | Le Barcares | 42,7869 | 3,036178 | 30/10/2000 | Coeur d'Acier | Salicaceae | Populus nigra L. |
| ACOE1062 | France | Languedoc-Roussillon | Hérault | Montferrier-sur-Lez | 43,682624 | 3,874869 | 18/05/2001 | Coeur d'Acier | Salicaceae | Populus nigra L. |
| ACOE1063 | France | Languedoc-Roussillon | Hérault | Montferrier-sur-Lez | 43,682624 | 3,874869 | 18/05/2001 | Coeur d'Acier | Salicaceae | Populus nigra L. |
| ACOE1064 | France | Languedoc-Roussillon | Hérault | Montferrier-sur-Lez | 43,682624 | 3,874869 | 18/05/2001 | Coeur d'Acier | Euphorbiaceae | Euphorbia sp. L. |
| ACOE1066 | France | Languedoc-Roussillon | Hérault | Montferrier-sur-Lez | 43,682624 | 3,874869 | 18/05/2001 | Coeur d'Acier | Salicaceae | Populus alba L. |
| ACOE1067 | France | Languedoc-Roussillon | Hérault | Montferrier-sur-Lez | 43,682624 | 3,874869 | 18/05/2001 | Coeur d'Acier | Rosaceae | Malus sp. P. Miller |
| ACOE1069 | France | Languedoc-Roussillon | Hérault | Montferrier-sur-Lez | 43,682624 | 3,874869 | 18/05/2001 | Coeur d'Acier | Cruciferaceae | Cruciferaceae sp. |
| ACOE1071 | France | Languedoc-Roussillon | Aude | Quillan | 42,874199 | 2,182978 | 21/05/2001 | Coeur d'Acier | Aceraceae | Acer sp. L. |
| ACOE1073 | France | Languedoc-Roussillon | Aude | Quillan | 42,874199 | 2,182978 | 21/05/2001 | Coeur d'Acier | Asteraceae | Sonchus sp. L. |
| ACOE1074 | France | Languedoc-Roussillon | Aude | Quillan | 42,874199 | 2,182978 | 21/05/2001 | Coeur d'Acier | Araliaceae | Hedera helix L. |
| ACOE1077 | France | Languedoc-Roussillon | Aude | Quillan | 42,874199 | 2,182978 | 21/05/2001 | Coeur d'Acier | Rosaceae | Prunus spinosa L. |
| ACOE1078 | France | Languedoc-Roussillon | Aude | Quillan | 42,874199 | 2,182978 | 21/05/2001 | Coeur d'Acier | Apiaceae | Apiaceae sp. Lindl. |
| ACOE1080 | France | Languedoc-Roussillon | Aude | Coudons | 42,8798 | 2,0757 | 22/05/2001 | Coeur d'Acier | Euphorbiaceae | Euphorbia sp. L. |
| ACOE1083 | France | Languedoc-Roussillon | Aude | Coudons | 42,8634 | 2,123578 | 22/05/2001 | Coeur d'Acier | Urticaceae | Urtica sp. L. |
| ACOE1085 | France | Languedoc-Roussillon | Aude | Coudons | 42,8634 | 2,123578 | 22/05/2001 | Coeur d'Acier | Asteraceae | Sonchus sp. L. |
| ACOE1086 | France | Languedoc-Roussillon | Aude | Coudons | 42,8634 | 2,123578 | 22/05/2001 | Coeur d'Acier | Rubiaceae | Galium aparine L. |
| ACOE1087 | France | Languedoc-Roussillon | Aude | Coudons | 42,8634 | 2,123578 | 22/05/2001 | Coeur d'Acier | Asteraceae | Senecio sp. L. |
| ACOE1088 | France | Languedoc-Roussillon | Aude | Coudons | 42,8634 | 2,123578 | 22/05/2001 | Coeur d'Acier |  |  |
| ACOE1091 | France | Languedoc-Roussillon | Aude | Belvianes-et-Cavirac | 42,8508 | 2,195578 | 24/05/2001 | Coeur d'Acier | Fabaceae | Vicia sp. L. |
| ACOE1092 | France | Languedoc-Roussillon | Aude | Belvianes-et-Cavirac | 42,8508 | 2,195578 | 24/05/2001 | Coeur d'Acier | Poaceae | Poaceae sp. Barnhart |
| ACOE1093 | France | Languedoc-Roussillon | Aude | Belvianes-et-Cavirac | 42,8508 | 2,195578 | 24/05/2001 | Coeur d'Acier | Dipsacaceae | Dipsacus sp. L. |
| ACOE1094 | France | Languedoc-Roussillon | Aude | Belvianes-et-Cavirac | 42,8508 | 2,195578 | 24/05/2001 | Coeur d'Acier | Caryophyllaceae | Caryophyllaceae sp. Juss. |
| ACOE1099 | France | Languedoc-Roussillon | Aude | Belvianes-et-Cavirac | 42,8508 | 2,195578 | 24/05/2001 | Coeur d'Acier | Malvaceae | Malva sp. L. |
| ACOE1100 | France | Languedoc-Roussillon | Hérault | Montpellier | 43,61717 | 3,858032 | 26/04/2001 | Coeur d'Acier | Apiaceae | Daucus carota L. |
| ACOE1101 | France | Languedoc-Roussillon | Hérault | Montpellier | 43,61717 | 3,858032 | 02/05/2001 | Coeur d'Acier | Fagaceae | Quercus ilex L. |
| ACOE1109 | France | Languedoc-Roussillon | Aude | Coudons | 42,870425 | 2,101598 | 29/05/2001 | Coeur d'Acier | Fabaceae | Fabaceae sp. Lindl. |
| ACOE1112 | France | Languedoc-Roussillon | Aude | Coudons | 42,870425 | 2,101598 | 29/05/2001 | Coeur d'Acier | Polygonaceae | Rumex sp. L. |
| ACOE1114 | France | Languedoc-Roussillon | Aude | Coudons | 42,870425 | 2,101598 | 29/05/2001 | Coeur d'Acier | Pinaceae | Pinus sp. L. |
| ACOE1115 | France | Languedoc-Roussillon | Aude | Coudons | 42,870425 | 2,101598 | 29/05/2001 | Coeur d'Acier | Fabaceae | Trifolium sp. L. |
| ACOE1116 | France | Languedoc-Roussillon | Aude | Bessede-de-Sault | 42,778771 | 2,152069 | 29/05/2001 | Coeur d'Acier | Dipsacaceae | Dipsacaceae sp. Juss. |
| ACOE1121 | France | Languedoc-Roussillon | Aude | Belvis | 42,849899 | 2,074978 | 29/05/2001 | Coeur d'Acier | Aceraceae | Acer sp. L. |
| ACOE1123 | France | Languedoc-Roussillon | Aude | Belvis | 42,849899 | 2,074978 | 29/05/2001 | Coeur d'Acier |  |  |
| ACOE1124 | France | Languedoc-Roussillon | Aude | Belvis | 42,849899 | 2,074978 | 29/05/2001 | Coeur d'Acier | Rosaceae | Prunus sp. L. |
| ACOE1125 | France | Languedoc-Roussillon | Aude | Belvis | 42,849899 | 2,074978 | 29/05/2001 | Coeur d'Acier | Oleaceae | Fraxinus sp. L. |
| ACOE1126 | France | Languedoc-Roussillon | Aude | Espezel | 42,821098 | 2,019178 | 30/05/2001 | Coeur d'Acier | Caryophyllaceae | Caryophyllaceae sp. Juss. |
| ACOE1127 | France | Languedoc-Roussillon | Aude | Espezel | 42,821098 | 2,019178 | 30/05/2001 | Coeur d'Acier |  |  |
| ACOE1128 | France | Languedoc-Roussillon | Aude | Espezel | 42,821098 | 2,019178 | 30/05/2001 | Coeur d'Acier | Asteraceae | Arctium sp. L. |
| ACOE1129 | France | Languedoc-Roussillon | Aude | Espezel | 42,821098 | 2,019178 | 30/05/2001 | Coeur d'Acier | Apiaceae | Apiaceae sp. Lindl. |
| ACOE1131 | France | Languedoc-Roussillon | Aude | Espezel | 42,821098 | 2,019178 | 30/05/2001 | Coeur d'Acier | Oleaceae | Fraxinus sp. L. |
| ACOE1132 | France | Languedoc-Roussillon | Aude | Espezel | 42,821098 | 2,019178 | 30/05/2001 | Coeur d'Acier | Polygonaceae | Rumex sp. L. |
| ACOE1136 | France | Midi-Pyrénées | Ariège | Belesta | 42,9039 | 1,933678 | 30/01/2001 | Coeur d'Acier | Rosaceae | Rubus sp. L. |
| ACOE1137 | France | Midi-Pyrénées | Ariège | Belesta | 42,9039 | 1,933678 | 30/01/2001 | Coeur d'Acier | Euphorbiaceae | Euphorbia sp. L. |
| ACOE1139 | France | Languedoc-Roussillon | Aude | Belcaire | 42,815701 | 1,957978 | 30/05/2001 | Coeur d'Acier | Urticaceae | Urtica sp. L. |
| ACOE1140 | France | Languedoc-Roussillon | Aude | Belcaire | 42,815701 | 1,957978 | 30/05/2001 | Coeur d'Acier | Salicaceae | Populus sp. L. |
| ACOE1141 | France | Languedoc-Roussillon | Aude | Belcaire | 42,815701 | 1,957978 | 31/05/2001 | Coeur d'Acier | Dipsacaceae | Dipsacus sp. L. |
| ACOE1142 | France | Languedoc-Roussillon | Aude | Belcaire | 42,815701 | 1,957978 | 31/05/2001 | Coeur d'Acier | Poaceae | Poaceae sp. Barnhart |
| ACOE1144 | France | Languedoc-Roussillon | Aude | Belcaire | 42,815701 | 1,957978 | 31/05/2001 | Coeur d'Acier | Caprifoliaceae | Lonicera sp. L. |
| ACOE1145 | France | Languedoc-Roussillon | Aude | Comus | 42,813 | 1,892278 | 31/05/2001 | Coeur d'Acier | Salicaceae | Salix sp. L. |
| ACOE1148 | France | Haute-Normandie | Seine-Maritime | Heurteauville | 49,445999 | 0,812278 | 13/06/2001 | Coeur d'Acier | Asteraceae | Achillea millefolium L. |
| ACOE1149 | France | Haute-Normandie | Seine-Maritime | Heurteauville | 49,445999 | 0,812278 | 13/06/2001 | Coeur d'Acier | Asteraceae | Achillea millefolium L. |
| ACOE1150 | France | Haute-Normandie | Seine-Maritime | Heurteauville | 49,445999 | 0,812278 | 13/06/2001 | Coeur d'Acier | Caprifoliaceae | Sambucus nigra L. |
| ACOE1152 | France | Haute-Normandie | Seine-Maritime | Heurteauville | 49,445999 | 0,812278 | 13/06/2001 | Coeur d'Acier | Caprifoliaceae | Viburnum opulus L. |
| ACOE1153 | France | Haute-Normandie | Seine-Maritime | Heurteauville | 49,445999 | 0,812278 | 13/06/2001 | Coeur d'Acier | Salicaceae | Salix sp. L. |
| ACOE1154 | France | Haute-Normandie | Seine-Maritime | Heurteauville | 49,445999 | 0,812278 | 13/06/2001 | Coeur d'Acier | Corylaceae | Corylus avellana L. |
| ACOE1155 | France | Haute-Normandie | Seine-Maritime | Heurteauville | 49,445999 | 0,812278 | 13/06/2001 | Coeur d'Acier | Salicaceae | Populus tremula L. |
| ACOE1157 | France | Haute-Normandie | Seine-Maritime | Heurteauville | 49,445999 | 0,812278 | 13/06/2001 | Coeur d'Acier | Salicaceae | Salix sp. L. |
| ACOE1158 | France | Haute-Normandie | Seine-Maritime | Heurteauville | 49,445999 | 0,812278 | 13/06/2001 | Coeur d'Acier | Aceraceae | Acer campestre L. |
| ACOE1159 | France | Haute-Normandie | Seine-Maritime | Heurteauville | 49,445999 | 0,812278 | 13/06/2001 | Coeur d'Acier | Grossulariaceae | Ribes sp. L. |
| ACOE1160 | France | Haute-Normandie | Seine-Maritime | Grand-Couronne | 49,351608 | 1,025269 | 14/06/2001 | Coeur d'Acier | Salicaceae | Salix sp. L. |
| ACOE1161 | France | Haute-Normandie | Seine-Maritime | Grand-Couronne | 49,351608 | 1,025269 | 14/06/2001 | Coeur d'Acier | Corylaceae | Carpinus betulus L. |
| ACOE1164 | France | Haute-Normandie | Seine-Maritime | Grand-Couronne | 49,351608 | 1,025269 | 14/06/2001 | Coeur d'Acier | Aquifoliaceae | Ilex aquifolium L. |
| ACOE1165 | France | Haute-Normandie | Seine-Maritime | Grand-Couronne | 49,351608 | 1,025269 | 14/06/2001 | Coeur d'Acier | Aquifoliaceae | Ilex aquifolium L. |
| ACOE1166 | France | Haute-Normandie | Seine-Maritime | Grand-Couronne | 49,351608 | 1,025269 | 14/06/2001 | Coeur d'Acier | Rosaceae | Prunus sp. L. |
| ACOE1169 | France | Haute-Normandie | Seine-Maritime | Yville-sur-Seine | 49,3992 | 0,880678 | 14/06/2001 | Coeur d'Acier | Asteraceae | Tanacetum sp. L. |
| ACOE1171 | France | Haute-Normandie | Seine-Maritime | Yville-sur-Seine | 49,3992 | 0,880678 | 14/06/2001 | Coeur d'Acier | Apiaceae | Apiaceae sp. Lindl. |
| ACOE1172 | France | Haute-Normandie | Seine-Maritime | Yville-sur-Seine | 49,3992 | 0,880678 | 14/06/2001 | Coeur d'Acier | Dipsacaceae | Dipsacus fullonum L. |
| ACOE1173 | France | Haute-Normandie | Seine-Maritime | Yville-sur-Seine | 49,3992 | 0,880678 | 14/06/2001 | Coeur d'Acier | Polygonaceae | Rumex sp. L. |
| ACOE1175 | France | Haute-Normandie | Seine-Maritime | Belbeuf | 49,372814 | 1,134327 | 15/06/2001 | Coeur d'Acier | Valerianaceae | Centranthus sp. A.P. de Candolle |
| ACOE1176 | France | Haute-Normandie | Seine-Maritime | Belbeuf | 49,372814 | 1,134327 | 15/06/2001 | Coeur d'Acier | Labiaceae | Labiaceae sp. Dulac |
| ACOE1178 | France | Haute-Normandie | Seine-Maritime | Belbeuf | 49,372814 | 1,134327 | 15/06/2001 | Coeur d'Acier | Asteraceae | Tragopogon pratensis L. |
| ACOE1179 | France | Haute-Normandie | Seine-Maritime | Belbeuf | 49,372814 | 1,134327 | 15/06/2001 | Coeur d'Acier | Asteraceae | Asteraceae sp. |
| ACOE1180 | France | Haute-Normandie | Seine-Maritime | Belbeuf | 49,372814 | 1,134327 | 15/06/2001 | Coeur d'Acier | Rosaceae | Prunus spinosa L. |
| ACOE1185 | France | Haute-Normandie | Seine-Maritime | Saint-Wandrille-Rancon | 49,527897 | 0,768178 | 15/06/2001 | Coeur d'Acier | Oenotheraceae | Epilobium sp. L. |
| ACOE1186 | France | Haute-Normandie | Seine-Maritime | Tancarville | 49,4874 | 0,451378 | 18/06/2001 | Coeur d'Acier | Asteraceae | Asteraceae sp. |
| ACOE1188 | France | Haute-Normandie | Seine-Maritime | Tancarville | 49,4874 | 0,451378 | 18/06/2001 | Coeur d'Acier | Caryophyllaceae | Caryophyllaceae sp. Juss. |
| ACOE1190 | France | Haute-Normandie | Seine-Maritime | Tancarville | 49,4874 | 0,451378 | 18/06/2001 | Coeur d'Acier | Urticaceae | Urtica sp. L. |
| ACOE1191 | France | Haute-Normandie | Seine-Maritime | Tancarville | 49,4874 | 0,451378 | 18/06/2001 | Coeur d'Acier | Apiaceae | Apiaceae sp. Lindl. |
| ACOE1192 | France | Haute-Normandie | Seine-Maritime | Tancarville | 49,4874 | 0,451378 | 18/06/2001 | Coeur d'Acier | Rosaceae | Rubus sp. L. |
| ACOE1193 | France | Haute-Normandie | Seine-Maritime | Tancarville | 49,4874 | 0,451378 | 18/06/2001 | Coeur d'Acier | Rosaceae | Rubus sp. L. |
| ACOE1194 | France | Haute-Normandie | Seine-Maritime | Saint-Jouin-Bruneval | 49,662899 | 0,166078 | 18/06/2001 | Coeur d'Acier | Apiaceae | Crithmum maritimum L. |
| ACOE1195 | France | Haute-Normandie | Seine-Maritime | La Fontelaye | 49,689899 | 0,952678 | 19/06/2001 | Coeur d'Acier |  |  |
| ACOE1196 | France | Haute-Normandie | Seine-Maritime | La Fontelaye | 49,689899 | 0,952678 | 19/06/2001 | Coeur d'Acier | Fagaceae | Quercus robur L. |
| ACOE1197 | France | Haute-Normandie | Seine-Maritime | La Fontelaye | 49,689899 | 0,952678 | 19/06/2001 | Coeur d'Acier | Fagaceae | Quercus robur L. |
| ACOE1198 | France | Haute-Normandie | Seine-Maritime | La Fontelaye | 49,689899 | 0,952678 | 19/06/2001 | Coeur d'Acier | Brassicaceae | Brassicaceae sp. Burnett |
| ACOE1201 | France | Haute-Normandie | Seine-Maritime | Imbleville | 49,714199 | 0,950878 | 19/06/2001 | Coeur d'Acier | Fagaceae | Fagus sylvatica L. |
| ACOE1206 | France | Haute-Normandie | Seine-Maritime | Imbleville | 49,714199 | 0,950878 | 19/06/2001 | Coeur d'Acier | Rosaceae | Prunus spinosa L. |
| ACOE1208 | France | Haute-Normandie | Seine-Maritime | Veulettes-sur-Mer | 49,846497 | 0,594478 | 20/06/2001 | Coeur d'Acier | Brassicaceae | Brassica sp. L. |
| ACOE1209 | France | Haute-Normandie | Seine-Maritime | Veulettes-sur-Mer | 49,846497 | 0,594478 | 20/06/2001 | Coeur d'Acier | Asteraceae | Senecio sp. L. |
| ACOE1211 | France | Haute-Normandie | Seine-Maritime | Veulettes-sur-Mer | 49,846497 | 0,594478 | 20/06/2001 | Coeur d'Acier | Asteraceae | Achillea sp. L. |
| ACOE1212 | France | Haute-Normandie | Seine-Maritime | Veulettes-sur-Mer | 49,846497 | 0,594478 | 20/06/2001 | Coeur d'Acier | Asteraceae | Asteraceae sp. |
| ACOE1215 | France | Haute-Normandie | Seine-Maritime | Les Petites-Dalles | 49,816666 | 0,533333 | 20/06/2001 | Coeur d'Acier | Asteraceae | Centaurea sp. L. |
| ACOE1216 | France | Haute-Normandie | Seine-Maritime | Les Petites-Dalles | 49,816666 | 0,533333 | 20/06/2001 | Coeur d'Acier | Aceraceae | Acer sp. L. |
| ACOE1217 | France | Haute-Normandie | Seine-Maritime | Les Petites-Dalles | 49,816666 | 0,533333 | 20/06/2001 | Coeur d'Acier | Asteraceae | Asteraceae sp. |
| ACOE1218 | France | Haute-Normandie | Seine-Maritime | Les Petites-Dalles | 49,816666 | 0,533333 | 20/06/2001 | Coeur d'Acier | Urticaceae | Urtica sp. L. |
| ACOE1219 | France | Haute-Normandie | Seine-Maritime | Rouen | 49,437901 | 1,089478 | 21/06/2001 | Coeur d'Acier | Betulaceae | Betula pendula Roth |
| ACOE1220 | France | Haute-Normandie | Seine-Maritime | Saint-Wandrille-Rancon | 49,527897 | 0,768178 | 22/06/2001 | Coeur d'Acier | Polygonaceae | Rumex sp. L. |
| ACOE1222 | France | Haute-Normandie | Seine-Maritime | Saint-Wandrille-Rancon | 49,527897 | 0,768178 | 22/06/2001 | Coeur d'Acier | Asteraceae | Asteraceae sp. |
| ACOE1224 | France | Haute-Normandie | Seine-Maritime | Saint-Wandrille-Rancon | 49,527897 | 0,768178 | 22/06/2001 | Coeur d'Acier | Valerianaceae | Valeriana sp. L. |
| ACOE1225 | France | Haute-Normandie | Seine-Maritime | Saint-Wandrille-Rancon | 49,527897 | 0,768178 | 22/06/2001 | Coeur d'Acier | Valerianaceae | Valeriana sp. L. |
| ACOE1229 | France | Haute-Normandie | Seine-Maritime | Sainte-Marguerite-sur-Mer | 49,9077 | 0,947278 | 23/06/2001 | Coeur d'Acier | Betulaceae | Betula alba L. |
| ACOE1230 | France | Haute-Normandie | Seine-Maritime | Veules-les-Roses | 49,873501 | 0,799678 | 23/06/2001 | Coeur d'Acier |  |  |
| ACOE1231 | France | Haute-Normandie | Seine-Maritime | Veules-les-Roses | 49,873501 | 0,799678 | 23/06/2001 | Coeur d'Acier | Salicaceae | Salix fragilis L. |
| ACOE1232 | France | Haute-Normandie | Seine-Maritime | Veules-les-Roses | 49,873501 | 0,799678 | 23/06/2001 | Coeur d'Acier | Apiaceae | Apiaceae sp. Lindl. |
| ACOE1233 | France | Haute-Normandie | Seine-Maritime | Veules-les-Roses | 49,873501 | 0,799678 | 23/06/2001 | Coeur d'Acier | Tiliaceae | Tilia x-vulgaris Hayne |
| ACOE1234 | France | Haute-Normandie | Seine-Maritime | Veules-les-Roses | 49,873501 | 0,799678 | 23/06/2001 | Coeur d'Acier | Betulaceae | Alnus glutinosa (L.) Gaertn. |
| ACOE1238 | France | Auvergne | Allier | Ebreuil | 46,116001 | 3,089278 | 16/07/2001 | Coeur d'Acier | Asteraceae | Taraxacum sp. F.H. Wiggers |
| ACOE1239 | France | Auvergne | Allier | Ebreuil | 46,116001 | 3,089278 | 16/07/2001 | Coeur d'Acier | Pinaceae | Pinus sp. L. |
| ACOE1240 | France | Auvergne | Allier | Ebreuil | 46,116001 | 3,089278 | 16/07/2001 | Coeur d'Acier | Asteraceae | Asteraceae sp. |
| ACOE1241 | France | Auvergne | Allier | Ebreuil | 46,116001 | 3,089278 | 16/07/2001 | Coeur d'Acier | Fagaceae | Quercus sp. L. |
| ACOE1242 | France | Auvergne | Allier | Ebreuil | 46,116001 | 3,089278 | 16/07/2001 | Coeur d'Acier | Rosaceae | Prunus avium L. |
| ACOE1243 | France | Auvergne | Allier | Chouvigny | 46,105523 | 3,025369 | 16/07/2001 | Coeur d'Acier | Rosaceae | Prunus spinosa L. |
| ACOE1244 | France | Auvergne | Allier | Chouvigny | 46,105523 | 3,025369 | 16/07/2001 | Coeur d'Acier | Asteraceae | Senecio jacobaea L. |
| ACOE1245 | France | Auvergne | Allier | Chouvigny | 46,105523 | 3,025369 | 16/07/2001 | Coeur d'Acier | Fabaceae | Trifolium sp. L. |
| ACOE1246 | France | Auvergne | Allier | Chouvigny | 46,105523 | 3,025369 | 16/07/2001 | Coeur d'Acier | Rosaceae | Crataegus sp. L. |
| ACOE1247 | France | Auvergne | Allier | Chouvigny | 46,105523 | 3,025369 | 16/07/2001 | Coeur d'Acier | Corylaceae | Corylus avellana L. |
| ACOE1248 | France | Auvergne | Allier | Chouvigny | 46,105523 | 3,025369 | 16/07/2001 | Coeur d'Acier |  |  |
| ACOE1253 | France | Limousin | Creuse | Aubusson | 45,940213 | 2,152429 | 17/07/2001 | Coeur d'Acier | Betulaceae | Betula sp. L. |
| ACOE1256 | France | Limousin | Creuse | Saint-Marc-a-Frongier | 45,929703 | 2,121778 | 17/07/2001 | Coeur d'Acier | Rosaceae | Prunus spinosa L. |
| ACOE1257 | France | Limousin | Creuse | Saint-Marc-a-Frongier | 45,929703 | 2,121778 | 17/07/2001 | Coeur d'Acier | Rosaceae | Crataegus sp. L. |
| ACOE1259 | France | Limousin | Creuse | Saint-Marc-a-Frongier | 45,929703 | 2,121778 | 17/07/2001 | Coeur d'Acier | Fagaceae | Quercus sp. L. |
| ACOE1260 | France | Limousin | Creuse | Saint-Marc-a-Frongier | 45,929703 | 2,121778 | 17/07/2001 | Coeur d'Acier | Fagaceae | Quercus sp. L. |
| ACOE1261 | France | Limousin | Creuse | Vallieres | 45,9063 | 2,038078 | 17/07/2001 | Coeur d'Acier | Pinaceae | Pinus sp. L. |
| ACOE1266 | France | Limousin | Creuse | Vallieres | 45,9063 | 2,038078 | 17/07/2001 | Coeur d'Acier | Rosaceae | Filipendula ulmaria (L.) Maxim. |
| ACOE1268 | France | Limousin | Creuse | Saint-Quentin-la-Chabanne | 45,864899 | 2,153278 | 18/07/2001 | Coeur d'Acier | Fabaceae | Cytisus sp. Desfontaines |
| ACOE1273 | France | Limousin | Creuse | Saint-Quentin-la-Chabanne | 45,864899 | 2,153278 | 18/07/2001 | Coeur d'Acier | Apiaceae | Apiaceae sp. Lindl. |
| ACOE1275 | France | Limousin | Creuse | Gentioux-Pigerolles | 45,784801 | 1,993978 | 18/07/2001 | Coeur d'Acier | Araliaceae | Hedera helix L. |
| ACOE1276 | France | Limousin | Creuse | Gentioux-Pigerolles | 45,784801 | 1,993978 | 18/07/2001 | Coeur d'Acier | Rosaceae | Spiraea sp. L. |
| ACOE1277 | France | Limousin | Creuse | Gentioux-Pigerolles | 45,784801 | 1,993978 | 18/07/2001 | Coeur d'Acier | Polygonaceae | Rumex sp. L. |
| ACOE1278 | France | Limousin | Creuse | Gentioux-Pigerolles | 45,784801 | 1,993978 | 18/07/2001 | Coeur d'Acier | Polygonaceae | Rumex sp. L. |
| ACOE1279 | France | Limousin | Creuse | Gentioux-Pigerolles | 45,784801 | 1,993978 | 18/07/2001 | Coeur d'Acier | Asteraceae | Cirsium sp. P. Miller |
| ACOE1282 | France | Limousin | Creuse | Faux-la-Montagne | 45,751499 | 1,935478 | 18/07/2001 | Coeur d'Acier | Fagaceae | Quercus sp. L. |
| ACOE1283 | France | Limousin | Creuse | Peyrat-la-Noniere | 46,089001 | 2,258578 | 19/07/2001 | Coeur d'Acier | Fabaceae | Trifolium sp. L. |
| ACOE1285 | France | Limousin | Creuse | Peyrat-la-Noniere | 46,089001 | 2,258578 | 19/07/2001 | Coeur d'Acier |  |  |
| ACOE1286 | France | Limousin | Creuse | Peyrat-la-Noniere | 46,089001 | 2,258578 | 19/07/2001 | Coeur d'Acier | Oenotheraceae | Epilobium sp. L. |
| ACOE1287 | France | Limousin | Creuse | Peyrat-la-Noniere | 46,089001 | 2,258578 | 19/07/2001 | Coeur d'Acier | Caprifoliaceae | Sambucus nigra L. |
| ACOE1289 | France | Limousin | Creuse | Peyrat-la-Noniere | 46,089001 | 2,258578 | 19/07/2001 | Coeur d'Acier | Oenotheraceae | Epilobium sp. L. |
| ACOE1296 | France | Limousin | Creuse | Chenerailles | 46,1133 | 2,178478 | 19/07/2001 | Coeur d'Acier | Salicaceae | Populus tremula L. |
| ACOE1297 | France | Limousin | Creuse | Chenerailles | 46,1133 | 2,178478 | 19/07/2001 | Coeur d'Acier | Asteraceae | Asteraceae sp. |
| ACOE1298 | France | Limousin | Creuse | Chenerailles | 46,1133 | 2,178478 | 19/07/2001 | Coeur d'Acier | Apiaceae | Apiaceae sp. Lindl. |
| ACOE1299 | France | Limousin | Creuse | Chenerailles | 46,1133 | 2,178478 | 19/07/2001 | Coeur d'Acier | Fagaceae | Castanea sativa Miller |
| ACOE1300 | France | Limousin | Creuse | Chenerailles | 46,1133 | 2,178478 | 19/07/2001 | Coeur d'Acier | Apiaceae | Apiaceae sp. Lindl. |
| ACOE1302 | France | Bretagne | Ille-et-Vilaine | Le Rheu | 48,1 | -1,796502 | 04/09/2001 | Coeur d'Acier | Asteraceae | Asteraceae sp. |
| ACOE1305 | France | Bretagne | Ille-et-Vilaine | Le Rheu | 48,1 | -1,796502 | 04/09/2001 | Coeur d'Acier | Fabaceae | Ulex europaeus L. |
| ACOE1306 | France | Bretagne | Ille-et-Vilaine | Le Rheu | 48,084464 | -1,743821 | 05/09/2001 | Coeur d'Acier | Asteraceae | Sonchus sp. L. |
| ACOE1307 | France | Bretagne | Ille-et-Vilaine | Le Rheu | 48,0798 | -1,773422 | 05/09/2001 | Coeur d'Acier | Asteraceae | Sonchus sp. L. |
| ACOE1308 | France | Bretagne | Ille-et-Vilaine | Le Rheu | 48,0798 | -1,773422 | 05/09/2001 | Coeur d'Acier | Asteraceae | Artemisia sp. L. |
| ACOE1309 | France | Bretagne | Ille-et-Vilaine | Le Rheu | 48,0798 | -1,773422 | 05/09/2001 | Coeur d'Acier | Fagaceae | Quercus sp. L. |
| ACOE1310 | France | Bretagne | Ille-et-Vilaine | Le Rheu | 48,091257 | -1,793306 | 05/09/2001 | Coeur d'Acier | Magnoliaceae | Liriodendron tulipifera L. |
| ACOE1311 | France | Bretagne | Ille-et-Vilaine | Le Rheu | 48,091257 | -1,793306 | 05/09/2001 | Coeur d'Acier | Oenotheraceae | Epilobium sp. L. |
| ACOE1314 | France | Bretagne | Ille-et-Vilaine | Le Rheu | 48,091257 | -1,793306 | 05/09/2001 | Coeur d'Acier | Araliaceae | Hedera helix L. |
| ACOE1316 | France | Bretagne | Ille-et-Vilaine | Le Rheu | 48,1 | -1,796502 | 05/09/2001 | Coeur d'Acier | Rosaceae | Spiraea vanhouttei Zabel |
| ACOE1318 | France | Bretagne | Ille-et-Vilaine | Le Rheu | 48,091257 | -1,793306 | 05/09/2001 | Coeur d'Acier | Asteraceae | Sonchus sp. L. |
| ACOE1320 | France | Bretagne | Ille-et-Vilaine | Le Rheu | 48,091257 | -1,793306 | 05/09/2001 | Coeur d'Acier | Salicaceae | Populus nigra L. |
| ACOE1322 | France | Bretagne | Ille-et-Vilaine | Montfort | 48,136501 | -1,954322 | 05/09/2001 | Coeur d'Acier | Rosaceae | Crataegus sp. L. |
| ACOE1323 | France | Bretagne | Ille-et-Vilaine | Montfort | 48,136501 | -1,954322 | 05/09/2001 | Coeur d'Acier | Salicaceae | Populus alba L. |
| ACOE1325 | France | Bretagne | Morbihan | Saint-Pierre-Quiberon | 47,65227 | -3,133015 | 08/09/2001 | Coeur d'Acier | Iridaceae | Iris sp. L. |
| ACOE1326 | France | Bretagne | Morbihan | Saint-Pierre-Quiberon | 47,65227 | -3,133015 | 08/09/2001 | Coeur d'Acier | Oenotheraceae | Oenothera sp. L. |
| ACOE1327 | France | Bretagne | Morbihan | Saint-Pierre-Quiberon | 47,65227 | -3,133015 | 08/09/2001 | Coeur d'Acier | Asteraceae | Sonchus sp. L. |
| ACOE1328 | France | Bretagne | Morbihan | Saint-Pierre-Quiberon | 47,65227 | -3,133015 | 08/09/2001 | Coeur d'Acier |  |  |
| ACOE1329 | France | Bretagne | Morbihan | Plouharnel | 47,598301 | -3,112622 | 08/09/2001 | Coeur d'Acier | Salicaceae | Populus alba L. |
| ACOE1349 | France | Languedoc-Roussillon | Hérault | Prades-le-Lez | 43,716663 | 3,846965 | 13/05/2002 | Coeur d'Acier | Rosaceae | Geum urbanum L. |
| ACOE1350 | France | Languedoc-Roussillon | Hérault | Prades-le-Lez | 43,691487 | 3,866609 | 13/05/2002 | Coeur d'Acier | Cruciferaceae | Cruciferaceae sp. |
| ACOE1351 | France | Languedoc-Roussillon | Hérault | Prades-le-Lez | 43,697701 | 3,862378 | 13/05/2002 | Coeur d'Acier | Asteraceae | Sonchus sp. L. |
| ACOE1352 | France | Languedoc-Roussillon | Hérault | Prades-le-Lez | 43,697701 | 3,862378 | 13/05/2002 | Coeur d'Acier | Aceraceae | Acer campestre L. |
| ACOE1353 | France | Centre | Loiret | Artenay | 48,0816 | 1,879678 | 18/05/2002 | Coeur d'Acier | Asteraceae | Arctium lappa L. |
| ACOE1354 | France | Centre | Loiret | Artenay | 48,0816 | 1,879678 | 18/05/2002 | Coeur d'Acier | Asteraceae | Arctium lappa L. |
| ACOE1355 | France | Centre | Loiret | Orleans | 47,895277 | 1,940006 | 19/05/2002 | Coeur d'Acier | Rosaceae | Geum urbanum L. |
| ACOE1359 | France | Centre | Loiret | Fleury-les-Aubrais | 47,9286 | 1,917478 | 20/05/2002 | Coeur d'Acier | Caprifoliaceae | Lonicera sp. L. |
| ACOE1360 | France | Centre | Loiret | Fleury-les-Aubrais | 47,9286 | 1,917478 | 20/05/2002 | Coeur d'Acier | Hydrangeaceae | Philadelphus sp. L. |
| ACOE1361 | France | Centre | Loiret | Fleury-les-Aubrais | 47,9286 | 1,917478 | 20/05/2002 | Coeur d'Acier | Betulaceae | Betula sp. L. |
| ACOE1362 | France | Centre | Loiret | Fleury-les-Aubrais | 47,9286 | 1,917478 | 20/05/2002 | Coeur d'Acier | Grossulariaceae | Ribes nigrum L. |
| ACOE1364 | France | Languedoc-Roussillon | Hérault | Montferrier-sur-Lez | 43,682624 | 3,874869 | 05/05/2002 | Coeur d'Acier | Asteraceae | Cichorium intybus L. |
| ACOE1367 | Greece | Peloponnisos (el) | Korinthia (el) | Mycènes | 37,739178 | 22,738847 | 26/05/2002 | Coeur d'Acier | Verbenaceae | Vitex agnus-castus L. |
| ACOE1368 | Greece | Peloponnisos (el) | Korinthia (el) | Mycènes | 37,739178 | 22,738847 | 26/05/2002 | Coeur d'Acier | Asteraceae | Sonchus oleraceus L. |
| ACOE1369 | Greece | Peloponnisos (el) | Korinthia (el) | Mycènes | 37,739178 | 22,738847 | 26/05/2002 | Coeur d'Acier | Polygonaceae | Rumex sp. L. |
| ACOE1370 | Greece | Peloponnisos (el) | Korinthia (el) | Mycènes | 37,739178 | 22,738847 | 26/05/2002 | Coeur d'Acier |  |  |
| ACOE1371 | Greece | Peloponnisos (el) | Korinthia (el) | Mycènes | 37,739178 | 22,738847 | 26/05/2002 | Coeur d'Acier | Gramineae | Avena sp. L. |
| ACOE1374 | Greece | Peloponnisos (el) | Korinthia (el) | Mycènes | 37,739178 | 22,738847 | 26/05/2002 | Coeur d'Acier | Malvaceae | Malva sylvestris L. |
| ACOE1375 | Greece | Peloponnisos (el) | Korinthia (el) | Mycènes | 37,739178 | 22,738847 | 26/05/2002 | Coeur d'Acier | Apiaceae | Apiaceae sp. Lindl. |
| ACOE1379 | Greece | Peloponnisos (el) | Korinthia (el) | Némea | 37,739022 | 22,735965 | 26/05/2002 | Coeur d'Acier | Cruciferae | Sisymbrium officinale (L.) SCOP. |
| ACOE1381 | Greece | Peloponnisos (el) | Korinthia (el) | Némea | 37,739022 | 22,735965 | 26/05/2002 | Coeur d'Acier | Euphorbiaceae | Euphorbia helioscopia L. |
| ACOE1382 | Greece | Peloponnisos (el) | Korinthia (el) | Némea | 37,739022 | 22,735965 | 26/05/2002 | Coeur d'Acier |  |  |
| ACOE1383 | Greece | Peloponnisos (el) | Korinthia (el) | Némea | 37,739022 | 22,735965 | 26/05/2002 | Coeur d'Acier | Compositae | Chondrilla ramosissima SIBTH. & SM. |
| ACOE1384 | Greece | Peloponnisos (el) | Korinthia (el) | Némea | 37,739022 | 22,735965 | 26/05/2002 | Coeur d'Acier | Asteraceae | Asteraceae sp. |
| ACOE1388 | Greece | Peloponnisos (el) | Korinthia (el) | Némea | 37,739022 | 22,735965 | 26/05/2002 | Coeur d'Acier | Asteraceae | Lactuca serriola L. |
| ACOE1390 | Greece | Peloponnisos (el) | Korinthia (el) | Kaliani | 37,859722 | 22,454722 | 26/05/2002 | Coeur d'Acier | Asteraceae | Carduus sp. L. |
| ACOE1391 | Greece | Peloponnisos (el) | Korinthia (el) | Kaliani | 37,859722 | 22,454722 | 26/05/2002 | Coeur d'Acier | Asteraceae | Asteraceae sp. |
| ACOE1393 | Greece | Peloponnisos (el) | Argolida (el) | Nauplie | 37,58624 | 22,789394 | 27/05/2002 | Coeur d'Acier | Tamaricaceae | Tamarix sp. L. |
| ACOE1394 | Greece | Peloponnisos (el) | Argolida (el) | Nauplie | 37,58624 | 22,789394 | 27/05/2002 | Coeur d'Acier | Poaceae | Arundo donax L. |
| ACOE1396 | Greece | Peloponnisos (el) | Argolida (el) | Nauplie | 37,58624 | 22,789394 | 27/05/2002 | Coeur d'Acier | Asteraceae | Sonchus sp. L. |
| ACOE1399 | Greece | Peloponnisos (el) | Argolida (el) | Kivéri | 37,526567 | 22,730998 | 27/05/2002 | Coeur d'Acier | Leguminosae | Melilotus indica (L.) ALL. |
| ACOE1401 | Greece | Peloponnisos (el) | Argolida (el) | Kivéri | 37,526567 | 22,730998 | 27/05/2002 | Coeur d'Acier | Apiaceae | Apiaceae sp. Lindl. |
| ACOE1403 | Greece | Peloponnisos (el) | Argolida (el) | Kivéri | 37,526567 | 22,730998 | 27/05/2002 | Coeur d'Acier | Apiaceae | Foeniculum vulgare Miller |
| ACOE1405 | Greece | Peloponnisos (el) | Argolida (el) | Mili | 37,552862 | 22,71991 | 27/05/2002 | Coeur d'Acier | Salicaceae | Salix sp. L. |
| ACOE1407 | Greece | Peloponnisos (el) | Argolida (el) | Mili | 37,552862 | 22,71991 | 27/05/2002 | Coeur d'Acier | Rosaceae | Rubus sp. L. |
| ACOE1408 | Greece | Peloponnisos (el) | Argolida (el) | Mili | 37,552862 | 22,71991 | 27/05/2002 | Coeur d'Acier | Myrtaceae | Eucalyptus globulus LABILL. |
| ACOE1410 | Greece | Peloponnisos (el) | Argolida (el) | Nauplie | 37,562013 | 22,802966 | 27/05/2002 | Coeur d'Acier | Fabaceae | Medicago arborea L. |
| ACOE1411 | Greece | Peloponnisos (el) | Argolida (el) | Nauplie | 37,562013 | 22,802966 | 27/05/2002 | Coeur d'Acier | Labiatae | Lamium sp. L. |
| ACOE1421 | Greece | Peloponnisos (el) | Korinthia (el) | Mycènes | 37,719002 | 22,740848 | 28/05/2002 | Coeur d'Acier | Asteraceae | Sonchus sp. L. |
| ACOE1422 | Greece | Peloponnisos (el) | Korinthia (el) | Mycènes | 37,719002 | 22,740848 | 28/05/2002 | Coeur d'Acier | Asteraceae | Matricaria recutita L. |
| ACOE1424 | Greece | Peloponnisos (el) | Korinthia (el) | Mycènes | 37,719002 | 22,740848 | 28/05/2002 | Coeur d'Acier | Malvaceae | Malva sp. L. |
| ACOE1426 | Greece | Peloponnisos (el) | Korinthia (el) | Mycènes | 37,719002 | 22,740848 | 28/05/2002 | Coeur d'Acier | Amaranthaceae | Amaranthus lividus L. |
| ACOE1427 | Greece | Peloponnisos (el) | Korinthia (el) | Mycènes | 37,719002 | 22,740848 | 28/05/2002 | Coeur d'Acier | Apiaceae | Apiaceae sp. Lindl. |
| ACOE1429 | Greece | Peloponnisos (el) | Korinthia (el) | Mycènes | 37,719002 | 22,740848 | 28/05/2002 | Coeur d'Acier | Asteraceae | Sonchus sp. L. |
| ACOE1430 | Greece | Peloponnisos (el) | Korinthia (el) | Mycènes | 37,719002 | 22,740848 | 28/05/2002 | Coeur d'Acier | Rosaceae | Prunus armeniaca L. |
| ACOE1435 | Greece | Peloponnisos (el) | Korinthia (el) | Mycènes | 37,719002 | 22,740848 | 28/05/2002 | Coeur d'Acier | Araliaceae | Hedera helix L. |
| ACOE1436 | Greece | Peloponnisos (el) | Korinthia (el) | Mycènes | 37,719002 | 22,740848 | 28/05/2002 | Coeur d'Acier | Rosaceae | Rosa sp. L. |
| ACOE1437 | Greece | Peloponnisos (el) | Korinthia (el) | Mycènes | 37,727707 | 22,749027 | 28/05/2002 | Coeur d'Acier | Apiaceae | Apiaceae sp. Lindl. |
| ACOE1439 | Greece | Peloponnisos (el) | Korinthia (el) | Mycènes | 37,727707 | 22,749027 | 28/05/2002 | Coeur d'Acier | Portulacaceae | Portulaca oleracea L. |
| ACOE1440 | Greece | Peloponnisos (el) | Korinthia (el) | Mycènes | 37,727707 | 22,749027 | 28/05/2002 | Coeur d'Acier | Zygophyllaceae | Tribulus terrestris L. |
| ACOE1441 | Greece | Peloponnisos (el) | Korinthia (el) | Mycènes | 37,727707 | 22,749027 | 28/05/2002 | Coeur d'Acier | Chenopodiaceae | Chenopodiaceae sp. Vent. |
| ACOE1442 | Greece | Peloponnisos (el) | Korinthia (el) | Mycènes | 37,727707 | 22,749027 | 28/05/2002 | Coeur d'Acier |  |  |
| ACOE1443 | Greece | Peloponnisos (el) | Korinthia (el) | Mycènes | 37,727707 | 22,749027 | 28/05/2002 | Coeur d'Acier |  |  |
| ACOE1444 | Greece | Peloponnisos (el) | Korinthia (el) | Mycènes | 37,727707 | 22,749027 | 28/05/2002 | Coeur d'Acier | Rosaceae | Rubus sp. L. |
| ACOE1445 | Greece | Peloponnisos (el) | Korinthia (el) | Mycènes | 37,727707 | 22,749027 | 28/05/2002 | Coeur d'Acier | Rutaceae | Citrus sp. L. |
| ACOE1446 | Greece | Peloponnisos (el) | Lakonia (el) | Kariés | 37,359489 | 22,440402 | 29/05/2002 | Coeur d'Acier | Rosaceae | Prunus sp. L. |
| ACOE1447 | Greece | Peloponnisos (el) | Lakonia (el) | Kariés | 37,359489 | 22,440402 | 29/05/2002 | Coeur d'Acier | Rosaceae | Rosa sp. L. |
| ACOE1448 | Greece | Peloponnisos (el) | Lakonia (el) | Kariés | 37,359489 | 22,440402 | 29/05/2002 | Coeur d'Acier | Rosaceae | Pyrus communis L. |
| ACOE1449 | Greece | Peloponnisos (el) | Lakonia (el) | Kariés | 37,359489 | 22,440402 | 29/05/2002 | Coeur d'Acier | Euphorbiaceae | Euphorbia characias L. |
| ACOE1450 | Greece | Peloponnisos (el) | Lakonia (el) | Kariés | 37,359489 | 22,440402 | 29/05/2002 | Coeur d'Acier | Polygonaceae | Rumex sp. L. |
| ACOE1451 | Greece | Peloponnisos (el) | Lakonia (el) | Kariés | 37,359489 | 22,440402 | 29/05/2002 | Coeur d'Acier | Fabaceae | Vicia cracca L. |
| ACOE1452 | Greece | Peloponnisos (el) | Lakonia (el) | Kariés | 37,359489 | 22,440402 | 29/05/2002 | Coeur d'Acier | Fagaceae | Quercus sp. L. |
| ACOE1453 | Greece | Peloponnisos (el) | Lakonia (el) | Kariés | 37,359489 | 22,440402 | 29/05/2002 | Coeur d'Acier | Compositae | Tragopogon crocifolius L. |
| ACOE1454 | Greece | Peloponnisos (el) | Lakonia (el) | Kariés | 37,359489 | 22,440402 | 29/05/2002 | Coeur d'Acier | Asteraceae | Carduus pycnocephalus L. |
| ACOE1455 | Greece | Peloponnisos (el) | Lakonia (el) | Kariés | 37,359489 | 22,440402 | 29/05/2002 | Coeur d'Acier | Asteraceae | Scorzonera sp. L. |
| ACOE1456 | Greece | Peloponnisos (el) | Lakonia (el) | Lagada | 37,077761 | 22,288898 | 29/05/2002 | Coeur d'Acier |  |  |
| ACOE1457 | Greece | Peloponnisos (el) | Lakonia (el) | Lagada | 37,077761 | 22,288898 | 29/05/2002 | Coeur d'Acier | Ranunculaceae | Clematis vitalba L. |
| ACOE1458 | Greece | Peloponnisos (el) | Lakonia (el) | Lagada | 37,077761 | 22,288898 | 29/05/2002 | Coeur d'Acier |  |  |
| ACOE1461 | Greece | Peloponnisos (el) | Lakonia (el) | Lagada | 37,077761 | 22,288898 | 29/05/2002 | Coeur d'Acier | Rosaceae | Rosaceae sp. L. |
| ACOE1463 | Greece | Peloponnisos (el) | Lakonia (el) | Lagada | 37,077761 | 22,288898 | 29/05/2002 | Coeur d'Acier | Umbelliferae | Orlaya sp. HOFFM. |
| ACOE1464 | Greece | Peloponnisos (el) | Lakonia (el) | Lagada | 37,086584 | 22,282577 | 29/05/2002 | Coeur d'Acier | Cistaceae | Cistus parviflorus LAM. |
| ACOE1467 | Greece | Peloponnisos (el) | Lakonia (el) | Lagada | 37,086584 | 22,282577 | 29/05/2002 | Coeur d'Acier | Labiaceae | Labiaceae sp. Dulac |
| ACOE1468 | Greece | Peloponnisos (el) | Lakonia (el) | Mystra | 37,070231 | 22,381719 | 30/05/2002 | Coeur d'Acier | Umbelliferae | Ferula communis L. |
| ACOE1470 | Greece | Peloponnisos (el) | Lakonia (el) | Mystra | 37,070231 | 22,381719 | 30/05/2002 | Coeur d'Acier |  |  |
| ACOE1471 | Greece | Peloponnisos (el) | Lakonia (el) | Mystra | 37,070231 | 22,381719 | 30/05/2002 | Coeur d'Acier | Ulmaceae | Celtis australis L. |
| ACOE1472 | Greece | Peloponnisos (el) | Lakonia (el) | Mystra | 37,070231 | 22,381719 | 30/05/2002 | Coeur d'Acier | Juglandaceae | Juglans regia L. |
| ACOE1473 | Greece | Peloponnisos (el) | Lakonia (el) | Mystra | 37,070231 | 22,381719 | 30/05/2002 | Coeur d'Acier | Malvaceae | Malva sylvestris L. |
| ACOE1474 | Greece | Peloponnisos (el) | Lakonia (el) | Mystra | 37,070231 | 22,381719 | 30/05/2002 | Coeur d'Acier | Caryophyllaceae | Silene vulgaris (Moench.) Garcke |
| ACOE1475 | Greece | Peloponnisos (el) | Lakonia (el) | Mystra | 37,070231 | 22,381719 | 30/05/2002 | Coeur d'Acier | Euphorbiaceae | Euphorbia characias L. |
| ACOE1478 | Greece | Peloponnisos (el) | Lakonia (el) | Mystra | 37,070231 | 22,381719 | 30/05/2002 | Coeur d'Acier | Fagaceae | Quercus coccifera L. |
| ACOE1479 | Greece | Peloponnisos (el) | Lakonia (el) | Mystra | 37,070231 | 22,381719 | 30/05/2002 | Coeur d'Acier | Aceraceae | Acer sempervirens L. |
| ACOE1480 | Greece | Peloponnisos (el) | Lakonia (el) | Mystra | 37,070231 | 22,381719 | 30/05/2002 | Coeur d'Acier | Leguminosae | Coronilla emerus (BOISS. & SPRUNER) HAYEK |
| ACOE1481 | Greece | Peloponnisos (el) | Lakonia (el) | Mystra | 37,070231 | 22,381719 | 30/05/2002 | Coeur d'Acier | Labiaceae | Labiaceae sp. Dulac |
| ACOE1482 | Greece | Peloponnisos (el) | Lakonia (el) | Mystra | 37,070231 | 22,381719 | 30/05/2002 | Coeur d'Acier | Punicaceae | Punica granatum L. |
| ACOE1484 | Greece | Peloponnisos (el) | Lakonia (el) | Mystra | 37,070231 | 22,381719 | 30/05/2002 | Coeur d'Acier |  |  |
| ACOE1485 | Greece | Peloponnisos (el) | Lakonia (el) | Mystra | 37,070231 | 22,381719 | 30/05/2002 | Coeur d'Acier | Hypericaceae | Hypericum sp. L. |
| ACOE1487 | Greece | Peloponnisos (el) | Lakonia (el) | Mystra | 37,070231 | 22,381719 | 30/05/2002 | Coeur d'Acier | Poaceae | Poaceae sp. Barnhart |
| ACOE1489 | Greece | Peloponnisos (el) | Lakonia (el) | Skoura | 37,013118 | 22,479884 | 31/05/2002 | Coeur d'Acier | Salicaceae | Salix sp. L. |
| ACOE1490 | Greece | Peloponnisos (el) | Lakonia (el) | Skoura | 37,013118 | 22,479884 | 31/05/2002 | Coeur d'Acier | Gramineae | Saccharum ravennae (L.) MURRAY |
| ACOE1493 | Greece | Peloponnisos (el) | Lakonia (el) | Kastoria | 37,164921 | 22,309185 | 31/05/2002 | Coeur d'Acier | Salicaceae | Populus nigra L. |
| ACOE1494 | Greece | Peloponnisos (el) | Lakonia (el) | Kastoria | 37,164921 | 22,309185 | 31/05/2002 | Coeur d'Acier | Rosaceae | Rubus sp. L. |
| ACOE1495 | Greece | Peloponnisos (el) | Lakonia (el) | Kastoria | 37,164921 | 22,309185 | 31/05/2002 | Coeur d'Acier | Verbenaceae | Vitex agnus-castus L. |
| ACOE1496 | Greece | Peloponnisos (el) | Lakonia (el) | Kastoria | 37,164921 | 22,309185 | 31/05/2002 | Coeur d'Acier | Rubiaceae | Galium aparine L. |
| ACOE1500 | Greece | Peloponnisos (el) | Lakonia (el) | Mystra | 37,070231 | 22,381719 | 30/05/2002 | Coeur d'Acier | Campanulaceae | Campanula sp. L. |
| ACOE1503 | Greece | Peloponnisos (el) | Lakonia (el) | Mystra | 27,078646 | 22,357121 | 31/05/2002 | Coeur d'Acier | Fabaceae | Vicia sp. L. |
| ACOE1504 | Greece | Peloponnisos (el) | Lakonia (el) | Mystra | 27,078646 | 22,357121 | 31/05/2002 | Coeur d'Acier | Fagaceae | Castanea sativa Miller |
| ACOE1505 | Greece | Peloponnisos (el) | Lakonia (el) | Mystra | 27,078646 | 22,357121 | 31/05/2002 | Coeur d'Acier | Leguminosae | Calicotome villosa (POIRET) LINK |
| ACOE1507 | Greece | Peloponnisos (el) | Lakonia (el) | Mystra | 37,098889 | 22,353611 | 31/05/2002 | Coeur d'Acier | Ranunculaceae | Clematis sp. L. |
| ACOE1508 | Greece | Peloponnisos (el) | Lakonia (el) | Mystra | 37,069951 | 22,381838 | 31/05/2002 | Coeur d'Acier | Salicaceae | Populus alba L. |
| ACOE1509 | Greece | Peloponnisos (el) | Lakonia (el) | Mystra | 37,069951 | 22,381838 | 31/05/2002 | Coeur d'Acier | Fabaceae | Glycine max Merrill. |
| ACOE1510 | Greece | Peloponnisos (el) | Lakonia (el) | Kelefa | 36,727003 | 22,527087 | 31/05/2002 | Coeur d'Acier | Salicaceae | Populus alba L. |
| ACOE1511 | Greece | Peloponnisos (el) | Lakonia (el) | Kelefa | 36,727003 | 22,527087 | 31/05/2002 | Coeur d'Acier | Labiaceae | Labiaceae sp. Dulac |
| ACOE1512 | Greece | Peloponnisos (el) | Lakonia (el) | Kelefa | 36,727003 | 22,527087 | 31/05/2002 | Coeur d'Acier | Fagaceae | Quercus macrolepis KOTSCHY |
| ACOE1513 | Greece | Peloponnisos (el) | Lakonia (el) | Kelefa | 36,727003 | 22,527087 | 31/05/2002 | Coeur d'Acier | Labiatae | Origanum sp. L. |
| ACOE1514 | Greece | Peloponnisos (el) | Lakonia (el) | Kelefa | 36,727003 | 22,527087 | 31/05/2002 | Coeur d'Acier | Malvaceae | Malvaceae sp. |
| ACOE1515 | Greece | Peloponnisos (el) | Lakonia (el) | Kelefa | 36,727003 | 22,527087 | 31/05/2002 | Coeur d'Acier | Fabaceae | Fabaceae sp. Lindl. |
| ACOE1516 | Greece | Peloponnisos (el) | Lakonia (el) | Pirgos Dirou | 36,62549 | 22,381763 | 01/06/2002 | Coeur d'Acier | Asteraceae | Asteraceae sp. |
| ACOE1519 | Greece | Peloponnisos (el) | Lakonia (el) | Porto kagio | 36,429422 | 22,487495 | 01/06/2002 | Coeur d'Acier | Plumbaginaceae | Limonium sp. |
| ACOE1521 | Greece | Peloponnisos (el) | Lakonia (el) | Porto kagio | 36,429422 | 22,487495 | 01/06/2002 | Coeur d'Acier | Asteraceae | Asteraceae sp. |
| ACOE1522 | Greece | Peloponnisos (el) | Lakonia (el) | Gythion | 36,727939 | 22,540677 | 02/06/2002 | Coeur d'Acier | Zygophyllaceae | Tribulus terrestris L. |
| ACOE1524 | Greece | Peloponnisos (el) | Lakonia (el) | Gythion | 36,727939 | 22,540677 | 02/06/2002 | Coeur d'Acier | Salicaceae | Populus sp. L. |
| ACOE1525 | Greece | Peloponnisos (el) | Lakonia (el) | Gythion | 36,727939 | 22,540677 | 02/06/2002 | Coeur d'Acier | Asclepiadaceae | Cionura erecta (L.) GRISEB. |
| ACOE1526 | Greece | Peloponnisos (el) | Lakonia (el) | Gythion | 36,727939 | 22,540677 | 02/06/2002 | Coeur d'Acier | Asteraceae | Cichorium sp. L. |
| ACOE1527 | Greece | Peloponnisos (el) | Lakonia (el) | Skala | 36,846988 | 22,656782 | 02/06/2002 | Coeur d'Acier | Fabaceae | Fabaceae sp. Lindl. |
| ACOE1537 | Greece | Peloponnisos (el) | Lakonia (el) | Monemvassia | 36,68848 | 23,054965 | 02/06/2002 | Coeur d'Acier | Pittosporaceae | Pittosporum tobira (Thunb.) Aiton fil. |
| ACOE1539 | Greece | Peloponnisos (el) | Lakonia (el) | Monemvassia | 36,68848 | 23,054965 | 02/06/2002 | Coeur d'Acier | Papaveraceae | Glaucium flavum Crantz |
| ACOE1540 | Greece | Peloponnisos (el) | Lakonia (el) | Monemvassia | 36,68848 | 23,054965 | 02/06/2002 | Coeur d'Acier | Brassicaceae | Matthiola sp. R. Brown |
| ACOE1542 | Greece | Dytiki Ellada (el) | Ileia (el) | Kaiafas | 37,512195 | 21,597219 | 03/06/2002 | Coeur d'Acier | Pinaceae | Pinus sp. L. |
| ACOE1544 | Greece | Dytiki Ellada (el) | Ileia (el) | Kaiafas | 37,512195 | 21,597219 | 03/06/2002 | Coeur d'Acier | Leguminosae | Dorycnium sp. |
| ACOE1545 | Greece | Dytiki Ellada (el) | Ileia (el) | Kaiafas | 37,512195 | 21,597219 | 03/06/2002 | Coeur d'Acier | Dipsacaceae | Scabiosa sp. L. |
| ACOE1546 | Greece | Dytiki Ellada (el) | Ileia (el) | Olympia | 37,643017 | 21,619943 | 03/05/2002 | Coeur d'Acier | Salicaceae | Populus nigra L. |
| ACOE1547 | Greece | Dytiki Ellada (el) | Ileia (el) | Olympia | 37,643017 | 21,619943 | 03/05/2002 | Coeur d'Acier | Salicaceae | Populus nigra L. |
| ACOE1549 | Greece | Dytiki Ellada (el) | Ileia (el) | Olympia | 37,643017 | 21,619943 | 03/05/2002 | Coeur d'Acier | Asteraceae | Asteraceae sp. |
| ACOE1550 | Greece | Dytiki Ellada (el) | Ileia (el) | Olympia | 37,643017 | 21,619943 | 03/05/2002 | Coeur d'Acier | Asteraceae | Asteraceae sp. |
| ACOE1551 | Greece | Dytiki Ellada (el) | Ileia (el) | Olympia | 37,643017 | 21,619943 | 03/05/2002 | Coeur d'Acier | Fabaceae | Lathyrus sp. L. |
| ACOE1552 | Greece | Dytiki Ellada (el) | Ileia (el) | Olympia | 37,643017 | 21,619943 | 03/05/2002 | Coeur d'Acier | Rosaceae | Rosaceae sp. L. |
| ACOE1553 | Greece | Dytiki Ellada (el) | Ileia (el) | Olympia | 37,643017 | 21,619943 | 03/05/2002 | Coeur d'Acier | Apiaceae | Apiaceae sp. Lindl. |
| ACOE1554 | Greece | Dytiki Ellada (el) | Ileia (el) | Olympia | 37,643017 | 21,619943 | 03/05/2002 | Coeur d'Acier | Fagaceae | Quercus ilex L. |
| ACOE1558 | Greece | Dytiki Ellada (el) | Ileia (el) | Lala | 37,743844 | 31,761898 | 04/06/2002 | Coeur d'Acier | Apiaceae | Torilis sp. Adanson |
| ACOE1560 | Greece | Dytiki Ellada (el) | Ileia (el) | Lala | 37,743844 | 31,761898 | 04/06/2002 | Coeur d'Acier | Rosaceae | Rubus sp. L. |
| ACOE1561 | Greece | Dytiki Ellada (el) | Ileia (el) | Lala | 37,743844 | 31,761898 | 04/06/2002 | Coeur d'Acier | Poaceae | Poaceae sp. Barnhart |
| ACOE1562 | Greece | Dytiki Ellada (el) | Ileia (el) | Lala | 37,743844 | 31,761898 | 04/06/2002 | Coeur d'Acier |  |  |
| ACOE1563 | Greece | Dytiki Ellada (el) | Ileia (el) | Lala | 37,743844 | 31,761898 | 04/06/2002 | Coeur d'Acier |  |  |
| ACOE1565 | Greece | Dytiki Ellada (el) | Ileia (el) | Lala | 37,80991 | 21,78609 | 04/06/2002 | Coeur d'Acier | Salicaceae | Salix sp. L. |
| ACOE1566 | Greece | Dytiki Ellada (el) | Achaïa (el) | Tripotama | 37,926656 | 21,926656 | 04/06/2002 | Coeur d'Acier | Salicaceae | Salix sp. L. |
| ACOE1567 | Greece | Dytiki Ellada (el) | Achaïa (el) | Tripotama | 37,926656 | 21,926656 | 04/06/2002 | Coeur d'Acier | Salicaceae | Salix sp. L. |
| ACOE1568 | Greece | Dytiki Ellada (el) | Achaïa (el) | Tripotama | 37,926656 | 21,926656 | 04/06/2002 | Coeur d'Acier | Urticaceae | Urtica sp. L. |
| ACOE1569 | Greece | Dytiki Ellada (el) | Achaïa (el) | Tripotama | 37,926656 | 21,926656 | 04/06/2002 | Coeur d'Acier | Labiaceae | Labiaceae sp. Dulac |
| ACOE1570 | Greece | Dytiki Ellada (el) | Achaïa (el) | Tripotama | 37,926656 | 21,926656 | 04/06/2002 | Coeur d'Acier | Polygonaceae | Polygonaceae sp. Juss. |
| ACOE1572 | Greece | Dytiki Ellada (el) | Achaïa (el) | Léchouri | 37,922068 | 21,912573 | 04/06/2002 | Coeur d'Acier | Urticaceae | Urtica sp. L. |
| ACOE1575 | Greece | Dytiki Ellada (el) | Achaïa (el) | Léchouri | 37,922068 | 21,912573 | 04/06/2002 | Coeur d'Acier | Caprifoliaceae | Sambucus nigra L. |
| ACOE1576 | Greece | Dytiki Ellada (el) | Ileia (el) | Mouria | 37,645499 | 21,731893 | 04/06/2002 | Coeur d'Acier | Fagaceae | Quercus ilex L. |
| ACOE1578 | Greece | Peloponnisos (el) | Arkadia (el) | Toumbitsi | 37,63318 | 21,791953 | 05/06/2002 | Coeur d'Acier | Verbenaceae | Verbena officinalis L. |
| ACOE1580 | Greece | Peloponnisos (el) | Arkadia (el) | Toumbitsi | 37,63318 | 21,791953 | 05/06/2002 | Coeur d'Acier | Asteraceae | Picris sp. L. |
| ACOE1585 | Greece | Peloponnisos (el) | Arkadia (el) | Lagadia | 37,681864 | 22,029981 | 05/06/2002 | Coeur d'Acier | Urticaceae | Parietaria sp. L. |
| ACOE1586 | Greece | Peloponnisos (el) | Arkadia (el) | Lagadia | 37,691944 | 22,038611 | 05/06/2002 | Coeur d'Acier |  |  |
| ACOE1587 | Greece | Peloponnisos (el) | Arkadia (el) | Lagadia | 37,691944 | 22,038611 | 05/06/2002 | Coeur d'Acier | Papaveraceae | Fumaria sp. L. |
| ACOE1588 | Greece | Peloponnisos (el) | Arkadia (el) | Lagadia | 37,691944 | 22,038611 | 05/06/2002 | Coeur d'Acier | Labiaceae | Labiaceae sp. Dulac |
| ACOE1590 | Greece | Peloponnisos (el) | Arkadia (el) | Lagadia | 37,691944 | 22,038611 | 05/06/2002 | Coeur d'Acier | Umbelliferae | Malabaila aurea (SIBTH. & SM.) BOISS. |
| ACOE1591 | Greece | Peloponnisos (el) | Arkadia (el) | Lagadia | 37,691944 | 22,038611 | 05/06/2002 | Coeur d'Acier | Iridaceae | Iris sp. L. |
| ACOE1592 | Greece | Peloponnisos (el) | Arkadia (el) | Lagadia | 37,691944 | 22,038611 | 05/06/2002 | Coeur d'Acier | Caprifoliaceae | Sambucus ebulus L. |
| ACOE1593 | Greece | Peloponnisos (el) | Arkadia (el) | Lagadia | 37,691944 | 22,038611 | 05/06/2002 | Coeur d'Acier | Polygonaceae | Rumex pulcher L. |
| ACOE1595 | Greece | Dytiki Ellada (el) | Achaïa (el) | Kalavrita | 38,0333 | 22,1167 | 06/06/2002 | Coeur d'Acier | Asteraceae | Asteraceae sp. |
| ACOE1599 | Greece | Dytiki Ellada (el) | Achaïa (el) | Kalavrita | 38,0333 | 22,1167 | 06/06/2002 | Coeur d'Acier | Asteraceae | Asteraceae sp. |
| ACOE1601 | Greece | Dytiki Ellada (el) | Achaïa (el) | Kalavrita | 38,0333 | 22,1167 | 06/06/2002 | Coeur d'Acier | Labiaceae | Labiaceae sp. Dulac |
| ACOE1602 | Greece | Dytiki Ellada (el) | Achaïa (el) | Kalavrita | 38,0333 | 22,1167 | 06/06/2002 | Coeur d'Acier |  |  |
| ACOE1605 | Greece | Dytiki Ellada (el) | Achaïa (el) | Kalavrita | 38,0333 | 22,1167 | 06/06/2002 | Coeur d'Acier | Fabaceae | Lathyrus sp. L. |
| ACOE1606 | Greece | Dytiki Ellada (el) | Achaïa (el) | Kalavrita | 38,0333 | 22,1167 | 06/06/2002 | Coeur d'Acier | Ranunculaceae | Ranunculus sp. L. |
| ACOE1607 | Greece | Dytiki Ellada (el) | Achaïa (el) | Kalavrita | 38,0333 | 22,1167 | 06/06/2002 | Coeur d'Acier | Labiaceae | Labiaceae sp. Dulac |
| ACOE1609 | Greece | Dytiki Ellada (el) | Achaïa (el) | Kalavrita | 38,0333 | 22,1167 | 06/06/2002 | Coeur d'Acier | Valerianaceae | Valeriana sp. L. |
| ACOE1610 | France | Rhône-Alpes | Drôme | Rousset-les-Vignes | 44,843897 | 5,409135 | 05/07/2003 | Coeur d'Acier | Oenotheraceae | Epilobium sp. L. |
| ACOE1611 | France | Rhône-Alpes | Drôme | Rousset-les-Vignes | 44,843897 | 5,409135 | 05/07/2003 | Coeur d'Acier | Apiaceae | Apiaceae sp. Lindl. |
| ACOE1612 | France | Rhône-Alpes | Drôme | Rousset-les-Vignes | 44,843897 | 5,409135 | 05/07/2003 | Coeur d'Acier | Rubiaceae | Galium sp. L. |
| ACOE1613 | France | Rhône-Alpes | Drôme | Rousset-les-Vignes | 44,843897 | 5,409135 | 05/07/2003 | Coeur d'Acier | Apiaceae | Apiaceae sp. Lindl. |
| ACOE1616 | France | Rhône-Alpes | Drôme | Vercheny | 44,695279 | 5,241437 | 05/07/2003 | Coeur d'Acier | Leguminosae | Melilotus sp. P. Miller |
| ACOE1618 | France | Rhône-Alpes | Drôme | Rousset-les-Vignes | 44,832675 | 5,397087 | 06/07/2003 | Coeur d'Acier | Poaceae | Poaceae sp. Barnhart |
| ACOE1619 | France | Rhône-Alpes | Drôme | Rousset-les-Vignes | 44,832675 | 5,397087 | 06/07/2003 | Coeur d'Acier | Dipsacaceae | Dipsacaceae sp. Juss. |
| ACOE1620 | France | Rhône-Alpes | Drôme | Rousset-les-Vignes | 44,832675 | 5,397087 | 06/07/2003 | Coeur d'Acier | Fabaceae | Trifolium sp. L. |
| ACOE1621 | France | Rhône-Alpes | Drôme | Vassieux-en-Vercors | 44,894699 | 5,367177 | 06/07/2003 | Coeur d'Acier | Asteraceae | Chardon sp. |
| ACOE1623 | France | Rhône-Alpes | Drôme | Vassieux-en-Vercors | 44,894699 | 5,367177 | 06/07/2003 | Coeur d'Acier | Tiliaceae | Tilia sp. L. |
| ACOE1624 | France | Rhône-Alpes | Drôme | La Chapelle-en-Vercors | 45,001927 | 5,421836 | 06/07/2003 | Coeur d'Acier | Rosaceae | Agrimonia eupatoria L. |
| ACOE1625 | France | Rhône-Alpes | Drôme | La Chapelle-en-Vercors | 45,083262 | 5,479715 | 06/07/2003 | Coeur d'Acier | Polygonaceae | Rumex sp. L. |
| ACOE1626 | France | Rhône-Alpes | Drôme | La Chapelle-en-Vercors | 45,083262 | 5,479715 | 06/07/2003 | Coeur d'Acier | Oenotheraceae | Epilobium sp. L. |
| ACOE1628 | France | Rhône-Alpes | Drôme | Rousset-les-Vignes | 44,851944 | 5,424722 | 07/07/2003 | Coeur d'Acier | Asteraceae | Asteraceae sp. |
| ACOE1629 | France | Rhône-Alpes | Drôme | Saint-Agnan-en-Vercors | 44,955954 | 5,438791 | 07/07/2003 | Coeur d'Acier | Rosaceae | Crataegus sp. L. |
| ACOE1630 | France | Rhône-Alpes | Drôme | Saint-Agnan-en-Vercors | 44,955954 | 5,438791 | 07/07/2003 | Coeur d'Acier | Salicaceae | Salix sp. L. |
| ACOE1632 | France | Rhône-Alpes | Drôme | La Chapelle-en-Vercors | 44,965801 | 5,413978 | 07/07/2003 | Coeur d'Acier | Plantaginaceae | Plantago sp. L. |
| ACOE1634 | France | Rhône-Alpes | Drôme | La Chapelle-en-Vercors | 44,965801 | 5,413978 | 07/07/2003 | Coeur d'Acier | Asteraceae | Arctium sp. L. |
| ACOE1635 | France | Rhône-Alpes | Drôme | La Chapelle-en-Vercors | 44,965801 | 5,413978 | 07/07/2003 | Coeur d'Acier | Oenotheraceae | Epilobium sp. L. |
| ACOE1636 | France | Rhône-Alpes | Drôme | La Chapelle-en-Vercors | 44,965801 | 5,413978 | 07/07/2003 | Coeur d'Acier | Oenotheraceae | Oenothera biennis L. |
| ACOE1637 | France | Rhône-Alpes | Drôme | La Chapelle-en-Vercors | 44,965801 | 5,413978 | 07/07/2003 | Coeur d'Acier | Rosaceae | Malus sp. P. Miller |
| ACOE1639 | France | Rhône-Alpes | Drôme | La Chapelle-en-Vercors | 44,965801 | 5,413978 | 07/07/2003 | Coeur d'Acier | Polygonaceae | Polygonaceae sp. Juss. |
| ACOE1641 | France | Rhône-Alpes | Drôme | Saint-Agnan-en-Vercors | 44,972778 | 5,440833 | 08/07/2003 | Coeur d'Acier | Asteraceae | Cirsium sp. P. Miller |
| ACOE1642 | France | Rhône-Alpes | Drôme | Saint-Agnan-en-Vercors | 44,972778 | 5,440833 | 08/07/2003 | Coeur d'Acier | Polygonaceae | Fallopia convolvulus (L.) 'A. Löve |
| ACOE1643 | France | Rhône-Alpes | Drôme | Saint-Agnan-en-Vercors | 44,972778 | 5,440833 | 08/07/2003 | Coeur d'Acier | Asteraceae | Sonchus sp. L. |
| ACOE1644 | France | Rhône-Alpes | Drôme | Saint-Agnan-en-Vercors | 44,972778 | 5,440833 | 08/07/2003 | Coeur d'Acier | Asteraceae | Sonchus sp. L. |
| ACOE1646 | France | Rhône-Alpes | Drôme | Saint-Martin-en-Vercors | 45,021599 | 5,442778 | 08/07/2003 | Coeur d'Acier | Araliaceae | Hedera helix L. |
| ACOE1647 | France | Rhône-Alpes | Drôme | Saint-Martin-en-Vercors | 45,021599 | 5,442778 | 08/07/2003 | Coeur d'Acier | Iridaceae | Iris sp. L. |
| ACOE1648 | France | Rhône-Alpes | Drôme | Saint-Martin-en-Vercors | 45,021599 | 5,442778 | 08/07/2003 | Coeur d'Acier | Oenotheraceae | Epilobium sp. L. |
| ACOE1650 | France | Rhône-Alpes | Drôme | Saint-Martin-en-Vercors | 45,032222 | 5,440556 | 08/07/2003 | Coeur d'Acier | Asteraceae | Sonchus sp. L. |
| ACOE1651 | France | Rhône-Alpes | Drôme | Saint-Martin-en-Vercors | 45,032222 | 5,440556 | 08/07/2003 | Coeur d'Acier | Asteraceae | Achillea sp. L. |
| ACOE1652 | France | Rhône-Alpes | Drôme | La Chapelle-en-Vercors | 44,994766 | 5,440599 | 08/07/2003 | Coeur d'Acier | Poaceae | Phragmites australis (Cav.) Steudel |
| ACOE1655 | France | Rhône-Alpes | Drôme | Saillans | 44,723354 | 5,2043 | 09/07/2003 | Coeur d'Acier | Apiaceae | Apiaceae sp. Lindl. |
| ACOE1656 | France | Rhône-Alpes | Drôme | Saillans | 44,723354 | 5,2043 | 09/07/2003 | Coeur d'Acier | Asteraceae | Sonchus sp. L. |
| ACOE1657 | France | Rhône-Alpes | Drôme | Saillans | 44,723354 | 5,2043 | 09/07/2003 | Coeur d'Acier | Plantaginaceae | Plantago major L. |
| ACOE1658 | France | Rhône-Alpes | Drôme | Saillans | 44,723354 | 5,2043 | 09/07/2003 | Coeur d'Acier | Verbenaceae | Verbena sp. L. |
| ACOE1659 | France | Rhône-Alpes | Drôme | Saillans | 44,723354 | 5,2043 | 09/07/2003 | Coeur d'Acier | Rosaceae | Malus sp. P. Miller |
| ACOE1660 | France | Rhône-Alpes | Drôme | Beaufort-sur-Gervanne | 44,776798 | 5,139478 | 09/07/2003 | Coeur d'Acier | Salicaceae | Populus sp. L. |
| ACOE1663 | France | Rhône-Alpes | Drôme | Ombleze | 44,864442 | 5,203031 | 09/07/2003 | Coeur d'Acier | Rosaceae | Crataegus sp. L. |
| ACOE1664 | France | Rhône-Alpes | Drôme | Ombleze | 44,864442 | 5,203031 | 09/07/2003 | Coeur d'Acier | Juglandaceae | Juglans regia L. |
| ACOE1665 | France | Rhône-Alpes | Drôme | Ombleze | 44,864442 | 5,203031 | 09/07/2003 | Coeur d'Acier |  |  |
| ACOE1666 | France | Rhône-Alpes | Drôme | Ombleze | 44,864442 | 5,203031 | 09/07/2003 | Coeur d'Acier | Apiaceae | Daucus carota L. |
| ACOE1668 | France | Rhône-Alpes | Drôme | Ombleze | 44,864442 | 5,203031 | 09/07/2003 | Coeur d'Acier | Fabaceae | Trifolium sp. L. |
| ACOE1669 | France | Rhône-Alpes | Drôme | Ombleze | 44,864442 | 5,203031 | 09/07/2003 | Coeur d'Acier | Fabaceae | Trifolium sp. L. |
| ACOE1670 | France | Rhône-Alpes | Drôme | Ombleze | 44,864442 | 5,203031 | 09/07/2003 | Coeur d'Acier | Apiaceae | Daucus sp. L. |
| ACOE1671 | France | Rhône-Alpes | Drôme | Ombleze | 44,864442 | 5,203031 | 09/07/2003 | Coeur d'Acier | Apiaceae | Daucus sp. L. |
| ACOE1673 | France | Rhône-Alpes | Drôme | Ombleze | 44,864442 | 5,203031 | 09/07/2003 | Coeur d'Acier | Rubiaceae | Galium sp. L. |
| ACOE1674 | France | Languedoc-Roussillon | Hérault | Teyran | 43,6833 | 3,926278 | 26/06/2003 | Coeur d'Acier | Rosaceae | Prunus mahaleb L. |
| ACOE1681 | France | Provence-Alpes-Côte- d'Azur | Bouches-du-Rhône | Saint-Martin-de-Crau | 43,598945 | 4,779848 | 04/04/2005 | Coeur.& Jous. | Asteraceae | Senecio sp. L. |
| ACOE1687 | France | Provence-Alpes-Côte- d'Azur | Var | Fayence | 43,56013 | 6,798099 | 06/04/2005 | Coeur.& Jous. | Rosaceae | Prunus dulcis (Miller) D.A. Webb |
| ACOE1693 | France | Provence-Alpes-Côte- d'Azur | Var | Fayence | 43,56013 | 6,798099 | 06/04/2005 | Coeur.& Jous. | Rosaceae | Prunus cerasus L. |
| ACOE1696 | France | Languedoc-Roussillon | Hérault | Rouet | 43,820397 | 3,813283 | 13/04/2005 | Coeur.& Jous. | Rosaceae | Prunus spinosa L. |
| ACOE1700 | France | Languedoc-Roussillon | Hérault | Saint-Guilhem-le-Desert | 43,7337 | 3,550078 | 13/04/2005 | Coeur.& Jous. | Rosaceae | Prunus cerasus L. |
| ACOE1704 | France | Languedoc-Roussillon | Hérault | Montferrier-sur-Lez | 43,665334 | 3,865982 | 22/04/2005 | Coeur.& Jous. | Caryophyllaceae | Silene sp. L. |
| ACOE1706 | France | Languedoc-Roussillon | Hérault | Montpellier | 43,64435 | 3,848899 | 22/04/2005 | Coeur.& Jous. | Rosaceae | Prunus dulcis (Miller) D.A. Webb |
| ACOE1707 | France | Languedoc-Roussillon | Hérault | Montpellier | 43,64435 | 3,848899 | 22/04/2005 | Coeur.& Jous. | Rosaceae | Prunus dulcis (Miller) D.A. Webb |
| ACOE1710 | France | Midi-Pyrénées | Gers | Saint-Clar | 43,891433 | 0,77306 | 28/04/2005 | Coeur.& Jous. | Rosaceae | Prunus dulcis (Miller) D.A. Webb |
| ACOE1714 | France | Midi-Pyrénées | Gers | Castelnau-d'Arbieu | 43,883999 | 0,702478 | 28/04/2005 | Coeur.& Jous. | Rosaceae | Rosaceae sp. L. |
| ACOE1715 | France | Midi-Pyrénées | Tarn-et-Garonne | Gramont | 43,937099 | 0,797778 | 29/04/2005 | Coeur.& Jous. | Compositae | Tragopogon sp. L. |
| ACOE1716 | France | Midi-Pyrénées | Tarn-et-Garonne | Gramont | 43,937099 | 0,797778 | 29/04/2005 | Coeur.& Jous. | Rosaceae | Prunus domestica L. |
| ACOE1721 | France | Midi-Pyrénées | Gers | Castelnau-d'Arbieu | 43,88566 | 0,703535 | 30/04/2005 | Coeur d'Acier | Valerianaceae | Centranthus ruber (L.) DC. |
| ACOE1722 | France | Midi-Pyrénées | Gers | Castelnau-d'Arbieu | 43,88566 | 0,703535 | 30/04/2005 | Coeur d'Acier | Urticaceae | Urtica urens L. |
| ACOE1725 | France | Midi-Pyrénées | Gers | Castelnau-d'Arbieu | 43,88566 | 0,703535 | 30/04/2005 | Coeur d'Acier | Scrophulariaceae | Veronica sp. L. |
| ACOE1729 | France | Languedoc-Roussillon | Hérault | Montpellier | 43,619327 | 3,893105 | 04/05/2005 | Coeur.& Jous. | Rosaceae | Prunus cerasus L. |
| ACOE1730 | France | Centre | Loiret | Germigny-Des-Pres | 47,844898 | 2,266678 | 05/05/2005 | Jousselin | Rosaceae | Prunus persica (L.) Batsch |
| ACOE1731 | France | Centre | Loiret | Chateauneuf-sur-Loire | 47,865433 | 2,226987 | 05/05/2005 | Jousselin | Rosaceae | Prunus cerasus L. |
| ACOE1732 | France | Centre | Loir-et-Cher | Pierrefitte-sur-Sauldre | 47,487456 | 2,077375 | 05/05/2005 | Jousselin | Polygonaceae | Rumex sp. L. |
| ACOE1734 | France | Languedoc-Roussillon | Hérault | Montferrier-sur-Lez | 43,671203 | 3,866294 | 09/05/2005 | Coeur d'Acier | Salicaceae | Populus sp. L. |
| ACOE1736 | France | Rhône-Alpes | Drôme | Saint-Marcel-les-Valence | 44,977722 | 4,919713 | 10/05/2005 | Coeur.& Jous. | Rosaceae | Prunus persica (L.) Batsch |
| ACOE1737 | France | Rhône-Alpes | Drôme | Saint-Marcel-les-Valence | 44,977722 | 4,919713 | 10/05/2005 | Coeur.& Jous. | Rosaceae | Prunus persica (L.) Batsch |
| ACOE1744 | France | Rhône-Alpes | Isère | Pont-en-Royans | 45,06523 | 5,359353 | 11/05/2005 | Coeur.& Jous. | Rosaceae | Rubus sp. L. |
| ACOE1745 | France | Alsace | Haut-Rhin | Colmar | 48,103453 | 7,304743 | 12/05/2005 | Coeur.& Jous. |  |  |
| ACOE1751 | France | Alsace | Haut-Rhin | Colmar | 48,049385 | 7,382353 | 13/05/2005 | Coeur.& Jous. | Asteraceae | Arctium sp. L. |
| ACOE1755 | France | Alsace | Haut-Rhin | Kaysersberg | 48,138298 | 7,262578 | 13/05/2005 | Coeur.& Jous. | Asteraceae | Taraxacum sp. F.H. Wiggers |
| ACOE1759 | France | Alsace | Haut-Rhin | Colmar | 48,049385 | 7,382353 | 14/05/2005 | Coeur.& Jous. | Aceraceae | Acer sp. L. |
| ACOE1760 | France | Languedoc-Roussillon | Gard | Le Vigan | 44,088544 | 3,520094 | 16/06/2005 | Coeur.& Jous. | Caryophyllaceae | Silene vulgaris (Moench.) Garcke |
| ACOE1761 | France | Languedoc-Roussillon | Gard | Le Vigan | 44,088544 | 3,520094 | 16/06/2005 | Coeur.& Jous. | Compositae | Tragopogon sp. L. |
| ACOE1762 | France | Languedoc-Roussillon | Gard | Le Vigan | 44,088544 | 3,520094 | 16/06/2005 | Coeur.& Jous. | Caryophyllaceae | Silene dioica (L.) Clairv. |
| ACOE1768 | France | Languedoc-Roussillon | Gard | Le Vigan | 44,088544 | 3,520094 | 16/06/2005 | Coeur.& Jous. | Caryophyllaceae | Silene dioica (L.) Clairv. |
| ACOE1769 | France | Languedoc-Roussillon | Gard | Le Vigan | 44,088544 | 3,520094 | 16/06/2005 | Coeur.& Jous. | Apiaceae | Apiaceae sp. Lindl. |
| ACOE1771 | France | Languedoc-Roussillon | Hérault | Saint-Guilhem-le-Desert | 43,7337 | 3,550078 | 29/06/2005 | Coeur.& Jous. | Scrophulariaceae | Linaria repens (L.) Miller |
| ACOE1772 | France | Provence-Alpes-Côte- d'Azur | Hautes-Alpes | Villar-d'Arene | 45,039285 | 6,348407 | 07/07/2005 | Coeur.& Jous. | Boraginaceae | Cerinthe glabra Miller |
| ACOE1777 | France | Haute-Normandie | Seine-Maritime | Louvetot | 49,572002 | 0,713278 | 19/07/2005 | Coeur d'Acier | Fabaceae | Lupinus sp. L. |
| ACOE1778 | France | Haute-Normandie | Seine-Maritime | Louvetot | 49,572002 | 0,713278 | 19/07/2005 | Coeur d'Acier | Rosaceae | Rubus idaeus L. |
| ACOE1779 | France | Haute-Normandie | Seine-Maritime | Louvetot | 49,572002 | 0,713278 | 19/07/2005 | Coeur d'Acier | Rosaceae | Spiraea sp. L. |
| ACOE1780 | France | Haute-Normandie | Seine-Maritime | Louvetot | 49,572002 | 0,713278 | 19/07/2005 | Coeur d'Acier | Betulaceae | Betula sp. L. |
| ACOE1781 | France | Haute-Normandie | Seine-Maritime | Louvetot | 49,572002 | 0,713278 | 19/07/2005 | Coeur d'Acier | Oenotheraceae | Epilobium sp. L. |
| ACOE1782 | France | Haute-Normandie | Seine-Maritime | Louvetot | 49,572002 | 0,713278 | 19/07/2005 | Coeur d'Acier | Oenotheraceae | Epilobium sp. L. |
| ACOE1783 | France | Haute-Normandie | Seine-Maritime | Louvetot | 49,572002 | 0,713278 | 19/07/2005 | Coeur d'Acier | Plantaginaceae | Plantago major L. |
| ACOE1784 | France | Haute-Normandie | Seine-Maritime | Louvetot | 49,572002 | 0,713278 | 19/07/2005 | Coeur d'Acier | Rosaceae | Spiraea vanhouttei Zabel |
| ACOE1785 | France | Haute-Normandie | Seine-Maritime | Louvetot | 49,572002 | 0,713278 | 19/07/2005 | Coeur d'Acier | Cupressaceae | Juniperus sp. L. |
| ACOE1786 | France | Haute-Normandie | Seine-Maritime | Louvetot | 49,572002 | 0,713278 | 19/07/2005 | Coeur d'Acier | Asteraceae | Asteraceae sp. |
| ACOE1787 | France | Haute-Normandie | Seine-Maritime | Louvetot | 49,572002 | 0,713278 | 19/07/2005 | Coeur d'Acier | Caprifoliaceae | Lonicera sp. L. |
| ACOE1790 | France | Midi-Pyrénées | Ariège | Mijanes | 42,736667 | 2,005278 | 10/08/2005 | Coeur.& Jous. | Ranunculaceae | Aconitum sp. L. |
| ACOE1791 | France | Languedoc-Roussillon | Lozère | La Bastide-Puylaurent | 44,59368 | 3,90607 | 31/08/2005 | Coeur.& Jous. | Plantaginaceae | Plantago major L. |
| ACOE1792 | France | Languedoc-Roussillon | Lozère | La Bastide-Puylaurent | 44,59368 | 3,90607 | 31/08/2005 | Coeur.& Jous. | Plantaginaceae | Plantago lanceolata L. |
| ACOE1794 | France | Languedoc-Roussillon | Lozère | La Bastide-Puylaurent | 44,587107 | 3,905933 | 31/08/2005 | Coeur.& Jous. | Asteraceae | Senecio sp. L. |
| ACOE1926 | France | Languedoc-Roussillon | Hérault | Rouet | 43,820397 | 3,813283 | 03/04/2006 | Cœur. & Jouss. | Asteraceae | Senecio vulgaris L. |
| ACOE1928 | France | Languedoc-Roussillon | Hérault | Saint-Guilhem-le-Desert | 43,7337 | 3,550078 | 18/04/2006 | Coeur d'Acier | Rosaceae | Prunus spinosa L. |
| ACOE1929 | France | Languedoc-Roussillon | Hérault | Saint-Guilhem-le-Desert | 43,7337 | 3,550078 | 18/04/2006 | Coeur d'Acier | Rosaceae | Prunus spinosa L. |
| ACOE1930 | France | Languedoc-Roussillon | Hérault | Saint-Guilhem-le-Desert | 43,7337 | 3,550078 | 18/04/2006 | Cœur. & Jouss. | Asteraceae | Senecio vulgaris L. |
| ACOE1931 | France | Languedoc-Roussillon | Hérault | Saint-Guilhem-le-Desert | 43,7337 | 3,550078 | 18/04/2006 | Coeur d'Acier | Brassicaceae | Brassicaceae sp. Burnett |
| ACOE1932 | France | Languedoc-Roussillon | Hérault | Saint-Guilhem-le-Desert | 43,7337 | 3,550078 | 18/04/2006 | Coeur d'Acier | Rosaceae | Malus sp. P. Miller |
| ACOE1933 | France | Languedoc-Roussillon | Hérault | Montferrier-sur-Lez | 43,682624 | 3,874869 | 10/05/2006 | Coeur d'Acier | Poaceae | Poaceae sp. Barnhart |
| ACOE1934 | France | Languedoc-Roussillon | Hérault | Montferrier-sur-Lez | 43,682624 | 3,874869 | 10/05/2006 | Coeur d'Acier | Caryophyllaceae | Silene latifolia (Miller) Greuter & Burdet |
| ACOE1935 | France | Languedoc-Roussillon | Hérault | Montferrier-sur-Lez | 43,682624 | 3,874869 | 10/05/2006 | Coeur d'Acier | Asteraceae | Cichorium intybus L. |
| ACOE1936 | France | Languedoc-Roussillon | Hérault | Montferrier-sur-Lez | 43,682624 | 3,874869 | 10/05/2006 | Coeur d'Acier | Poaceae | Poaceae sp. Barnhart |
| ACOE1937 | France | Languedoc-Roussillon | Hérault | Montferrier-sur-Lez | 43,682624 | 3,874869 | 10/05/2006 | Cœur. & Jouss. | Rosaceae | Prunus spinosa L. |
| ACOE1938 | France | Languedoc-Roussillon | Hérault | Montferrier-sur-Lez | 43,682624 | 3,874869 | 10/05/2006 | Coeur d'Acier | Scrophulariaceae | Linaria sp. |
| ACOE1939 | France | Languedoc-Roussillon | Hérault | Rouet | 43,820397 | 3,813283 | 17/05/2006 | Cœur. & Jouss. | Rosaceae | Prunus spinosa L. |
| ACOE1940 | France |  |  |  |  |  | 15/05/2006 | Coeur d'Acier | Alliaceae | Allium cepa L. |
| ACOE1942 | Italy | Sicilia (it) | Catania (it) | Catania | 37,51010192 | 15,082678 | 20/05/2006 | Coeur d'Acier | Apocynaceae | Nerium oleander L. |
| ACOE1943 | Italy | Sicilia (it) | Catania (it) | Catania | 37,51010192 | 15,082678 | 20/05/2006 | Coeur d'Acier | Rosaceae | Rosa sp. L. |
| ACOE1944 | Italy | Sicilia (it) | Catania (it) | Catania | 37,499534 | 15,084506 | 20/05/2006 | Coeur d'Acier | Anacardiaceae | Pistacia sp. L. |
| ACOE1945 | Italy | Sicilia (it) | Catania (it) | Catania | 37,499534 | 15,084506 | 20/05/2006 | Coeur d'Acier | Anacardiaceae | Pistacia sp. L. |
| ACOE1946 | Italy | Sicilia (it) | Catania (it) | Catania | 37,499534 | 15,084506 | 20/05/2006 | Coeur d'Acier | Asteraceae | Chrysanthemum sp. L. |
| ACOE1947 | Italy | Sicilia (it) | Catania (it) | Catania | 37,500428 | 15,098856 | 20/05/2006 | Coeur d'Acier | Solanaceae | Solanum sp. L. |
| ACOE1948 | Italy | Sicilia (it) | Catania (it) | Catania | 37,500428 | 15,098856 | 20/05/2006 | Coeur d'Acier | Solanaceae | Solanum sp. L. |
| ACOE1949 | Italy | Sicilia (it) | Catania (it) | Catania | 37,500428 | 15,098856 | 20/05/2006 | Coeur d'Acier | Poaceae | Poaceae sp. Barnhart |
| ACOE1950 | Italy | Sicilia (it) | Catania (it) | Catania | 37,5 | 15,1 | 20/05/2006 | Coeur d'Acier | Apiaceae | Daucus carota L. |
| ACOE1951 | Italy | Sicilia (it) | Catania (it) | Catania | 37,5 | 15,1 | 20/05/2006 | Coeur d'Acier | Apiaceae | Daucus carota L. |
| ACOE1952 | Italy | Sicilia (it) | Catania (it) | Catania | 37,5 | 15,1 | 20/05/2006 | Coeur d'Acier | Asteraceae | Dittrichia viscosa (L.) W. Greuter |
| ACOE1953 | Italy | Sicilia (it) | Catania (it) | Catania | 37,5 | 15,1 | 20/05/2006 | Coeur d'Acier | Asteraceae | Sonchus sp. L. |
| ACOE1954 | Italy | Sicilia (it) | Catania (it) | Catania | 37,5 | 15,1 | 20/05/2006 | Coeur d'Acier | Brassicaceae | Diplotaxis erucoides (L.) DC. |
| ACOE1955 | Italy | Sicilia (it) | Catania (it) | Catania | 37,5 | 15,1 | 20/05/2006 | Coeur d'Acier | Leguminosae | Melilotus sp. P. Miller |
| ACOE1956 | Italy | Sicilia (it) | Catania (it) | Catania | 37,5 | 15,1 | 20/05/2006 | Coeur d'Acier | Scrophulariaceae | Scrophulariaceae sp. |
| ACOE1957 | Italy | Sicilia (it) | Catania (it) | Catania | 37,5 | 15,1 | 21/05/2006 | Coeur d'Acier | Fagaceae | Quercus sp. L. |
| ACOE1959 | Italy | Sicilia (it) | Catania (it) | Catania | 37,5 | 15,1 | 21/05/2006 | Coeur d'Acier | Fabaceae | Fabaceae sp. Lindl. |
| ACOE1960 | Italy | Sicilia (it) | Catania (it) | Catania | 37,524098 | 15,113458 | 21/05/2006 | Coeur d'Acier | Moraceae | Ficus microcarpa |
| ACOE1961 | Italy | Sicilia (it) | Catania (it) | Catania | 37,524098 | 15,113458 | 21/05/2006 | Coeur d'Acier |  |  |
| ACOE1962 | Italy | Sicilia (it) | Catania (it) | Catania | 37,524098 | 15,113458 | 21/05/2006 | Coeur d'Acier | Asteraceae | Asteraceae sp. |
| ACOE1963 | Italy | Sicilia (it) | Catania (it) | Catania | 37,524098 | 15,113458 | 21/05/2006 | Coeur d'Acier | Cruciferae | Isatis tinctoria L. |
| ACOE1965 | Italy | Sicilia (it) | Catania (it) | Catania | 37,5 | 15,1 | 21/05/2006 | Coeur d'Acier | Asteraceae | Sonchus sp. L. |
| ACOE1966 | Italy | Sicilia (it) | Catania (it) | Catania | 37,5 | 15,1 | 21/05/2006 | Coeur d'Acier | Tiliaceae | Tilia sp. L. |
| ACOE1967 | Italy | Sicilia (it) | Catania (it) | Acireale | 37,616699 | 15,1667 | 20/05/2006 | Coeur d'Acier | Fagaceae | Quercus ilex L. |
| ACOE1968 | Italy | Sicilia (it) | Ragusa (it) | Ragusa | 36,916698 | 14,7333 | 22/05/2006 | Coeur d'Acier | Tiliaceae | Tilia x-vulgaris Hayne |
| ACOE1969 | Italy | Sicilia (it) | Ragusa (it) | Ragusa | 36,926698 | 14,748393 | 22/05/2006 | Coeur d'Acier | Cupressaceae | Chamaecyparis sp. SPACH |
| ACOE1971 | Italy | Sicilia (it) | Ragusa (it) | Ragusa | 36,926698 | 14,748393 | 22/05/2006 | Coeur d'Acier | Pinaceae | Cedrus sp. |
| ACOE1973 | Italy | Sicilia (it) | Ragusa (it) | Ragusa | 36,926698 | 14,748393 | 22/05/2006 | Coeur d'Acier | Pinaceae | Cedrus sp. |
| ACOE1974 | Italy | Sicilia (it) | Ragusa (it) | Ragusa | 36,926698 | 14,748393 | 22/05/2006 | Coeur d'Acier | Pinaceae | Cedrus sp. |
| ACOE1975 | Italy | Sicilia (it) | Catania (it) | Piedimonte = Piedimonte Etneo | 37,799999 | 15,2 | 23/05/2006 | Coeur d'Acier | Ranunculaceae | Clematis sp. L. |
| ACOE1976 | Italy | Sicilia (it) | Catania (it) | Linguaglossa | 37,842799 | 15,141783 | 23/05/2006 | Coeur d'Acier | Caryophyllaceae | Silene vulgaris (Moench.) Garcke |
| ACOE1977 | Italy | Sicilia (it) | Catania (it) | Linguaglossa | 37,833302 | 15,1333 | 23/05/2006 | Coeur d'Acier | Umbelliferae | Ferula communis L. |
| ACOE1978 | Italy | Sicilia (it) | Catania (it) | Linguaglossa | 37,833302 | 15,1333 | 23/05/2006 | Coeur d'Acier | Caryophyllaceae | Silene vulgaris (Moench.) Garcke |
| ACOE1979 | Italy | Sicilia (it) | Catania (it) | Linguaglossa | 37,833302 | 15,1333 | 23/05/2006 | Coeur d'Acier | Caryophyllaceae | Silene vulgaris (Moench.) Garcke |
| ACOE1980 | Italy | Sicilia (it) | Catania (it) | Linguaglossa | 37,833302 | 15,1333 | 23/05/2006 | Coeur d'Acier | Compositae | Tragopogon sp. L. |
| ACOE1981 | Italy | Sicilia (it) | Catania (it) | Linguaglossa | 37,833302 | 15,1333 | 23/05/2006 | Coeur d'Acier | Fabaceae | Vicia cracca L. |
| ACOE1982 | Italy | Sicilia (it) | Catania (it) | Linguaglossa | 37,833302 | 15,1333 | 23/05/2006 | Cœur. & Jouss. | Polygonaceae | Rumex acetosella L. |
| ACOE1983 | Italy | Sicilia (it) | Catania (it) | Linguaglossa | 37,833302 | 15,1333 | 23/05/2006 | Coeur d'Acier | Cruciferae | Isatis tinctoria L. |
| ACOE1984 | Italy | Sicilia (it) | Catania (it) | Linguaglossa | 37,833302 | 15,1333 | 24/05/2006 | Coeur d'Acier | Rosaceae | Prunus dulcis (Miller) D.A. Webb |
| ACOE1985 | Italy | Sicilia (it) | Catania (it) | Linguaglossa | 37,833302 | 15,1333 | 24/05/2006 | Coeur d'Acier | Asteraceae | Cirsium arvense (L.) Scop. |
| ACOE1986 | Italy | Sicilia (it) | Catania (it) | Linguaglossa | 37,833302 | 15,1333 | 24/05/2006 | Coeur d'Acier | Compositae | Tragopogon sp. L. |
| ACOE1987 | Italy | Sicilia (it) | Catania (it) | Castiglione di Sicilia | 37,88098 | 15,124026 | 24/05/2006 | Coeur d'Acier | Asteraceae | Asteraceae sp. |
| ACOE1988 | Italy | Sicilia (it) | Catania (it) | Castiglione di Sicilia | 37,88098 | 15,124026 | 24/05/2006 | Coeur d'Acier | Fagaceae | Castanea sativa Miller |
| ACOE1989 | Italy | Sicilia (it) | Catania (it) | Castiglione di Sicilia | 37,88098 | 15,124026 | 24/05/2006 | Coeur d'Acier | Rosaceae | Prunus spinosa L. |
| ACOE1990 | Italy | Sicilia (it) | Catania (it) | Castiglione di Sicilia | 37,88098 | 15,124026 | 24/05/2006 | Coeur d'Acier | Rosaceae | Prunus spinosa L. |
| ACOE1991 | Italy | Sicilia (it) | Catania (it) | Castiglione di Sicilia | 37,88098 | 15,124026 | 24/05/2006 | Coeur d'Acier | Fagaceae | Quercus pubescens WILLD. |
| ACOE1992 | Italy | Sicilia (it) | Catania (it) | Randazzo | 37,873655 | 14,993997 | 23/05/2006 | Coeur d'Acier |  |  |
| ACOE1993 | Italy | Sicilia (it) | Catania (it) | Randazzo | 37,873655 | 14,993997 | 23/05/2006 | Coeur d'Acier | Fabaceae | Spartium junceum L. |
| ACOE1994 | Italy | Sicilia (it) | Catania (it) | Randazzo | 37,873655 | 14,993997 | 23/05/2006 | Coeur d'Acier | Asteraceae | Carduus sp. L. |
| ACOE1995 | Italy | Sicilia (it) | Catania (it) | Linguaglossa | 37,842741 | 15,141786 | 23/05/2006 | Coeur d'Acier | Geraniaceae | Pelargonium sp. L'Heritier ex W. Aiton |
| ACOE1996 | Italy | Sicilia (it) | Catania (it) | Linguaglossa | 37,842741 | 15,141786 | 23/05/2006 | Coeur d'Acier | Fabaceae | Wisteria sp. Nutt. |
| ACOE1997 | Italy | Sicilia (it) | Catania (it) | Linguaglossa | 37,842741 | 15,141786 | 23/05/2006 | Coeur d'Acier | Asteraceae | Cirsium arvense (L.) Scop. |
| ACOE1998 | Italy | Sicilia (it) | Catania (it) | Linguaglossa | 37,842741 | 15,141786 | 23/05/2006 | Coeur d'Acier | Umbelliferae | Ferula communis L. |
| ACOE1999 | Italy | Sicilia (it) | Catania (it) | Linguaglossa | 37,842741 | 15,141786 | 25/05/2006 | Coeur d'Acier | Juglandaceae | Juglans regia L. |
| ACOE2000 | Italy | Sicilia (it) | Catania (it) | Linguaglossa | 37,842741 | 15,141786 | 25/05/2006 | Coeur d'Acier | Umbelliferae | Ferula communis L. |
| ACOE2001 | Italy | Sicilia (it) | Catania (it) | Linguaglossa | 37,829846 | 15,134507 | 25/05/2006 | Coeur d'Acier | Betulaceae | Betula pendula Roth |
| ACOE2002 | Italy | Sicilia (it) | Catania (it) | Linguaglossa | 37,829846 | 15,134507 | 25/05/2006 | Coeur d'Acier | Betulaceae | Betula pendula Roth |
| ACOE2003 | Italy | Sicilia (it) | Catania (it) | Linguaglossa | 37,791601 | 15,13166 | 26/05/2006 | Coeur d'Acier | Aceraceae | Acer campestre L. |
| ACOE2004 | Italy | Sicilia (it) | Catania (it) | Linguaglossa | 37,791601 | 15,13166 | 26/05/2006 | Coeur d'Acier | Caryophyllaceae | Silene vulgaris (Moench.) Garcke |
| ACOE2005 | Italy | Sicilia (it) | Catania (it) | Linguaglossa | 37,791601 | 15,13166 | 26/05/2006 | Coeur d'Acier | Asteraceae | Achillea sp. L. |
| ACOE2006 | Italy | Sicilia (it) | Catania (it) | Linguaglossa | 37,791601 | 15,13166 | 26/05/2006 | Coeur d'Acier | Caryophyllaceae | Silene vulgaris (Moench.) Garcke |
| ACOE2007 | Italy | Sicilia (it) | Catania (it) | Linguaglossa | 37,791601 | 15,13166 | 26/05/2006 | Coeur d'Acier | Labiatae | Mentha sp. L. |
| ACOE2008 | Italy | Sicilia (it) | Catania (it) | Linguaglossa | 37,791601 | 15,13166 | 26/05/2006 | Coeur d'Acier | Asteraceae | Asteraceae sp. |
| ACOE2011 | Italy | Sicilia (it) | Catania (it) | Sant'Alfio | 37,742791 | 15,139163 | 26/05/2006 | Coeur d'Acier | Rosaceae | Rubus sp. L. |
| ACOE2012 | Italy | Sicilia (it) | Catania (it) | Sant'Alfio | 37,742791 | 15,139163 | 26/05/2006 | Coeur d'Acier | Rubiaceae | Galium sp. L. |
| ACOE2013 | Italy | Sicilia (it) | Catania (it) | Sant'Alfio | 37,742791 | 15,139163 | 26/05/2006 | Coeur d'Acier | Polygonaceae | Rumex sp. L. |
| ACOE2014 | Italy | Sicilia (it) | Catania (it) | Sant'Alfio | 37,742791 | 15,139163 | 26/05/2006 | Coeur d'Acier | Corylaceae | Corylus avellana L. |
| ACOE2015 | Italy | Sicilia (it) | Catania (it) | Sant'Alfio | 37,742791 | 15,139163 | 26/05/2006 | Coeur d'Acier | Corylaceae | Corylus avellana L. |
| ACOE2017 | Italy | Sicilia (it) | Catania (it) | Zafferana Etnea | 37,686893 | 15,109471 | 26/05/2006 | Coeur d'Acier | Pinaceae | Pinus sp. L. |
| ACOE2018 | Italy | Sicilia (it) | Catania (it) | Zafferana Etnea | 37,686893 | 15,109471 | 26/05/2006 | Coeur d'Acier | Apiaceae | Daucus carota L. |
| ACOE2020 | Italy | Sicilia (it) | Catania (it) | Zafferana Etnea | 37,686893 | 15,109471 | 26/05/2006 | Coeur d'Acier | Boraginaceae | Cerinthe sp. |
| ACOE2022 | Italy | Sicilia (it) | Catania (it) | Zafferana Etnea | 37,686893 | 15,109471 | 26/05/2006 | Coeur d'Acier | Asteraceae | Chrysanthemum sp. L. |
| ACOE2023 | Italy | Sicilia (it) | Catania (it) | Fiumefreddo di Sicilia | 37,786298 | 15,233708 | 27/05/2006 | Coeur d'Acier | Poaceae | Poaceae sp. Barnhart |
| ACOE2024 | Italy | Sicilia (it) | Catania (it) | Fiumefreddo di Sicilia | 37,786298 | 15,233708 | 27/05/2006 | Coeur d'Acier | Apiaceae | Apiaceae sp. Lindl. |
| ACOE2025 | Italy | Sicilia (it) | Catania (it) | Fiumefreddo di Sicilia | 37,786298 | 15,233708 | 27/05/2006 | Coeur d'Acier | Umbelliferae | Cachrys sicula L. |
| ACOE2026 | Italy | Sicilia (it) | Catania (it) | Fiumefreddo di Sicilia | 37,792201 | 15,20628 | 27/05/2006 | Coeur d'Acier | Scrophulariaceae | Verbascum sp. L. |
| ACOE2028 | Italy | Sicilia (it) | Catania (it) | Fiumefreddo di Sicilia | 37,792201 | 15,20628 | 27/05/2006 | Coeur d'Acier | Fabaceae | Robinia pseudoacacia L. |
| ACOE2029 | Italy | Sicilia (it) | Catania (it) | Linguaglossa | 37,842741 | 15,141786 | 27/05/2006 | Coeur d'Acier | Iridaceae | Iris sp. L. |
| ACOE2030 | Italy | Sicilia (it) | Catania (it) | Linguaglossa | 37,842741 | 15,141786 | 27/05/2006 | Coeur d'Acier | Salicaceae | Salix babylonica L. |
| ACOE2031 | Italy | Sicilia (it) | Catania (it) | Linguaglossa | 37,842741 | 15,141786 | 28/05/2006 | Coeur d'Acier | Salicaceae | Salix babylonica L. |
| ACOE2032 | Italy | Sicilia (it) | Catania (it) | Trecastagni | 37,616699 | 15,0833 | 28/05/2006 | Coeur d'Acier | Fagaceae | Quercus sp. L. |
| ACOE2034 | Italy | Sicilia (it) | Catania (it) | Trecastagni | 37,616699 | 15,0833 | 28/05/2006 | Coeur d'Acier | Fagaceae | Quercus sp. L. |
| ACOE2035 | Italy | Sicilia (it) | Catania (it) | Paterno | 37,567778 | 14,900278 | 28/05/2006 | Coeur d'Acier | Salicaceae | Populus sp. L. |
| ACOE2036 | Italy | Sicilia (it) | Catania (it) | Catania | 37,51010192 | 15,082678 | 29/05/2006 | Coeur d'Acier | Crassulaceae | Aeonium canariense (L.) |
| ACOE2041 | Italy | Sicilia (it) | Catania (it) | Catania | 37,51010192 | 15,082678 | 29/05/2006 | Coeur d'Acier | Asparagaceae | Asparagus sprenegeri |
| ACOE2042 | Italy | Sicilia (it) | Catania (it) | Catania | 37,51010192 | 15,082678 | 29/05/2006 | Coeur d'Acier | Asparagaceae | Asparagus sprenegeri |
| ACOE2043 | Italy | Sicilia (it) | Catania (it) | Catania | 37,518235 | 15,071077 | 29/05/2006 | Coeur d'Acier | Gramineae | Phyllostachys sp. SIEBOLD & ZUCC. |
| ACOE2044 | Italy | Sicilia (it) | Catania (it) | Zafferana Etnea | 37,704387 | 15,075645 | 30/05/2006 | Coeur d'Acier | Asteraceae | Achillea ligustica All. |
| ACOE2045 | Italy | Sicilia (it) | Catania (it) | Zafferana Etnea | 37,704387 | 15,075645 | 30/05/2006 | Coeur d'Acier | Apiaceae | Pimpinella tragium Vill. |
| ACOE2046 | Italy | Sicilia (it) | Catania (it) | Zafferana Etnea | 37,704387 | 15,075645 | 30/05/2006 | Coeur d'Acier | Asteraceae | Tanacetum vulgare L. |
| ACOE2047 | Italy | Sicilia (it) | Catania (it) | Zafferana Etnea | 37,704387 | 15,075645 | 30/05/2006 | Coeur d'Acier | Scrophulariaceae | Linaria sp. |
| ACOE2048 | Italy | Sicilia (it) | Catania (it) | Zafferana Etnea | 37,704387 | 15,075645 | 30/05/2006 | Coeur d'Acier | Asteraceae | Tanacetum vulgare L. |
| ACOE2049 | Italy | Sicilia (it) | Catania (it) | Zafferana Etnea | 37,704387 | 15,075645 | 30/05/2006 | Coeur d'Acier | Rosaceae | Rubus sp. L. |
| ACOE2050 | Italy | Sicilia (it) | Catania (it) | Sant'Alfio | 37,782621 | 15,132029 | 30/05/2006 | Coeur d'Acier | Umbelliferae | Ferula communis L. |
| ACOE2051 | Italy | Sicilia (it) | Catania (it) | Sant'Alfio | 37,782621 | 15,132029 | 30/05/2006 | Coeur d'Acier | Asteraceae | Hypochoeris sp. L. |
| ACOE2052 | Italy | Sicilia (it) | Catania (it) | Randazzo | 37,921632 | 14,957012 | 30/05/2006 | Coeur d'Acier | Umbelliferae | Opopanax chironium (L.) KOCH |
| ACOE2053 | Italy | Sicilia (it) | Catania (it) | Randazzo | 37,921632 | 14,957012 | 30/05/2006 | Coeur d'Acier | Umbelliferae | Opopanax chironium (L.) KOCH |
| ACOE2054 | Italy | Sicilia (it) | Catania (it) | Randazzo | 37,921632 | 14,957012 | 30/05/2006 | Coeur d'Acier | Rosaceae | Malus sp. P. Miller |
| ACOE2055 | Italy | Sicilia (it) | Catania (it) | Randazzo | 37,921632 | 14,957012 | 30/05/2006 | Coeur d'Acier | Rosaceae | Malus sp. P. Miller |
| ACOE2056 | Italy | Sicilia (it) | Catania (it) | Randazzo | 37,921632 | 14,957012 | 30/05/2006 | Coeur d'Acier | Asteraceae | Achillea ligustica All. |
| ACOE2057 | Italy | Sicilia (it) | Messina (it) | Santa Domenica Vittoria | 37,941924 | 14,959767 | 30/05/2006 | Coeur d'Acier | Oleaceae | Fraxinus sp. L. |
| ACOE2058 | Italy | Sicilia (it) | Messina (it) | Santa Domenica Vittoria | 37,941924 | 14,959767 | 30/05/2006 | Coeur d'Acier | Aceraceae | Acer campestre L. |
| ACOE2059 | Italy | Sicilia (it) | Messina (it) | Santa Domenica Vittoria | 37,941924 | 14,959767 | 30/05/2006 | Coeur d'Acier | Fabaceae | Vicia sp. L. |
| ACOE2060 | Italy | Sicilia (it) | Catania (it) | Floresta | 37,992071 | 14,930588 | 30/05/2006 | Coeur d'Acier | Apiaceae | Heracleum sphondylium |
| ACOE2061 | Italy | Sicilia (it) | Catania (it) | Catania | 37,518235 | 15,071077 | 30/05/2006 | Coeur d'Acier | Betulaceae | Alnus cordata (LOISEL.) LOISEL. |
| ACOE2062 | Italy | Sicilia (it) | Catania (it) | Catania | 37,518235 | 15,071077 | 31/05/2006 | Coeur d'Acier | Punicaceae | Punica granatum L. |
| ACOE2063 | France | Rhône-Alpes | Haute-Savoie | La Roche-sur-Foron | 46,065598 | 6,310378 | 04/06/2006 | Jousselin | Caryophyllaceae | Silene dioica (L.) Clairv. |
| ACOE2064 | France | Languedoc-Roussillon | Gard | Le Vigan | 44,088544 | 3,520094 | 14/06/2006 | Cœur. & Jouss. | Caryophyllaceae | Silene dioica (L.) Clairv. |
| ACOE2065 | France | Midi-Pyrénées | Ariège | Ax-les-Thermes | 42,717602 | 1,840078 | 22/06/2006 | Coeur d'Acier | Fabaceae | Robinia pseudoacacia L. |
| ACOE2066 | France | Midi-Pyrénées | Ariège | Ax-les-Thermes | 42,726656 | 1,913367 | 22/06/2006 | Coeur d'Acier | Fagaceae | Quercus sp. L. |
| ACOE2067 | France | Midi-Pyrénées | Ariège | Ax-les-Thermes | 42,726656 | 1,913367 | 22/06/2006 | Coeur d'Acier | Salicaceae | Salix sp. L. |
| ACOE2069 | France | Midi-Pyrénées | Ariège | Ax-les-Thermes | 42,726656 | 1,913367 | 22/06/2006 | Coeur d'Acier | Scrophulariaceae | Linaria sp. |
| ACOE2070 | France | Midi-Pyrénées | Ariège | Ax-les-Thermes | 42,747498 | 1,956856 | 22/06/2006 | Coeur d'Acier | Ranunculaceae | Ranunculaceae sp. L. |
| ACOE2071 | France | Midi-Pyrénées | Ariège | Ax-les-Thermes | 42,747498 | 1,956856 | 22/06/2006 | Coeur d'Acier | Caryophyllaceae | Silene dioica (L.) Clairv. |
| ACOE2073 | France | Midi-Pyrénées | Ariège | Ax-les-Thermes | 42,747498 | 1,956856 | 22/06/2006 | Coeur d'Acier | Asteraceae | Asteraceae sp. |
| ACOE2074 | France | Midi-Pyrénées | Ariège | Ax-les-Thermes | 42,747498 | 1,956856 | 22/06/2006 | Coeur d'Acier | Cruciferaceae | Cruciferaceae sp. |
| ACOE2129 | United Kingdom | Scotland (en) |  | Shieldaig | 57,532009 | -5,649745 | 09/08/2006 | Coeur d'Acier | Betulaceae | Betula sp. L. |
| ACOE2130 | United Kingdom | Scotland (en) |  | Shieldaig | 57,532009 | -5,649745 | 09/08/2006 | Coeur d'Acier | Salicaceae | Salix sp. L. |
| ACOE2131 | United Kingdom | Scotland (en) |  | Contin | 57,5667 | -4,5667 | 09/08/2006 | Coeur d'Acier | Fabaceae | Vicia sp. L. |
| ACOE2132 | United Kingdom | Scotland (en) |  | Contin | 57,5667 | -4,5667 | 09/08/2006 | Coeur d'Acier | Rosaceae | Rosa sp. L. |
| ACOE2133 | United Kingdom | Scotland (en) |  | Contin | 57,5667 | -4,5667 | 09/08/2006 | Coeur d'Acier | Caprifoliaceae | Lonicera sp. L. |
| ACOE2134 | United Kingdom | Scotland (en) |  | Contin | 57,5667 | -4,5667 | 09/08/2006 | Coeur d'Acier | Apiaceae | Apiaceae sp. Lindl. |
| ACOE2135 | United Kingdom | Scotland (en) |  | Applecross | 57,432492 | -5,814932 | 11/08/2006 | Coeur d'Acier | Asteraceae | Sonchus sp. L. |
| ACOE2136 | United Kingdom | Scotland (en) |  | Applecross | 57,432492 | -5,814932 | 11/08/2006 | Coeur d'Acier | Apiaceae | Apiaceae sp. Lindl. |
| ACOE2137 | United Kingdom | Scotland (en) |  | Lochcarron | 57,400002 | -5,5 | 12/08/2006 | Coeur d'Acier | Rosaceae | Prunus sp. L. |
| ACOE2140 | United Kingdom | Scotland (en) |  | Kinlochewe | 57,604035 | -5,299391 | 12/08/2006 | Coeur d'Acier | Rosaceae | Rosa sp. L. |
| ACOE2141 | United Kingdom | Scotland (en) |  | Kinlochewe | 57,604035 | -5,299391 | 12/08/2006 | Coeur d'Acier | Asteraceae | Senecio vulgaris L. |
| ACOE2142 | United Kingdom | Scotland (en) |  | Kinlochewe | 57,604035 | -5,299391 | 12/08/2006 | Cœur. & Jouss. | Asteraceae | Senecio vulgaris L. |
| ACOE2143 | United Kingdom | Scotland (en) |  | Lochcarron | 57,400002 | -5,5 | 12/08/2006 | Cœur. & Jouss. | Rosaceae | Spiraea sp. L. |
| ACOE2144 | United Kingdom | Scotland (en) |  | Glencoe | 56,671709 | -5,086839 | 13/08/2006 | Coeur d'Acier | Asteraceae | Centaurea sp. L. |
| ACOE2145 | United Kingdom | Scotland (en) |  | Glencoe | 56,671709 | -5,086839 | 13/08/2006 | Coeur d'Acier | Asteraceae | Cirsium sp. P. Miller |
| ACOE2146 | United Kingdom | Scotland (en) |  | Glencoe | 56,671709 | -5,086839 | 13/08/2006 | Coeur d'Acier | Rosaceae | Rubus idaeus L. |
| ACOE2147 | United Kingdom | Scotland (en) |  | Glencoe | 56,671709 | -5,086839 | 13/08/2006 | Coeur d'Acier | Betulaceae | Betula sp. L. |
| ACOE2148 | United Kingdom | Scotland (en) |  | Glencoe | 56,671709 | -5,086839 | 13/08/2006 | Coeur d'Acier | Fagaceae | Quercus sp. L. |
| ACOE2149 | United Kingdom | Scotland (en) |  | Spean Bridge | 56,887803 | -4,919622 | 15/08/2006 | Coeur d'Acier | Asteraceae | Taraxacum officinale Weber |
| ACOE2150 | United Kingdom | Scotland (en) |  | Spean Bridge | 56,887803 | -4,919622 | 15/08/2006 | Coeur d'Acier | Fabaceae | Vicia sp. L. |
| ACOE2151 | United Kingdom | Scotland (en) |  | Spean Bridge | 56,887803 | -4,919622 | 15/08/2006 | Coeur d'Acier | Oenotheraceae | Epilobium angustifolium L. |
| ACOE2153 | United Kingdom | Scotland (en) |  | Spean Bridge | 56,887803 | -4,919622 | 15/08/2006 | Coeur d'Acier | Scrophulariaceae | Digitalis sp. L. |
| ACOE2154 | United Kingdom | Scotland (en) |  | Glencoe | 56,660527 | -5,037522 | 14/08/2006 | Cœur. & Jouss. | Crassulaceae | Rhodiola rosea L. |
| ACOE2155 | United Kingdom | Scotland (en) | Aberdeenshire (en) | Inverurie | 57,205331 | -2,460967 | 16/08/2006 | Coeur d'Acier | Fagaceae | Quercus sp. L. |
| ACOE2275 | France | Languedoc-Roussillon | Hérault | Montferrier-sur-Lez | 43,682624 | 3,874869 | 22/05/2007 | Coeur d'Acier | Fabaceae | Spartium junceum L. |
| ACOE2277 | France | Languedoc-Roussillon | Hérault | Montferrier-sur-Lez | 43,682624 | 3,874869 | 22/05/2007 | Coeur d'Acier | Salicaceae | Populus sp. L. |
| ACOE2278 | France | Languedoc-Roussillon | Hérault | Montferrier-sur-Lez | 43,682624 | 3,874869 | 22/05/2007 | Coeur d'Acier | Salicaceae | Populus sp. L. |
| ACOE2279 | France | Languedoc-Roussillon | Hérault | Montferrier-sur-Lez | 43,682624 | 3,874869 | 22/05/2007 | Coeur d'Acier | Asteraceae | Lactuca sp. L. |
| ACOE2280 | France | Languedoc-Roussillon | Hérault | Montferrier-sur-Lez | 43,682624 | 3,874869 | 22/05/2007 | Coeur d'Acier | Dipsacaceae | Dipsacaceae sp. Juss. |
| ACOE2281 | France | Languedoc-Roussillon | Hérault | Montferrier-sur-Lez | 43,682624 | 3,874869 | 22/05/2007 | Coeur d'Acier | Compositae | Tragopogon sp. L. |
| ACOE2284 | France | Languedoc-Roussillon | Hérault | Montferrier-sur-Lez | 43,682624 | 3,874869 | 22/05/2007 | Coeur d'Acier | Apiaceae | Foeniculum vulgare Miller |
| ACOE2285 | France | Languedoc-Roussillon | Hérault | Montferrier-sur-Lez | 43,682624 | 3,874869 | 22/05/2007 | Coeur d'Acier | Dipsacaceae | Dipsacaceae sp. Juss. |
| ACOE2286 | France | Languedoc-Roussillon | Hérault | Montferrier-sur-Lez | 43,682624 | 3,874869 | 22/05/2007 | Coeur d'Acier | Apocynaceae | Nerium oleander L. |
| ACOE2287 | France | Languedoc-Roussillon | Hérault | Montferrier-sur-Lez | 43,682624 | 3,874869 | 22/05/2007 | Coeur d'Acier | Rosaceae | Rubus sp. L. |
| ACOE2292 | France | Languedoc-Roussillon | Hérault | Montferrier-sur-Lez | 43,682624 | 3,874869 | 06/06/2007 | Coeur d'Acier | Ulmaceae | Ulmus sp. L. |
| ACOE2296 | France | Centre | Loir-et-Cher | Monthou-sur-Cher | 47,344498 | 1,294678 | 10/07/2007 | Jousselin | Asteraceae | Leucanthemum sp. |
| ACOE2299 | France | Languedoc-Roussillon | Pyrénées-Orientales | Porte-Puymorens | 42,566666 | 1,816667 | 21/08/2007 | Coeur d'Acier | Asteraceae | Tanacetum sp. L. |
| ACOE2360 | France | Rhône-Alpes | Drôme | Saint-Marcel-les-Valence | 44,969398 | 4,921667 | 22/05/2007 | Morel K. | Rosaceae | Malus domestica Borckh. |
| ACOE2406 | France | Languedoc-Roussillon | Hérault | Montpellier | 43,604322 | 3,871604 | 22/04/2008 | Jousselin | Rosaceae | Prunus domestica L. |
| ACOE2407 | France | Languedoc-Roussillon | Hérault | Montpellier | 43,604322 | 3,871604 | 22/04/2008 | Jousselin | Asteraceae | Cynara scolymus L. |
| ACOE2408 | France | Languedoc-Roussillon | Hérault | Montpellier | 43,61717 | 3,858032 | 23/05/2008 | Tayeh A. | Boraginaceae | Myosotis sp. L. |
| ACOE2411 | France | Languedoc-Roussillon | Hérault | Montferrier-sur-Lez | 43,682624 | 3,874869 | 05/05/2008 | Coeur d'Acier | Boraginaceae | Cynoglossum creticum MILLER |
| ACOE2412 | France | Languedoc-Roussillon | Hérault | Montpellier | 43,61717 | 3,858032 | 05/05/2008 | Tayeh A. | Rosaceae | Prunus domestica L. |
| ACOE2413 | France | Languedoc-Roussillon | Hérault | Montferrier-sur-Lez | 43,682778 | 3,873611 | 07/05/2008 | Coeur d'Acier | Caryophyllaceae | Silene latifolia (Miller) Greuter & Burdet |
| ACOE2414 | France | Languedoc-Roussillon | Hérault | Montferrier-sur-Lez | 43,682778 | 3,873611 | 07/05/2008 | Coeur d'Acier | Asteraceae | Cirsium arvense (L.) Scop. |
| ACOE2415 | France | Languedoc-Roussillon | Hérault | Montferrier-sur-Lez | 43,682778 | 3,873611 | 07/05/2008 | Coeur d'Acier | Poaceae | Triticum sp. L. |
| ACOE2416 | France | Languedoc-Roussillon | Hérault | Montferrier-sur-Lez | 43,682624 | 3,874869 | 10/05/2008 | Coeur d'Acier | Gramineae | Avena sp. L. |
| ACOE2418 | France | Languedoc-Roussillon | Hérault | Montferrier-sur-Lez | 43,682624 | 3,874869 | 11/05/2008 | Coeur d'Acier | Dipsacaceae | Dipsacaceae sp. Juss. |
| ACOE2419 | France | Languedoc-Roussillon | Hérault | Montferrier-sur-Lez | 43,682624 | 3,874869 | 11/05/2008 | Coeur d'Acier | Asteraceae | Sonchus sp. L. |
| ACOE2421 | France | Languedoc-Roussillon | Hérault | Montferrier-sur-Lez | 43,682624 | 3,874869 | 11/05/2008 | Coeur d'Acier | Rosaceae | Malus domestica Borckh. |
| ACOE2422 | France | Languedoc-Roussillon | Hérault | Montpellier | 43,608597 | 3,873178 | 06/05/2008 | Tayeh A. | Rosaceae | Prunus domestica L. |
| ACOE2424 | France | Provence-Alpes-Côte- d'Azur | Alpes-Maritimes | Antibes | 43,564944 | 7,12555 | 15/05/2008 | Coeur d'Acier | Rosaceae | Prunus domestica L. |
| ACOE2425 | France | Provence-Alpes-Côte- d'Azur | Alpes-Maritimes | Antibes | 43,564944 | 7,12555 | 15/05/2008 | Coeur d'Acier | Asteraceae | Santolina chamaecyparissus L. |
| ACOE2426 | France | Provence-Alpes-Côte- d'Azur | Alpes-Maritimes | Antibes | 43,564944 | 7,12555 | 15/05/2008 | Coeur d'Acier | Rosaceae | Prunus sp. L. |
| ACOE2429 | France | Provence-Alpes-Côte- d'Azur | Alpes-Maritimes | Antibes | 43,564944 | 7,12555 | 15/05/2008 | Coeur d'Acier | Poaceae | Poaceae sp. Barnhart |
| ACOE2430 | France | Provence-Alpes-Côte- d'Azur | Alpes-Maritimes | Antibes | 43,564944 | 7,12555 | 15/05/2008 | Coeur d'Acier | Euphorbiaceae | Euphorbia sp. L. |
| ACOE2431 | France | Provence-Alpes-Côte- d'Azur | Alpes-Maritimes | Antibes | 43,564944 | 7,12555 | 15/05/2008 | Coeur d'Acier | Gramineae | Phyllostachys sp. SIEBOLD & ZUCC. |
| ACOE2432 | France | Provence-Alpes-Côte- d'Azur | Alpes-Maritimes | Antibes | 43,564944 | 7,12555 | 15/05/2008 | Coeur d'Acier | Gramineae | Phyllostachys sp. SIEBOLD & ZUCC. |
| ACOE2433 | France | Provence-Alpes-Côte- d'Azur | Alpes-Maritimes | Antibes | 43,564944 | 7,12555 | 15/05/2008 | Coeur d'Acier | Asteraceae | Senecio vulgaris L. |
| ACOE2435 | France | Provence-Alpes-Côte- d'Azur | Alpes-Maritimes | Sophia Antipolis | 43,633221 | 7,062336 | 15/05/2008 | Coeur d'Acier | Ericaceae | Arbutus unedo L. |
| ACOE2437 | France | Provence-Alpes-Côte- d'Azur | Alpes-Maritimes | Sophia Antipolis | 43,633221 | 7,062336 | 15/05/2008 | Coeur d'Acier | Gramineae | Avena sp. L. |
| ACOE2438 | France | Poitou-Charentes | Charente-Maritime | Saint-Jean-de-Liversay | 46,269001 | -0,873422 | 19/05/2008 | Fauvin | Asteraceae | Helianthus annuus L. |
| ACOE2439 | France | Languedoc-Roussillon | Pyrénées-Orientales | Banyuls-sur-Mer | 42,483597 | 3,127978 | 22/05/2008 | Coeur d'Acier |  |  |
| ACOE2440 | France | Languedoc-Roussillon | Pyrénées-Orientales | Banyuls-sur-Mer | 42,483597 | 3,127978 | 22/05/2008 | Coeur d'Acier | Poaceae | Bambou sp. |
| ACOE2441 | France | Languedoc-Roussillon | Pyrénées-Orientales | Banyuls-sur-Mer | 42,46844 | 3,132173 | 22/05/2008 | Coeur d'Acier | Asteraceae | Asteraceae sp. |
| ACOE2442 | France | Languedoc-Roussillon | Pyrénées-Orientales | Banyuls-sur-Mer | 42,46844 | 3,132173 | 22/05/2008 | Coeur d'Acier | Fabaceae | Vicia sp. L. |
| ACOE2443 | France | Languedoc-Roussillon | Pyrénées-Orientales | Banyuls-sur-Mer | 42,469444 | 3,142222 | 22/05/2008 | Coeur d'Acier | Rosaceae | Prunus dulcis (Miller) D.A. Webb |
| ACOE2444 | France | Languedoc-Roussillon | Pyrénées-Orientales | Banyuls-sur-Mer | 42,46844 | 3,132173 | 22/05/2008 | Coeur d'Acier | Euphorbiaceae | Euphorbia characias L. |
| ACOE2445 | France | Languedoc-Roussillon | Pyrénées-Orientales | Banyuls-sur-Mer | 42,476633 | 3,114749 | 22/05/2008 | Coeur d'Acier | Brassicaceae | Brassicaceae sp. Burnett |
| ACOE2446 | France | Languedoc-Roussillon | Pyrénées-Orientales | Banyuls-sur-Mer | 42,469444 | 3,142222 | 22/05/2008 | Coeur d'Acier | Ulmaceae | Ulmus sp. L. |
| ACOE2447 | France | Languedoc-Roussillon | Pyrénées-Orientales | Banyuls-sur-Mer | 42,469444 | 3,142222 | 22/05/2008 | Coeur d'Acier | Verbenaceae | Vitex sp. |
| ACOE2448 | France | Languedoc-Roussillon | Pyrénées-Orientales | Banyuls-sur-Mer | 42,490165 | 3,129961 | 22/05/2008 | Coeur d'Acier | Asteraceae | Carthamus lanatus L. |
| ACOE2449 | France | Languedoc-Roussillon | Pyrénées-Orientales | Banyuls-sur-Mer | 42,490165 | 3,129961 | 22/05/2008 | Coeur d'Acier | Asteraceae | Asteraceae sp. |
| ACOE2451 | France | Languedoc-Roussillon | Pyrénées-Orientales | Banyuls-sur-Mer | 42,490165 | 3,129961 | 22/05/2008 | Coeur d'Acier | Brassicaceae | Brassicaceae sp. Burnett |
| ACOE2452 | France | Languedoc-Roussillon | Pyrénées-Orientales | Banyuls-sur-Mer | 42,490165 | 3,129961 | 22/05/2008 | Coeur d'Acier | Gramineae | Avena sp. L. |
| ACOE2453 | France | Languedoc-Roussillon | Pyrénées-Orientales | Banyuls-sur-Mer | 42,490165 | 3,129961 | 22/05/2008 | Coeur d'Acier | Asteraceae | Senecio sp. L. |
| ACOE2454 | France | Languedoc-Roussillon | Pyrénées-Orientales | Banyuls-sur-Mer | 42,490165 | 3,129961 | 22/05/2008 | Coeur d'Acier | Malvaceae | Malvaceae sp. |
| ACOE2455 | France | Languedoc-Roussillon | Pyrénées-Orientales | Banyuls-sur-Mer | 42,490165 | 3,129961 | 22/05/2008 | Coeur d'Acier | Asteraceae | Helichrysum sp. P. Miller |
| ACOE2456 | France | Languedoc-Roussillon | Pyrénées-Orientales | Banyuls-sur-Mer | 42,494641 | 3,128663 | 22/05/2008 | Coeur d'Acier | Apiaceae | Foeniculum vulgare Miller |
| ACOE2457 | France | Languedoc-Roussillon | Pyrénées-Orientales | Collioure | 42,524674 | 3,080349 | 23/05/2008 | Coeur d'Acier | Polygonaceae | Polygonum aviculare L. |
| ACOE2458 | France | Languedoc-Roussillon | Pyrénées-Orientales | Collioure | 42,524674 | 3,080349 | 23/05/2008 | Coeur d'Acier | Asteraceae | Calendula sp. L. |
| ACOE2459 | France | Languedoc-Roussillon | Pyrénées-Orientales | Collioure | 42,524674 | 3,080349 | 23/05/2008 | Coeur d'Acier | Poaceae | Arundo donax L. |
| ACOE2460 | France | Languedoc-Roussillon | Pyrénées-Orientales | Collioure | 42,524674 | 3,080349 | 23/05/2008 | Coeur d'Acier | Cyperaceae | Cyperus sp. L. |
| ACOE2461 | France | Languedoc-Roussillon | Pyrénées-Orientales | Collioure | 42,524674 | 3,080349 | 23/05/2008 | Coeur d'Acier | Asteraceae | Chrysanthemum sp. L. |
| ACOE2463 | France | Bourgogne | Côte-d'Or | Saint-Usage | 47,1096 | 5,261878 | 15/05/2008 | Coeur d'Acier | Asteraceae | Helianthus annuus L. |
| ACOE2464 | France | Lorraine | Meuse | Thillot | 49,0284 | 5,668678 | 15/05/2008 | Coeur d'Acier | Rosaceae | Prunus insititia |
| ACOE2465 | France | Lorraine | Meuse |  | 48,983334 | 5,716667 | 15/05/2008 | Coeur d'Acier | Rosaceae | Prunus insititia |
| ACOE2466 | France | Bourgogne | Côte-d'Or | Saint-Didier | 47,330101 | 4,181878 | 13/05/2008 | Houis | Pinaceae | Abies nordmanniana (STEVEN) SPACH |
| ACOE2467 | France | Bourgogne | Côte-d'Or | Saint-Didier | 47,330101 | 4,181878 | 06/05/2008 | Houis | Pinaceae | Picea abies (L.) Karsten |
| ACOE2468 | France | Centre | Loiret | Beaugency | 47,772015 | 1,654737 | 23/05/2008 | Quartier | Asteraceae | Helianthus annuus L. |
| ACOE2469 | France | Lorraine | Meurthe-et-Moselle | Morville-sur-Seille | 48,915901 | 6,156477 | 26/05/2008 | CETIOM | Asteraceae | Helianthus annuus L. |
| ACOE2470 | France | Rhône-Alpes | Rhône | Lyon | 45,730689 | 5,067616 | 27/05/2008 | CETIOM | Asteraceae | Helianthus annuus L. |
| ACOE2471 | France | Languedoc-Roussillon | Pyrénées-Orientales | Collioure | 42,525898 | 3,083878 | 23/05/2008 | Coeur d'Acier |  |  |
| ACOE2472 | France | Languedoc-Roussillon | Gard | Gaujac | 44,09049 | 4,563467 | 31/05/2008 | Coeur d'Acier | Rosaceae | Malus domestica Borckh. |
| ACOE2473 | France | Languedoc-Roussillon | Gard | Gaujac | 44,09049 | 4,563467 | 31/05/2008 | Coeur d'Acier | Rosaceae | Prunus persica (L.) Batsch |
| ACOE2474 | France | Auvergne | Allier | Biozat | 46,077297 | 3,268378 | 28/05/2008 | Forunier | Asteraceae | Helianthus annuus L. |
| ACOE2476 | France | Champagne-Ardenne | Aube | Saint-Pouange | 48,226498 | 4,040577 | 29/05/2008 | Piccom | Asteraceae | Helianthus annuus L. |
| ACOE2477 | France | Aquitaine | Lot-et-Garonne | Tonneins | 44,390701 | 0,310078 | 29/05/2008 | Segura | Asteraceae | Helianthus annuus L. |
| ACOE2478 | France | Languedoc-Roussillon | Hérault | Gignac | 43,623423 | 3,62097 | 12/06/2008 | Coeur d'Acier | Apiaceae | Bupleurum fruticosum L. |
| ACOE2480 | France | Languedoc-Roussillon | Hérault | La Boissiere | 43,646111 | 3,6375 | 12/06/2008 | Coeur d'Acier | Leguminosae | Melilotus sp. P. Miller |
| ACOE2481 | France | Languedoc-Roussillon | Hérault | Aniane | 43,674674 | 3,60686 | 12/06/2008 | Coeur d'Acier | Rosaceae | Prunus mahaleb L. |
| ACOE2482 | France | Languedoc-Roussillon | Hérault | Saint-Guilhem-le-Desert | 43,744167 | 3,563333 | 12/06/2008 | Coeur d'Acier | Ranunculaceae | Clematis vitalba L. |
| ACOE2483 | France | Languedoc-Roussillon | Hérault | Saint-Guilhem-le-Desert | 43,744167 | 3,563333 | 12/06/2008 | Coeur d'Acier | Oenotheraceae | Oenothera sp. L. |
| ACOE2486 | France | Basse-Normandie | Orne | Vrigny | 48,6693 | -0,022922 | 05/06/2008 | Ballanger | Asteraceae | Helianthus annuus L. |
| ACOE2487 | France | Pays-de-la-Loire | Sarthe | Fresnay-sur-Sarthe | 48,2841 | 0,018478 | 05/06/2008 | Ballanger | Asteraceae | Helianthus annuus L. |
| ACOE2602 | France | Rhône-Alpes | Savoie | Bessans | 45,309167 | 7,053333 | 22/07/2008 | Coeur d'Acier | Cistaceae | Helianthemum sp. P. Miller |
| ACOE2603 | France | Rhône-Alpes | Savoie | Bessans | 45,309167 | 7,053333 | 22/07/2008 | Coeur d'Acier | Scrophulariaceae | Rhinanthus minor L. |
| ACOE2604 | France | Rhône-Alpes | Savoie | Bessans | 45,309167 | 7,053333 | 22/07/2008 | Coeur d'Acier | Caryophyllaceae | Silene sp. L. |
| ACOE2611 | France | Rhône-Alpes | Savoie | Lanslebourg-Mont-Cenis | 45,276006 | 6,907521 | 23/07/2008 | Coeur d'Acier | Salicaceae | Salix sp. L. |
| ACOE2612 | France | Rhône-Alpes | Savoie | Lanslebourg-Mont-Cenis | 45,276006 | 6,907521 | 23/07/2008 | Coeur d'Acier | Pinaceae | Larix decidua Miller |
| ACOE2614 | France | Rhône-Alpes | Savoie | Bessans | 45,321034 | 6,992121 | 23/07/2008 | Coeur d'Acier | Salicaceae | Salix sp. L. |
| ACOE2615 | France | Rhône-Alpes | Savoie | Bessans | 45,321034 | 6,992121 | 23/07/2008 | Coeur d'Acier | Salicaceae | Salix sp. L. |
| ACOE2638 | Serbia |  |  |  | 44,833332 | 20,4 | 15/07/2008 | Petrovic O. | Rosaceae | Chaenomeles speciosa (Sweet) Nakai |
| ACOE2639 | Serbia |  |  |  | 43,933334 | 20,633333 | 14/07/2008 | Petrovic O. | Rosaceae | Malus domestica Borckh. |
| ACOE2640 | Serbia |  |  |  | 44,983334 | 20,166666 | 15/07/2008 | Petrovic O. | Rosaceae | Malus domestica Borckh. |
| ACOE2641 | Serbia |  |  |  | 44,833332 | 20,4 | 16/07/2008 | Petrovic O. | Rosaceae | Malus domestica Borckh. |
| ACOE2642 | Serbia |  |  |  | 44,216667 | 20,299999 | 07/09/2008 | Petrovic O. | Rosaceae | Cydonia oblonga Miller |
