# Supplementary material for: DNA Barcoding and the Associated PhylAphidB@se Website for the Identification of European Aphids (Insecta: Hemiptera: Aphididae)
Source: PLoS One. 2014 Jun 4;9(6):e97620. doi: 10.1371/journal.pone.0097620 (PMC4045754; doi:10.1371/journal.pone.0097620)
Supplement: Table S2 — Identification and taxonomic data. Identification and taxonomic data for the 1020 specimens used in the study. (DOCX) [file pone.0097620.s003.docx]

| ID Voucher | Familly | Sub-Familly | Genus | Species | Descriptor | Identified by | Identification year |
| --- | --- | --- | --- | --- | --- | --- | --- |
| ACOE365 | Aphididae | Aphidinae | *Cavariella* | *aegopodii* | (Scopoli, 1763) | Coeur d'Acier | 1999 |
| ACOE366 | Aphididae | Aphidinae | *Hyperomyzus* | *lactucae* | (Linnaeus, 1758) | Coeur d'Acier | 1999 |
| ACOE372 | Aphididae | Aphidinae | *Myzus* | *varians* | Davidson, 1912 | Coeur d'Acier | 1999 |
| ACOE383 | Aphididae | Aphidinae | *Cavariella* | *theobaldi* | (Gillette & Bragg, 1918) | Coeur d'Acier | 2007 |
| ACOE384 | Aphididae | Calaphidinae | *Chromaphis* | *juglandicola* | (Kaltenbach,1843) | Coeur d'Acier | 2007 |
| ACOE385 | Aphididae | Aphidinae | *Macrosiphum* | *albifrons* | Essig, 1911 | Coeur d'Acier | 1999 |
| ACOE386 | Aphididae | Aphidinae | *Phorodon* | *humuli* | (Schrank, 1801) | Coeur d'Acier | 2007 |
| ACOE387 | Aphididae | Calaphidinae | *Eucallipterus* | *tiliae* | (Linnaeus, 1758) | Coeur d'Acier | 1999 |
| ACOE388 | Aphididae | Aphidinae | *Cavariella* | *theobaldi* | (Gillette & Bragg, 1918) | Coeur d'Acier | 1999 |
| ACOE389 | Aphididae | Aphidinae | *Cavariella* | *theobaldi* | (Gillette & Bragg, 1918) | Coeur d'Acier | 1999 |
| ACOE391 | Aphididae | Eriosomatinae | *Eriosoma* | *lanuginosum* | (Hartig, 1839) | Coeur d'Acier | 1999 |
| ACOE392 | Aphididae | Aphidinae | *Megoura* | *viciae* | Buckton, 1876 | Coeur d'Acier | 1999 |
| ACOE393 | Aphididae | Aphidinae | *Amphorophora* | *rubi* | (Kaltenbach, 1843) | Coeur d'Acier | 1999 |
| ACOE394 | Aphididae | Aphidinae | *Brachycaudus* | *cardui* | (Linnaeus, 1860) | Coeur d'Acier | 1999 |
| ACOE395 | Aphididae | Aphidinae | *Myzus* | *lythri* | (Schrank, 1801) | Coeur d'Acier | 2007 |
| ACOE398 | Aphididae | Calaphidinae | *Myzocallis* | *coryli* | (Goeze, 1778) | Coeur d'Acier | 1999 |
| ACOE399 | Aphididae | Aphidinae | *Brachycaudus* | *cardui* | (Linnaeus, 1860) | Coeur d'Acier | 1999 |
| ACOE402 | Aphididae | Aphidinae | *Ovatus* | *crataegarius* | (Walker, 1850) | Coeur d'Acier | 1999 |
| ACOE403 | Aphididae | Calaphidinae | *Callipterinella* | *tuberculata* | (von Heyden, 1837) | Coeur d'Acier | 1999 |
| ACOE409 | Aphididae | Aphidinae | *Semiaphis* | *dauci* | (Fabricius, 1775) | Coeur d'Acier | 2007 |
| ACOE410 | Aphididae | Aphidinae | *Macrosiphum* | *cholodkovskyi* | (Mordvilko, 1909) | Coeur d'Acier | 1999 |
| ACOE411 | Aphididae | Aphidinae | *Myzus* | *lythri* | (Schrank, 1801) | Coeur d'Acier | 2007 |
| ACOE412 | Aphididae | Aphidinae | *Myzus* | *lythri* | (Schrank, 1801) | Coeur d'Acier | 2007 |
| ACOE413 | Aphididae | Thelaxinae | *Thelaxes* | *dryophila* | (Schrank, 1801) | Coeur d'Acier | 1999 |
| ACOE416 | Aphididae | Aphidinae | *Aphis* | *grossulariae* | Kaltenbach, 1843 | Coeur d'Acier | 1999 |
| ACOE418 | Aphididae | Aphidinae | *Aphis* | *ulmariae* | Schrank, 1801 | Coeur d'Acier | 1999 |
| ACOE424 | Aphididae | Aphidinae | *Brachycaudus* | *cardui* | (Linnaeus, 1860) | Coeur d'Acier | 1999 |
| ACOE425 | Aphididae | Aphidinae | *Metopeurum* | *fuscoviride* | Stroyan, 1950 | Coeur d'Acier | 1999 |
| ACOE428 | Aphididae | Eriosomatinae | *Thecabius* | *affinis* | (Kaltenbach, 1843) | Coeur d'Acier | 1999 |
| ACOE430 | Aphididae | Aphidinae | *Macrosiphoniella* | *absinthii* | (Linnaeus, 1758) | Coeur d'Acier | 1999 |
| ACOE432 | Aphididae | Aphidinae | *Aphis* | *verbasci* | Schrank, 1801 | Coeur d'Acier | 1999 |
| ACOE433 | Aphididae | Aphidinae | *Aphis* | *farinosa* | Gmelin, 1790 | Coeur d'Acier | 1999 |
| ACOE434 | Aphididae | Aphidinae | *Brachycaudus* | *cardui* | (Linnaeus, 1860) | Coeur d'Acier | 1999 |
| ACOE437 | Aphididae | Aphidinae | *Nasonovia* | *ribisnigri* | (Mosley, 1841) | Coeur d'Acier | 1999 |
| ACOE438 | Aphididae | Aphidinae | *Anuraphis* | *shaposhnikovi* | Barbagallo & Cocuzza, 2003 | Coeur d'Acier | 2007 |
| ACOE439 | Aphididae | Aphidinae | *Aphis* | *verbasci* | Schrank, 1801 | Coeur d'Acier | 1999 |
| ACOE442 | Aphididae | Aphidinae | *Aphis* | *epilobii* | Kaltenbach, 1843 | Coeur d'Acier | 1999 |
| ACOE443 | Aphididae | Aphidinae | *Aphis* | *epilobii* | Kaltenbach, 1843 | Coeur d'Acier | 1999 |
| ACOE445 | Aphididae | Lachninae | *Cinara* | *laricis* | (Hartig, 1839) | Coeur d'Acier | 1999 |
| ACOE446 | Aphididae | Lachninae | *Cinara* | *pectinatae* | (Nördlinger, 1880) | Coeur d'Acier | 1999 |
| ACOE448 | Aphididae | Aphidinae | *Rhopalosiphum* | *insertum* | (Walker, 1849) | Coeur d'Acier | 1999 |
| ACOE449 | Aphididae | Aphidinae | *Uroleucon* | *hypochoeridis* | (Fabricius, 1779) | Coeur d'Acier | 1999 |
| ACOE450 | Aphididae | Aphidinae | *Uroleucon* | *jaceae* | (Linnaeus, 1758) | Coeur d'Acier | 1999 |
| ACOE453 | Aphididae | Aphidinae | *Melanaphis* | *luzulella* | (Hille Ris Lambers, 1947) | Coeur d'Acier | 1999 |
| ACOE454 | Aphididae | Aphidinae | *Uroleucon* | *solidaginis* | (Fabricius, 1779) | Coeur d'Acier | 1999 |
| ACOE455 | Aphididae | Aphidinae | *Aphis* | *teucrii* | (Börner, 1942) | Coeur d'Acier | 1999 |
| ACOE456 | Aphididae | Aphidinae | *Macrosiphum* | *stellariae* | Theobald, 1913 | Coeur d'Acier | 1999 |
| ACOE458 | Aphididae | Aphidinae | *Aphis* | *salicariae* | Koch, 1855 | Coeur d'Acier | 1999 |
| ACOE459 | Aphididae | Eriosomatinae | *Baizongia* | *pistaciae* | (Linnaeus, 1767) | Coeur d'Acier | 1999 |
| ACOE460 | Aphididae | Aphidinae | *Aphis* | *fabae* | Scopoli, 1763 | Coeur d'Acier | 1999 |
| ACOE461 | Aphididae | Aphidinae | *Aphis* | *verbasci* | Schrank, 1801 | Coeur d'Acier | 1999 |
| ACOE462 | Aphididae | Aphidinae | *Cavariella* | *theobaldi* | (Gillette & Bragg, 1918) | Coeur d'Acier | 1999 |
| ACOE463 | Aphididae | Lachninae | *Cinara* | *brauni* | Börner, 1940 | Coeur d'Acier | 1999 |
| ACOE464 | Aphididae | Aphidinae | *Uroleucon* | *picridis* | (Fabricius, 1775) | Coeur d'Acier | 1999 |
| ACOE465 | Aphididae | Aphidinae | *Uroleucon* | *sonchi* | (Linnaeus, 1767) | Coeur d'Acier | 1999 |
| ACOE466 | Aphididae | Aphidinae | *Macrosiphoniella* | *abrotani* | (Walker, 1852) | Coeur d'Acier | 1999 |
| ACOE467 | Aphididae | Aphidinae | *Uroleucon* | *jaceae* | (Linnaeus, 1758) | Coeur d'Acier | 1999 |
| ACOE470 | Aphididae | Aphidinae | *Brevicoryne* | *brassicae* | (Linnaeus, 1758) | Coeur d'Acier | 1999 |
| ACOE475 | Aphididae | Aphidinae | *Hyadaphis* | *foeniculi* | (Passerini, 1860) | Coeur d'Acier | 2007 |
| ACOE477 | Aphididae | Aphidinae | *Dysaphis* | *plantaginea* | (Passerini, 1860) | Coeur d'Acier | 2007 |
| ACOE482 | Aphididae | Aphidinae | *Aphis* | *althaeae* | (Nevsky,1929) | Coeur d'Acier | 1999 |
| ACOE483 | Aphididae | Aphidinae | *Aphis* | *hederae* | Kaltenbach, 1843 | Coeur d'Acier | 1999 |
| ACOE495 | Aphididae | Aphidinae | *Ceruraphis* | *eriophori* | (Walker, 1848) | Coeur d'Acier | 1999 |
| ACOE504 | Aphididae | Aphidinae | *Ceruraphis* | *eriophori* | (Walker, 1848) | Coeur d'Acier | 1999 |
| ACOE506 | Aphididae | Aphidinae | *Aphis* | *fabae* | Linnaeus, 1758 | Coeur d'Acier | 1999 |
| ACOE507 | Aphididae | Aphidinae | *Aphis* | *sambuci* | Linnaeus, 1758 | Coeur d'Acier | 1999 |
| ACOE509 | Aphididae | Aphidinae | *Aphis* | *hederae* | Kaltenbach, 1843 | Coeur d'Acier | 1999 |
| ACOE510 | Aphididae | Aphidinae | *Aphis* | *fabae* | Scopoli, 1763 | Coeur d'Acier | 1999 |
| ACOE511 | Aphididae | Aphidinae | *Aphis* | *fabae* | Scopoli, 1763 | Coeur d'Acier | 1999 |
| ACOE512 | Aphididae | Aphidinae | *Aphis* | *fabae* | Scopoli, 1763 | Coeur d'Acier | 1999 |
| ACOE513 | Aphididae | Aphidinae | *Aphis* | *fabae* | Scopoli, 1763 | Coeur d'Acier | 1999 |
| ACOE514 | Aphididae | Aphidinae | *Aphis* | *fabae* | Scopoli, 1763 | Coeur d'Acier | 1999 |
| ACOE515 | Aphididae | Aphidinae | *Aphis* | *fabae* | Scopoli, 1763 | Coeur d'Acier | 1999 |
| ACOE516 | Aphididae | Aphidinae | *Aphis* | *fabae* | Scopoli, 1763 | Coeur d'Acier | 1999 |
| ACOE517 | Aphididae | Aphidinae | *Aphis* | *fabae* | Scopoli, 1763 | Coeur d'Acier | 2000 |
| ACOE518 | Aphididae | Aphidinae | *Aphis* | *fabae* | Scopoli, 1763 | Coeur d'Acier | 1999 |
| ACOE519 | Aphididae | Aphidinae | *Aphis* | *fabae* | Scopoli, 1763 | Coeur d'Acier | 2000 |
| ACOE520 | Aphididae | Aphidinae | *Aphis* | *fabae* | Scopoli, 1763 | Coeur d'Acier | 1999 |
| ACOE521 | Aphididae | Aphidinae | *Aphis* | *fabae* | Scopoli, 1763 | Coeur d'Acier | 1999 |
| ACOE522 | Aphididae | Aphidinae | *Aphis* | *fabae* | Scopoli, 1763 | Coeur d'Acier | 1999 |
| ACOE523 | Aphididae | Aphidinae | *Aphis* | *fabae* | Scopoli, 1763 | Coeur d'Acier | 1999 |
| ACOE525 | Aphididae | Aphidinae | *Aphis* | *fabae* | Scopoli, 1763 | Coeur d'Acier | 1999 |
| ACOE527 | Aphididae | Aphidinae | *Aphis* | *newtoni* | Theobald, 1927 | Coeur d'Acier | 1999 |
| ACOE533 | Aphididae | Aphidinae | *Aphis* | *fabae* | Scopoli, 1763 | Coeur d'Acier | 1999 |
| ACOE534 | Aphididae | Aphidinae | *Aphis* | *fabae* | Scopoli, 1763 | Coeur d'Acier | 1999 |
| ACOE535 | Aphididae | Aphidinae | *Aphis* | *fabae* | Scopoli, 1763 | Coeur d'Acier | 1999 |
| ACOE539 | Aphididae | Aphidinae | *Aphis* | *fabae* | Scopoli, 1763 | Coeur d'Acier | 1999 |
| ACOE540 | Aphididae | Aphidinae | *Aphis* | *rumicis* | Linnaeus, 1758 | Coeur d'Acier | 1999 |
| ACOE542 | Aphididae | Aphidinae | *Aphis* | *fabae* | Scopoli, 1763 | Coeur d'Acier | 1999 |
| ACOE547 | Aphididae | Aphidinae | *Aphis* | *fabae* | Scopoli, 1763 | Coeur d'Acier | 1999 |
| ACOE548 | Aphididae | Aphidinae | *Aphis* | *fabae* | Scopoli, 1763 | Coeur d'Acier | 1999 |
| ACOE549 | Aphididae | Aphidinae | *Aphis* | *fabae* | Scopoli, 1763 | Coeur d'Acier | 1999 |
| ACOE552 | Aphididae | Aphidinae | *Aphis* | *fabae* | Scopoli, 1763 | Coeur d'Acier | 1999 |
| ACOE555 | Aphididae | Aphidinae | *Aphis* | *viburni* | Scopoli, 1763 | Coeur d'Acier | 1999 |
| ACOE556 | Aphididae | Aphidinae | *Aphis* | *fabae* | Scopoli, 1763 | Coeur d'Acier | 1999 |
| ACOE557 | Aphididae | Aphidinae | *Aphis* | *fabae* | Scopoli, 1763 | Coeur d'Acier | 1999 |
| ACOE558 | Aphididae | Aphidinae | *Aphis* | *fabae* | Scopoli, 1763 | Coeur d'Acier | 1999 |
| ACOE559 | Aphididae | Aphidinae | *Aphis* | *fabae* | Scopoli, 1763 | Coeur d'Acier | 1999 |
| ACOE560 | Aphididae | Aphidinae | *Aphis* | *fabae* | Scopoli, 1763 | Coeur d'Acier | 1999 |
| ACOE565 | Aphididae | Aphidinae | *Aphis* | *veratri* | Walker, 1852 | Coeur d'Acier | 1999 |
| ACOE566 | Aphididae | Aphidinae | *Aphis* | *fabae* | Scopoli, 1763 | Coeur d'Acier | 2000 |
| ACOE567 | Aphididae | Aphidinae | *Aphis* | *sp.* | Linnaeus, 1758 | Coeur d'Acier | 1999 |
| ACOE570 | Aphididae | Chaitophorinae | *Periphyllus* | *testudinaceus* | (Fernie, 1852) | Coeur d'Acier | 2007 |
| ACOE571 | Aphididae | Aphidinae | *Ovatus* | *crataegarius* | (Walker, 1850) | Coeur d'Acier | 2007 |
| ACOE572 | Aphididae | Aphidinae | *Aphis* | *clematidis* | Linnaeus, 1758 | Coeur d'Acier | 1999 |
| ACOE573 | Aphididae | Aphidinae | *Macrosiphoniella* | *helichrysi* | Remaudière, 1952 | Coeur d'Acier | 1999 |
| ACOE574 | Aphididae | Aphidinae | *Uroleucon* | *jaceae* | (Linnaeus, 1758) | Coeur d'Acier | 1999 |
| ACOE575 | Aphididae | Aphidinae | *Ephedraphis* | *ephedrae* | (Nevsky, 1929) | Coeur d'Acier | 1999 |
| ACOE576 | Aphididae | Aphidinae | *Melanaphis* | *donacis* | (Passerini, 1862) | Coeur d'Acier | 1999 |
| ACOE577 | Aphididae | Anoeciinae | *Anoecia* | *corni* | (Fabricius, 1775) | Coeur d'Acier | 1999 |
| ACOE578 | Aphididae | Aphidinae | *Aphis* | *sanguisorbae* | Schrank, 1801 | Coeur d'Acier | 1999 |
| ACOE579 | Aphididae | Aphidinae | *Aphis* | *sanguisorbae* | Schrank, 1801 | Coeur d'Acier | 1999 |
| ACOE580 | Aphididae | Aphidinae | *Aphis* | *fabae* | Scopoli, 1763 | Coeur d'Acier | 1999 |
| ACOE581 | Aphididae | Chaitophorinae | *Chaitophorus* | *leucomelas* | Koch, 1854 | Coeur d'Acier | 1999 |
| ACOE582 | Aphididae | Pterocommatinae | *Pterocomma* | *populeum* | (Kaltenbach, 1843) | Coeur d'Acier | 1999 |
| ACOE583 | Aphididae | Aphidinae | *Acyrthosiphon* | *pisum* | (Harris, 1776) | Coeur d'Acier | 1999 |
| ACOE585 | Aphididae | Drepanosiphinae | *Drepanosiphum* | *sp.* | Koch, 1855 | Coeur d'Acier | 1999 |
| ACOE587 | Aphididae | Aphidinae | *Brachycaudus* | *tragopogonis* | (Kaltenbach, 1843) | Coeur d'Acier | 1999 |
| ACOE588 | Aphididae | Aphidinae | *Protaphis* | *terricola* | (Rondani, 1848) | Coeur d'Acier | 2007 |
| ACOE589 | Aphididae | Aphidinae | *Protaphis* | *terricola* | (Rondani, 1848) | Coeur d'Acier | 1999 |
| ACOE590 | Aphididae | Aphidinae | *Uroleucon* | *jaceae* | (Linnaeus, 1758) | Coeur d'Acier | 1999 |
| ACOE592 | Aphididae | Aphidinae | *Brachyunguis* | *tamaricis* | (Lichtenstein, 1885) | Coeur d'Acier | 1999 |
| ACOE593 | Aphididae | Chaitophorinae | *Periphyllus* | *bulgaricus* | Tashev, 1964 | Coeur d'Acier | 1999 |
| ACOE595 | Aphididae | Aphidinae | *Myzus* | *lythri* | (Schrank, 1801) | Coeur d'Acier | 1999 |
| ACOE596 | Aphididae | Aphidinae | *Aphis* | *fabae* | Scopoli, 1763 | Coeur d'Acier | 1999 |
| ACOE599 | Aphididae | Aphidinae | *Dysaphis* | *radicola* | (Mordvilko, 1897) | Coeur d'Acier | 1999 |
| ACOE601 | Aphididae | Aphidinae | *Hyperomyzus* | *lactucae* | (Linnaeus, 1758) | Coeur d'Acier | 1999 |
| ACOE602 | Aphididae | Aphidinae | *Uroleucon* | *sonchi* | (Linnaeus, 1767) | Coeur d'Acier | 1999 |
| ACOE603 | Aphididae | Aphidinae | *Aphis* | *fabae* | Scopoli, 1763 | Coeur d'Acier | 1999 |
| ACOE604 | Aphididae | Aphidinae | *Aphis* | *spiraecola* | Patch, 1914 | Coeur d'Acier | 1999 |
| ACOE605 | Aphididae | Aphidinae | *Aulacorthum* | *solani* | (Kaltenbach, 1843) | Coeur d'Acier | 2002 |
| ACOE607 | Aphididae | Aphidinae | *Microlophium* | *carnosum* | (Buckton, 1876) | Coeur d'Acier | 1999 |
| ACOE609 | Aphididae | Aphidinae | *Aphis* | *ruborum* | (Börner, 1932) | Coeur d'Acier | 1999 |
| ACOE610 | Aphididae | Aphidinae | *Aphis* | *fabae* | Scopoli, 1763 | Coeur d'Acier | 1999 |
| ACOE611 | Aphididae | Aphidinae | *Macrosiphum* | *rosae* | (Linnaeus, 1758) | Coeur d'Acier | 1999 |
| ACOE612 | Aphididae | Aphidinae | *Megourella* | *tribulis* | (Walker, 1849) | Coeur d'Acier | 1999 |
| ACOE613 | Aphididae | Aphidinae | *Macrosiphoniella* | *oblonga* | (Mordvilko, 1901) | Coeur d'Acier | 1999 |
| ACOE614 | Aphididae | Aphidinae | *Uroleucon* | *sp.* | Mordvilko, 1914 | Coeur d'Acier | 1999 |
| ACOE615 | Aphididae | Aphidinae | *Dysaphis* | *aucupariae* | (Buckton, 1879) | Coeur d'Acier | 1999 |
| ACOE616 | Aphididae | Aphidinae | *Aphis* | *fabae* | Scopoli, 1763 | Coeur d'Acier | 1999 |
| ACOE617 | Aphididae | Aphidinae | *Aphis* | *craccivora* | Koch, 1854 | Coeur d'Acier | 1999 |
| ACOE618 | Aphididae | Aphidinae | *Uroleucon* | *jaceae* | (Linnaeus, 1758) | Coeur d'Acier | 1999 |
| ACOE619 | Aphididae | Aphidinae | *Aphis* | *gossypii* | Glover, 1877 | Coeur d'Acier | 2002 |
| ACOE620 | Aphididae | Aphidinae | *Uroleucon* | *nigrocampanulae* | (Theobald, 1928) | Coeur d'Acier | 1999 |
| ACOE621 | Aphididae | Aphidinae | *Aphis* | *aliena* | Theobald, 1915 | Coeur d'Acier | 1999 |
| ACOE622 | Aphididae | Aphidinae | *Aphis* | *taraxacicola* | (Börner, 1940) | Coeur d'Acier | 1999 |
| ACOE623 | Aphididae | Aphidinae | *Uroleucon* | *hypochoeridis* | (Fabricius, 1779) | Coeur d'Acier | 2007 |
| ACOE625 | Aphididae | Aphidinae | *Aphis* | *urticata* | Gmelin, 1790 | Coeur d'Acier | 1999 |
| ACOE627 | Aphididae | Aphidinae | *Macrosiphum* | *rosae* | (Linnaeus, 1758) | Coeur d'Acier | 1999 |
| ACOE629 | Aphididae | Aphidinae | *Brachycaudus* | *tragopogonis* | (Kaltenbach, 1843) | Coeur d'Acier | 1999 |
| ACOE630 | Aphididae | Aphidinae | *Brachycaudus* | *cardui* | (Linnaeus, 1860) | Coeur d'Acier | 1999 |
| ACOE631 | Aphididae | Aphidinae | *Uroleucon* | *hypochoeridis* | (Fabricius, 1779) | Coeur d'Acier | 2007 |
| ACOE632 | Aphididae | Pterocommatinae | *Pterocomma* | *pilosum* | Buckton, 1879 | Coeur d'Acier | 1999 |
| ACOE633 | Aphididae | Lachninae | *Cinara* | *pilicornis* | (Hartig, 1841) | Coeur d'Acier | 1999 |
| ACOE635 | Aphididae | Aphidinae | *Aphis* | *taraxacicola* | (Börner, 1940) | Coeur d'Acier | 1999 |
| ACOE636 | Aphididae | Aphidinae | *Hyalopterus* | *pruni* | (Geoffroy, 1762) | Coeur d'Acier | 1999 |
| ACOE637 | Aphididae | Aphidinae | *Aphis* | *ruborum* | Linnaeus, 1758 | Coeur d'Acier | 1999 |
| ACOE638 | Aphididae | Aphidinae | *Brachycaudus* | *cardui* | (Linnaeus, 1860) | Coeur d'Acier | 1999 |
| ACOE639 | Aphididae | Aphidinae | *Myzus* | *lythri* | (Schrank, 1801) | Coeur d'Acier | 1999 |
| ACOE640 | Aphididae | Lachninae | *Cinara* | *juniperi* | (de Geer, 1773) | Coeur d'Acier | 1999 |
| ACOE641 | Aphididae | Aphidinae | *Aphis* | *salviae* | Walker, 1852 | Coeur d'Acier | 1999 |
| ACOE642 | Aphididae | Aphidinae | *Dysaphis* | *apiifolia* | (Theobald, 1923) | Coeur d'Acier | 1999 |
| ACOE644 | Aphididae | Aphidinae | *Brachycaudus* | *lychnidis* | (Linnaeus, 1758) | Coeur d'Acier | 1999 |
| ACOE645 | Aphididae | Aphidinae | *Anuraphis* | *subterranea* | (Walker, 1852) | Coeur d'Acier | 1999 |
| ACOE646 | Aphididae | Aphidinae | *Hyalopterus* | *pruni* | (Geoffroy, 1762) | Coeur d'Acier | 1999 |
| ACOE648 | Aphididae | Aphidinae | *Pleotrichophorus* | *glandulosus* | (Kaltenbach, 1846) | Coeur d'Acier | 1999 |
| ACOE649 | Aphididae | Aphidinae | *Brachycaudus* | *lychnidis* | (Linnaeus, 1758) | Coeur d'Acier | 1999 |
| ACOE651 | Aphididae | Aphidinae | *Uroleucon* | *hypochoeridis* | (Fabricius, 1779) | Coeur d'Acier | 1999 |
| ACOE654 | Aphididae | Eriosomatinae | *Thecabius* | *affinis* | (Kaltenbach, 1843) | Coeur d'Acier | 1999 |
| ACOE655 | Aphididae | Eriosomatinae | *Pemphigus* | *vesicarius* | Passerini, 1861 | Coeur d'Acier | 2007 |
| ACOE656 | Aphididae | Eriosomatinae | *Pemphigus* | *immunis* | Buckton, 1896 | Coeur d'Acier | 1999 |
| ACOE658 | Aphididae | Aphidinae | *Aphis* | *craccivora* | Koch, 1854 | Coeur d'Acier | 1999 |
| ACOE659 | Aphididae | Aphidinae | *Aphis* | *fabae* | Scopoli, 1763 | Coeur d'Acier | 1999 |
| ACOE661 | Aphididae | Lachninae | *Cinara* | *pruinosa* | (Hartig, 1841) | Coeur d'Acier | 1999 |
| ACOE662 | Aphididae | Aphidinae | *Aphis* | *fabae* | Scopoli, 1763 | Coeur d'Acier | 1999 |
| ACOE665 | Aphididae | Aphidinae | *Aphis* | *umbrella* | (Börner, 1950) | Coeur d'Acier | 1999 |
| ACOE666 | Aphididae | Aphidinae | *Aphis* | *sambuci* | Linnaeus, 1758 | Coeur d'Acier | 1999 |
| ACOE668 | Aphididae | Aphidinae | *Aphis* | *crepidis* | (Börner, 1940) | Coeur d'Acier | 1999 |
| ACOE669 | Aphididae | Aphidinae | *Aphis* | *gossypii* | Walker, 1849 | Coeur d'Acier | 1999 |
| ACOE671 | Aphididae | Aphidinae | *Aphis* | *sp.frangulae-like* |  | Coeur d'Acier | 1999 |
| ACOE673 | Aphididae | Aphidinae | *Aphis* | *tirucallis* | Hille Ris Lambers, 1954 | Coeur d'Acier | 1999 |
| ACOE674 | Aphididae | Pterocommatinae | *Pterocomma* | *populeum* | (Kaltenbach, 1843) | Coeur d'Acier | 1999 |
| ACOE675 | Aphididae | Calaphidinae | *Panaphis* | *juglandis* | (Goeze, 1778) | Coeur d'Acier | 1999 |
| ACOE677 | Aphididae | Aphidinae | *Aphis* | *spiraecola* | Patch, 1914 | Coeur d'Acier | 1999 |
| ACOE678 | Aphididae | Aphidinae | *Aphis* | *newtoni* | Theobald, 1927 | Coeur d'Acier | 1999 |
| ACOE679 | Aphididae | Aphidinae | *Brachycaudus* | *schwartzi* | (Börner, 1931) | Coeur d'Acier | 1999 |
| ACOE682 | Aphididae | Aphidinae | *Brachycaudus* | *cardui* | (Linnaeus, 1860) | Coeur d'Acier | 1999 |
| ACOE683 | Aphididae | Aphidinae | *Aphis* | *verbasci* | Schrank, 1801 | Coeur d'Acier | 1999 |
| ACOE684 | Aphididae | Aphidinae | *Aphis* | *fabae* | Scopoli, 1763 | Coeur d'Acier | 1999 |
| ACOE685 | Aphididae | Aphidinae | *Aphis* | *fabae* | Scopoli, 1763 | Coeur d'Acier | 1999 |
| ACOE686 | Aphididae | Aphidinae | *Aphis* | *pseudocomosa* | Stroyan, 1972 | Coeur d'Acier | 1999 |
| ACOE687 | Aphididae | Aphidinae | *Uroleucon* | *achilleae* | (Koch, 1855) | Coeur d'Acier | 1999 |
| ACOE688 | Aphididae | Aphidinae | *Toxopterina* | *vandergooti* | (Börner, 1939) | Coeur d'Acier | 1999 |
| ACOE691 | Aphididae | Aphidinae | *Aphis* | *sp.frangulae-like* |  | Coeur d'Acier | 1999 |
| ACOE692 | Aphididae | Aphidinae | *Macrosiphoniella* | *artemisiae* | (Boyer de Fonscolombe, 1841) | Coeur d'Acier | 2007 |
| ACOE693 | Aphididae | Chaitophorinae | *Periphyllus* | *testudinaceus* | (Fernie, 1852) | Coeur d'Acier | 1999 |
| ACOE694 | Aphididae | Aphidinae | *Aphis* | *cacaliasteris* | Hille Ris Lambers, 1947 | Coeur d'Acier | 1999 |
| ACOE695 | Aphididae | Aphidinae | *Macrosiphum* | *rosae* | (Linnaeus, 1758) | Coeur d'Acier | 1999 |
| ACOE696 | Aphididae | Aphidinae | *Aphis* | *grossulariae* | Kaltenbach, 1843 | Coeur d'Acier | 1999 |
| ACOE697 | Aphididae | Aphidinae | *Aphis* | *fabae* | Scopoli, 1763 | Coeur d'Acier | 1999 |
| ACOE700 | Aphididae | Aphidinae | *Aphis* | *confusa* | Walker, 1849 | Coeur d'Acier | 1999 |
| ACOE701 | Aphididae | Aphidinae | *Toxopterina* | *vandergooti* | (Börner, 1939) | Coeur d'Acier | 1999 |
| ACOE702 | Aphididae | Aphidinae | *Aphis* | *fabae* | Scopoli, 1763 | Coeur d'Acier | 1999 |
| ACOE704 | Aphididae | Aphidinae | *Aphis* | *fabae* | Scopoli, 1763 | Coeur d'Acier | 1999 |
| ACOE705 | Aphididae | Aphidinae | *Brachycaudus* | *helichrysi* | (Kaltenbach, 1843) | Coeur d'Acier | 1999 |
| ACOE706 | Aphididae | Aphidinae | *Aphis* | *fabae* | Scopoli, 1763 | Coeur d'Acier | 1999 |
| ACOE707 | Aphididae | Aphidinae | *Brachycaudus* | *helichrysi* | (Kaltenbach, 1843) | Coeur d'Acier | 1999 |
| ACOE708 | Aphididae | Aphidinae | *Aphis* | *fabae* | Scopoli, 1763 | Coeur d'Acier | 1999 |
| ACOE711 | Aphididae | Aphidinae | *Aphis* | *leontodontis* | (Börner, 1950) | Coeur d'Acier | 1999 |
| ACOE713 | Aphididae | Aphidinae | *Aphis* | *sambuci* | Linnaeus, 1758 | Coeur d'Acier | 1999 |
| ACOE718 | Aphididae | Aphidinae | *Aphis* | *pomi* | de Geer, 1773 | Coeur d'Acier | 1999 |
| ACOE720 | Aphididae | Aphidinae | *Macrosiphum* | *rosae* | (Linnaeus, 1758) | Coeur d'Acier | 1999 |
| ACOE721 | Aphididae | Aphidinae | *Aphis* | *pomi* | de Geer, 1773 | Coeur d'Acier | 1999 |
| ACOE722 | Aphididae | Aphidinae | *Uroleucon* | *sonchi* | (Linnaeus, 1767) | Coeur d'Acier | 1999 |
| ACOE724 | Aphididae | Aphidinae | *Uroleucon* | *hypochoeridis* | (Fabricius, 1779) | Coeur d'Acier | 1999 |
| ACOE726 | Aphididae | Aphidinae | *Aphis* | *grossulariae* | Kaltenbach, 1843 | Coeur d'Acier | 1999 |
| ACOE729 | Aphididae | Aphidinae | *Aphis* | *ruborum* | (Börner, 1932) | Coeur d'Acier | 1999 |
| ACOE730 | Aphididae | Aphidinae | *Aphis* | *coronillae* | Ferrari, 1872 | Coeur d'Acier | 1999 |
| ACOE731 | Aphididae | Aphidinae | *Aphis* | *fabae* | Scopoli, 1763 | Coeur d'Acier | 1999 |
| ACOE732 | Aphididae | Aphidinae | *Brachycaudus* | *lateralis* | (Walker, 1848) | Coeur d'Acier | 1999 |
| ACOE733 | Aphididae | Aphidinae | *Hyperomyzus* | *lactucae* | (Linnaeus, 1758) | Coeur d'Acier | 1999 |
| ACOE734 | Aphididae | Aphidinae | *Semiaphis* | *dauci* | (Fabricius, 1775) | Coeur d'Acier | 2007 |
| ACOE735 | Aphididae | Aphidinae | *Dysaphis* | *crataegi* | (Kaltenbach, 1843) | Coeur d'Acier | 1999 |
| ACOE736 | Aphididae | Aphidinae | *Aphis* | *coronillae* | Ferrari, 1872 | Coeur d'Acier | 1999 |
| ACOE739 | Aphididae | Aphidinae | *Uroleucon* | *hypochoeridis* | (Fabricius, 1779) | Coeur d'Acier | 1999 |
| ACOE741 | Aphididae | Aphidinae | *Dysaphis* | *radicola* | (Mordvilko, 1897) | Coeur d'Acier | 1999 |
| ACOE742 | Aphididae | Calaphidinae | *Myzocallis* | *coryli* | (Schrank, 1801) | Coeur d'Acier | 1999 |
| ACOE744 | Aphididae | Aphidinae | *Aphis* | *fabae* | Scopoli, 1763 | Coeur d'Acier | 1999 |
| ACOE747 | Aphididae | Aphidinae | *Brachycaudus* | *lateralis* | (Walker, 1848) | Coeur d'Acier | 1999 |
| ACOE748 | Aphididae | Aphidinae | *Hyalopterus* | *pruni* | (Geoffroy, 1762) | Coeur d'Acier | 1999 |
| ACOE749 | Aphididae | Aphidinae | *Aphis* | *fabae* | Scopoli, 1763 | Coeur d'Acier | 1999 |
| ACOE750 | Aphididae | Aphidinae | *Aphis* | *newtoni* | Theobald, 1927 | Coeur d'Acier | 1999 |
| ACOE752 | Aphididae | Aphidinae | *Macrosiphum* | *rosae* | (Linnaeus, 1758) | Coeur d'Acier | 1999 |
| ACOE755 | Aphididae | Thelaxinae | *Thelaxes* | *dryophila* | (Schrank, 1801) | Coeur d'Acier | 1999 |
| ACOE756 | Aphididae | Calaphidinae | *Pterocallis* | *alni* | (De Geer, 1773) | Coeur d'Acier | 1999 |
| ACOE758 | Aphididae | Lachninae | *Cinara* | *pilicornis* | (Hartig, 1841) | Coeur d'Acier | 1999 |
| ACOE759 | Aphididae | Aphidinae | *Aphis* | *fabae* | Scopoli, 1763 | Coeur d'Acier | 1999 |
| ACOE761 | Aphididae | Aphidinae | *Brachycaudus* | *helichrysi* | (Kaltenbach, 1843) | Coeur d'Acier | 1999 |
| ACOE762 | Aphididae | Aphidinae | *Corylobium* | *avellanae* | (Schrank, 1801) | Coeur d'Acier | 1999 |
| ACOE765 | Aphididae | Lachninae | *Stomaphis* | *longirostris* | (Fabricius, 1787) | Coeur d'Acier | 2007 |
| ACOE819 | Aphididae | Lachninae | *Cinara* | *maghrebica* | Mimeur, 1934 | Coeur d'Acier | 2000 |
| ACOE824 | Aphididae | Aphidinae | *Nearctaphis* | *bakeri* | (Cowen, 1895) | Coeur d'Acier | 2000 |
| ACOE827 | Aphididae | Aphidinae | *Aphis* | *serpylli* | Koch, 1854 | Coeur d'Acier | 2000 |
| ACOE828 | Aphididae | Aphidinae | *Macchiatiella* | *rhamni* | (Boyer de Fonscolombe, 1841) | Coeur d'Acier | 2000 |
| ACOE829 | Aphididae | Aphidinae | *Aphis* | *cytisorum* | Hartig, 1841 | Coeur d'Acier | 2000 |
| ACOE830 | Aphididae | Aphidinae | *Hyperomyzus* | *picridis* | (Börner & Blunck, 1916) | Coeur d'Acier | 2000 |
| ACOE831 | Aphididae | Aphidinae | *Sitobion* | *fragariae* | (Walker, 1848) | Coeur d'Acier | 2000 |
| ACOE832 | Aphididae | Aphidinae | *Uroleucon* | *sp.* | Mordvilko, 1914 | Coeur d'Acier | 2000 |
| ACOE834 | Aphididae | Aphidinae | *Dysaphis* | *crataegi* | (Kaltenbach, 1843) | Coeur d'Acier | 2007 |
| ACOE838 | Aphididae | Aphidinae | *Macrosiphum* | *euphorbiae* | (Thomas, 1878) | Coeur d'Acier | 2000 |
| ACOE855 | Aphididae | Aphidinae | *Aphis* | *oenotherae* | Oestlund, 1887 | Coeur d'Acier | 2000 |
| ACOE875 | Aphididae | Chaitophorinae | *Chaitophorus* | *truncatus* | (Hausmann, 1802) | Coeur d'Acier | 2000 |
| ACOE899 | Aphididae | Aphidinae | *Aphis* | *fabae* | Linnaeus, 1758 | Coeur d'Acier | 2000 |
| ACOE902 | Aphididae | Aphidinae | *Aphis* | *cytisorum* | Hartig, 1841 | Coeur d'Acier | 2000 |
| ACOE903 | Aphididae | Chaitophorinae | *Chaitophorus* | *leucomelas* | Koch, 1854 | Coeur d'Acier | 2000 |
| ACOE904 | Aphididae | Aphidinae | *Aphis* | *lambersi* | (Börner, 1940) | Coeur d'Acier | 2000 |
| ACOE905 | Aphididae | Aphidinae | *Uroleucon* | *hypochoeridis* | (Fabricius, 1779) | Coeur d'Acier | 2000 |
| ACOE906 | Aphididae | Aphidinae | *Aphis* | *craccivora* | Koch, 1854 | Coeur d'Acier | 2000 |
| ACOE907 | Aphididae | Aphidinae | *Aphis* | *fabae* | Scopoli, 1763 | Coeur d'Acier | 2000 |
| ACOE908 | Aphididae | Chaitophorinae | *Chaitophorus* | *populeti* | Mimeur, 1934 | Coeur d'Acier | 2000 |
| ACOE910 | Aphididae | Aphidinae | *Hyperomyzus* | *lactucae* | (Linnaeus, 1758) | Coeur d'Acier | 2000 |
| ACOE911 | Aphididae | Aphidinae | *Aphis* | *spiraecola* | Patch, 1914 | Coeur d'Acier | 2000 |
| ACOE912 | Aphididae | Aphidinae | *Aphis* | *fabae* | Scopoli, 1763 | Coeur d'Acier | 2000 |
| ACOE913 | Aphididae | Aphidinae | *Macrosiphum* | *euphorbiae* | (Thomas, 1878) | Coeur d'Acier | 2000 |
| ACOE917 | Aphididae | Aphidinae | *Melanaphis* | *donacis* | (Passerini, 1862) | Coeur d'Acier | 2000 |
| ACOE918 | Aphididae | Aphidinae | *Brachycaudus* | *lychnidis* | (Linnaeus, 1758) | Coeur d'Acier | 2000 |
| ACOE922 | Aphididae | Aphidinae | *Uroleucon* | *jaceae* | (Linnaeus, 1758) | Coeur d'Acier | 2000 |
| ACOE923 | Aphididae | Chaitophorinae | *Chaitophorus* | *leucomelas* | Koch, 1854 | Coeur d'Acier | 2000 |
| ACOE924 | Aphididae | Aphidinae | *Myzus* | *cerasi* | (Fabricius, 1775) | Coeur d'Acier | 2000 |
| ACOE926 | Aphididae | Aphidinae | *Hyperomyzus* | *lactucae* | (Linnaeus, 1758) | Coeur d'Acier | 2000 |
| ACOE929 | Aphididae | Aphidinae | *Aphis* | *lichtensteini* | Leclant & Remaudière, 1972 | Coeur d'Acier | 2000 |
| ACOE932 | Aphididae | Aphidinae | *Dysaphis* | *apiifolia* | (Theobald, 1923) | Coeur d'Acier | 2000 |
| ACOE935 | Aphididae | Aphidinae | *Uroleucon* | *hypochoeridis* | (Fabricius, 1779) | Coeur d'Acier | 2000 |
| ACOE936 | Aphididae | Aphidinae | *Aphis* | *oenotherae* | Oestlund, 1887 | Coeur d'Acier | 2000 |
| ACOE939 | Aphididae | Aphidinae | *Aphis* | *sedi* | Kaltenbach, 1843 | Coeur d'Acier | 2000 |
| ACOE941 | Aphididae | Aphidinae | *Macrosiphum* | *euphorbiae* | (Thomas, 1878) | Coeur d'Acier | 2000 |
| ACOE943 | Aphididae | Aphidinae | *Hyalopterus* | *pruni* | (Geoffroy, 1762) | Coeur d'Acier | 2000 |
| ACOE944 | Aphididae | Calaphidinae | *Therioaphis* | *riehmi* | (Börner, 1949) | Coeur d'Acier | 2000 |
| ACOE945 | Aphididae | Aphidinae | *Brachycaudus* | *lateralis* | (Walker, 1848) | Coeur d'Acier | 2000 |
| ACOE946 | Aphididae | Aphidinae | *Uroleucon* | *sonchi* | (Linnaeus, 1767) | Coeur d'Acier | 2000 |
| ACOE947 | Aphididae | Aphidinae | *Dysaphis* | *apiifolia* | (Theobald, 1923) | Coeur d'Acier | 2000 |
| ACOE948 | Aphididae | Chaitophorinae | *Chaitophorus* | *populeti* | (Panzer, 1801) | Coeur d'Acier | 2000 |
| ACOE952 | Aphididae | Aphidinae | *Aphis* | *fabae* | Scopoli, 1763 | Coeur d'Acier | 2000 |
| ACOE953 | Aphididae | Aphidinae | *Aphis* | *fabae* | Scopoli, 1763 | Coeur d'Acier | 2000 |
| ACOE955 | Aphididae | Chaitophorinae | *Chaitophorus* | *populeti* | (Panzer, 1801) | Coeur d'Acier | 2000 |
| ACOE956 | Aphididae | Chaitophorinae | *Chaitophorus* | *populialbae* | (Boyer de Fonscolombe, 1841) | Coeur d'Acier | 2000 |
| ACOE957 | Aphididae | Aphidinae | *Aphis* | *oenotherae* | Oestlund, 1887 | Coeur d'Acier | 2000 |
| ACOE959 | Aphididae | Aphidinae | *Aphis* | *ulicis* | Walker, 1870 | Coeur d'Acier | 2000 |
| ACOE960 | Aphididae | Aphidinae | *Aphis* | *epilobii* | Kaltenbach, 1843 | Coeur d'Acier | 2000 |
| ACOE962 | Aphididae | Aphidinae | *Amphorophora* | *rubi* | (Kaltenbach, 1843) | Coeur d'Acier | 2000 |
| ACOE963 | Aphididae | Aphidinae | *Brachycaudus* | *bicolor* | (Nevsky, 1929) | Coeur d'Acier | 2001 |
| ACOE966 | Aphididae | Aphidinae | *Aphis* | *longirostris* | (Börner, 1950) | Coeur d'Acier | 2000 |
| ACOE967 | Aphididae | Aphidinae | *Hyperomyzus* | *lactucae* | (Linnaeus, 1758) | Coeur d'Acier | 2000 |
| ACOE969 | Aphididae | Aphidinae | *Uroleucon* | *hypochoeridis* | (Fabricius, 1779) | Coeur d'Acier | 2000 |
| ACOE970 | Aphididae | Aphidinae | *Hayhurstia* | *atriplicis* | (Linnaeus, 1761) | Coeur d'Acier | 2000 |
| ACOE971 | Aphididae | Aphidinae | *Brachycaudus* | *lateralis* | (Walker, 1848) | Coeur d'Acier | 2000 |
| ACOE973 | Aphididae | Aphidinae | *Dysaphis* | *crithmi* | (Buckton, 1886) | Coeur d'Acier | 2000 |
| ACOE974 | Aphididae | Aphidinae | *Cavariella* | *theobaldi* | (Gillette & Bragg, 1918) | Coeur d'Acier | 2000 |
| ACOE975 | Aphididae | Aphidinae | *Hyperomyzus* | *lactucae* | (Linnaeus, 1758) | Coeur d'Acier | 2000 |
| ACOE977 | Aphididae | Aphidinae | *Hayhurstia* | *atriplicis* | (Linnaeus, 1761) | Coeur d'Acier | 2000 |
| ACOE979 | Aphididae | Aphidinae | *Aphis* | *fabae* | Scopoli, 1763 | Coeur d'Acier | 2000 |
| ACOE980 | Aphididae | Chaitophorinae | *Chaitophorus* | *leucomelas* | Koch, 1854 | Coeur d'Acier | 2000 |
| ACOE984 | Aphididae | Aphidinae | *Dysaphis* | *plantaginea* | (Passerini, 1860) | Coeur d'Acier | 2000 |
| ACOE987 | Aphididae | Aphidinae | *Aphis* | *ruborum* | (Börner, 1932) | Coeur d'Acier | 2000 |
| ACOE988 | Aphididae | Aphidinae | *Aphis* | *sanguisorbae* | Schrank, 1801 | Coeur d'Acier | 2000 |
| ACOE991 | Aphididae | Aphidinae | *Aphis* | *fabae* | Scopoli, 1763 | Coeur d'Acier | 2000 |
| ACOE992 | Aphididae | Aphidinae | *Brachycaudus* | *prunicola* | (Kaltenbach, 1843) | Coeur d'Acier | 2000 |
| ACOE993 | Aphididae | Aphidinae | *Aphis* | *ruborum* | (Börner, 1932) | Coeur d'Acier | 2000 |
| ACOE994 | Aphididae | Calaphidinae | *Tuberculatus* | *annulatus* | (Hartig, 1841) | Coeur d'Acier | 2000 |
| ACOE995 | Aphididae | Aphidinae | *Aphis* | *newtoni* | Theobald, 1927 | Coeur d'Acier | 2000 |
| ACOE996 | Aphididae | Aphidinae | *Aphis* | *fabae* | Theobald, 1914 | Coeur d'Acier | 2000 |
| ACOE997 | Aphididae | Aphidinae | *Aphis* | *rumicis* | Linnaeus, 1758 | Coeur d'Acier | 2000 |
| ACOE998 | Aphididae | Aphidinae | *Uroleucon* | *hypochoeridis* | (Fabricius, 1779) | Coeur d'Acier | 2000 |
| ACOE999 | Aphididae | Aphidinae | *Aphis* | *grossulariae* | Kaltenbach, 1843 | Coeur d'Acier | 2000 |
| ACOE1001 | Aphididae | Thelaxinae | *Thelaxes* | *dryophila* | (Schrank, 1801) | Coeur d'Acier | 2000 |
| ACOE1002 | Aphididae | Aphidinae | *Aphis* | *acetosae* | Heie, 1986 | Coeur d'Acier | 2000 |
| ACOE1003 | Aphididae | Aphidinae | *Hyalopterus* | *pruni* | (Geoffroy, 1762) | Coeur d'Acier | 2000 |
| ACOE1004 | Aphididae | Aphidinae | *Aphis* | *craccivora* | Koch, 1854 | Coeur d'Acier | 2000 |
| ACOE1005 | Aphididae | Chaitophorinae | *Chaitophorus* | *salicti* | (Schrank, 1801) | Coeur d'Acier | 2000 |
| ACOE1006 | Aphididae | Aphidinae | *Aphis* | *serpylli* | Koch, 1854 | Coeur d'Acier | 2000 |
| ACOE1007 | Aphididae | Aphidinae | *Hydaphias* | *mosana* | Hille Ris Lambers, 1956 | Coeur d'Acier | 2000 |
| ACOE1009 | Aphididae | Aphidinae | *Cavariella* | *theobaldi* | (Gillette & Bragg, 1918) | Coeur d'Acier | 2000 |
| ACOE1010 | Aphididae | Aphidinae | *Aphis* | *sp.rostellum.like* |  | Coeur d'Acier | 2000 |
| ACOE1011 | Aphididae | Aphidinae | *Dysaphis* | *lauberti* | (Börner, 1940) | Coeur d'Acier | 2000 |
| ACOE1012 | Aphididae | Aphidinae | *Hyalopterus* | *pruni* | (Geoffroy, 1762) | Coeur d'Acier | 2000 |
| ACOE1014 | Aphididae | Aphidinae | *Uroleucon* | *picridis* | (Fabricius, 1775) | Coeur d'Acier | 2000 |
| ACOE1015 | Aphididae | Aphidinae | *Dysaphis* | *crithmi* | (Buckton, 1886) | Coeur d'Acier | 2000 |
| ACOE1016 | Aphididae | Aphidinae | *Aphis* | *lambersi* | (Börner, 1940) | Coeur d'Acier | 2000 |
| ACOE1017 | Aphididae | Aphidinae | *Dysaphis* | *crataegi* | (Kaltenbach, 1843) | Coeur d'Acier | 2000 |
| ACOE1018 | Aphididae | Aphidinae | *Uroleucon* | *jaceae* | (Linnaeus, 1758) | Coeur d'Acier | 2000 |
| ACOE1020 | Aphididae | Aphidinae | *Nearctaphis* | *bakeri* | (Cowen, 1895) | Coeur d'Acier | 2000 |
| ACOE1022 | Aphididae | Aphidinae | *Aphis* | *ulicis* | Walker, 1870 | Coeur d'Acier | 2000 |
| ACOE1028 | Aphididae | Aphidinae | *Aphis* | *fabae* | Scopoli, 1763 | Coeur d'Acier | 2000 |
| ACOE1029 | Aphididae | Aphidinae | *Macrosiphum* | *euphorbiae* | (Thomas, 1878) | Coeur d'Acier | 2000 |
| ACOE1031 | Aphididae | Aphidinae | *Brachycaudus* | *klugkisti* | (Börner, 1942) | Coeur d'Acier | 2000 |
| ACOE1033 | Aphididae | Aphidinae | *Uroleucon* | *hypochoeridis* | (Fabricius, 1779) | Coeur d'Acier | 2007 |
| ACOE1036 | Aphididae | Aphidinae | *Amphorophora* | *rubi* | (Kaltenbach, 1843) | Coeur d'Acier | 2000 |
| ACOE1037 | Aphididae | Aphidinae | *Aphis* | *ilicis* | Kaltenbach, 1843 | Coeur d'Acier | 2000 |
| ACOE1038 | Aphididae | Aphidinae | *Aphis* | *fabae* | Scopoli, 1763 | Coeur d'Acier | 2000 |
| ACOE1041 | Aphididae | Aphidinae | *Aphis* | *sedi* | Kaltenbach, 1843 | Coeur d'Acier | 2000 |
| ACOE1044 | Aphididae | Aphidinae | *Brevicoryne* | *brassicae* | (Linnaeus, 1758) | Coeur d'Acier | 2002 |
| ACOE1045 | Aphididae | Aphidinae | *Aphis* | *nerii* | Boyer de Fonscolombe, 1841 | Coeur d'Acier | 2002 |
| ACOE1046 | Aphididae | Lachninae | *Cinara* | *palaestinensis* | Hille Ris Lambers, 1938 | Coeur d'Acier | 2002 |
| ACOE1047 | Aphididae | Aphidinae | *Protaphis* | *terricola* | (Rondani, 1848) | Coeur d'Acier | 2007 |
| ACOE1048 | Aphididae | Aphidinae | *Aphis* | *craccivora* | Koch, 1854 | Coeur d'Acier | 2007 |
| ACOE1049 | Aphididae | Aphidinae | *Aphis* | *gossypii* | Glover, 1877 | Coeur d'Acier | 2007 |
| ACOE1051 | Aphididae | Aphidinae | *Aphis* | *craccivora* | Koch, 1854 | Coeur d'Acier | 2007 |
| ACOE1052 | Aphididae | Aphidinae | *Aphis* | *craccivora* | Koch, 1854 | Coeur d'Acier | 2007 |
| ACOE1053 | Aphididae | Aphidinae | *Uroleucon* | *inulae* | (Ferrari, 1872) | Coeur d'Acier | 2002 |
| ACOE1054 | Aphididae | Aphidinae | *Aphis* | *sp.frangulae-like* |  | Coeur d'Acier | 2002 |
| ACOE1055 | Aphididae | Aphidinae | *Aphis* | *craccivora* | Koch, 1854 | Coeur d'Acier | 2007 |
| ACOE1056 | Aphididae | Aphidinae | *Acyrthosiphon* | *lambersi* | Leclant & Remaudière, 1974 | Coeur d'Acier | 2007 |
| ACOE1057 | Aphididae | Aphidinae | *Anuraphis* | *cachryos* | Barbagallo & Stroyan, 1980 | Coeur d'Acier | 2007 |
| ACOE1058 | Aphididae | Chaitophorinae | *Chaitophorus* | *populeti* | (Panzer, 1801) | Coeur d'Acier | 2007 |
| ACOE1059 | Aphididae | Aphidinae | *Protaphis* | *terricola* | (Rondani, 1848) | Coeur d'Acier | 2007 |
| ACOE1060 | Aphididae | Chaitophorinae | *Chaitophorus* | *populeti* | (Panzer, 1801) | Coeur d'Acier | 2001 |
| ACOE1062 | Aphididae | Chaitophorinae | *Chaitophorus* | *leucomelas* | Koch, 1854 | Coeur d'Acier | 2001 |
| ACOE1063 | Aphididae | Pterocommatinae | *Pterocomma* | *populeum* | (Kaltenbach, 1843) | Coeur d'Acier | 2001 |
| ACOE1064 | Aphididae | Aphidinae | *Aphis* | *craccivora* | Koch, 1854 | Coeur d'Acier | 2007 |
| ACOE1066 | Aphididae | Chaitophorinae | *Chaitophorus* | *populeti* | (Panzer, 1801) | Coeur d'Acier | 2001 |
| ACOE1067 | Aphididae | Aphidinae | *Aphis* | *spiraecola* | Patch, 1914 | Coeur d'Acier | 2001 |
| ACOE1069 | Aphididae | Aphidinae | *Brevicoryne* | *brassicae* | (Linnaeus, 1758) | Coeur d'Acier | 2001 |
| ACOE1071 | Aphididae | Chaitophorinae | *Periphyllus* | *acericola* | (Walker, 1848) | Coeur d'Acier | 2001 |
| ACOE1073 | Aphididae | Aphidinae | *Hyperomyzus* | *lactucae* | (Linnaeus, 1758) | Coeur d'Acier | 2001 |
| ACOE1074 | Aphididae | Aphidinae | *Aphis* | *hederae* | Kaltenbach, 1843 | Coeur d'Acier | 2001 |
| ACOE1077 | Aphididae | Aphidinae | *Brachycaudus* | *persicae* | (Passerini, 1860) | Coeur d'Acier | 2001 |
| ACOE1078 | Aphididae | Aphidinae | *Anuraphis* | *subterranea* | (Walker, 1852) | Coeur d'Acier | 2001 |
| ACOE1080 | Aphididae | Aphidinae | *Macrosiphum* | *euphorbiae* | (Thomas, 1878) | Coeur d'Acier | 2003 |
| ACOE1083 | Aphididae | Aphidinae | *Aphis* | *urticata* | Gmelin, 1790 | Coeur d'Acier | 2001 |
| ACOE1085 | Aphididae | Aphidinae | *Hyperomyzus* | *lactucae* | (Linnaeus, 1758) | Coeur d'Acier | 2001 |
| ACOE1086 | Aphididae | Aphidinae | *Aphis* | *fabae* | Scopoli, 1763 | Coeur d'Acier | 2001 |
| ACOE1087 | Aphididae | Aphidinae | *Aphis* | *lugentis* | Williams, 1911 | Coeur d'Acier | 2001 |
| ACOE1088 | Aphididae | Aphidinae | *Aphis* | *fabae* | Scopoli, 1763 | Coeur d'Acier | 2001 |
| ACOE1091 | Aphididae | Aphidinae | *Megoura* | *viciae* | Buckton, 1876 | Coeur d'Acier | 2001 |
| ACOE1092 | Aphididae | Chaitophorinae | *Sipha* | *elegans* | del Guercio, 1905 | Coeur d'Acier | 2001 |
| ACOE1093 | Aphididae | Aphidinae | *Macrosiphum* | *rosae* | (Linnaeus, 1758) | Coeur d'Acier | 2001 |
| ACOE1094 | Aphididae | Aphidinae | *Brachycaudus* | *lychnidis* | (Linnaeus, 1758) | Coeur d'Acier | 2001 |
| ACOE1099 | Aphididae | Aphidinae | *Aphis* | *umbrella* | (Börner, 1950) | Coeur d'Acier | 2001 |
| ACOE1100 | Aphididae | Aphidinae | *Semiaphis* | *dauci* | (Fabricius, 1775) | Coeur d'Acier | 2001 |
| ACOE1101 | Aphididae | Lachninae | *Lachnus* | *roboris* | (Linnaeus, 1758) | Coeur d'Acier | 2001 |
| ACOE1109 | Aphididae | Aphidinae | *Aphis* | *craccivora* | Koch, 1854 | Coeur d'Acier | 2001 |
| ACOE1112 | Aphididae | Aphidinae | *Aphis* | *fabae* | Scopoli, 1763 | Coeur d'Acier | 2001 |
| ACOE1114 | Aphididae | Lachninae | *Cinara* | *pinea* | (Mordvilko, 1895) | Coeur d'Acier | 2001 |
| ACOE1115 | Aphididae | Aphidinae | *Nearctaphis* | *bakeri* | (Cowen, 1895) | Coeur d'Acier | 2001 |
| ACOE1116 | Aphididae | Aphidinae | *Macrosiphum* | *rosae* | (Linnaeus, 1758) | Coeur d'Acier | 2001 |
| ACOE1121 | Aphididae | Chaitophorinae | *Periphyllus* | *testudinaceus* | (Fernie, 1852) | Coeur d'Acier | 2001 |
| ACOE1123 | Aphididae | Aphidinae | *Hyperomyzus* | *picridis* | (Börner & Blunck, 1916) | Coeur d'Acier | 2002 |
| ACOE1124 | Aphididae | Aphidinae | *Myzus* | *cerasi* | (Fabricius, 1775) | Coeur d'Acier | 2001 |
| ACOE1125 | Aphididae | Eriosomatinae | *Prociphilus* | *bumeliae* | (Schrank, 1801) | Coeur d'Acier | 2007 |
| ACOE1126 | Aphididae | Aphidinae | *Brachycaudus* | *klugkisti* | (Börner, 1942) | Coeur d'Acier | 2007 |
| ACOE1127 | Aphididae | Aphidinae | *Aphis* | *lugentis* | Williams, 1911 | Coeur d'Acier | 2002 |
| ACOE1128 | Aphididae | Aphidinae | *Aphis* | *fabae* | Scopoli, 1763 | Coeur d'Acier | 2001 |
| ACOE1129 | Aphididae | Aphidinae | *Macrosiphum* | *euphorbiae* | (Thomas, 1878) | Coeur d'Acier | 2001 |
| ACOE1131 | Aphididae | Eriosomatinae | *Prociphilus* | *bumeliae* | (Schrank, 1801) | Coeur d'Acier | 2001 |
| ACOE1132 | Aphididae | Aphidinae | *Aphis* | *fabae* | Scopoli, 1763 | Coeur d'Acier | 2002 |
| ACOE1136 | Aphididae | Aphidinae | *Amphorophora* | *rubi* | (Kaltenbach, 1843) | Coeur d'Acier | 2001 |
| ACOE1137 | Aphididae | Aphidinae | *Macrosiphum* | *euphorbiae* | (Thomas, 1878) | Coeur d'Acier | 2002 |
| ACOE1139 | Aphididae | Aphidinae | *Aphis* | *urticata* | Gmelin, 1790 | Coeur d'Acier | 2002 |
| ACOE1140 | Aphididae | Chaitophorinae | *Chaitophorus* | *populeti* | (Panzer, 1801) | Coeur d'Acier | 2002 |
| ACOE1141 | Aphididae | Aphidinae | *Aphis* | *confusa* | Walker, 1849 | Coeur d'Acier | 2007 |
| ACOE1142 | Aphididae | Eriosomatinae | *Forda* | *marginata* | Koch, 1857 | Coeur d'Acier | 2002 |
| ACOE1144 | Aphididae | Aphidinae | *Hyadaphis* | *foeniculi* | (Passerini, 1860) | Coeur d'Acier | 2002 |
| ACOE1145 | Aphididae | Pterocommatinae | *Pterocomma* | *pilosum* | Buckton, 1879 | Coeur d'Acier | 2001 |
| ACOE1148 | Aphididae | Aphidinae | *Macrosiphoniella* | *millefolii* | (de Geer, 1773) | Coeur d'Acier | 2001 |
| ACOE1149 | Aphididae | Aphidinae | *Brachycaudus* | *helichrysi* | (Kaltenbach, 1843) | Coeur d'Acier | 2001 |
| ACOE1150 | Aphididae | Aphidinae | *Aphis* | *sambuci* | Linnaeus, 1758 | Coeur d'Acier | 2001 |
| ACOE1152 | Aphididae | Aphidinae | *Aphis* | *viburni* | Scopoli, 1763 | Coeur d'Acier | 2001 |
| ACOE1153 | Aphididae | Aphidinae | *Aphis* | *farinosa* | Gmelin, 1790 | Coeur d'Acier | 2001 |
| ACOE1154 | Aphididae | Calaphidinae | *Myzocallis* | *coryli* | (Goeze, 1778) | Coeur d'Acier | 2007 |
| ACOE1155 | Aphididae | Chaitophorinae | *Chaitophorus* | *populeti* | (Panzer, 1801) | Coeur d'Acier | 2001 |
| ACOE1157 | Aphididae | Chaitophorinae | *Chaitophorus* | *salicti* | (Schrank, 1801) | Coeur d'Acier | 2001 |
| ACOE1158 | Aphididae | Chaitophorinae | *Periphyllus* | *testudinaceus* | (Fernie, 1852) | Coeur d'Acier | 2001 |
| ACOE1159 | Aphididae | Aphidinae | *Aphis* | *grossulariae* | Kaltenbach, 1843 | Coeur d'Acier | 2001 |
| ACOE1160 | Aphididae | Chaitophorinae | *Chaitophorus* | *capreae* | (Mosley, 1841) | Coeur d'Acier | 2001 |
| ACOE1161 | Aphididae | Calaphidinae | *Myzocallis* | *carpini* | (Koch, 1855) | Coeur d'Acier | 2001 |
| ACOE1164 | Aphididae | Aphidinae | *Aphis* | *ilicis* | Kaltenbach, 1843 | Coeur d'Acier | 2001 |
| ACOE1165 | Aphididae | Aphidinae | *Macrosiphum* | *rosae* | (Linnaeus, 1758) | Coeur d'Acier | 2001 |
| ACOE1166 | Aphididae | Aphidinae | *Myzus* | *cerasi* | (Fabricius, 1775) | Coeur d'Acier | 2001 |
| ACOE1169 | Aphididae | Aphidinae | *Macrosiphoniella* | *tanacetaria* | (Kaltenbach, 1843) | Coeur d'Acier | 2001 |
| ACOE1171 | Aphididae | Aphidinae | *Cavariella* | *theobaldi* | (Gillette & Bragg, 1918) | Coeur d'Acier | 2001 |
| ACOE1172 | Aphididae | Aphidinae | *Macrosiphum* | *rosae* | (Linnaeus, 1758) | Coeur d'Acier | 2001 |
| ACOE1173 | Aphididae | Aphidinae | *Aphis* | *fabae* | Scopoli, 1763 | Coeur d'Acier | 2001 |
| ACOE1175 | Aphididae | Aphidinae | *Macrosiphum* | *rosae* | (Linnaeus, 1758) | Coeur d'Acier | 2001 |
| ACOE1176 | Aphididae | Aphidinae | *Aphis* | *sp.frangulae-like* |  | Coeur d'Acier | 2002 |
| ACOE1178 | Aphididae | Aphidinae | *Brachycaudus* | *tragopogonis* | (Kaltenbach, 1843) | Coeur d'Acier | 2001 |
| ACOE1179 | Aphididae | Aphidinae | *Brachycaudus* | *cardui* | (Linnaeus, 1860) | Coeur d'Acier | 2002 |
| ACOE1180 | Aphididae | Aphidinae | *Phorodon* | *humuli* | (Schrank, 1801) | Coeur d'Acier | 2001 |
| ACOE1185 | Aphididae | Aphidinae | *Aphis* | *oenotherae* | Oestlund, 1887 | Coeur d'Acier | 2001 |
| ACOE1186 | Aphididae | Aphidinae | *Brachycaudus* | *cardui* | (Linnaeus, 1860) | Coeur d'Acier | 2001 |
| ACOE1188 | Aphididae | Aphidinae | *Brachycaudus* | *lychnidis* | (Linnaeus, 1758) | Coeur d'Acier | 2001 |
| ACOE1190 | Aphididae | Aphidinae | *Aphis* | *urticata* | Gmelin, 1790 | Coeur d'Acier | 2001 |
| ACOE1191 | Aphididae | Aphidinae | *Dysaphis* | *lauberti* | (Börner, 1940) | Coeur d'Acier | 2001 |
| ACOE1192 | Aphididae | Aphidinae | *Aphis* | *ruborum* | (Börner, 1932) | Coeur d'Acier | 2001 |
| ACOE1193 | Aphididae | Aphidinae | *Amphorophora* | *rubi* | (Kaltenbach, 1843) | Coeur d'Acier | 2001 |
| ACOE1194 | Aphididae | Aphidinae | *Dysaphis* | *crithmi* | (Buckton, 1886) | Coeur d'Acier | 2001 |
| ACOE1195 | Aphididae | Aphidinae | *Macrosiphum* | *euphorbiae* | (Thomas, 1878) | Coeur d'Acier | 2002 |
| ACOE1196 | Aphididae | Calaphidinae | *Tuberculatus* | *annulatus* | (Hartig, 1841) | Coeur d'Acier | 2001 |
| ACOE1197 | Aphididae | Thelaxinae | *Thelaxes* | *dryophila* | (Schrank, 1801) | Coeur d'Acier | 2001 |
| ACOE1198 | Aphididae | Aphidinae | *Brevicoryne* | *brassicae* | (Linnaeus, 1758) | Coeur d'Acier | 2001 |
| ACOE1201 | Aphididae | Phyllaphidinae | *Phyllaphis* | *fagi* | (Linnaeus, 1767) | Coeur d'Acier | 2001 |
| ACOE1206 | Aphididae | Aphidinae | *Phorodon* | *humuli* | (Schrank, 1801) | Coeur d'Acier | 2001 |
| ACOE1208 | Aphididae | Aphidinae | *Brevicoryne* | *brassicae* | (Linnaeus, 1758) | Coeur d'Acier | 2001 |
| ACOE1209 | Aphididae | Aphidinae | *Brachycaudus* | *cardui* | (Linnaeus, 1860) | Coeur d'Acier | 2001 |
| ACOE1211 | Aphididae | Lachninae | *Trama* | *troglodytes* | von Heyden, 1837 | Coeur d'Acier | 2001 |
| ACOE1212 | Aphididae | Aphidinae | *Aphis* | *gossypii* | Glover, 1877 | Coeur d'Acier | 2002 |
| ACOE1215 | Aphididae | Aphidinae | *Uroleucon* | *jaceae* | (Linnaeus, 1758) | Coeur d'Acier | 2001 |
| ACOE1216 | Aphididae | Chaitophorinae | *Periphyllus* | *testudinaceus* | (Fernie, 1852) | Coeur d'Acier | 2001 |
| ACOE1217 | Aphididae | Aphidinae | *Brachycaudus* | *cardui* | (Linnaeus, 1860) | Coeur d'Acier | 2001 |
| ACOE1218 | Aphididae | Aphidinae | *Microlophium* | *carnosum* | (Buckton, 1876) | Coeur d'Acier | 2001 |
| ACOE1219 | Aphididae | Calaphidinae | *Betulaphis* | *brevipilosa* | Börner, 1940 | Coeur d'Acier | 2001 |
| ACOE1220 | Aphididae | Aphidinae | *Dysaphis* | *radicola* | (Mordvilko, 1897) | Coeur d'Acier | 2002 |
| ACOE1222 | Aphididae | Aphidinae | *Hyperomyzus* | *picridis* | (Börner & Blunck, 1916) | Coeur d'Acier | 2002 |
| ACOE1224 | Aphididae | Aphidinae | *Aphis* | *fabae* | Scopoli, 1763 | Coeur d'Acier | 2001 |
| ACOE1225 | Aphididae | Aphidinae | *Macrosiphum* | *euphorbiae* | (Thomas, 1878) | Coeur d'Acier | 2001 |
| ACOE1229 | Aphididae | Calaphidinae | *Euceraphis* | *punctipennis* | Walker, 1870 | Coeur d'Acier | 2001 |
| ACOE1230 | Aphididae | Aphidinae | *Aphis* | *oenotherae* | Oestlund, 1887 | Coeur d'Acier | 2001 |
| ACOE1231 | Aphididae | Aphidinae | *Cavariella* | *theobaldi* | (Gillette & Bragg, 1918) | Coeur d'Acier | 2001 |
| ACOE1232 | Aphididae | Aphidinae | *Macrosiphum* | *euphorbiae* | (Thomas, 1878) | Coeur d'Acier | 2001 |
| ACOE1233 | Aphididae | Calaphidinae | *Eucallipterus* | *tiliae* | (Linnaeus, 1758) | Coeur d'Acier | 2001 |
| ACOE1234 | Aphididae | Calaphidinae | *Pterocallis* | *alni* | (De Geer, 1773) | Coeur d'Acier | 2001 |
| ACOE1238 | Aphididae | Aphidinae | *Aphis* | *taraxacicola* | (Börner, 1940) | Coeur d'Acier | 2001 |
| ACOE1239 | Aphididae | Lachninae | *Cinara* | *pinea* | (Mordvilko, 1895) | Coeur d'Acier | 2001 |
| ACOE1240 | Aphididae | Aphidinae | *Aphis* | *fabae* | Scopoli, 1763 | Coeur d'Acier | 2001 |
| ACOE1241 | Aphididae | Thelaxinae | *Thelaxes* | *dryophila* | (Schrank, 1801) | Coeur d'Acier | 2001 |
| ACOE1242 | Aphididae | Aphidinae | *Myzus* | *cerasi* | (Fabricius, 1775) | Coeur d'Acier | 2001 |
| ACOE1243 | Aphididae | Aphidinae | *Hyalopterus* | *pruni* | (Geoffroy, 1762) | Coeur d'Acier | 2001 |
| ACOE1244 | Aphididae | Aphidinae | *Brachycaudus* | *cardui* | (Linnaeus, 1860) | Coeur d'Acier | 2001 |
| ACOE1245 | Aphididae | Aphidinae | *Aphis* | *coronillae* | Ferrari, 1872 | Coeur d'Acier | 2001 |
| ACOE1246 | Aphididae | Aphidinae | *Aphis* | *pomi* | de Geer, 1773 | Coeur d'Acier | 2001 |
| ACOE1247 | Aphididae | Calaphidinae | *Myzocallis* | *coryli* | (Goeze, 1778) | Coeur d'Acier | 2001 |
| ACOE1248 | Aphididae | Aphidinae | *Macrosiphum* | *euphorbiae* | Passerini, 1860 | Coeur d'Acier | 2002 |
| ACOE1253 | Aphididae | Calaphidinae | *Euceraphis* | *betulae* | (Koch, 1855) | Coeur d'Acier | 2001 |
| ACOE1256 | Aphididae | Aphidinae | *Hyalopterus* | *pruni* | (Geoffroy, 1762) | Coeur d'Acier | 2001 |
| ACOE1257 | Aphididae | Aphidinae | *Aphis* | *pomi* | de Geer, 1773 | Coeur d'Acier | 2001 |
| ACOE1259 | Aphididae | Calaphidinae | *Tuberculatus* | *annulatus* | (Hartig, 1841) | Coeur d'Acier | 2001 |
| ACOE1260 | Aphididae | Thelaxinae | *Thelaxes* | *dryophila* | (Schrank, 1801) | Coeur d'Acier | 2001 |
| ACOE1261 | Aphididae | Lachninae | *Cinara* | *pini* | (Linnaeus, 1758) | Coeur d'Acier | 2001 |
| ACOE1266 | Aphididae | Aphidinae | *Aphis* | *ulmariae* | Schrank, 1801 | Coeur d'Acier | 2001 |
| ACOE1268 | Aphididae | Aphidinae | *Aphis* | *cytisorum* | Hartig, 1841 | Coeur d'Acier | 2001 |
| ACOE1273 | Aphididae | Aphidinae | *Dysaphis* | *angelicae* | (Koch, 1854) | Coeur d'Acier | 2001 |
| ACOE1275 | Aphididae | Aphidinae | *Aphis* | *hederae* | Kaltenbach, 1843 | Coeur d'Acier | 2001 |
| ACOE1276 | Aphididae | Aphidinae | *Aphis* | *spiraecola* | Patch, 1914 | Coeur d'Acier | 2001 |
| ACOE1277 | Aphididae | Aphidinae | *Aphis* | *fabae* | Scopoli, 1763 | Coeur d'Acier | 2001 |
| ACOE1278 | Aphididae | Aphidinae | *Aphis* | *fabae* | Scopoli, 1763 | Coeur d'Acier | 2002 |
| ACOE1279 | Aphididae | Aphidinae | *Aphis* | *fabae* | Scopoli, 1763 | Coeur d'Acier | 2001 |
| ACOE1282 | Aphididae | Calaphidinae | *Tuberculatus* | *annulatus* | (Hartig, 1841) | Coeur d'Acier | 2001 |
| ACOE1283 | Aphididae | Aphidinae | *Nearctaphis* | *bakeri* | (Cowen, 1895) | Coeur d'Acier | 2001 |
| ACOE1285 | Aphididae | Aphidinae | *Aphis* | *pomi* | de Geer, 1773 | Coeur d'Acier | 2001 |
| ACOE1286 | Aphididae | Aphidinae | *Aphis* | *epilobii* | Kaltenbach, 1843 | Coeur d'Acier | 2001 |
| ACOE1287 | Aphididae | Aphidinae | *Aphis* | *sambuci* | Linnaeus, 1758 | Coeur d'Acier | 2001 |
| ACOE1289 | Aphididae | Aphidinae | *Aphis* | *grossulariae* | Kaltenbach, 1843 | Coeur d'Acier | 2001 |
| ACOE1296 | Aphididae | Chaitophorinae | *Chaitophorus* | *populeti* | (Panzer, 1801) | Coeur d'Acier | 2001 |
| ACOE1297 | Aphididae | Aphidinae | *Uroleucon* | *hypochoeridis* | (Fabricius, 1779) | Coeur d'Acier | 2001 |
| ACOE1298 | Aphididae | Aphidinae | *Aphis* | *fabae* | Scopoli, 1763 | Coeur d'Acier | 2001 |
| ACOE1299 | Aphididae | Calaphidinae | *Myzocallis* | *castanicola* | Baker, 1917 | Coeur d'Acier | 2001 |
| ACOE1300 | Aphididae | Aphidinae | *Cavariella* | *theobaldi* | (Gillette & Bragg, 1918) | Coeur d'Acier | 2001 |
| ACOE1302 | Aphididae | Aphidinae | *Aphis* | *jacobaeae* | Schrank, 1801 | Coeur d'Acier | 2001 |
| ACOE1305 | Aphididae | Aphidinae | *Aphis* | *ulicis* | Walker, 1870 | Coeur d'Acier | 2001 |
| ACOE1306 | Aphididae | Aphidinae | *Uroleucon* | *sonchi* | (Linnaeus, 1767) | Coeur d'Acier | 2001 |
| ACOE1307 | Aphididae | Aphidinae | *Uroleucon* | *sonchi* | (Linnaeus, 1767) | Coeur d'Acier | 2001 |
| ACOE1308 | Aphididae | Aphidinae | *Macrosiphoniella* | *artemisiae* | (Boyer de Fonscolombe, 1841) | Coeur d'Acier | 2001 |
| ACOE1309 | Aphididae | Lachninae | *Lachnus* | *roboris* | (Linnaeus, 1758) | Coeur d'Acier | 2001 |
| ACOE1310 | Aphididae | Aphidinae | *Illinoia* | *liriodendri* | (Monell, 1879) | Coeur d'Acier | 2001 |
| ACOE1311 | Aphididae | Aphidinae | *Aphis* | *grossulariae* | Kaltenbach, 1843 | Coeur d'Acier | 2001 |
| ACOE1314 | Aphididae | Aphidinae | *Aphis* | *hederae* | Kaltenbach, 1843 | Coeur d'Acier | 2001 |
| ACOE1316 | Aphididae | Aphidinae | *Aphis* | *spiraecola* | Patch, 1914 | Coeur d'Acier | 2001 |
| ACOE1318 | Aphididae | Aphidinae | *Uroleucon* | *sonchi* | (Linnaeus, 1767) | Coeur d'Acier | 2001 |
| ACOE1320 | Aphididae | Chaitophorinae | *Chaitophorus* | *leucomelas* | Koch, 1854 | Coeur d'Acier | 2001 |
| ACOE1322 | Aphididae | Aphidinae | *Aphis* | *pomi* | de Geer, 1773 | Coeur d'Acier | 2001 |
| ACOE1323 | Aphididae | Chaitophorinae | *Chaitophorus* | *populialbae* | (Panzer, 1801) | Coeur d'Acier | 2001 |
| ACOE1325 | Aphididae | Aphidinae | *Aphis* | *newtoni* | Theobald, 1927 | Coeur d'Acier | 2001 |
| ACOE1326 | Aphididae | Aphidinae | *Aphis* | *oenotherae* | Oestlund, 1887 | Coeur d'Acier | 2001 |
| ACOE1327 | Aphididae | Aphidinae | *Uroleucon* | *sonchi* | (Linnaeus, 1767) | Coeur d'Acier | 2001 |
| ACOE1328 | Aphididae | Aphidinae | *Brachycaudus* | *bicolor* | (Nevsky, 1929) | Coeur d'Acier | 2001 |
| ACOE1329 | Aphididae | Chaitophorinae | *Chaitophorus* | *populialbae* | (Boyer de Fonscolombe, 1841) | Coeur d'Acier | 2001 |
| ACOE1349 | Aphididae | Aphidinae | *Aulacorthum* | *solani* | (Kaltenbach, 1843) | Coeur d'Acier | 2007 |
| ACOE1350 | Aphididae | Aphidinae | *Lipaphis* | *lepidii* | (Nevsky, 1929) | Coeur d'Acier | 2007 |
| ACOE1351 | Aphididae | Aphidinae | *Uroleucon* | *sonchi* | (Linnaeus, 1767) | Coeur d'Acier | 2007 |
| ACOE1352 | Aphididae | Chaitophorinae | *Periphyllus* | *testudinaceus* | (Fernie, 1852) | Coeur d'Acier | 2007 |
| ACOE1353 | Aphididae | Aphidinae | *Dysaphis* | *lappae* | (Koch, 1854) | Coeur d'Acier | 2007 |
| ACOE1354 | Aphididae | Aphidinae | *Brachycaudus* | *cardui* | (Linnaeus, 1860) | Coeur d'Acier | 2007 |
| ACOE1355 | Aphididae | Aphidinae | *Macrosiphum* | *euphorbiae* | (Thomas, 1878) | Coeur d'Acier | 2007 |
| ACOE1359 | Aphididae | Aphidinae | *Hyadaphis* | *passerini* | (del Guercio, 1911) | Coeur d'Acier | 2007 |
| ACOE1360 | Aphididae | Aphidinae | *Aphis* | *fabae* | Scopoli, 1763 | Coeur d'Acier | 2007 |
| ACOE1361 | Aphididae | Calaphidinae | *Euceraphis* | *betulae* | (Koch, 1855) | Coeur d'Acier | 2007 |
| ACOE1362 | Aphididae | Aphidinae | *Aphis* | *grossulariae* | (Börner, 1940) | Coeur d'Acier | 2007 |
| ACOE1364 | Aphididae | Aphidinae | *Aphis* | *intybi* | Koch, 1855 | Coeur d'Acier | 2007 |
| ACOE1367 | Aphididae | Aphidinae | *Aphis* | *viticis* | Ferrari, 1872 | Coeur d'Acier | 2002 |
| ACOE1368 | Aphididae | Aphidinae | *Uroleucon* | *sonchi* | (Linnaeus, 1767) | Coeur d'Acier | 2002 |
| ACOE1369 | Aphididae | Aphidinae | *Aphis* | *nasturtii* | Kaltenbach, 1843 | Coeur d'Acier | 2009 |
| ACOE1370 | Aphididae | Aphidinae | *Aphis* | *fabae* | Scopoli, 1763 | Coeur d'Acier | 2002 |
| ACOE1371 | Aphididae | Aphidinae | *Rhopalosiphum* | *padi* | (Linnaeus, 1758) | Coeur d'Acier | 2002 |
| ACOE1374 | Aphididae | Aphidinae | *Aphis* | *sp.rostellum.like* |  | Coeur d'Acier | 2002 |
| ACOE1375 | Aphididae | Aphidinae | *Dysaphis* | *crataegi* | (Kaltenbach, 1843) | Coeur d'Acier | 2009 |
| ACOE1379 | Aphididae | Aphidinae | *Brevicoryne* | *brassicae* | (Linnaeus, 1758) | Coeur d'Acier | 2002 |
| ACOE1381 | Aphididae | Aphidinae | *Aphis* | *craccivora* | Koch, 1854 | Coeur d'Acier | 2002 |
| ACOE1382 | Aphididae | Aphidinae | *Aphis* | *serpylli* | Koch, 1854 | Coeur d'Acier | 2002 |
| ACOE1383 | Aphididae | Aphidinae | *Uroleucon* | *jaceae* | (Linnaeus, 1758) | Coeur d'Acier | 2007 |
| ACOE1384 | Aphididae | Aphidinae | *Uroleucon* | *hypochoeridis* | (Fabricius, 1779) | Coeur d'Acier | 2002 |
| ACOE1388 | Aphididae | Aphidinae | *Aphis* | *intybi* | Koch, 1855 | Coeur d'Acier | 2002 |
| ACOE1390 | Aphididae | Aphidinae | *Uroleucon* | *aeneum* | (Hille Ris Lambers, 1939) | Coeur d'Acier | 2007 |
| ACOE1391 | Aphididae | Aphidinae | *Uroleucon* | *hypochoeridis* | (Fabricius, 1779) | Coeur d'Acier | 2007 |
| ACOE1393 | Aphididae | Aphidinae | *Brachyunguis* | *tamaricis* | (Lichtenstein, 1885) | Coeur d'Acier | 2002 |
| ACOE1394 | Aphididae | Aphidinae | *Hyalopterus* | *pruni* | (Geoffroy, 1762) | Coeur d'Acier | 2002 |
| ACOE1396 | Aphididae | Aphidinae | *Uroleucon* | *sonchi* | (Linnaeus, 1767) | Coeur d'Acier | 2002 |
| ACOE1399 | Aphididae | Calaphidinae | *Therioaphis* | *riehmi* | (Börner, 1949) | Coeur d'Acier | 2002 |
| ACOE1401 | Aphididae | Aphidinae | *Dysaphis* | *crataegi* | (Kaltenbach, 1843) | Coeur d'Acier | 2002 |
| ACOE1403 | Aphididae | Aphidinae | *Aphis* | *fabae* | Scopoli, 1763 | Coeur d'Acier | 2002 |
| ACOE1405 | Aphididae | Chaitophorinae | *Chaitophorus* | *salijaponicus* | Essig & Kuwana, 1918 | Coeur d'Acier | 2002 |
| ACOE1407 | Aphididae | Aphidinae | *Aphis* | *ruborum* | (Börner, 1932) | Coeur d'Acier | 2002 |
| ACOE1408 | Aphididae | Aphidinae | *Aphis* | *gossypii* | Glover, 1877 | Coeur d'Acier | 2009 |
| ACOE1410 | Aphididae | Aphidinae | *Aphis* | *craccivora* | Koch, 1854 | Coeur d'Acier | 2002 |
| ACOE1411 | Aphididae | Aphidinae | *Cryptomyzus* | *sp.* | (Kaltenbach, 1843) | Coeur d'Acier | 2002 |
| ACOE1421 | Aphididae | Aphidinae | *Hyperomyzus* | *lactucae* | (Linnaeus, 1758) | Coeur d'Acier | 2002 |
| ACOE1422 | Aphididae | Aphidinae | *Aphis* | *gossypii* | Glover, 1877 | Coeur d'Acier | 2002 |
| ACOE1424 | Aphididae | Aphidinae | *Aphis* | *gossypii* | Glover, 1877 | Coeur d'Acier | 2002 |
| ACOE1426 | Aphididae | Aphidinae | *Aphis* | *craccivora* | Koch, 1854 | Coeur d'Acier | 2002 |
| ACOE1427 | Aphididae | Aphidinae | *Dysaphis* | *apiifolia* | Börner, 1931 | Coeur d'Acier | 2002 |
| ACOE1429 | Aphididae | Aphidinae | *Hyperomyzus* | *lactucae* | (Linnaeus, 1758) | Coeur d'Acier | 2002 |
| ACOE1430 | Aphididae | Aphidinae | *Hyalopterus* | *persikonus* | Miller, Lozier & Footit, 2008 | Coeur d'Acier | 2011 |
| ACOE1435 | Aphididae | Aphidinae | *Aphis* | *hederae* | Kaltenbach, 1843 | Coeur d'Acier | 2002 |
| ACOE1436 | Aphididae | Aphidinae | *Macrosiphum* | *rosae* | (Linnaeus, 1758) | Coeur d'Acier | 2002 |
| ACOE1437 | Aphididae | Aphidinae | *Aphis* | *gossypii* | Glover, 1877 | Coeur d'Acier | 2002 |
| ACOE1439 | Aphididae | Aphidinae | *Aphis* | *gossypii* | Glover, 1877 | Coeur d'Acier | 2002 |
| ACOE1440 | Aphididae | Aphidinae | *Aphis* | *craccivora* | Koch, 1854 | Coeur d'Acier | 2002 |
| ACOE1441 | Aphididae | Aphidinae | *Hayhurstia* | *atriplicis* | (Linnaeus, 1761) | Coeur d'Acier | 2002 |
| ACOE1442 | Aphididae | Aphidinae | *Aphis* | *gossypii* | Glover, 1877 | Coeur d'Acier | 2002 |
| ACOE1443 | Aphididae | Aphidinae | *Aphis* | *gossypii* | Glover, 1877 | Coeur d'Acier | 2002 |
| ACOE1444 | Aphididae | Aphidinae | *Aphis* | *ruborum* | (Börner, 1932) | Coeur d'Acier | 2002 |
| ACOE1445 | Aphididae | Aphidinae | *Aphis* | *gossypii* | Glover, 1877 | Coeur d'Acier | 2002 |
| ACOE1446 | Aphididae | Aphidinae | *Aphis* | *craccivora* | Koch, 1854 | Coeur d'Acier | 2002 |
| ACOE1447 | Aphididae | Aphidinae | *Macrosiphum* | *rosae* | (Linnaeus, 1758) | Coeur d'Acier | 2002 |
| ACOE1448 | Aphididae | Aphidinae | *Melanaphis* | *pyraria* | (Passerini, 1861) | Coeur d'Acier | 2002 |
| ACOE1449 | Aphididae | Aphidinae | *Aphis* | *brotericola* | Linnaeus, 1758 | Coeur d'Acier | 2002 |
| ACOE1450 | Aphididae | Aphidinae | *Aphis* | *fabae* | Scopoli, 1763 | Coeur d'Acier | 2002 |
| ACOE1451 | Aphididae | Aphidinae | *Aphis* | *craccae* | Linnaeus, 1758 | Coeur d'Acier | 2002 |
| ACOE1452 | Aphididae | Thelaxinae | *Thelaxes* | *suberi* | (del Guercio, 1911) | Coeur d'Acier | 2002 |
| ACOE1453 | Aphididae | Aphidinae | *Brachycaudus* | *tragopogonis* | (Kaltenbach, 1843) | Coeur d'Acier | 2002 |
| ACOE1454 | Aphididae | Aphidinae | *Aphis* | *fabae* | Scopoli, 1763 | Coeur d'Acier | 2002 |
| ACOE1455 | Aphididae | Aphidinae | *Brachycaudus* | *helichrysi* | (Kaltenbach, 1843) | Coeur d'Acier | 2002 |
| ACOE1456 | Aphididae | Aphidinae | *Aphis* | *gossypii* | Glover, 1877 | Coeur d'Acier | 2002 |
| ACOE1457 | Aphididae | Aphidinae | *Aphis* | *vitalbae* | Ferrari, 1872 | Coeur d'Acier | 2002 |
| ACOE1458 | Aphididae | Aphidinae | *Brachycaudus* | *bicolor* | (Nevsky, 1929) | Coeur d'Acier | 2002 |
| ACOE1461 | Aphididae | Aphidinae | *Dysaphis* | *reaumuri* | (Mordvilko, 1928) | Coeur d'Acier | 2002 |
| ACOE1463 | Aphididae | Aphidinae | *Dysaphis* | *crataegi* | (Kaltenbach, 1843) | Coeur d'Acier | 2002 |
| ACOE1464 | Aphididae | Aphidinae | *Aphis* | *cisticola* | Leclant & Remaudière, 1972 | Coeur d'Acier | 2002 |
| ACOE1467 | Aphididae | Aphidinae | *Aphis* | *origani* | Linnaeus, 1758 | Coeur d'Acier | 2002 |
| ACOE1468 | Aphididae | Aphidinae | *Dysaphis* | *crataegi* | (Kaltenbach, 1843) | Coeur d'Acier | 2002 |
| ACOE1470 | Aphididae | Aphidinae | *Aphis* | *spiraecola* | Patch, 1914 | Coeur d'Acier | 2002 |
| ACOE1471 | Aphididae | Aphidinae | *Aphis* | *spiraecola* | Patch, 1914 | Coeur d'Acier | 2002 |
| ACOE1472 | Aphididae | Calaphidinae | *Panaphis* | *juglandis* | (Goeze, 1778) | Coeur d'Acier | 2002 |
| ACOE1473 | Aphididae | Aphidinae | *Aphis* | *sp.rostella.like* |  | Coeur d'Acier | 2002 |
| ACOE1474 | Aphididae | Aphidinae | *Brachycaudus* | *populi* | (del Guercio, 1911) | Coeur d'Acier | 2002 |
| ACOE1475 | Aphididae | Aphidinae | *Aphis* | *vallei* | Hille Ris Lambers & Stroyan, 1959 | Coeur d'Acier | 2002 |
| ACOE1478 | Aphididae | Lachninae | *Lachnus* | *roboris* | (Linnaeus, 1758) | Coeur d'Acier | 2002 |
| ACOE1479 | Aphididae | Chaitophorinae | *Periphyllus* | *sp.* | van der Hoeven, 1863 | Coeur d'Acier | 2002 |
| ACOE1480 | Aphididae | Aphidinae | *Acyrthosiphon* | *caraganae* | (Cholodkovsky, 1907) | Coeur d'Acier | 2002 |
| ACOE1481 | Aphididae | Aphidinae | *Aphis* | *sp.frangulae-like* |  | Coeur d'Acier | 2002 |
| ACOE1482 | Aphididae | Aphidinae | *Aphis* | *punicae* | Passerini, 1863 | Coeur d'Acier | 2002 |
| ACOE1484 | Aphididae | Aphidinae | *Aphis* | *spiraecola* | Patch, 1914 | Coeur d'Acier | 2002 |
| ACOE1485 | Aphididae | Aphidinae | *Aphis* | *chloris* | Koch, 1854 | Coeur d'Acier | 2002 |
| ACOE1487 | Aphididae | Aphidinae | *Sitobion* | *fragariae* | (Walker, 1848) | Coeur d'Acier | 2002 |
| ACOE1489 | Aphididae | Chaitophorinae | *Chaitophorus* | *nigricantis* | Pintera, 1987 | Coeur d'Acier | 2007 |
| ACOE1490 | Aphididae | Aphidinae | *Melanaphis* | *donacis* | (Passerini, 1862) | Coeur d'Acier | 2002 |
| ACOE1493 | Aphididae | Chaitophorinae | *Chaitophorus* | *leucomelas* | Koch, 1854 | Coeur d'Acier | 2002 |
| ACOE1494 | Aphididae | Aphidinae | *Aphis* | *ruborum* | (Börner, 1932) | Coeur d'Acier | 2002 |
| ACOE1495 | Aphididae | Aphidinae | *Aphis* | *viticis* | Ferrari, 1872 | Coeur d'Acier | 2002 |
| ACOE1496 | Aphididae | Aphidinae | *Aphis* | *fabae* | Scopoli, 1763 | Coeur d'Acier | 2002 |
| ACOE1500 | Aphididae | Aphidinae | *Uroleucon* | *rapunculoidis* | (Börner, 1939) | Coeur d'Acier | 2002 |
| ACOE1503 | Aphididae | Aphidinae | *Aphis* | *craccae* | Linnaeus, 1758 | Coeur d'Acier | 2002 |
| ACOE1504 | Aphididae | Calaphidinae | *Myzocallis* | *castanicola* | Baker, 1917 | Coeur d'Acier | 2002 |
| ACOE1505 | Aphididae | Aphidinae | *Aphis* | *cytisorum* | Linnaeus, 1758 | Coeur d'Acier | 2002 |
| ACOE1507 | Aphididae | Aphidinae | *Aphis* | *vitalbae* | Ferrari, 1872 | Coeur d'Acier | 2002 |
| ACOE1508 | Aphididae | Chaitophorinae | *Chaitophorus* | *populialbae* | (Boyer de Fonscolombe, 1841) | Coeur d'Acier | 2002 |
| ACOE1509 | Aphididae | Aphidinae | *Aphis* | *craccivora* | Koch, 1854 | Coeur d'Acier | 2002 |
| ACOE1510 | Aphididae | Chaitophorinae | *Chaitophorus* | *populeti* | (Panzer, 1801) | Coeur d'Acier | 2002 |
| ACOE1511 | Aphididae | Aphidinae | *Aphis* | *sp.frangulae-like* |  | Coeur d'Acier | 2002 |
| ACOE1512 | Aphididae | Thelaxinae | *Thelaxes* | *suberi* | (del Guercio, 1911) | Coeur d'Acier | 2002 |
| ACOE1513 | Aphididae | Aphidinae | *Aphis* | *origani* | Passerini, 1860 | Coeur d'Acier | 2002 |
| ACOE1514 | Aphididae | Aphidinae | *Aphis* | *sp.rostella.like* |  | Coeur d'Acier | 2002 |
| ACOE1515 | Aphididae | Aphidinae | *Aphis* | *cytisorum* | Hartig, 1841 | Coeur d'Acier | 2002 |
| ACOE1516 | Aphididae | Aphidinae | *Aphis* | *fabae* | Scopoli, 1763 | Coeur d'Acier | 2002 |
| ACOE1519 | Aphididae | Aphidinae | *Staticobium* | *sp.* | Mordvilko, 1914 | Coeur d'Acier | 2002 |
| ACOE1521 | Aphididae | Aphidinae | *Uroleucon* | *sonchi* | (Linnaeus, 1767) | Coeur d'Acier | 2007 |
| ACOE1522 | Aphididae | Aphidinae | *Aphis* | *craccivora* | Koch, 1854 | Coeur d'Acier | 2002 |
| ACOE1524 | Aphididae | Chaitophorinae | *Chaitophorus* | *leucomelas* | Koch, 1854 | Coeur d'Acier | 2002 |
| ACOE1525 | Aphididae | Aphidinae | *Aphis* | *nerii* | Boyer de Fonscolombe, 1841 | Coeur d'Acier | 2002 |
| ACOE1526 | Aphididae | Aphidinae | *Uroleucon* | *hypochoeridis* | (Fabricius, 1779) | Coeur d'Acier | 2007 |
| ACOE1527 | Aphididae | Aphidinae | *Aphis* | *craccivora* | Koch, 1854 | Coeur d'Acier | 2002 |
| ACOE1537 | Aphididae | Aphidinae | *Aphis* | *spiraecola* | Patch, 1914 | Coeur d'Acier | 2002 |
| ACOE1539 | Aphididae | Aphidinae | *Macrosiphum* | *euphorbiae* | (Thomas, 1878) | Coeur d'Acier | 2002 |
| ACOE1540 | Aphididae | Aphidinae | *Lipaphis* | *erysimi* | (Kaltenbach, 1843) | Coeur d'Acier | 2002 |
| ACOE1542 | Aphididae | Lachninae | *Cinara* | *palaestinensis* | Hille Ris Lambers, 1938 | Coeur d'Acier | 2002 |
| ACOE1544 | Aphididae | Aphidinae | *Aphis* | *craccivora* | Koch, 1854 | Coeur d'Acier | 2002 |
| ACOE1545 | Aphididae | Aphidinae | *Aphis* | *spiraecola* | Patch, 1914 | Coeur d'Acier | 2002 |
| ACOE1546 | Aphididae | Chaitophorinae | *Chaitophorus* | *leucomelas* | Koch, 1854 | Coeur d'Acier | 2002 |
| ACOE1547 | Aphididae | Pterocommatinae | *Pterocomma* | *populeum* | (Kaltenbach, 1843) | Coeur d'Acier | 2002 |
| ACOE1549 | Aphididae | Aphidinae | *Uroleucon* | *hypochoeridis* | (Fabricius, 1779) | Coeur d'Acier | 2002 |
| ACOE1550 | Aphididae | Aphidinae | *Aphis* | *hypochoeridis* | (Börner, 1940) | Coeur d'Acier | 2002 |
| ACOE1551 | Aphididae | Aphidinae | *Aphis* | *craccivora* | Koch, 1854 | Coeur d'Acier | 2002 |
| ACOE1552 | Aphididae | Aphidinae | *Aphis* | *sanguisorbae* | Schrank, 1801 | Coeur d'Acier | 2002 |
| ACOE1553 | Aphididae | Aphidinae | *Aphis* | *fabae* | Scopoli, 1763 | Coeur d'Acier | 2002 |
| ACOE1554 | Aphididae | Thelaxinae | *Thelaxes* | *suberi* | (del Guercio, 1911) | Coeur d'Acier | 2002 |
| ACOE1558 | Aphididae | Aphidinae | *Semiaphis* | *dauci* | (Fabricius, 1775) | Coeur d'Acier | 2002 |
| ACOE1560 | Aphididae | Aphidinae | *Aphis* | *ruborum* | (Börner, 1932) | Coeur d'Acier | 2002 |
| ACOE1561 | Aphididae | Aphidinae | *Sitobion* | *fragariae* | (Walker, 1848) | Coeur d'Acier | 2002 |
| ACOE1562 | Aphididae | Aphidinae | *Aphis* | *fabae* | Linnaeus, 1758 | Coeur d'Acier | 2002 |
| ACOE1563 | Aphididae | Aphidinae | *Uroleucon* | *hypochoeridis* | (Fabricius, 1779) | Coeur d'Acier | 2007 |
| ACOE1565 | Aphididae | Chaitophorinae | *Chaitophorus* | *salijaponicus* | Essig & Kuwana, 1918 | Coeur d'Acier | 2007 |
| ACOE1566 | Aphididae | Chaitophorinae | *Chaitophorus* | *salijaponicus* | Essig & Kuwana, 1918 | Coeur d'Acier | 2007 |
| ACOE1567 | Aphididae | Chaitophorinae | *Chaitophorus* | *salijaponicus* | Mordvilko, 1929 | Coeur d'Acier | 2002 |
| ACOE1568 | Aphididae | Aphidinae | *Microlophium* | *carnosum* | (Buckton, 1876) | Coeur d'Acier | 2002 |
| ACOE1569 | Aphididae | Aphidinae | *Aphis* | *serpylli* | Koch, 1854 | Coeur d'Acier | 2002 |
| ACOE1570 | Aphididae | Aphidinae | *Aphis* | *fabae* | Scopoli, 1763 | Coeur d'Acier | 2002 |
| ACOE1572 | Aphididae | Aphidinae | *Aphis* | *urticata* | Gmelin, 1790 | Coeur d'Acier | 2002 |
| ACOE1575 | Aphididae | Aphidinae | *Aphis* | *sambuci* | Linnaeus, 1758 | Coeur d'Acier | 2002 |
| ACOE1576 | Aphididae | Lachninae | *Lachnus* | *roboris* | (Linnaeus, 1758) | Coeur d'Acier | 2002 |
| ACOE1578 | Aphididae | Aphidinae | *Aphis* | *mamonthovae* | Davletschina, 1964 | Coeur d'Acier | 2002 |
| ACOE1580 | Aphididae | Aphidinae | *Hyperomyzus* | *picridis* | (Börner & Blunck, 1916) | Coeur d'Acier | 2002 |
| ACOE1585 | Aphididae | Aphidinae | *Aphis* | *parietariae* | Theobald, 1923 | Coeur d'Acier | 2002 |
| ACOE1586 | Aphididae | Aphidinae | *Uroleucon* | *sp.* | Mordvilko, 1914 | Coeur d'Acier | 2007 |
| ACOE1587 | Aphididae | Aphidinae | *Aphis* | *fabae* | Scopoli, 1763 | Coeur d'Acier | 2002 |
| ACOE1588 | Aphididae | Aphidinae | *Aphis* | *sp.frangulae-like* |  | Coeur d'Acier | 2002 |
| ACOE1590 | Aphididae | Aphidinae | *Cavariella* | *theobaldi* | (Gillette & Bragg, 1918) | Coeur d'Acier | 2002 |
| ACOE1591 | Aphididae | Aphidinae | *Dysaphis* | *tulipae* | (Boyer de Fonscolombe, 1841) | Coeur d'Acier | 2002 |
| ACOE1592 | Aphididae | Aphidinae | *Aphis* | *sambuci* | Linnaeus, 1758 | Coeur d'Acier | 2002 |
| ACOE1593 | Aphididae | Aphidinae | *Dysaphis* | *radicola* | (Mordvilko, 1897) | Coeur d'Acier | 2002 |
| ACOE1595 | Aphididae | Aphidinae | *Aulacorthum* | *solani* | (Kaltenbach, 1843) | Coeur d'Acier | 2002 |
| ACOE1599 | Aphididae | Aphidinae | *Brachycaudus* | *cardui* | (Linnaeus, 1860) | Coeur d'Acier | 2002 |
| ACOE1601 | Aphididae | Aphidinae | *Aphis* | *sp.frangulae-like* |  | Coeur d'Acier | 2002 |
| ACOE1602 | Aphididae | Aphidinae | *Acyrthosiphon* | *malvae* | (Mosley, 1841) | Coeur d'Acier | 2002 |
| ACOE1605 | Aphididae | Aphidinae | *Acyrthosiphon* | *pisum* | (Harris, 1776) | Coeur d'Acier | 2002 |
| ACOE1606 | Aphididae | Aphidinae | *Acyrthosiphon* | *malvae* | (Mosley, 1841) | Coeur d'Acier | 2002 |
| ACOE1607 | Aphididae | Aphidinae | *Aulacorthum* | *solani* | (Kaltenbach, 1843) | Coeur d'Acier | 2002 |
| ACOE1609 | Aphididae | Aphidinae | *Aphis* | *fabae* | Scopoli, 1763 | Coeur d'Acier | 2007 |
| ACOE1610 | Aphididae | Aphidinae | *Aphis* | *salicariae* | Koch, 1855 | Coeur d'Acier | 2003 |
| ACOE1611 | Aphididae | Aphidinae | *Dysaphis* | *apiifolia* | (Theobald, 1923) | Coeur d'Acier | 2007 |
| ACOE1612 | Aphididae | Aphidinae | *Aphis* | *galiiscabri* | Schrank, 1801 | Coeur d'Acier | 2007 |
| ACOE1613 | Aphididae | Aphidinae | *Dysaphis* | *crataegi* | (Kaltenbach, 1843) | Coeur d'Acier | 2007 |
| ACOE1616 | Aphididae | Aphidinae | *Aphis* | *craccivora* | Koch, 1854 | Coeur d'Acier | 2003 |
| ACOE1618 | Aphididae | Chaitophorinae | *Sipha* | *maydis* | Passerini, 1860 | Coeur d'Acier | 2003 |
| ACOE1619 | Aphididae | Aphidinae | *Aphis* | *confusa* | Walker, 1849 | Coeur d'Acier | 2003 |
| ACOE1620 | Aphididae | Aphidinae | *Nearctaphis* | *bakeri* | (Cowen, 1895) | Coeur d'Acier | 2007 |
| ACOE1621 | Aphididae | Aphidinae | *Uroleucon* | *aeneum* | (Hille Ris Lambers, 1939) | Coeur d'Acier | 2007 |
| ACOE1623 | Aphididae | Calaphidinae | *Eucallipterus* | *tiliae* | (Linnaeus, 1758) | Coeur d'Acier | 2007 |
| ACOE1624 | Aphididae | Aphidinae | *Aphis* | *proffti* | (Börner, 1942) | Coeur d'Acier | 2003 |
| ACOE1625 | Aphididae | Aphidinae | *Aphis* | *fabae* | Scopoli, 1763 | Coeur d'Acier | 2007 |
| ACOE1626 | Aphididae | Aphidinae | *Aphis* | *epilobii* | Kaltenbach, 1843 | Coeur d'Acier | 2007 |
| ACOE1628 | Aphididae | Aphidinae | *Aphis* | *crepidis* | (Börner, 1940) | Coeur d'Acier | 2007 |
| ACOE1629 | Aphididae | Aphidinae | *Aphis* | *pomi* | de Geer, 1773 | Coeur d'Acier | 2003 |
| ACOE1630 | Aphididae | Chaitophorinae | *Chaitophorus* | *capreae* | (Mosley, 1841) | Coeur d'Acier | 2007 |
| ACOE1632 | Aphididae | Aphidinae | *Aphis* | *plantaginis* | Goeze, 1778 | Coeur d'Acier | 2007 |
| ACOE1634 | Aphididae | Aphidinae | *Aphis* | *fabae* | Scopoli, 1763 | Coeur d'Acier | 2007 |
| ACOE1635 | Aphididae | Aphidinae | *Aphis* | *epilobii* | Kaltenbach, 1843 | Coeur d'Acier | 2007 |
| ACOE1636 | Aphididae | Aphidinae | *Aphis* | *oenotherae* | Oestlund, 1887 | Coeur d'Acier | 2007 |
| ACOE1637 | Aphididae | Aphidinae | *Aphis* | *pomi* | de Geer, 1773 | Coeur d'Acier | 2007 |
| ACOE1639 | Aphididae | Aphidinae | *Hayhurstia* | *atriplicis* | (Linnaeus, 1761) | Coeur d'Acier | 2007 |
| ACOE1641 | Aphididae | Aphidinae | *Aphis* | *fabae* | Scopoli, 1763 | Coeur d'Acier | 2007 |
| ACOE1642 | Aphididae | Aphidinae | *Aphis* | *fabae* | Scopoli, 1763 | Coeur d'Acier | 2007 |
| ACOE1643 | Aphididae | Aphidinae | *Uroleucon* | *sonchi* | (Linnaeus, 1767) | Coeur d'Acier | 2007 |
| ACOE1644 | Aphididae | Aphidinae | *Hyperomyzus* | *lactucae* | (Linnaeus, 1758) | Coeur d'Acier | 2007 |
| ACOE1646 | Aphididae | Aphidinae | *Aphis* | *hederae* | Kaltenbach, 1843 | Coeur d'Acier | 2003 |
| ACOE1647 | Aphididae | Aphidinae | *Dysaphis* | *tulipae* | (Boyer de Fonscolombe, 1841) | Coeur d'Acier | 2003 |
| ACOE1648 | Aphididae | Aphidinae | *Aphis* | *grossulariae* | Kaltenbach, 1843 | Coeur d'Acier | 2007 |
| ACOE1650 | Aphididae | Aphidinae | *Macrosiphum* | *euphorbiae* | (Thomas, 1878) | Coeur d'Acier | 2007 |
| ACOE1651 | Aphididae | Aphidinae | *Macrosiphoniella* | *millefolii* | (de Geer, 1773) | Coeur d'Acier | 2007 |
| ACOE1652 | Aphididae | Aphidinae | *Hyalopterus* | *pruni* | (Geoffroy, 1762) | Coeur d'Acier | 2007 |
| ACOE1655 | Aphididae | Aphidinae | *Aphis* | *fabae* | Scopoli, 1763 | Coeur d'Acier | 2007 |
| ACOE1656 | Aphididae | Aphidinae | *Hyperomyzus* | *lactucae* | (Linnaeus, 1758) | Coeur d'Acier | 2007 |
| ACOE1657 | Aphididae | Aphidinae | *Aphis* | *plantaginis* | Goeze, 1778 | Coeur d'Acier | 2007 |
| ACOE1658 | Aphididae | Aphidinae | *Aphis* | *mamonthovae* | Davletschina, 1964 | Coeur d'Acier | 2007 |
| ACOE1659 | Aphididae | Aphidinae | *Aphis* | *spiraecola* | Patch, 1914 | Coeur d'Acier | 2007 |
| ACOE1660 | Aphididae | Chaitophorinae | *Chaitophorus* | *leucomelas* | Koch, 1854 | Coeur d'Acier | 2007 |
| ACOE1663 | Aphididae | Aphidinae | *Aphis* | *pomi* | de Geer, 1773 | Coeur d'Acier | 2007 |
| ACOE1664 | Aphididae | Calaphidinae | *Panaphis* | *juglandis* | (Goeze, 1778) | Coeur d'Acier | 2007 |
| ACOE1665 | Aphididae | Aphidinae | *Aphis* | *gossypii* | Glover, 1877 | Coeur d'Acier | 2007 |
| ACOE1666 | Aphididae | Aphidinae | *Semiaphis* | *dauci* | (Fabricius, 1775) | Coeur d'Acier | 2007 |
| ACOE1668 | Aphididae | Aphidinae | *Aphis* | *coronillae* | Ferrari, 1872 | Coeur d'Acier | 2007 |
| ACOE1669 | Aphididae | Aphidinae | *Aphis* | *coronillae* | Ferrari, 1872 | Coeur d'Acier | 2007 |
| ACOE1670 | Aphididae | Aphidinae | *Aphis* | *lambersi* | (Börner, 1940) | Coeur d'Acier | 2007 |
| ACOE1671 | Aphididae | Aphidinae | *Semiaphis* | *dauci* | (Fabricius, 1775) | Coeur d'Acier | 2007 |
| ACOE1673 | Aphididae | Aphidinae | *Hydaphias* | *molluginis* | Börner, 1939 | Coeur d'Acier | 2007 |
| ACOE1674 | Aphididae | Aphidinae | *Roepkea* | *marchali* | (Börner, 1931) | Coeur d'Acier | 2007 |
| ACOE1681 | Aphididae | Aphidinae | *Brachycaudus* | *helichrysi* | (Kaltenbach, 1843) | Coeur d'Acier | 2005 |
| ACOE1687 | Aphididae | Aphidinae | *Myzus* | *persicae* | (Sulzer, 1776) | Coeur d'Acier | 2005 |
| ACOE1693 | Aphididae | Aphidinae | *Myzus* | *cerasi* | (Fabricius, 1775) | Coeur d'Acier | 2007 |
| ACOE1696 | Aphididae | Aphidinae | *Brachycaudus* | *persicae* | (Passerini, 1860) | Coeur d'Acier | 2005 |
| ACOE1700 | Aphididae | Aphidinae | *Myzus* | *cerasi* | (Fabricius, 1775) | Coeur d'Acier | 2007 |
| ACOE1704 | Aphididae | Aphidinae | *Brachycaudus* | *lychnidis* | (Linnaeus, 1758) | Coeur d'Acier | 2005 |
| ACOE1706 | Aphididae | Aphidinae | *Brachycaudus* | *amygdalinus* | (Schouteden, 1905) | Coeur d'Acier | 2005 |
| ACOE1707 | Aphididae | Aphidinae | *Hyalopterus* | *amygdali* | (Blanchard E., 1840) | Coeur d'Acier | 2005 |
| ACOE1710 | Aphididae | Aphidinae | *Brachycaudus* | *amygdalinus* | (Schouteden, 1905) | Coeur d'Acier | 2005 |
| ACOE1714 | Aphididae | Aphidinae | *Myzus* | *lythri* | (Schrank, 1801) | Coeur d'Acier | 2007 |
| ACOE1715 | Aphididae | Aphidinae | *Brachycaudus* | *tragopogonis* | (Kaltenbach, 1843) | Coeur d'Acier | 2005 |
| ACOE1716 | Aphididae | Aphidinae | *Brachycaudus* | *helichrysi* | (Kaltenbach, 1843) | Coeur d'Acier | 2005 |
| ACOE1721 | Aphididae | Aphidinae | *Macrosiphum* | *rosae* | (Linnaeus, 1758) | Coeur d'Acier | 2007 |
| ACOE1722 | Aphididae | Aphidinae | *Aphis* | *urticata* | Gmelin, 1790 | Coeur d'Acier | 2007 |
| ACOE1725 | Aphididae | Aphidinae | *Brachycaudus* | *helichrysi* | (Kaltenbach, 1843) | Coeur d'Acier | 2007 |
| ACOE1729 | Aphididae | Aphidinae | *Myzus* | *cerasi* | (Fabricius, 1775) | Coeur d'Acier | 2007 |
| ACOE1730 | Aphididae | Aphidinae | *Brachycaudus* | *schwartzi* | (Börner, 1931) | Coeur d'Acier | 2005 |
| ACOE1731 | Aphididae | Aphidinae | *Myzus* | *cerasi* | (Fabricius, 1775) | Coeur d'Acier | 2007 |
| ACOE1732 | Aphididae | Aphidinae | *Aphis* | *acetosae* | Linnaeus, 1761 | Coeur d'Acier | 2007 |
| ACOE1734 | Aphididae | Pterocommatinae | *Pterocomma* | *populeum* | (Kaltenbach, 1843) | Coeur d'Acier | 2007 |
| ACOE1736 | Aphididae | Aphidinae | *Brachycaudus* | *persicae* | (Passerini, 1860) | Coeur d'Acier | 2005 |
| ACOE1737 | Aphididae | Aphidinae | *Brachycaudus* | *schwartzi* | (Börner, 1931) | Coeur d'Acier | 2007 |
| ACOE1744 | Aphididae | Aphidinae | *Sitobion* | *fragariae* | (Walker, 1848) | Coeur d'Acier | 2007 |
| ACOE1745 | Aphididae | Aphidinae | *Cryptomyzus* | *ribis* | (Linnaeus, 1758) | Coeur d'Acier | 2007 |
| ACOE1751 | Aphididae | Aphidinae | *Brachycaudus* | *lateralis* | (Walker, 1848) | Coeur d'Acier | 2005 |
| ACOE1755 | Aphididae | Aphidinae | *Aulacorthum* | *solani* | (Thomas, 1878) | Coeur d'Acier | 2007 |
| ACOE1759 | Aphididae | Chaitophorinae | *Periphyllus* | *testudinaceus* | (Fernie, 1852) | Coeur d'Acier | 2007 |
| ACOE1760 | Aphididae | Aphidinae | *Brachycaudus* | *populi* | (del Guercio, 1911) | Coeur d'Acier | 2007 |
| ACOE1761 | Aphididae | Aphidinae | *Brachycaudus* | *tragopogonis* | (Kaltenbach, 1843) | Coeur d'Acier | 2007 |
| ACOE1762 | Aphididae | Aphidinae | *Brachycaudus* | *lychnidis* | (Linnaeus, 1758) | Coeur d'Acier | 2007 |
| ACOE1768 | Aphididae | Aphidinae | *Macrosiphum* | *stellariae* | Theobald, 1913 | Coeur d'Acier | 2007 |
| ACOE1769 | Aphididae | Aphidinae | *Aphis* | *fabae* | Scopoli, 1763 | Coeur d'Acier | 2007 |
| ACOE1771 | Aphididae | Aphidinae | *Brachycaudus* | *linariae* | Stroyan, 1950 | Coeur d'Acier | 2007 |
| ACOE1772 | Aphididae | Aphidinae | *Brachycaudus* | *cerinthis* | Bozhko, 1961 | Coeur d'Acier | 2007 |
| ACOE1777 | Aphididae | Aphidinae | *Macrosiphum* | *albifrons* | Essig, 1911 | Coeur d'Acier | 2007 |
| ACOE1778 | Aphididae | Aphidinae | *Aphis* | *idaei* | van der Goot, 1912 | Coeur d'Acier | 2007 |
| ACOE1779 | Aphididae | Aphidinae | *Aphis* | *pomi* | de Geer, 1773 | Coeur d'Acier | 2007 |
| ACOE1780 | Aphididae | Calaphidinae | *Euceraphis* | *betulae* | (Koch, 1855) | Coeur d'Acier | 2006 |
| ACOE1781 | Aphididae | Aphidinae | *Aphis* | *grossulariae* | Kaltenbach, 1843 | Coeur d'Acier | 2007 |
| ACOE1782 | Aphididae | Aphidinae | *Aphis* | *epilobii* | Kaltenbach, 1843 | Coeur d'Acier | 2006 |
| ACOE1783 | Aphididae | Aphidinae | *Aphis* | *plantaginis* | Goeze, 1778 | Coeur d'Acier | 2006 |
| ACOE1784 | Aphididae | Aphidinae | *Aphis* | *spiraephaga* | Müller F.P., 1961 | Coeur d'Acier | 2007 |
| ACOE1785 | Aphididae | Lachninae | *Cinara* | *fresai* | Blanchard E.E., 1939 | Coeur d'Acier | 2006 |
| ACOE1786 | Aphididae | Aphidinae | *Uroleucon* | *jaceae* | (Linnaeus, 1758) | Coeur d'Acier | 2006 |
| ACOE1787 | Aphididae | Aphidinae | *Hyadaphis* | *passerini* | (del Guercio, 1911) | Coeur d'Acier | 2006 |
| ACOE1790 | Aphididae | Aphidinae | *Brachycaudus* | *aconiti* | (Mordvilko, 1928) | Coeur d'Acier | 2007 |
| ACOE1791 | Aphididae | Aphidinae | *Dysaphis* | *aucupariae* | (Buckton, 1879) | Coeur d'Acier | 2006 |
| ACOE1792 | Aphididae | Aphidinae | *Dysaphis* | *plantaginea* | (Passerini, 1860) | Coeur d'Acier | 2006 |
| ACOE1794 | Aphididae | Aphidinae | *Brachycaudus* | *lateralis* | (Walker, 1848) | Coeur d'Acier | 2006 |
| ACOE1926 | Aphididae | Aphidinae | *Brachycaudus* | *helichrysi* | (Kaltenbach, 1843) | Coeur d'Acier | 2006 |
| ACOE1928 | Aphididae | Aphidinae | *Phorodon* | *humuli* | (Schrank, 1801) | Coeur d'Acier | 2006 |
| ACOE1929 | Aphididae | Aphidinae | *Rhopalosiphum* | *nymphaeae* | (Linnaeus, 1761) | Coeur d'Acier | 2006 |
| ACOE1930 | Aphididae | Aphidinae | *Brachycaudus* | *helichrysi* | (Kaltenbach, 1843) | Coeur d'Acier | 2006 |
| ACOE1931 | Aphididae | Aphidinae | *Macrosiphum* | *euphorbiae* | (Thomas, 1878) | Coeur d'Acier | 2006 |
| ACOE1932 | Aphididae | Aphidinae | *Dysaphis* | *plantaginea* | (Passerini, 1860) | Coeur d'Acier | 2006 |
| ACOE1933 | Aphididae | Chaitophorinae | *Sipha* | *elegans* | del Guercio, 1905 | Coeur d'Acier | 2006 |
| ACOE1934 | Aphididae | Aphidinae | *Aulacorthum* | *solani* | (Kaltenbach, 1843) | Coeur d'Acier | 2006 |
| ACOE1935 | Aphididae | Aphidinae | *Aphis* | *intybi* | Koch, 1855 | Coeur d'Acier | 2006 |
| ACOE1936 | Aphididae | Chaitophorinae | *Sipha* | *maydis* | Passerini, 1860 | Coeur d'Acier | 2006 |
| ACOE1937 | Aphididae | Aphidinae | *Brachycaudus* | *helichrysi* | (Kaltenbach, 1843) | Coeur d'Acier | 2006 |
| ACOE1938 | Aphididae | Aphidinae | *Brachycaudus* | *linariae* | Stroyan, 1950 | Coeur d'Acier | 2006 |
| ACOE1939 | Aphididae | Aphidinae | *Brachycaudus* | *helichrysi* | (Kaltenbach, 1843) | Coeur d'Acier | 2006 |
| ACOE1940 | Aphididae | Aphidinae | *Neotoxoptera* | *formosana* | (Takahashi, 1921) | Coeur d'Acier | 2006 |
| ACOE1942 | Aphididae | Aphidinae | *Aphis* | *nerii* | Boyer de Fonscolombe, 1841 | Coeur d'Acier | 2006 |
| ACOE1943 | Aphididae | Aphidinae | *Macrosiphum* | *rosae* | (Linnaeus, 1758) | Coeur d'Acier | 2006 |
| ACOE1944 | Aphididae | Aphidinae | *Aphis* | *nasturtii* | Kaltenbach, 1843 | Coeur d'Acier | 2007 |
| ACOE1945 | Aphididae | Eriosomatinae | *Aploneura* | *lentisci* | (Passerini, 1856) | Coeur d'Acier | 2007 |
| ACOE1946 | Aphididae | Aphidinae | *Brachycaudus* | *helichrysi* | (Kaltenbach, 1843) | Coeur d'Acier | 2006 |
| ACOE1947 | Aphididae | Aphidinae | *Aphis* | *fabae* | Scopoli, 1763 | Coeur d'Acier | 2006 |
| ACOE1948 | Aphididae | Aphidinae | *Myzus* | *persicae* | (Sulzer, 1776) | Coeur d'Acier | 2006 |
| ACOE1949 | Aphididae | Aphidinae | *Rhopalosiphum* | *maidis* | (Fitch, 1856) | Coeur d'Acier | 2006 |
| ACOE1950 | Aphididae | Aphidinae | *Semiaphis* | *dauci* | (Fabricius, 1775) | Coeur d'Acier | 2007 |
| ACOE1951 | Aphididae | Aphidinae | *Dysaphis* | *foeniculus* | (Theobald, 1923) | Coeur d'Acier | 2006 |
| ACOE1952 | Aphididae | Aphidinae | *Uroleucon* | *inulae* | (Ferrari, 1872) | Coeur d'Acier | 2006 |
| ACOE1953 | Aphididae | Aphidinae | *Hyperomyzus* | *lactucae* | (Linnaeus, 1758) | Coeur d'Acier | 2007 |
| ACOE1954 | Aphididae | Aphidinae | *Brevicoryne* | *brassicae* | (Linnaeus, 1758) | Coeur d'Acier | 2006 |
| ACOE1955 | Aphididae | Calaphidinae | *Therioaphis* | *riehmi* | (Börner, 1949) | Coeur d'Acier | 2006 |
| ACOE1956 | Aphididae | Aphidinae | *Myzus* | *persicae* | (Sulzer, 1776) | Coeur d'Acier | 2006 |
| ACOE1957 | Aphididae | Calaphidinae | *Hoplocallis* | *picta* | (Ferrari, 1872) | Coeur d'Acier | 2006 |
| ACOE1959 | Aphididae | Aphidinae | *Aphis* | *cytisorum* | Hartig, 1841 | Coeur d'Acier | 2007 |
| ACOE1960 | Aphididae | Greenideinae | *Greenidea* | *ficicola* | Takahashi, 1921 | Coeur d'Acier | 2006 |
| ACOE1961 | Aphididae | Aphidinae | *Aphis* | *fabae* | Scopoli, 1763 | Coeur d'Acier | 2007 |
| ACOE1962 | Aphididae | Aphidinae | *Uroleucon* | *sonchi* | (Linnaeus, 1767) | Coeur d'Acier | 2006 |
| ACOE1963 | Aphididae | Aphidinae | *Lipaphis* | *erysimi* | (Kaltenbach, 1843) | Coeur d'Acier | 2006 |
| ACOE1965 | Aphididae | Aphidinae | *Uroleucon* | *sonchi* | (Linnaeus, 1767) | Coeur d'Acier | 2006 |
| ACOE1966 | Aphididae | Calaphidinae | *Eucallipterus* | *tiliae* | (Linnaeus, 1758) | Coeur d'Acier | 2006 |
| ACOE1967 | Aphididae | Calaphidinae | *Myzocallis* | *boerneri* | Stroyan, 1957 | Coeur d'Acier | 2007 |
| ACOE1968 | Aphididae | Eriosomatinae | *Patchiella* | *reaumuri* | (Kaltenbach, 1843) | Coeur d'Acier | 2006 |
| ACOE1969 | Aphididae | Lachninae | *Cinara* | *tujafilina* | (del Guercio, 1909) | Coeur d'Acier | 2006 |
| ACOE1971 | Aphididae | Lachninae | *Cinara* | *cedri* | Mimeur, 1929 | Coeur d'Acier | 2006 |
| ACOE1973 | Aphididae | Lachninae | *Cinara* | *cedri* | Mimeur, 1929 | Coeur d'Acier | 2006 |
| ACOE1974 | Aphididae | Lachninae | *Cinara* | *cedri* | Mimeur, 1929 | Coeur d'Acier | 2006 |
| ACOE1975 | Aphididae | Aphidinae | *Myzus* | *varians* | Davidson, 1912 | Coeur d'Acier | 2006 |
| ACOE1976 | Aphididae | Aphidinae | *Brachycaudus* | *populi* | (del Guercio, 1911) | Coeur d'Acier | 2006 |
| ACOE1977 | Aphididae | Aphidinae | *Megoura* | *viciae* | Buckton, 1876 | Coeur d'Acier | 2007 |
| ACOE1978 | Aphididae | Aphidinae | *Macrosiphum* | *stellariae* | Theobald, 1913 | Coeur d'Acier | 2007 |
| ACOE1979 | Aphididae | Aphidinae | *Brachycolus* | *cucubali* | (Passerini, 1863) | Coeur d'Acier | 2006 |
| ACOE1980 | Aphididae | Aphidinae | *Brachycaudus* | *tragopogonis* | (Kaltenbach, 1843) | Coeur d'Acier | 2006 |
| ACOE1981 | Aphididae | Aphidinae | *Aphis* | *craccae* | Linnaeus, 1758 | Coeur d'Acier | 2006 |
| ACOE1982 | Aphididae | Aphidinae | *Brachycaudus* | *rumexicolens* | (Patch, 1917) | Coeur d'Acier | 2006 |
| ACOE1983 | Aphididae | Aphidinae | *Brevicoryne* | *brassicae* | (Linnaeus, 1758) | Coeur d'Acier | 2006 |
| ACOE1984 | Aphididae | Aphidinae | *Brachycaudus* | *amygdalinus* | (Schouteden, 1905) | Coeur d'Acier | 2006 |
| ACOE1985 | Aphididae | Aphidinae | *Brachycaudus* | *cardui* | (Linnaeus, 1860) | Coeur d'Acier | 2006 |
| ACOE1986 | Aphididae | Aphidinae | *Brachycaudus* | *tragopogonis* | (Kaltenbach, 1843) | Coeur d'Acier | 2006 |
| ACOE1987 | Aphididae | Aphidinae | *Brachycaudus* | *helichrysi* | (Kaltenbach, 1843) | Coeur d'Acier | 2006 |
| ACOE1988 | Aphididae | Calaphidinae | *Myzocallis* | *castanicola* | Quednau & Remaudière, 1994 | Coeur d'Acier | 2006 |
| ACOE1989 | Aphididae | Aphidinae | *Brachycaudus* | *persicae* | (Passerini, 1860) | Coeur d'Acier | 2006 |
| ACOE1990 | Aphididae | Aphidinae | *Brachycaudus* | *prunicola* | (Kaltenbach, 1843) | Coeur d'Acier | 2006 |
| ACOE1991 | Aphididae | Thelaxinae | *Thelaxes* | *suberi* | (del Guercio, 1911) | Coeur d'Acier | 2006 |
| ACOE1992 | Aphididae | Aphidinae | *Aphis* | *spiraecola* | Patch, 1914 | Coeur d'Acier | 2006 |
| ACOE1993 | Aphididae | Aphidinae | *Aphis* | *cytisorum* | Hartig, 1841 | Coeur d'Acier | 2006 |
| ACOE1994 | Aphididae | Aphidinae | *Brachycaudus* | *cardui* | (Linnaeus, 1860) | Coeur d'Acier | 2006 |
| ACOE1995 | Aphididae | Aphidinae | *Acyrthosiphon* | *malvae* | (Mosley, 1841) | Coeur d'Acier | 2007 |
| ACOE1996 | Aphididae | Aphidinae | *Aphis* | *craccivora* | Koch, 1854 | Coeur d'Acier | 2006 |
| ACOE1997 | Aphididae | Aphidinae | *Uroleucon* | *aeneum* | (Hille Ris Lambers, 1939) | Coeur d'Acier | 2006 |
| ACOE1998 | Aphididae | Aphidinae | *Anuraphis* | *pyrilaseri* | Shaposhnikov, 1950 | Coeur d'Acier | 2006 |
| ACOE1999 | Aphididae | Calaphidinae | *Panaphis* | *juglandis* | (Goeze, 1778) | Coeur d'Acier | 2006 |
| ACOE2000 | Aphididae | Aphidinae | *Anuraphis* | *pyrilaseri* | Shaposhnikov, 1950 | Coeur d'Acier | 2006 |
| ACOE2001 | Aphididae | Calaphidinae | *Euceraphis* | *betulae* | (Koch, 1855) | Coeur d'Acier | 2006 |
| ACOE2002 | Aphididae | Calaphidinae | *Euceraphis* | *betulae* | (Koch, 1855) | Coeur d'Acier | 2006 |
| ACOE2003 | Aphididae | Drepanosiphinae | *Drepanosiphum* | *oregonense* | Granovsky, 1939 | Coeur d'Acier | 2006 |
| ACOE2004 | Aphididae | Aphidinae | *Brachycaudus* | *populi* | (del Guercio, 1911) | Coeur d'Acier | 2006 |
| ACOE2005 | Aphididae | Aphidinae | *Aphis* | *spiraecola* | Patch, 1914 | Coeur d'Acier | 2007 |
| ACOE2006 | Aphididae | Aphidinae | *Macrosiphum* | *stellariae* | Theobald, 1913 | Coeur d'Acier | 2006 |
| ACOE2007 | Aphididae | Aphidinae | *Eucarazzia* | *elegans* | (Ferrari, 1872) | Coeur d'Acier | 2006 |
| ACOE2008 | Aphididae | Aphidinae | *Uroleucon* | *hypochoeridis* | (Fabricius, 1779) | Coeur d'Acier | 2006 |
| ACOE2011 | Aphididae | Aphidinae | *Macrosiphum* | *funestum* | (Macchiati, 1885) | Coeur d'Acier | 2006 |
| ACOE2012 | Aphididae | Aphidinae | *Aphis* | *galiiscabri* | Schrank, 1801 | Coeur d'Acier | 2006 |
| ACOE2013 | Aphididae | Aphidinae | *Aphis* | *fabae* | Scopoli, 1763 | Coeur d'Acier | 2006 |
| ACOE2014 | Aphididae | Calaphidinae | *Myzocallis* | *coryli* | (Goeze, 1778) | Coeur d'Acier | 2006 |
| ACOE2015 | Aphididae | Aphidinae | *Corylobium* | *avellanae* | (Schrank, 1801) | Coeur d'Acier | 2006 |
| ACOE2017 | Aphididae | Lachninae | *Cinara* | *pinimaritimae* | (Dufour, 1933) | Coeur d'Acier | 2006 |
| ACOE2018 | Aphididae | Aphidinae | *Aphis* | *lambersi* | (Börner, 1940) | Coeur d'Acier | 2006 |
| ACOE2020 | Aphididae | Aphidinae | *Macrosiphum* | *cerinthiacum* | Börner, 1950 | Coeur d'Acier | 2006 |
| ACOE2022 | Aphididae | Aphidinae | *Macrosiphoniella* | *sanborni* | (Gillette, 1908) | Coeur d'Acier | 2006 |
| ACOE2023 | Aphididae | Aphidinae | *Melanaphis* | *donacis* | (Passerini, 1862) | Coeur d'Acier | 2006 |
| ACOE2024 | Aphididae | Aphidinae | *Anuraphis* | *pyrilaseri* | Shaposhnikov, 1950 | Coeur d'Acier | 2006 |
| ACOE2025 | Aphididae | Aphidinae | *Anuraphis* | *pyrilaseri* | Shaposhnikov, 1950 | Coeur d'Acier | 2006 |
| ACOE2026 | Aphididae | Aphidinae | *Aphis* | *verbasci* | Schrank, 1801 | Coeur d'Acier | 2006 |
| ACOE2028 | Aphididae | Calaphidinae | *Appendiseta* | *robiniae* | (Gillette, 1907) | Coeur d'Acier | 2006 |
| ACOE2029 | Aphididae | Aphidinae | *Dysaphis* | *tulipae* | (Boyer de Fonscolombe, 1841) | Coeur d'Acier | 2006 |
| ACOE2030 | Aphididae | Aphidinae | *Aphis* | *gossypii* | Glover, 1877 | Coeur d'Acier | 2006 |
| ACOE2031 | Aphididae | Aphidinae | *Cavariella* | *aegopodii* | (Scopoli, 1763) | Coeur d'Acier | 2006 |
| ACOE2032 | Aphididae | Lachninae | *Lachnus* | *roboris* | (Linnaeus, 1758) | Coeur d'Acier | 2006 |
| ACOE2034 | Aphididae | Calaphidinae | *Tuberculatus* | *eggleri* | Börner, 1950 | Coeur d'Acier | 2006 |
| ACOE2035 | Aphididae | Chaitophorinae | *Chaitophorus* | *leucomelas* | Koch, 1854 | Coeur d'Acier | 2006 |
| ACOE2036 | Aphididae | Aphidinae | *Aphis* | *nerii* | Boyer de Fonscolombe, 1841 | Coeur d'Acier | 2006 |
| ACOE2041 | Aphididae | Aphidinae | *Aphis* | *craccivora* | Koch, 1854 | Coeur d'Acier | 2007 |
| ACOE2042 | Aphididae | Aphidinae | *Aphis* | *spiraecola* | Patch, 1914 | Coeur d'Acier | 2006 |
| ACOE2043 | Aphididae | Calaphidinae | *Takecallis* | *taiwana* | (Takahashi, 1926) | Coeur d'Acier | 2006 |
| ACOE2044 | Aphididae | Aphidinae | *Anthemidaphis* | *ligusticae* | (Barbagallo & Stroyan, 1980) | Coeur d'Acier | 2006 |
| ACOE2045 | Aphididae | Aphidinae | *Aphis* | *fabae* | Scopoli, 1763 | Coeur d'Acier | 2007 |
| ACOE2046 | Aphididae | Aphidinae | *Macrosiphoniella* | *tanacetaria* | (Kaltenbach, 1843) | Coeur d'Acier | 2006 |
| ACOE2047 | Aphididae | Aphidinae | *Brachycaudus* | *linariae* | Stroyan, 1950 | Coeur d'Acier | 2006 |
| ACOE2048 | Aphididae | Aphidinae | *Brachycaudus* | *helichrysi* | (Kaltenbach, 1843) | Coeur d'Acier | 2006 |
| ACOE2049 | Aphididae | Aphidinae | *Macrosiphum* | *funestum* | (Macchiati, 1885) | Coeur d'Acier | 2006 |
| ACOE2050 | Aphididae | Aphidinae | *Anuraphis* | *pyrilaseri* | Shaposhnikov, 1950 | Coeur d'Acier | 2006 |
| ACOE2051 | Aphididae | Aphidinae | *Uroleucon* | *hypochoeridis* | (Fabricius, 1779) | Coeur d'Acier | 2006 |
| ACOE2052 | Aphididae | Aphidinae | *Anuraphis* | *shaposhnikovi* | Barbagallo & Cocuzza, 2003 | Coeur d'Acier | 2006 |
| ACOE2053 | Aphididae | Aphidinae | *Anuraphis* | *subterranea* | (Walker, 1852) | Coeur d'Acier | 2006 |
| ACOE2054 | Aphididae | Aphidinae | *Melanaphis* | *pyraria* | (Passerini, 1861) | Coeur d'Acier | 2006 |
| ACOE2055 | Aphididae | Aphidinae | *Melanaphis* | *pyraria* | (Passerini, 1861) | Coeur d'Acier | 2006 |
| ACOE2056 | Aphididae | Aphidinae | *Macrosiphoniella* | *millefolii* | (de Geer, 1773) | Coeur d'Acier | 2007 |
| ACOE2057 | Aphididae | Eriosomatinae | *Prociphilus* | *bumeliae* | (Schrank, 1801) | Coeur d'Acier | 2006 |
| ACOE2058 | Aphididae | Chaitophorinae | *Periphyllus* | *testudinaceus* | (Fernie, 1852) | Coeur d'Acier | 2007 |
| ACOE2059 | Aphididae | Aphidinae | *Megoura* | *viciae* | Buckton, 1876 | Coeur d'Acier | 2007 |
| ACOE2060 | Aphididae | Aphidinae | *Anuraphis* | *subterranea* | (Walker, 1852) | Coeur d'Acier | 2006 |
| ACOE2061 | Aphididae | Calaphidinae | *Crypturaphis* | *grassii* | Silvestri, 1935 | Coeur d'Acier | 2006 |
| ACOE2062 | Aphididae | Aphidinae | *Aphis* | *punicae* | Shinji, 1863 | Coeur d'Acier | 2006 |
| ACOE2063 | Aphididae | Aphidinae | *Brachycaudus* | *klugkisti* | (Börner, 1942) | Coeur d'Acier | 2006 |
| ACOE2064 | Aphididae | Aphidinae | *Brachycaudus* | *klugkisti* | (Börner, 1942) | Coeur d'Acier | 2006 |
| ACOE2065 | Aphididae | Calaphidinae | *Appendiseta* | *robiniae* | (Gillette, 1907) | Coeur d'Acier | 2006 |
| ACOE2066 | Aphididae | Calaphidinae | *Tuberculatus* | *eggleri* | Börner, 1950 | Coeur d'Acier | 2006 |
| ACOE2067 | Aphididae | Aphidinae | *Aphis* | *farinosa* | Gmelin, 1790 | Coeur d'Acier | 2006 |
| ACOE2069 | Aphididae | Aphidinae | *Brachycaudus* | *linariae* | Stroyan, 1950 | Coeur d'Acier | 2007 |
| ACOE2070 | Aphididae | Aphidinae | *Delphiniobium* | *sp.* | Börner, 1950 | Coeur d'Acier | 2007 |
| ACOE2071 | Aphididae | Aphidinae | *Brachycaudus* | *klugkisti* | (Börner, 1942) | Coeur d'Acier | 2007 |
| ACOE2073 | Aphididae | Aphidinae | *Macrosiphum* | *stellariae* | Theobald, 1913 | Coeur d'Acier | 2006 |
| ACOE2074 | Aphididae | Aphidinae | *Myzus* | *persicae* | (Sulzer, 1776) | Coeur d'Acier | 2006 |
| ACOE2129 | Aphididae | Calaphidinae | *Betulaphis* | *quadrituberculata* | (Kaltenbach, 1843) | Coeur d'Acier | 2006 |
| ACOE2130 | Aphididae | Aphidinae | *Cavariella* | *theobaldi* | (Gillette & Bragg, 1918) | Coeur d'Acier | 2006 |
| ACOE2131 | Aphididae | Aphidinae | *Megoura* | *viciae* | Buckton, 1876 | Coeur d'Acier | 2006 |
| ACOE2132 | Aphididae | Aphidinae | *Macrosiphum* | *rosae* | (Linnaeus, 1758) | Coeur d'Acier | 2006 |
| ACOE2133 | Aphididae | Aphidinae | *Hyadaphis* | *passerini* | (del Guercio, 1911) | Coeur d'Acier | 2006 |
| ACOE2134 | Aphididae | Aphidinae | *Cavariella* | *pastinacae* | (Linnaeus, 1758) | Coeur d'Acier | 2006 |
| ACOE2135 | Aphididae | Aphidinae | *Hyperomyzus* | *lactucae* | (Linnaeus, 1758) | Coeur d'Acier | 2006 |
| ACOE2136 | Aphididae | Aphidinae | *Cavariella* | *theobaldi* | (Gillette & Bragg, 1918) | Coeur d'Acier | 2006 |
| ACOE2137 | Aphididae | Aphidinae | *Hyalopterus* | *pruni* | (Geoffroy, 1762) | Coeur d'Acier | 2006 |
| ACOE2140 | Aphididae | Aphidinae | *Macrosiphum* | *rosae* | (Linnaeus, 1758) | Coeur d'Acier | 2006 |
| ACOE2141 | Aphididae | Aphidinae | *Macrosiphum* | *euphorbiae* | (Thomas, 1878) | Coeur d'Acier | 2006 |
| ACOE2142 | Aphididae | Aphidinae | *Brachycaudus* | *helichrysi* | (Kaltenbach, 1843) | Coeur d'Acier | 2006 |
| ACOE2143 | Aphididae | Aphidinae | *Brachycaudus* | *spiraeae* | Börner, 1932 | Coeur d'Acier | 2006 |
| ACOE2144 | Aphididae | Aphidinae | *Uroleucon* | *jaceae* | (Linnaeus, 1758) | Coeur d'Acier | 2006 |
| ACOE2145 | Aphididae | Aphidinae | *Aphis* | *fabae* | Scopoli, 1763 | Coeur d'Acier | 2007 |
| ACOE2146 | Aphididae | Aphidinae | *Amphorophora* | *rubi* | (Kaltenbach, 1843) | Coeur d'Acier | 2006 |
| ACOE2147 | Aphididae | Calaphidinae | *Euceraphis* | *punctipennis* | (Koch, 1855) | Coeur d'Acier | 2006 |
| ACOE2148 | Aphididae | Calaphidinae | *Tuberculatus* | *annulatus* | (Hartig, 1841) | Coeur d'Acier | 2006 |
| ACOE2149 | Aphididae | Aphidinae | *Aphis* | *taraxacicola* | (Börner, 1940) | Coeur d'Acier | 2006 |
| ACOE2150 | Aphididae | Aphidinae | *Aphis* | *craccae* | Linnaeus, 1758 | Coeur d'Acier | 2006 |
| ACOE2151 | Aphididae | Aphidinae | *Aphis* | *salicariae* | Koch, 1855 | Coeur d'Acier | 2007 |
| ACOE2153 | Aphididae | Aphidinae | *Aphis* | *fabae* | Scopoli, 1763 | Coeur d'Acier | 2006 |
| ACOE2154 | Aphididae | Aphidinae | *Brachycaudus* | *sedi* | (Jacob, 1964) | Coeur d'Acier | 2006 |
| ACOE2155 | Aphididae | Calaphidinae | *Tuberculatus* | *annulatus* | (Hartig, 1841) | Coeur d'Acier | 2006 |
| ACOE2275 | Aphididae | Aphidinae | *Aphis* | *cytisorum* | Hartig, 1841 | Coeur d'Acier | 2007 |
| ACOE2277 | Aphididae | Eriosomatinae | *Pemphigus* | *vesicarius* | Passerini, 1861 | Coeur d'Acier | 2007 |
| ACOE2278 | Aphididae | Chaitophorinae | *Chaitophorus* | *leucomelas* | Koch, 1854 | Coeur d'Acier | 2007 |
| ACOE2279 | Aphididae | Aphidinae | *Aphis* | *intybi* | Koch, 1855 | Coeur d'Acier | 2007 |
| ACOE2280 | Aphididae | Aphidinae | *Aphis* | *confusa* | Walker, 1849 | Coeur d'Acier | 2007 |
| ACOE2281 | Aphididae | Aphidinae | *Brachycaudus* | *tragopogonis* | (Kaltenbach, 1843) | Coeur d'Acier | 2007 |
| ACOE2284 | Aphididae | Aphidinae | *Dysaphis* | *apiifolia* | (Theobald, 1923) | Coeur d'Acier | 2007 |
| ACOE2285 | Aphididae | Aphidinae | *Macrosiphum* | *rosae* | (Linnaeus, 1758) | Coeur d'Acier | 2007 |
| ACOE2286 | Aphididae | Aphidinae | *Aphis* | *nerii* | Boyer de Fonscolombe, 1841 | Coeur d'Acier | 2007 |
| ACOE2287 | Aphididae | Aphidinae | *Aphis* | *ruborum* | (Börner, 1932) | Coeur d'Acier | 2007 |
| ACOE2292 | Aphididae | Eriosomatinae | *Eriosoma* | *lanuginosum* | (Hartig, 1839) | Coeur d'Acier | 2007 |
| ACOE2296 | Aphididae | Aphidinae | *Brachycaudus* | *cardui* | (Linnaeus, 1860) | Coeur d'Acier | 2007 |
| ACOE2299 | Aphididae | Aphidinae | *Metopeurum* | *fuscoviride* | Stroyan, 1950 | Coeur d'Acier | 2007 |
| ACOE2360 | Aphididae | Aphidinae | *Aphis* | *spiraecola* | Patch, 1914 | Coeur d'Acier | 2007 |
| ACOE2406 | Aphididae | Aphidinae | *Brachycaudus* | *helichrysi* | (Kaltenbach, 1843) | Coeur d'Acier | 2008 |
| ACOE2407 | Aphididae | Aphidinae | *Brachycaudus* | *helichrysi* | (Kaltenbach, 1843) | Coeur d'Acier | 2008 |
| ACOE2408 | Aphididae | Aphidinae | *Brachycaudus* | *helichrysi* | (Kaltenbach, 1843) | Coeur d'Acier | 2008 |
| ACOE2411 | Aphididae | Aphidinae | *Brachycaudus* | *helichrysi* | (Kaltenbach, 1843) | Coeur d'Acier | 2008 |
| ACOE2412 | Aphididae | Aphidinae | *Brachycaudus* | *helichrysi* | (Kaltenbach, 1843) | Coeur d'Acier | 2008 |
| ACOE2413 | Aphididae | Aphidinae | *Brachycaudus* | *lychnidis* | (Linnaeus, 1758) | Coeur d'Acier | 2009 |
| ACOE2414 | Aphididae | Aphidinae | *Uroleucon* | *aeneum* | (Hille Ris Lambers, 1939) | Coeur d'Acier | 2009 |
| ACOE2415 | Aphididae | Aphidinae | *Metopolophium* | *dirhodum* | (Walker, 1849) | Coeur d'Acier | 2009 |
| ACOE2416 | Aphididae | Chaitophorinae | *Sipha* | *maydis* | Passerini, 1860 | Coeur d'Acier | 2009 |
| ACOE2418 | Aphididae | Aphidinae | *Aphis* | *confusa* | Walker, 1849 | Coeur d'Acier | 2009 |
| ACOE2419 | Aphididae | Aphidinae | *Hyperomyzus* | *lactucae* | (Linnaeus, 1758) | Coeur d'Acier | 2009 |
| ACOE2421 | Aphididae | Eriosomatinae | *Eriosoma* | *lanigerum* | (Hausmann, 1802) | Coeur d'Acier | 2009 |
| ACOE2422 | Aphididae | Aphidinae | *Brachycaudus* | *helichrysi* | (Kaltenbach, 1843) | Coeur d'Acier | 2008 |
| ACOE2424 | Aphididae | Aphidinae | *Brachycaudus* | *helichrysi* | (Kaltenbach, 1843) | Coeur d'Acier | 2008 |
| ACOE2425 | Aphididae | Aphidinae | *Brachycaudus* | *helichrysi* | (Kaltenbach, 1843) | Coeur d'Acier | 2009 |
| ACOE2426 | Aphididae | Aphidinae | *Brachycaudus* | *amygdalinus* | (Schouteden, 1905) | Coeur d'Acier | 2009 |
| ACOE2429 | Aphididae | Aphidinae | *Rhopalosiphum* | *padi* | (Linnaeus, 1758) | Coeur d'Acier | 2009 |
| ACOE2430 | Aphididae | Aphidinae | *Myzus* | *persicae* | (Sulzer, 1776) | Coeur d'Acier | 2009 |
| ACOE2431 | Aphididae | Calaphidinae | *Takecallis* | *arundinariae* | (Essig, 1917) | Coeur d'Acier | 2008 |
| ACOE2432 | Aphididae | Aphidinae | *Melanaphis* | *bambusae* | (Fullaway, 1910) | Coeur d'Acier | 2008 |
| ACOE2433 | Aphididae | Aphidinae | *Brachycaudus* | *helichrysi* | (Kaltenbach, 1843) | Coeur d'Acier | 2008 |
| ACOE2435 | Aphididae | Aphidinae | *Wahlgreniella* | *arbuti* | (Davidson, 1910) | Coeur d'Acier | 2009 |
| ACOE2437 | Aphididae | Chaitophorinae | *Sipha* | *maydis* | Passerini, 1860 | Coeur d'Acier | 2009 |
| ACOE2438 | Aphididae | Aphidinae | *Brachycaudus* | *helichrysi* | (Kaltenbach, 1843) | Coeur d'Acier | 2008 |
| ACOE2439 | Aphididae | Aphidinae | *Macrosiphum* | *euphorbiae* | (Thomas, 1878) | Coeur d'Acier | 2009 |
| ACOE2440 | Aphididae | Calaphidinae | *Takecallis* | *arundicolens* | (Clarke, 1903) | Coeur d'Acier | 2009 |
| ACOE2441 | Aphididae | Aphidinae | *Uroleucon* | *inulae* | (Ferrari, 1872) | Coeur d'Acier | 2009 |
| ACOE2442 | Aphididae | Aphidinae | *Megoura* | *viciae* | Buckton, 1876 | Coeur d'Acier | 2009 |
| ACOE2443 | Aphididae | Aphidinae | *Hyalopterus* | *amygdali* | (Blanchard E., 1840) | Coeur d'Acier | 2009 |
| ACOE2444 | Aphididae | Aphidinae | *Aphis* | *vallei* | Hille Ris Lambers & Stroyan, 1959 | Coeur d'Acier | 2009 |
| ACOE2445 | Aphididae | Aphidinae | *Myzus* | *persicae* | (Sulzer, 1776) | Coeur d'Acier | 2009 |
| ACOE2446 | Aphididae | Calaphidinae | *Tinocallis* | *takachioensis* | Higuchi, 1972 | Coeur d'Acier | 2009 |
| ACOE2447 | Aphididae | Aphidinae | *Aphis* | *viticis* | Ferrari, 1872 | Coeur d'Acier | 2009 |
| ACOE2448 | Aphididae | Aphidinae | *Brachycaudus* | *helichrysi* | (Kaltenbach, 1843) | Coeur d'Acier | 2008 |
| ACOE2449 | Aphididae | Aphidinae | *Macrosiphum* | *euphorbiae* | (Thomas, 1878) | Coeur d'Acier | 2009 |
| ACOE2451 | Aphididae | Aphidinae | *Brevicoryne* | *brassicae* | (Linnaeus, 1758) | Coeur d'Acier | 2009 |
| ACOE2452 | Aphididae | Chaitophorinae | *Sipha* | *maydis* | Passerini, 1860 | Coeur d'Acier | 2009 |
| ACOE2453 | Aphididae | Aphidinae | *Aphis* | *lugentis* | Williams, 1911 | Coeur d'Acier | 2009 |
| ACOE2454 | Aphididae | Aphidinae | *Aphis* | *umbrella* | (Börner, 1950) | Coeur d'Acier | 2009 |
| ACOE2455 | Aphididae | Aphidinae | *Brachycaudus* | *helichrysi* | (Kaltenbach, 1843) | Coeur d'Acier | 2008 |
| ACOE2456 | Aphididae | Aphidinae | *Hyadaphis* | *foeniculi* | (Passerini, 1860) | Coeur d'Acier | 2009 |
| ACOE2457 | Aphididae | Aphidinae | *Brachycaudus* | *amygdalinus* | (Schouteden, 1905) | Coeur d'Acier | 2009 |
| ACOE2458 | Aphididae | Aphidinae | *Brachycaudus* | *helichrysi* | (Kaltenbach, 1843) | Coeur d'Acier | 2008 |
| ACOE2459 | Aphididae | Aphidinae | *Melanaphis* | *donacis* | (Passerini, 1862) | Coeur d'Acier | 2009 |
| ACOE2460 | Aphididae | Aphidinae | *Ceruraphis* | *eriophori* | (Walker, 1848) | Coeur d'Acier | 2009 |
| ACOE2461 | Aphididae | Aphidinae | *Brachycaudus* | *helichrysi* | (Kaltenbach, 1843) | Coeur d'Acier | 2008 |
| ACOE2463 | Aphididae | Aphidinae | *Brachycaudus* | *helichrysi* | (Kaltenbach, 1843) | Coeur d'Acier | 2008 |
| ACOE2464 | Aphididae | Aphidinae | *Brachycaudus* | *helichrysi* | (Kaltenbach, 1843) | Coeur d'Acier | 2008 |
| ACOE2465 | Aphididae | Aphidinae | *Brachycaudus* | *helichrysi* | (Kaltenbach, 1843) | Coeur d'Acier | 2008 |
| ACOE2466 | Aphididae | Lachninae | *Cinara* | *confinis* | (Koch, 1856) | Coeur d'Acier | 2008 |
| ACOE2467 | Aphididae | Aphidinae | *Elatobium* | *abietinum* | (Walker, 1849) | Coeur d'Acier | 2008 |
| ACOE2468 | Aphididae | Aphidinae | *Brachycaudus* | *helichrysi* | (Kaltenbach, 1843) | Coeur d'Acier | 2008 |
| ACOE2469 | Aphididae | Aphidinae | *Brachycaudus* | *helichrysi* | (Kaltenbach, 1843) | Coeur d'Acier | 2008 |
| ACOE2470 | Aphididae | Aphidinae | *Brachycaudus* | *helichrysi* | (Kaltenbach, 1843) | Coeur d'Acier | 2008 |
| ACOE2471 | Aphididae | Aphidinae | *Brachycaudus* | *helichrysi* | (Kaltenbach, 1843) | Coeur d'Acier | 2009 |
| ACOE2472 | Aphididae | Aphidinae | *Dysaphis* | *plantaginea* | (Passerini, 1860) | Coeur d'Acier | 2009 |
| ACOE2473 | Aphididae | Aphidinae | *Myzus* | *cerasi* | (Fabricius, 1775) | Coeur d'Acier | 2009 |
| ACOE2474 | Aphididae | Aphidinae | *Brachycaudus* | *helichrysi* | (Kaltenbach, 1843) | Coeur d'Acier | 2008 |
| ACOE2476 | Aphididae | Aphidinae | *Brachycaudus* | *helichrysi* | (Kaltenbach, 1843) | Coeur d'Acier | 2008 |
| ACOE2477 | Aphididae | Aphidinae | *Brachycaudus* | *helichrysi* | (Kaltenbach, 1843) | Coeur d'Acier | 2008 |
| ACOE2478 | Aphididae | Aphidinae | *Aphis* | *caroliboerneri* | (Remaudière, 1952) | Coeur d'Acier | 2008 |
| ACOE2480 | Aphididae | Calaphidinae | *Therioaphis* | *riehmi* | (Börner, 1949) | Coeur d'Acier | 2008 |
| ACOE2481 | Aphididae | Aphidinae | *Myzus* | *lythri* | (Schrank, 1801) | Coeur d'Acier | 2008 |
| ACOE2482 | Aphididae | Aphidinae | *Aphis* | *clematidis* | Koch, 1854 | Coeur d'Acier | 2008 |
| ACOE2483 | Aphididae | Aphidinae | *Aphis* | *oenotherae* | Oestlund, 1887 | Coeur d'Acier | 2008 |
| ACOE2486 | Aphididae | Aphidinae | *Brachycaudus* | *helichrysi* | (Kaltenbach, 1843) | Coeur d'Acier | 2008 |
| ACOE2487 | Aphididae | Aphidinae | *Brachycaudus* | *helichrysi* | (Kaltenbach, 1843) | Coeur d'Acier | 2008 |
| ACOE2602 | Aphididae | Aphidinae | *Aphis* | *cisticola* | Leclant & Remaudière, 1972 | Coeur d'Acier | 2009 |
| ACOE2603 | Aphididae | Aphidinae | *Hyperomyzus* | *rhinanthi* | (Schouteden, 1903) | Coeur d'Acier | 2009 |
| ACOE2604 | Aphididae | Aphidinae | *Brachycaudus* | *populi* | (del Guercio, 1911) | Coeur d'Acier | 2009 |
| ACOE2611 | Aphididae | Pterocommatinae | *Pterocomma* | *rufipes* | (Hartig, 1841) | Coeur d'Acier | 2009 |
| ACOE2612 | Aphididae | Lachninae | *Cinara* | *cuneomaculata* | (del Guercio, 1909) | Coeur d'Acier | 2009 |
| ACOE2614 | Aphididae | Aphidinae | *Aphis* | *farinosa* | Gmelin, 1790 | Coeur d'Acier | 2009 |
| ACOE2615 | Aphididae | Pterocommatinae | *Pterocomma* | *pilosum* | Buckton, 1879 | Coeur d'Acier | 2009 |
| ACOE2638 | Aphididae | Aphidinae | *Aphis* | *spiraecola* | Patch, 1914 | Coeur d'Acier | 2009 |
| ACOE2639 | Aphididae | Aphidinae | *Aphis* | *spiraecola* | Patch, 1914 | Coeur d'Acier | 2009 |
| ACOE2640 | Aphididae | Aphidinae | *Aphis* | *pomi* | de Geer, 1773 | Coeur d'Acier | 2009 |
| ACOE2641 | Aphididae | Aphidinae | *Aphis* | *pomi* | de Geer, 1773 | Coeur d'Acier | 2009 |
| ACOE2642 | Aphididae | Aphidinae | *Aphis* | *pomi* | de Geer, 1773 | Coeur d'Acier | 2009 |
| ACOE2643 | Aphididae | Aphidinae | *Aphis* | *spiraecola* | Patch, 1914 | Coeur d'Acier | 2009 |
