# Supplementary material for: DNA Barcoding and the Associated PhylAphidB@se Website for the Identification of European Aphids (Insecta: Hemiptera: Aphididae)
Source: PLoS One. 2014 Jun 4;9(6):e97620. doi: 10.1371/journal.pone.0097620 (PMC4045754; doi:10.1371/journal.pone.0097620)
Supplement: Table S3 — List of species included in the study. List of species included in the study, with a summary of the results obtained in barcoding analysis. (DOCX) [file pone.0097620.s004.docx]

| Species Name | NS. | NH. | Min-WSD | Mean-WSD | Max-WSD | Min-BSD | Type Clade | BP value |
| --- | --- | --- | --- | --- | --- | --- | --- | --- |
| *Acyrthosiphon caraganae* (Cholodkovsky, 1907) | 1 | 1 | - | - | - | 3,92% | - | - |
| *Acyrthosiphon lambersi* Leclant & Remaudière, 1974 | 1 | 1 | - | - | - | 3,59% | - | - |
| *Acyrthosiphon malvae* (Mosley, 1841) | 3 | 2 | 0,00% | 0,10% | 0,15% | 3,42% | M | 100 |
| *Acyrthosiphon pisum* (Harris, 1776) | 2 | 1 | 0,00% | 0,00% | 0,00% | 4,72% | M | 100 |
| *Amphorophora rubi* (Kaltenbach, 1843) | 6 | 3 | 0,00% | 0,32% | 0,46% | 4,07% | M | 100 |
| *Anoecia corni* (Fabricius, 1775) | 1 | 1 | - | - | - | 10,97% | - | - |
| *Anthemidaphis ligusticae* (Barbagallo & Stroyan, 1980) | 1 | 1 | - | - | - | 7,04% | - | - |
| *Anuraphis cachryos* Barbagallo & Stroyan, 1980 | 1 | 1 | - | - | - | 5,07% | - | - |
| *Anuraphis pyrilaseri* Shaposhnikov, 1950 | 5 | 3 | 0,00% | 0,24% | 0,30% | 5,06% | M | 100 |
| *Anuraphis shaposhnikovi* Barbagallo & Cocuzza, 2003 | 2 | 1 | 0,00% | 0,00% | 0,00% | 4,08% | M | 100 |
| *Anuraphis subterranea* (Walker, 1852) | 4 | 2 | 0,00% | 0,08% | 0,15% | 4,08% | M | 100 |
| *Aphis acetosae* Linnaeus, 1761 | 2 | 1 | 0,00% | 0,00% | 0,00% | 3,59% | M | 100 |
| *Aphis aliena* Theobald, 1915 | 1 | 1 | - | - | - | 1,23% | - | - |
| *Aphis althaeae (Nevsky, 1929* | 1 | 1 | - | - | - | 0,30% | - | - |
| *Aphis brotericola* Linnaeus, 1758 | 1 | 1 | - | - | - | 2,32% | - | - |
| *Aphis cacaliasteris* Hille Ris Lambers, 1947 | 1 | 1 | - | - | - | 0,76% | - | - |
| *Aphis caroliboerneri* (Remaudière, 1952) | 1 | 1 | - | - | - | 1,38% | - | - |
| *Aphis chloris* Koch, 1854 | 1 | 1 | - | - | - | 0,15% | - | - |
| *Aphis cisticola* Leclant & Remaudière, 1972 | 2 | 1 | 0,00% | 0,00% | 0,00% | 0,61% | M | 99 |
| *Aphis clematidis* Linnaeus, 1758 | 2 | 1 | 0,00% | 0,00% | 0,00% | 4,56% | M | 100 |
| *Aphis confusa* Walker, 1849 | 5 | 4 | 0,00% | 0,24% | 0,46% | 0,00% | P | 45 |
| *Aphis coronillae* Ferrari, 1872 | 5 | 1 | 0,00% | 0,00% | 0,00% | 0,15% | M | 64 |
| *Aphis craccae* Linnaeus, 1758 | 4 | 3 | 0,00% | 0,31% | 0,61% | 1,85% | M | 96 |
| *Aphis craccivora* Koch, 1854 | 23 | 7 | 0,00% | 0,22% | 0,46% | 0,15% | P | 97 |
| *Aphis crepidis* (Börner, 1940) | 2 | 1 | 0,00% | 0,00% | 0,00% | 0,00% | P | 44 |
| *Aphis cytisorum* Hartig, 1841 | 8 | 6 | 0,00% | 0,43% | 0,92% | 0,15% | P | 100 |
| *Aphis epilobii* Kaltenbach, 1843 | 7 | 2 | 0,00% | 0,07% | 0,15% | 1,07% | M | 100 |
| *Aphis fabae* Scopoli, 1763 | 96 | 13 | 0,00% | 0,11% | 0,76% | 0,00% | P | 77 |
| *Aphis farinosa* Gmelin, 1790 | 4 | 3 | 0,00% | 0,18% | 0,30% | 7,07% | M | 100 |
| *Aphis galiiscabri* Schrank, 1801 | 2 | 2 | 0,15% | 0,15% | 0,15% | 0,00% | P | 100 |
| *Aphis gossypii* Glover, 1877 | 15 | 3 | 0,00% | 0,08% | 0,61% | 0,00% | P | 90 |
| *Aphis grossulariae* Kaltenbach, 1843 | 10 | 2 | 0,00% | 0,03% | 0,15% | 1,07% | M | 99 |
| *Aphis hederae* Kaltenbach, 1843 | 7 | 4 | 0,00% | 0,22% | 0,46% | 0,00% | P | 9 |
| *Aphis hypochoeridis* (Börner, 1940) | 1 | 1 | - | - | - | 1.38% | - | - |
| *Aphis idaei* van der Goot, 1912 | 1 | 1 | - | - | - | 4,56% | - | - |
| *Aphis ilicis* Kaltenbach, 1843 | 2 | 1 | 0,00% | 0,00% | 0,00% | 1,23% | M | 100 |
| *Aphis intybi* Koch, 1855 | 4 | 3 | 0,00% | 0,15% | 0,30% | 0,61% | M | 98 |
| *Aphis jacobaeae* Schrank, 1801 | 1 | 1 | - | - | - | 1,07% | - | - |
| *Aphis lambersi* (Börner, 1940) | 4 | 4 | 0,15% | 0,53% | 0,92% | 0,15% | P | 77 |
| *Aphis leontodontis* (Börner, 1940) | 1 | 1 | - | - | - | 0,00% | - | - |
| *Aphis lichtensteini* Leclant & Remaudière, 1972 | 1 | 1 | - | - | - | 0,61% | - | - |
| *Aphis longirostris*(Börner, 1950) | 1 | 1 | - | - | - | 0,00% | - | - |
| *Aphis lugentis* Williams, 1911 | 3 | 1 | 0,00% | 0,00% | 0,00% | 2,17% | M | 100 |
| *Aphis mamonthovae* Davletschina, 1964 | 2 | 2 | 0,15% | 0,15% | 0,15% | 0,00% | P | 60 |
| *Aphis nasturtii* Kaltenbach, 1843 | 2 | 2 | 0,46% | 0,46% | 0,46% | 0,15% | P | 100 |
| *Aphis nerii* Boyer de Fonscolombe, 1841 | 5 | 1 | 0,00% | 0,00% | 0,00% | 6,21% | M | 100 |
| *Aphis newtoni* Theobald, 1927 | 5 | 2 | 0,00% | 0,18% | 0,46% | 0,15% | P | 9 |
| *Aphis oenotherae* Oestlund, 1887 | 8 | 1 | 0,00% | 0,00% | 0,00% | 2,81% | M | 100 |
| *Aphis origani* Linnaeus, 1758 | 2 | 2 | 0,15% | 0,15% | 0,15% | 0,15% | M | 89 |
| *Aphis parietariae* Theobald, 1923 | 1 | 1 | - | - | - | 0,00% | - | - |
| *Aphis plantaginis* Goeze, 1778 | 3 | 1 | 0,00% | 0,00% | 0,00% | 0,00% | P | 44 |
| *Aphis pomi* de Geer, 1773 | 13 | 2 | 0,00% | 0,16% | 0,30% | 4,93% | M | 100 |
| *Aphis proffti* (Börner, 1942) | 1 | 1 | - | - | - | 4,27% | - | - |
| *Aphis pseudocomosa* Stroyan, 1972 | 1 | 1 | - | - | - | 1,85% | - | - |
| *Aphis punicae* Passerini, 1863 | 2 | 1 | 0,00% | 0,00% | 0,00% | 0,00% | P | 50 |
| *Aphis ruborum* (Börner, 1932) | 11 | 4 | 0,00% | 0,13% | 0,30% | 2,32% | M | 100 |
| *Aphis rumicis* Linnaeus, 1758 | 2 | 2 | 0,15% | 0,15% | 0,15% | 3,92% | M | 100 |
| *Aphis salicariae* Koch, 1855 | 3 | 2 | 0,00% | 0,10% | 0,15% | 6,04% | M | 100 |
| *Aphis salviae* Walker, 1852 | 1 | 1 | - | - | - | 2,63% | - | - |
| *Aphis sambuci* Linnaeus, 1758 | 7 | 4 | 0,00% | 0,44% | 1,07% | 6,05% | M | 100 |
| *Aphis sanguisorbae* Schrank, 1801 | 4 | 3 | 0,00% | 0,69% | 1,38% | 4,89% | M | 100 |
| *Aphis sedi* Kaltenbach, 1843 | 2 | 1 | 0,00% | 0,00% | 0,00% | 0,00% | P | 44 |
| *Aphis serpylli* Koch, 1854 | 4 | 4 | 0,30% | 0,61% | 1,07% | 0,15% | P | 98 |
| *Aphis sp.567* | 1 | 1 | - | - | - | 0,76% | - | - |
| *Aphis sp.frangulae-like* | 8 | 4 | 0,00% | 0,22% | 0,61% | 0,00% | P | 82 |
| *Aphis sp.rostellum-like* | 4 | 1 | 0,00% | 0,00% | 0,00% | 0,15% | M | 55 |
| *Aphis spiraecola* Patch, 1914 | 19 | 2 | 0,00% | 0,22% | 0,46% | 4,93% | M | 100 |
| *Aphis spiraephaga* Müller F.P., 1961 | 1 | 1 | - | - | - | 0,00% | - | - |
| *Aphis taraxacicola* (Börner, 1940) | 4 | 2 | 0,00% | 0,08% | 0,15% | 0,00% | P | 39 |
| *Aphis teucrii* (Börner, 1942) | 1 | 1 | - | - | - | 0,00% | - | - |
| *Aphis tirucallis* Hille Ris Lambers, 1954 | 1 | 1 | - | - | - | 0,15% | - | - |
| *Aphis ulicis* Walker, 1870 | 3 | 2 | 0,00% | 0,10% | 0,15% | 0,15% | M | 26 |
| *Aphis ulmariae* Schrank, 1801 | 2 | 1 | 0,00% | 0,00% | 0,00% | 4,73% | M | 100 |
| *Aphis umbrella* (Börner, 1950) | 3 | 1 | 0,00% | 0,00% | 0,00% | 0,15% | M | 66 |
| *Aphis urticata* Gmelin, 1790 | 6 | 2 | 0,00% | 0,05% | 0,15% | 4,09% | M | 100 |
| *Aphis vallei* Hille Ris Lambers & Stroyan, 1959 | 2 | 2 | 0,30% | 0,30% | 0,30% | 2,01% | M | 100 |
| *Aphis veratri* Walker, 1852 | 1 | 1 | - | - | - | 4,89% | - | - |
| *Aphis verbasci* Schrank, 1801 | 5 | 3 | 0,00% | 0,12% | 0,30% | 5,54% | M | 100 |
| *Aphis viburni* Scopoli, 1763 | 2 | 1 | 0,00% | 0,00% | 0,00% | 0,00% | P | 9 |
| *Aphis vitalbae* Ferrari, 1872 | 2 | 1 | 0,00% | 0,00% | 0,00% | 5,23% | M | 100 |
| *Aphis viticis* Ferrari, 1872 | 3 | 2 | 0,00% | 0,10% | 0,15% | 1,38% | M | 100 |
| *Aploneura lentisci* (Passerini, 1856) | 1 | 1 | - | - | - | 10,09% | - | - |
| *Appendiseta robiniae* (Gillette, 1907) | 2 | 2 | 0,92% | 0,92% | 0,92% | 12,04% | M | 100 |
| *Aulacorthum solani* (Kaltenbach, 1843) | 6 | 3 | 0,00% | 0,19% | 0,46% | 3,93% | M | 100 |
| *Baizongia pistaciae* (Linnaeus, 1767) | 1 | 1 | - | - | - | 10,42% | - | - |
| *Betulaphis brevipilosa* Börner, 1940 | 1 | 1 | - | - | - | 0,30% | - | - |
| *Betulaphis quadrituberculata* (Kaltenbach, 1843) | 1 | 1 | - | - | - | 0,30% | - | - |
| *Brachycaudus aconiti* (Mordvilko, 1928) | 1 | 1 | - | - | - | 6,05% | - | - |
| *Brachycaudus amygdalinus* (Schouteden, 1905) | 5 | 1 | 0,00% | 0,00% | 0,00% | 3,76% | M | 100 |
| *Brachycaudus bicolor* (Nevsky, 1929) | 3 | 1 | 0,00% | 0,00% | 0,00% | 3,28% | M | 100 |
| *Brachycaudus cardui* (Linnaeus, 1860) | 17 | 5 | 0,00% | 0,32% | 0,92% | 0,00% | P | 100 |
| *Brachycaudus cerinthis* Bozhko, 1961 | 1 | 1 | - | - | - | 3,60% | - | - |
| *Brachycaudus helichrysi* (Kaltenbach, 1843) | 42 | 8 | 0,00% | 1,26% | 2,81% | 1,70% | P | 88 |
| *Brachycaudus klugkisti* (Börner, 1942) | 5 | 3 | 0,00% | 0,40% | 0,92% | 3,45% | M | 100 |
| *Brachycaudus lateralis* (Walker, 1848) | 6 | 2 | 0,00% | 0,10% | 0,30% | 0,00% | P | 100 |
| *Brachycaudus linariae* Stroyan, 1950 | 4 | 3 | 0,00% | 0,56% | 0,76% | 2,96% | M | 100 |
| *Brachycaudus lychnidis* (Linnaeus, 1758) | 8 | 2 | 0,00% | 0,13% | 0,30% | 0,15% | P | 51 |
| *Brachycaudus persicae* (Passerini, 1860) | 4 | 1 | 0,00% | 0,00% | 0,00% | 2,49% | M | 100 |
| *Brachycaudus populi* (del Guercio, 1911) | 5 | 3 | 0,00% | 0,61% | 1,08% | 0,15% | P | 100 |
| *Brachycaudus prunicola* (Kaltenbach, 1843) | 2 | 1 | 0,00% | 0,00% | 0,00% | 0,00% | P | 87 |
| *Brachycaudus rumexicolens* (Patch, 1917) | 1 | 1 | - | - | - | 0,96% | - | - |
| *Brachycaudus schwartzi* (Börner, 1931) | 3 | 2 | 0,00% | 0,10% | 0,15% | 0,00% | P | 82 |
| *Brachycaudus sedi* (Jacob, 1964) | 1 | 1 | - | - | - | 0,96% | - | - |
| *Brachycaudus spiraeae* Börner, 1932 | 1 | 1 | - | - | - | 1,70% | - | - |
| *Brachycaudus tragopogonis* (Kaltenbach, 1843) | 9 | 2 | 0,00% | 0,17% | 0,30% | 0,00% | P | 100 |
| *Brachycolus cucubali* (Passerini, 1863) | 1 | 1 | - | - | - | 3,77% | - | - |
| *Brachyunguis tamaricis* (Lichtenstein, 1885) | 2 | 2 | 2,64% | 2,64% | 2,64% | 6,69% | M | 100 |
| *Brevicoryne brassicae* (Linnaeus, 1758) | 9 | 1 | 0,00% | 0,00% | 0,00% | 3,59% | M | 100 |
| *Callipterinella tuberculata* (von Heyden, 1837) | 1 | 1 | - | - | - | 10,25% | - | - |
| *Cavariella aegopodii* (Scopoli, 1763) | 2 | 1 | 0,00% | 0,00% | 0,00% | 5,44% | M | 100 |
| *Cavariella pastinacae* (Linnaeus, 1758) | 1 | 1 | - | - | - | 4,93% | - | - |
| *Cavariella theobaldi* (Gillette & Bragg, 1918) | 12 | 4 | 0,00% | 0,18% | 0,92% | 4,93% | M | 100 |
| *Ceruraphis eriophori* (Walker, 1848) | 3 | 1 | 0,00% | 0,00% | 0,00% | 5,06% | M | 100 |
| *Chaitophorus capreae* (Mosley, 1841) | 2 | 2 | 0,61% | 0,61% | 0,61% | 8,08% | M | 100 |
| *Chaitophorus leucomelas* Koch, 1854 | 12 | 6 | 0,00% | 0,72% | 1,70% | 7,07% | M | 100 |
| *Chaitophorus nigricantis* Pintera, 1987 | 1 | 1 | - | - | - | 7,11% | - | - |
| *Chaitophorus populeti* (Panzer, 1801) | 10 | 2 | 0,00% | 0,18% | 0,92% | 11,95% | M | 100 |
| *Chaitophorus populialbae* (Boyer de Fonscolombe, 1841) | 4 | 3 | 0,00% | 0,49% | 0,92% | 8,28% | M | 100 |
| *Chaitophorus salicti* (Schrank, 1801) | 2 | 2 | 0,15% | 0,15% | 0,15% | 7,43% | M | 100 |
| *Chaitophorus salijaponicus* Essig & Kuwana, 1918 | 4 | 2 | 0,00% | 0,08% | 0,15% | 7,97% | M | 100 |
| *Chaitophorus truncatus* (Hausmann, 1802) | 1 | 1 | - | - | - | 7,07% | - | - |
| *Chromaphis juglandicola* (Kaltenbach,1843) | 1 | 1 | - | - | - | 9,61% | - | - |
| *Cinara brauni* Börner, 1940 | 1 | 1 | - | - | - | 2,49% | - | - |
| *Cinara cedri* Mimeur, 1929 | 3 | 2 | 0,00% | 0,10% | 0,15% | 8,53% | M | 100 |
| *Cinara confinis* (Koch, 1856) | 1 | 1 | - | - | - | 7,91% | - | - |
| *Cinara cuneomaculata* (del Guercio, 1909) | 1 | 1 | - | - | - | 6,36% | - | - |
| *Cinara fresai* Blanchard E.E., 1939 | 1 | 1 | - | - | - | 5,42% | - | - |
| *Cinara juniperi* (de Geer, 1773) | 1 | 1 | - | - | - | 5,41% | - | - |
| *Cinara laricis* (Hartig, 1839) | 1 | 1 | - | - | - | 6,36% | - | - |
| *Cinara maghrebica* Mimeur, 1934 | 1 | 1 | - | - | - | 2,65% | - | - |
| *Cinara palaestinensis* Hille Ris Lambers, 1938 | 2 | 1 | 0,00% | 0,00% | 0,00% | 2,65% | M | 100 |
| *Cinara pectinatae* (Nördlinger, 1880) | 1 | 1 | - | - | - | 7,86% | - | - |
| *Cinara pilicornis* (Hartig, 1841) | 2 | 1 | 0,00% | 0,00% | 0,00% | 6,54% | M | 100 |
| *Cinara pinea* (Mordvilko, 1895) | 2 | 2 | 0,30% | 0,30% | 0,30% | 4,42% | M | 100 |
| *Cinara pini* (Linnaeus, 1758) | 1 | 1 | - | - | - | 3,76% | - | - |
| *Cinara pinimaritimae* (Dufour, 1933) | 1 | 1 | - | - | - | 2,49% | - | - |
| *Cinara pruinosa* (Hartig, 1841) | 1 | 1 | - | - | - | 6,54% | - | - |
| *Cinara tujafilina* (del Guercio, 1909) | 1 | 1 | - | - | - | 5,41% |  | - |
| *Corylobium avellanae* (Schrank, 1801) | 2 | 2 | 0,46% | 0,46% | 0,46% | 3,59% | M | 100 |
| *Cryptomyzus ribis* (Linnaeus, 1758) | 1 | 1 | - | - | - | 4,40% | - | - |
| *Cryptomyzus sp.1411* (Kaltenbach, 1843) | 1 | 1 | - | - | - | 3,58% | - | - |
| *Crypturaphis grassii* Silvestri, 1935 | 1 | 1 | - | - | - | 10,96% | - | - |
| *Delphiniobium sp.2070* Börner, 1950 | 1 | 1 | - | - | - | 4,08% | - | - |
| *Drepanosiphum oregonense* Granovsky, 1939 | 1 | 1 | - | - | - | 6,23% | - | - |
| *Drepanosiphum sp.585* Koch, 1855 | 1 | 1 | - | - | - | 6,23% | - | - |
| *Dysaphis angelicae* (Koch, 1854) | 1 | 1 | - | - | - | 0,30% | - | - |
| *Dysaphis apiifolia* (Theobald, 1923) | 6 | 4 | 0,00% | 0,19% | 0,30% | 0,00% | P | 99 |
| *Dysaphis aucupariae* (Buckton, 1879) | 2 | 1 | 0,00% | 0,00% | 0,00% | 4,26% | M | 100 |
| *Dysaphis crataegi* (Kaltenbach, 1843) | 8 | 5 | 0,00% | 0,25% | 0,61% | 0,00% | P | 9 |
| *Dysaphis crithmi* (Buckton, 1886) | 3 | 2 | 0,00% | 0,10% | 0,15% | 0,00% | P | 14 |
| *Dysaphis foeniculus* (Theobald, 1923) | 1 | 1 | - | - | - | 2,33% | - | - |
| *Dysaphis lappae* (Koch, 1854) | 1 | 1 | - | - | - | 2,65% | - | - |
| *Dysaphis lauberti* (Börner, 1940) | 2 | 2 | 0,15% | 0,15% | 0,15% | 0,30% | M | 87 |
| *Dysaphis plantaginea* (Passerini, 1860) | 5 | 2 | 0,00% | 0,06% | 0,15% | 3,60% | M | 100 |
| *Dysaphis radicola* (Mordvilko, 1897) | 4 | 2 | 0,00% | 0,08% | 0,15% | 3,13% | M | 100 |
| *Dysaphis reaumuri* (Mordvilko, 1928) | 1 | 1 | - | - | - | 3,60% | - | - |
| *Dysaphis tulipae* (Boyer de Fonscolombe, 1841) | 3 | 1 | 0,00% | 0,00% | 0,00% | 0,15% | M | 63 |
| *Elatobium abietinum* (Walker, 1849) | 1 | 1 | - | - | - | 7,51% | - | - |
| *Ephedraphis ephedrae* (Nevsky, 1929) | 1 | 1 | - | - | - | 3,59% | - | - |
| *Eriosoma lanigerum* (Hausmann, 1802) | 1 | 1 | - | - | - | 9,04% | - | - |
| *Eriosoma lanuginosum* (Hartig, 1839) | 2 | 1 | 0,00% | 0,00% | 0,00% | 9,21% | M | 100 |
| *Eucallipterus tiliae* (Linnaeus, 1758) | 4 | 2 | 0,00% | 0,15% | 0,30% | 9,39% | M | 100 |
| *Eucarazzia elegans* (Ferrari, 1872) | 1 | 1 | - | - | - | 5,03% | - | - |
| *Euceraphis betulae* (Koch, 1855) | 5 | 4 | 0,00% | 0,46% | 0,61% | 3,76% | M | 100 |
| *Euceraphis punctipennis* (Zetterstedt, 1828) | 2 | 1 | 0,00% | 0,00% | 0,00% | 3,76% | M | 100 |
| *Forda marginata* Koch, 1857 | 1 | 1 | - | - | - | 10,09% | - | - |
| *Greenidea ficicola* Takahashi, 1921 | 1 | 1 | - | - | - | 8,54% | - | - |
| *Hayhurstia atriplicis* (Linnaeus, 1761) | 4 | 2 | 0,00% | 0,23% | 0,46% | 3,59% | M | 100 |
| *Hoplocallis picta* (Ferrari, 1872) | 1 | 1 | - | - | - | 8,87% | - | - |
| *Hyadaphis foeniculi* (Passerini, 1860) | 3 | 1 | 0,00% | 0,00% | 0,00% | 3,75% | M | 100 |
| *Hyadaphis passerini* (del Guercio, 1911) | 3 | 1 | 0,00% | 0,00% | 0,00% | 3,75% | M | 100 |
| *Hyalopterus amygdali* (Blanchard E., 1840) | 2 | 1 | 0,00% | 0,00% | 0,00% | 3,76% | M | 100 |
| *Hyalopterus persikonus* Miller, Lozier & Footit, 2008 | 1 | 1 | - | - | - | 5,71% | - | - |
| *Hyalopterus pruni* (Geoffroy, 1762) | 11 | 3 | 0,00% | 0,18% | 0,46% | 3,76% | M | 100 |
| *Hydaphias molluginis* Börner, 1939 | 1 | 1 | - | - | - | 3,19% | - | - |
| *Hydaphias mosana* Hille Ris Lambers, 1956 | 1 | 1 | - | - | - | 3,19% | - | - |
| *Hyperomyzus lactucae* (Linnaeus, 1758) | 16 | 1 | 0,00% | 0,00% | 0,00% | 3,59% | M | 100 |
| *Hyperomyzus picridis* (Börner & Blunck, 1916) | 4 | 1 | 0,00% | 0,00% | 0,00% | 3,92% | M | 100 |
| *Hyperomyzus rhinanthi* (Schouteden, 1903) | 1 | 1 | - | - | - | 4,72% | - | - |
| *Illinoia liriodendri* (Monell, 1879) | 1 | 1 | - | - | - | 4,58% | - | - |
| *Lachnus roboris* (Linnaeus, 1758) | 5 | 4 | 0,00% | 1,64% | 2,33% | 10,80% | M | 100 |
| *Lipaphis erysimi* (Kaltenbach, 1843) | 2 | 1 | 0,00% | 0,00% | 0,00% | 5,36% | M | 100 |
| *Lipaphis lepidii* (Nevsky, 1929) | 1 | 1 | - | - | - | 4,87% | - | - |
| *Macchiatiella rhamni* (Boyer de Fonscolombe, 1841) | 1 | 1 | - | - | - | 4,56% | - | - |
| *Macrosiphoniella abrotani* (Walker, 1852) | 1 | 1 | - | - | - | 2,64% | - | - |
| *Macrosiphoniella absinthii* (Linnaeus, 1758) | 1 | 1 | - | - | - | 0,61% | - | - |
| *Macrosiphoniella artemisiae* (Boyer de Fonscolombe, 1841) | 2 | 1 | 0,00% | 0,00% | 0,00% | 2,64% | M | 100 |
| *Macrosiphoniella helichrysi* Remaudière, 1952 | 1 | 1 | - | - | - | 0,61% | - | - |
| *Macrosiphoniella millefolii* (de Geer, 1773) | 3 | 3 | 0,15% | 0,20% | 0,30% | 4,07% | M | 100 |
| *Macrosiphoniella oblonga* (Mordvilko, 1901) | 1 | 1 | - | - | - | 5,37% | - | - |
| *Macrosiphoniella sanborni* (Gillette, 1908) | 1 | 1 | - | - | - | 4,55% | - | - |
| *Macrosiphoniella tanacetaria* (Kaltenbach, 1843) | 2 | 1 | 0,00% | 0,00% | 0,00% | 2,64% | M | 100 |
| *Macrosiphum albifrons* Essig, 1911 | 2 | 1 | 0,00% | 0,00% | 0,00% | 1,23% | M | 100 |
| *Macrosiphum cerinthiacum* Börner, 1950 | 1 | 1 | - | - | - | 0,00% | - | - |
| *Macrosiphum cholodkovskyi* (Mordvilko, 1909) | 1 | 1 | - | - | - | 0,46% | - | - |
| *Macrosiphum euphorbiae* (Thomas, 1878) | 18 | 11 | 0,00% | 0,57% | 1,23% | 0,46% | P | 71 |
| *Macrosiphum funestum* (Macchiati, 1885) | 2 | 2 | 0,30% | 0,30% | 0,30% | 0,76% | M | 77 |
| *Macrosiphum rosae* (Linnaeus, 1758) | 17 | 2 | 0,00% | 0,08% | 0,15% | 0,76% | M | 95 |
| *Macrosiphum stellariae* Theobald, 1913 | 5 | 1 | 0,00% | 0,00% | 0,00% | 0,00% | P | 100 |
| *Megoura viciae* Buckton, 1876 | 6 | 2 | 0,00% | 0,15% | 0,46% | 3,74% | M | 100 |
| *Megourella tribulis* (Walker, 1849) | 1 | 1 | - | - | - | 3,75% | - | - |
| *Melanaphis bambusae* (Fullaway, 1910) | 1 | 1 | - | - | - | 7,35% | - | - |
| *Melanaphis donacis* (Passerini, 1862) | 5 | 1 | 0,00% | 0,00% | 0,00% | 8,72% | M | 100 |
| *Melanaphis luzulella* (Hille Ris Lambers, 1947) | 1 | 1 | - | - | - | 7,71% | - | - |
| *Melanaphis pyraria* (Passerini, 1861) | 3 | 1 | 0,00% | 0,00% | 0,00% | 7,17% | M | 100 |
| *Metopeurum fuscoviride* Stroyan, 1950 | 2 | 1 | 0,00% | 0,00% | 0,00% | 2,64% | M | 100 |
| *Metopolophium dirhodum* (Walker, 1849) | 1 | 1 | - | - | - | 4,72% | - | - |
| *Microlophium carnosum* (Buckton, 1876) | 3 | 2 | 0,00% | 0,10% | 0,15% | 4,88% | M | 100 |
| *Myzocallis boerneri* Stroyan, 1957 | 1 | 1 | - | - | - | 7,73% | - | - |
| *Myzocallis carpini* (Koch, 1855) | 1 | 1 | - | - | - | 2,33% | - | - |
| *Myzocallis castanicola* Baker, 1917 | 3 | 2 | 0,00% | 0,31% | 0,46% | 9,22% | M | 100 |
| *Myzocallis coryli* (Goeze, 1778) | 5 | 4 | 0,00% | 1,27% | 3,12% | 2,33% | P | 100 |
| *Myzus cerasi* (Fabricius, 1775) | 9 | 6 | 0,00% | 0,51% | 1,38% | 5,52% | M | 100 |
| *Myzus lythri* (Schrank, 1801) | 7 | 1 | 0,00% | 0,00% | 0,00% | 5,22% | M | 100 |
| *Myzus persicae* (Sulzer, 1776) | 6 | 3 | 0,00% | 0,10% | 0,30% | 5,39% | M | 100 |
| *Myzus varians* Davidson, 1912 | 2 | 1 | 0,00% | 0,00% | 0,00% | 5,19% | M | 100 |
| *Nasonovia ribisnigri* (Mosley, 1841) | 1 | 1 | - | - | - | 4,55% | - | - |
| *Nearctaphis bakeri* (Cowen, 1895) | 5 | 1 | 0,00% | 0,00% | 0,00% | 4,87% | M | 100 |
| *Neotoxoptera formosana* (Takahashi, 1921) | 1 | 1 | - | - | - | 6,18% | - | - |
| *Ovatus crataegarius* (Walker, 1850) | 2 | 1 | 0,00% | 0,00% | 0,00% | 4,87% | M | 100 |
| *Panaphis juglandis* (Goeze, 1778) | 4 | 2 | 0,00% | 0,08% | 0,15% | 9,21% | M | 100 |
| *Patchiella reaumuri* (Kaltenbach, 1843) | 1 | 1 | - | - | - | 9,05% | - | - |
| *Pemphigus immunis* Buckton, 1896 | 1 | 1 | - | - | - | 8,74% | - | - |
| *Pemphigus vesicarius* Passerini, 1861 | 2 | 2 | 0,15% | 0,15% | 0,15% | 7,39% | M | 100 |
| *Periphyllus acericola* (Walker, 1848) | 1 | 1 | - | - | - | 7,92% | - | - |
| *Periphyllus bulgaricus* Tashev, 1964 | 1 | 1 | - | - | - | 8,08% | - | - |
| *Periphyllus sp.1479* van der Hoeven, 1863 | 1 | 1 | - | - | - | 7,92% | - | - |
| *Periphyllus testudinaceus* (Fernie, 1852) | 8 | 3 | 0,00% | 0,12% | 0,30% | 11,56% | M | 100 |
| *Phorodon humuli* (Schrank, 1801) | 4 | 2 | 0,00% | 0,08% | 0,15% | 5,04% | M | 100 |
| *Phyllaphis fagi* (Linnaeus, 1767) | 1 | 1 | - | - | - | 9,91% | - | - |
| *Pleotrichophorus glandulosus* (Kaltenbach, 1846) | 1 | 1 | - | - | - | 5,85% | - | - |
| *Prociphilus bumeliae* (Schrank, 1801) | 3 | 1 | 0,00% | 0,00% | 0,00% | 9,38% | M | 100 |
| *Protaphis terricola* (Rondani, 1848) | 4 | 2 | 0,00% | 0,15% | 0,30% | 5,70% | M | 100 |
| *Pterocallis alni* (De Geer, 1773) | 2 | 1 | 0,00% | 0,00% | 0,00% | 8,71% | M | 100 |
| *Pterocomma pilosum* Buckton, 1879 | 3 | 2 | 0,00% | 0,10% | 0,15% | 1,85% | M | 100 |
| *Pterocomma populeum* (Kaltenbach, 1843) | 5 | 3 | 0,00% | 0,24% | 0,46% | 1,85% | M | 100 |
| *Pterocomma rufipes* (Hartig, 1841) | 1 | 1 | - | - | - | 3,45% | - | - |
| *Rhopalosiphum insertum* (Walker, 1849) | 1 | 1 | - | - | - | 3,27% | - | - |
| *Rhopalosiphum maidis* (Fitch, 1856) | 1 | 1 | - | - | - | 6,69% | - | - |
| *Rhopalosiphum nymphaeae* (Linnaeus, 1761) | 1 | 1 | - | - | - | 6,35% | - | - |
| *Rhopalosiphum padi* (Linnaeus, 1758) | 2 | 2 | 1,23% | 1,23% | 1,23% | 3,27% | M | 100 |
| *Roepkea marchali* (Börner, 1931) | 1 | 1 | - | - | - | 6,18% | - | - |
| *Semiaphis dauci* (Fabricius, 1775) | 7 | 2 | 0,00% | 0,04% | 0,15% | 4,83% | M | 100 |
| *Sipha elegans* del Guercio, 1905 | 2 | 1 | 0,00% | 0,00% | 0,00% | 7,57% | M | 100 |
| *Sipha maydis* Passerini, 1860 | 5 | 4 | 0,00% | 2,00% | 3,92% | 7,57% | M | 100 |
| *Sitobion fragariae* (Walker, 1848) | 4 | 4 | 0,15% | 0,41% | 0,61% | 5,38% | M | 100 |
| *Staticobium sp.1519* Mordvilko, 1914 | 1 | 1 | - | - | - | 3,58% | - | - |
| *Stomaphis longirostris* (Fabricius, 1787) | 1 | 1 | - | - | - | 8,53% | - | - |
| *Takecallis arundicolens* (Clarke, 1903) | 1 | 1 | - | - | - | 8,45% | - | - |
| *Takecallis arundinariae* (Essig, 1917) | 1 | 1 | - | - | - | 8,45% | - | - |
| *Takecallis taiwana* (Takahashi, 1926) | 1 | 1 | - | - | - | 9,93% | - | - |
| *Thecabius affinis* (Kaltenbach, 1843) | 2 | 2 | 0,30% | 0,30% | 0,30% | 7,39% | M | 100 |
| *Thelaxes dryophila* (Schrank, 1801) | 6 | 1 | 0,00% | 0,00% | 0,00% | 5,91% | M | 100 |
| *Thelaxes suberi* (del Guercio, 1911) | 4 | 3 | 0,00% | 1,21% | 1,86% | 5,91% | M | 100 |
| *Therioaphis riehmi* (Börner, 1949) | 4 | 3 | 0,00% | 0,38% | 0,76% | 9,04% | M | 100 |
| *Tinocallis takachioensis* Higuchi, 1972 | 1 | 1 | - | - | - | 9,62% | - | - |
| *Toxopterina vandergooti* (Börner, 1939) | 2 | 2 | 0,30% | 0,30% | 0,30% | 5,40% | M | 100 |
| *Trama troglodytes* von Heyden, 1837 | 1 | 1 | - | - | - | 11,12% | - | - |
| *Tuberculatus annulatus* (Hartig, 1841) | 6 | 3 | 0,00% | 1,34% | 3,91% | 7,92% | M | 100 |
| *Tuberculatus eggleri* Börner, 1950 | 2 | 2 | 0,61% | 0,61% | 0,61% | 7,92% | M | 100 |
| *Uroleucon achilleae* (Koch, 1855) | 1 | 1 | - | - | - | 4,08% | - | - |
| *Uroleucon aeneum* (Hille Ris Lambers, 1939) | 4 | 1 | 0,00% | 0,00% | 0,00% | 2,65% | M | 100 |
| *Uroleucon hypochoeridis (Fabricius, 1779)* | 19 | 4 | 0,00% | 0,92% | 2,32% | 3,27% | M | 98 |
| *Uroleucon inulae* (Ferrari, 1872) | 3 | 3 | 0,15% | 0,20% | 0,30% | 3,27% | M | 100 |
| *Uroleucon jaceae* (Linnaeus, 1758) | 11 | 5 | 0,00% | 0,41% | 0,92% | 2,64% | M | 93 |
| *Uroleucon nigrocampanulae* (Theobald, 1928) | 1 | 1 | - | - | - | 0,15% | - | - |
| *Uroleucon picridis* (Fabricius, 1775) | 2 | 1 | 0,00% | 0,00% | 0,00% | 3,13% | M | 100 |
| *Uroleucon rapunculoidis* (Börner, 1939) | 1 | 1 | - | - | - | 0,15% | - | - |
| *Uroleucon solidaginis* (Fabricius, 1779) | 1 | 1 | - | - | - | 2,64% | - | - |
| *Uroleucon sonchi* (Linnaeus, 1767) | 15 | 4 | 0,00% | 0,10% | 0,46% | 2,96% | M | 100 |
| *Uroleucon sp.1586* Mordvilko, 1914 | 1 | 1 | - | - | - | 3,75% | - | - |
| *Uroleucon sp.614* Mordvilko, 1914 | 1 | 1 | - | - | - | 2,96% | - | - |
| *Uroleucon sp.832* Mordvilko, 1914 | 1 | 1 | - | - | - | 3,12% | - | - |
| *Wahlgreniella arbuti* (Davidson, 1910) | 1 | 1 | - | - | - | 4,07% | - | - |

Number of specimens sampled (NS.), number of haplotypes (NH.), minimum, mean and maximum within species divergence (WSD) and minimum between species divergence (BSD) calculated using a K2P model of evolution. Bootstrap values BP are reported for the smallest clades including all representatives of the species, which are either monophyletic (M) or polyphyletic (P) as retrieved in the tree from Figure S.4.
