# Supplementary material for: DNA Barcoding and the Associated PhylAphidB@se Website for the Identification of European Aphids (Insecta: Hemiptera: Aphididae)
Source: PLoS One. 2014 Jun 4;9(6):e97620. doi: 10.1371/journal.pone.0097620 (PMC4045754; doi:10.1371/journal.pone.0097620)
Supplement: Text S1 — Detailed discussion on aphid species groups. Detailed discussion and references for each aphid species groups encountered in the study and the match to our DNA barcoding data. (DOCX) [file pone.0097620.s005.docx]

**Detailed discussion and references for each aphid species group encountered in the study and their match to our DNA barcoding data**

***Aphis* species group**

Four morphological groups of *Aphis* have been recognized in recent studies in Europe [1,2]: frangulae-like, black-backed aphids (=*A. craccivora* group), black aphids (=*A. fabae* group) and *nasturtii*-like aphids.

In our study, species from the morphological *frangulae*-like species group were scattered between three polyphyletic clades (clades A, F and G). Consistent with previous studies [3,4], the two most frequent and most studied species (i.e. *A. frangulae* and *A. gossypii)* had distinct barcode sequences. Barcodes therefore appear to be more effective than morphology for the assignment of individuals to these two species. However, *A. frangulae* and *A. gossypii* also share haplotypes with other *Aphis* species that were not sampled in previous studies, potentially rendering species assignment by barcoding ambiguous. The undersampling of closely related species is a well known problem in barcoding studies and may lead to the underestimation of global error rates [5]. Clade F included specimens of *Aphis frangulae*, *Aphis frangulae-like* species feeding on Labiateae*, Aphis mamonthovae*, *Aphis parietariae* and *Aphis punicae*. All these species are so closely related to *A. frangulae* that no reliable identification can be made on the basis of morphological characters alone [2,6,7]. Consistent with the findings of Stroyan [2], the only specimen of *A. teucrii* we sequenced belonged to the *frangulae-*like clade. By contrast, *Aphis alienus,* which is closely related to and possibly even synonymous with *Aphis teucrii* [6], had a distinct barcode. *Aphis origani* and *A. serpilly* clustered in a polyphyletic clade (Clade G) and were clearly differentiated from other aphids of the *frangulae*-like species group to which they are thought to belong [2].

*Aphis gossypii* belongs to clade A, which also includes several species developing on Sedaceae (*A. sedi*), Asteraceae (*A.crepidis* *A. leontodontis*, *A. taraxacicola*), Plantaginaceae (*A. longirostris, A. plantaginis*) and Hypericaceae (*A. chloris*). With the exception of *A. sedi*, all these species live in ant shelters on root collars and etiolated leaf bases of their host plants. Heie [1] placed most of them in a distinct species group named the *plantaginis* group. However, Stroyan [2] suggested that they were quite similar to *Aphis frangulae* (including *A. gossypii* as a subspecies), although they could be distinguished by their shorter hairs and more numerous marginal tubercles. He also felt that these characters might be adaptive convergences not indicative of a common ancestry. On the basis of our genetic data, it is not possible to differentiate species of the *plantaginis* group from those of the *frangulae*-like species group. Consequently, the use of the *plantaginis* group does not appear to be justified, as already suggested by Stroyan.

In our sample, the intraspecific genetic divergence observed within *A. gossypii* was similar to that observed over a much larger, worldwide scale (0.61/0.62) [8]. This result goes against the view that the lack of broad geographic sampling for a given species is likely to result in an underestimation of within-species variability [5]. However, *Aphis gossypii* has recently expanded around the world through human activities, and this may account for our result.

Stroyan [2] placed 11 European species in the black-backed species group, which is mostly associated with Leguminosae. Seven of these species are present in our dataset. Two have distinct barcodes (*Aphis craccae* and *Aphis pseudocomosa*), the five remaining species being included in two polyphyletic clades (B and C). *Aphis cytisorum* and *Aphis ulicis* are grouped within clade C. These species develop on woody Leguminosae and differ morphologically from each other by barely discernible differences [2] in the ratio of length to basal width of the apical rostral segment [9]. *Aphis craccivora*, *Aphis coronillae* and *Aphis tirucallis* (= *Aphis euphorbiae spp tirucallis*) clustered within clade B. The first two of these species, which may infest the same host plants, could be separated morphologically by the regular development of marginal tubercles on tergites in *A. coronillae* [2], a character less frequent in *A. craccivora*. Finally, *Aphis tirucallis,* which lives on *Euphorbia spp.* (Euphorbiaceae), is considered to be an isolated species. *Aphis intybi*, which lives on *Cichorium intybus* (Asteraceae), is also included in this clade. The specimens of these species cluster in a monophyletic clade and they are included in clade B due to a specimen of *A. craccivora* being more closely related to them than to its conspecific specimens. *A. craccivora* has a relatively high level of intraspecific variability for an *Aphis* species, with its different haplotypes grouping into different clades. This suggests that *A. craccivora*, as currently understood, may actually be a complex of host races or biotypes, as already suggested by Stroyan [2] and by Wang *et al.* [4]. If this result is confirmed, then *Aphis intybi* may belong to this complex.

Black aphids, corresponding to the *Aphis fabae* species group, have been discussed in detail in previous publications [2,10,11,12,13,14,15]. Iglish [11,12] subdivided this species group into the black aphid species group *sensu stricto* and the black aphid species group *sensu lato*. For the first species group (*sensu stricto*), it is not possible to produce an identification key based on morphological and biological (host-plant) characters only [14], but species and subspecies from this species group have been differentiated on the basis of isoenzyme data [15]. In our study, all the specimens from the *sensu stricto* species group belonged to various subspecies of *Aphis fabae.* All are named simply “*Aphis fabae”*, because we did not consider the subspecies rank in this study. However, we detected no genetic structure within *Aphis fabae*, strongly suggesting that the subdivision of *A. fabae* into subspecies may not be valid.

The black aphid species group *sensu lato* includes 11 species [1,2,14]. Most of the species pairs in this species group display highly significant morphometric differences, which are used for identification. However, there is often an overlap in values between species, and it is not feasible to identify these species on the basis of morphological characters alone [14]. All aphid species from the *sensu lato* species group are easily separated by a combination of host-plant identification and morphological characters [14]. Our dataset includes seven species from this species group. Four of these species (*Aphis ilicis*, *A. rumicis*, *A. veratri* and *A. sambuci*) have distinct barcode sequences. By contrast to the findings of several taxonomic studies [1,2,12,15], our analysis clustered specimens from the other three species (*Aphis newtoni*, *A. hederae* and *A. viburni*) with the *Aphis fabae* specimens, to form clade D. The poor discriminating power of COI for these species has been reported before [16] and Coeur d’acier *et al.* (2007) obtained similar results with several mitochondrial genes. More surprisingly, *Aphis lambersi* was found to cluster within this clade. This species lives on root collars and in basal leaf sheaths of *Daucus carota* and *Foeniculus sp.* [6]. *A. lambersi* is morphologically similar to *A. longirostrata*, *A. plantaginis* and *A. taraxacicola* [1,2], which belong to the *frangulae*-like species group (see discussion above), and all these species have short hairs and numerous marginal tubercles. However, *A. lambersi* differs from the other species in having a heavier sclerotic pattern, a relatively short apical segment and an association with Apiaceae [2]. These traits are consistent with the assignment of *A. lambersi* to the black aphid group. Apiaceae also host *Aphis fabae* and the presence of dorsal sclerites on the abdominal segment of the aphid and a bluish green or black color [1] are morphological traits common to the members of the black aphid species group. A re-evaluation of the taxonomic position of all these species might improve the definition of the morphological characteristics of these species groups.

Clade H contains specimens of the polyphagous *Aphis nasturtii* and the Malvaceae-feeding species *Aphis althaeae* (= *A. davletshinae*), *A. umbrella* and *A. rostellum*-like species. These species do not constitute a formally recognized group, but several authors have already noted their resemblance and suggested that they might be related [6]. *Aphis althaeae* develops on *Alcea* spp. and Althaea spp., whereas the other two species live on *Malva* spp. *Aphis rostellum* has been found on *Malva chinensis* in China and is sometimes considered to be a geographic variant of *A. umbrella* [6]. However, these species differ in the length of the apical rostral segment, a character that has often been used to separate *Aphis* species. The specimens we included in our study were sampled on *Malva* in Europe and had an apical rostral segment of similar length to that of *Aphis rostellum*. We currently refer to them as “*A. rostellum*-like”, whilst awaiting the examination of Chinese specimens.

With the exception of *Aphis nasturtii*, these species differ by only a few, nucleotide differences, but these differences are sufficiently consistent for the correct assignment of species to the appropriate cluster on the NJ tree. *Aphis nasturtii* was found to be paraphylic, but this polyphagous species has been little studied [2]. Future studies may confirm the existence of several entities (subspecies or biotypes). *Aphis spiraephaga* and *Aphis galiiscabri* cluster together in clade E and both have the small barcode sequence. This result is surprising, because these species have never been considered to be closely related. Both are relatively uncommon, oligophagous and associated with different host plants. *Aphis spiraephaga* develops on *Spiraeae* spp*.,* whereas *A. galiiscabri* is associated with *Galium* spp*.* However, the two species have been described similarly, as “dark greenish brown and heavily wax-powdered” aphids. Furthermore, *Aphis spiraephaga* appears to be more polyphagous than previously thought [6], being found on host plants from different plant families: *Epilobium* spp., *Arabis*, *Carum*, *Erica*, *Filipendula*, *Helipterum*, *Symphoricarpus*, *Trinia, Valeriana* and *Centranthus ruber* [17,18,19]. Further investigations are required to determine whether *Aphis galiiscabri* is a distinct species or simply *A. spiraephaga* developing on *Galium* sp*.*

**The *Brachycaudus* species group**

Clade I groups together specimens of *Brachycaudus cardui* and *B. lateralis*. The status of these species is debatable. Börner [20] , Remaudière & Remaudière [21], Heie [22] Burger [23] and others considered these two taxa to correspond to different species, whereas, Müller & Horatschek [24] and Andreev [25] downgraded *B. lateralis* to a subspecies of *B. cardui*, and Shaposhnikov [26], Burger [23] and Eastop and Hille Ris Lambers [27] treated *B. lateralis* as a synonym of *B. cardui*. Our data cannot resolve this taxonomic ambiguity and we were unable to distinguish between these two taxa.

Clade K includes specimens of *B. prunicola*, *B. tragopogonis* and *B. schwartzi* and is traditionally recognized as the *B. prunicola* group [28]. The morphological similarities between these taxa have given rise to controversy concerning their taxonomic status. These species were defined principally on the basis of their host plants [20,29,30,31], with each species living year-round on its respective host: *Prunus spinosa* (*B. prunicola*), *Prunus persica* (*B. schwartzi*) and *Tragopogon* *spp*. (*B. tragopogonis*). However, Hille Ris Lambers [32] pointed out that the spring migrants of *B. schwartzi* and *B. prunicola* were successfully transferred to *Tragopogon pratensis* and he therefore suggested that these three species should be considered to be synonymous. A detailed investigation of the species of the *prunicola* group on the basis of comparative morphology, biology and cross-breeding trials was carried out by Thomas [33]. He concluded that the three taxa should be treated as subspecies and suggested the name *B.* *prunicola* ssp. *prunifex* for the population that alternates between *Prunus* and *Tragopogon*. This proposal is partly consistent with that of Shaposhnikov [26], who considered *B. tragopogonis* to be a subspecies of *B. prunicola,* which he considered to be synonymous with *B. schwartzi,* and with that of Cocuzza *et al.* [28], who treated *B. tragopogonis* as a valid species and *B. schwartzi* as a subspecies of *B. prunicola.* In these two studies, *B. prunicola* s.str. was considered to be the taxon alternating between *Prunus* and *Tragopogon,* but with some populations living on *Prunus spinosa* throughout the year. Some of these populations from Britain and northern France have recently been distinguished as a different species, *B. prunifex,* on the basis of a small number of morphometric differences [34]. Recent studies have recognized four specific taxa: the three species defined by Börner plus *B. prunifex*.

This short historical review highlights the difficulties encountered when trying to define the taxa within these species groups, to specify their taxonomic rank and even to characterize their respective biological features. Specimens identified as *B. schwartzi* and *B. prunicola* in our study could not be distinguished on the basis of their barcode sequences. Our results were particularly ambiguous for *B. tragopogonis*. Indeed, five specimens shared a haplotype with other species of the group, but four specimens formed a distinct clade. It is possible that we confused summer populations of *B. prunicola* on *Tragopogon* with *B. tragopogonis.* This might account for the five unidentified specimens, the other four corresponding to the distinct barcode of true *B. tragopogonis*. Alternatively, this last species may simply be more genetically variable than the other species of this group. *Brachycaudus lychnidis* and *B. populi* could not be distinguished on the basis of their barcode sequences. They are now recognized as two closely related species, but they were confused before the study by Burger [23]. The morphological characters that Burger reported to be important for identification discriminate perfectly between these two species, ruling out the misidentification of our specimens. This is perhaps one of the rare cases in which barcoding was clearly less successful than the use of morphological data.

**The *Dysaphis* species group**

Clade L includes several species of the genus *Dysaphis* subgenus *Dysaphis*. This large subgenus is traditionally divided in eight well defined species groups and a number of isolated species of uncertain taxonomic position [35]. Six of these species groups (the *D. brancoi*, *D. crataegi*, *D. devecta*, *D. emicis*, *D. foeniculus* and *D. lappae* species groups) contain species from the European fauna, and we sampled four of them. Specimens of *Dysaphis* *lappae* (the only European species representative of the *D. lappae* group) have a distinct barcode sequence. This is also the case for *Dysaphis radicicola,* a member of the *D. devecta* species group. The *D. devecta* species group includes three other European species that were, unfortunately, not represented in our dataset. All European species belonging to the *D. foeniculus* species group are represented in our dataset. One of these species, *Dysaphis foeniculus,* has a distinct barcode sequence, but the other two, *Dysaphis crithmi* and *D. tulipae,* are nested within clade L in the *D. crataegi* species group. Stroyan [35] discussed the positioning of these two species and our results seem to confirm his conclusion. Specimens of the four species from the *D. crataegi* group (which includes six European species), *Dysaphis crataegi*, *D. angelicae*, *D. lauberti* and *D. apiifolia* grouped together in clade L, which contains species displaying little or no divergence. This group has been fully documented in Western Europe by Börner [36] and Stroyan [35,37,38]. These morphologically similar species all feed on *Crataegus* as a primary host and have Umbelliferae as secondary hosts. It remains unclear whether these taxa should be treated as separate species or as subspecies of *D. crataegi,* because the current classification is arbitrary and not based on valid biological criteria [39]. Further sampling is required for the *Dysaphis* species not included in this study. This should make it possible to investigate (i) the concordance between traditionally recognized species groups and barcoding data ( ii) the lack of resolution of barcodes within species groups.

**The *Macrosiphum* species group**

The last genus containing poorly discriminated species was *Macrosiphum*. Four of the seven species present in our sampling formed two pairs of species: *M. cerinthiacum / N. stellariae* (Clade M) and *M. cholodkovskyi / M. euphorbiae* (Clade N). With the exception of the understudied *M. cerinthiacum*, these species belong to the *M. euphorbiae* species group [40,41]. The *M. euphorbiae* species group has been investigated in morphology studies [42,43], host plant transfer trials [41,43,44] and hybridization experiments [41,44,45]. These studies concluded that *M. cholodkovskyi* was an isolated species displaying discrete morphological differences, almost complete reproductive isolation and strict monophagy on *Filipendula ulmariae,* a host plant not used by the other species of the group. Conversely, *M. euphorbiae* and *M. stellariae* may colonize the same host plants and hybridize. These conclusions are not consistent with our results, which differentiated *M. stellariae* but clustered specimens of *M. euphorbiae* and *M. cholodkovsyi* together. As reported by Foottit [8], who sampled Nearctic aphids, specimens of *M. eurphorbiae* displayed a higher within-species diversity than most aphid species. Some haplotypes are grouped into well sustained clades. This may reflect the presence of different biotypes, subspecies or undescribed species in this polyphagous species. The specimen of *M. cholodkovskyi* was nested within the *M. euphorbiae* clade, but it had a distinctive, different haplotype. Its assignment to the polyphyletic species *M. euphorbiae* might be artifactual, due to unresolved taxonomic issues.

*Macrosiphum stellariae* was long considered to be a monophagous species associated with *Stellaria holostea*, before host plant transfers demonstrated its polyphagy [41,43]. *M. cerinthiacum* lives under the leaves of *Cerinthe minor* (Boraginaceae)*.* Its life cycle is unknown. Further investigations are required to determine whether *M. cerinthiacum* actually corresponds to specimens of *M. stellariae* living on *Cerinthe* or whether these two species are valid separate taxa.

**References**

1. Heie OE (1986) The Aphidoidea (Hemiptera) of Fennoscandia and Denmark. III. Family Aphididae: subfamily Pterocommatinae & tribe Aphidini of subfamily Aphidinae. Fauna Entomologica Scandinavica 17: 1-314.

2. Stroyan HLG (1984) Aphids- Pterocommatinae and Aphidinae (Aphidini) Homoptera, Aphididae. London: The Royal Entomological Society of London. 232 p.

3. Carletto J, Blin A, Vanlerberghe-Masutti F (2009) DNA-based discrimination between the sibling species *Aphis gossypii* Glover and *Aphis frangulae* Kaltenbach. Systematic Entomology 34: 307-314.

4. Wang JF, Jiang LY, Qia GX (2011) Use of mitochondrial COI sequence to identify species of the subtribe Aphidina. ZooKeys 122: 1-17.

5. Meyer CP, Paulay G (2005) DNA Barcoding: error rates based on comprehensive sampling. PLoS Biology 3: 2229-2238.

6. Blackman RL, Eastop VF (2006) Aphids on the world's herbaceous plants and schrubs. Chichester: John Wiley & Sons Ltd. 1439 p.

7. Nieto Nafria JM, Mier Durante MP, Garcia Prieto F, Perez Hidalgo N (2005) Hemiptera Aphididae III. Madrid: Museo Nacional de Ciencias Naturales, CSIC. 362 p.

8. Foottit RG, Maw HEL, von Dohlen CD, Hebert PDN (2008) Species identification of aphids (Insecta: Hemiptera: Aphididae) through DNA barcodes. Molecular Ecology Resources 8: 1189-1201.

9. Jacob FH (1948) An account of a "Black aphid" *Aphis ulicis* Walker, 1870 (Hemiptera, Aphididae). Proceedings of the Royal Entomological Society of London (B) 17: 57-62.

10. Coeur d'acier A, Jousselin E, Martin J-F, Raplus J-Y (2007) Phylogeny of the genus *Aphis* Linnaeus, 1758 (Homoptera: Aphididae) inferred from mitochondrial DNA sequences. Molecular Phylogenetics and Evolution 42: 598-611.

11. Iglisch I (1968) Über die Entstehung der Rassen der "Schwarzen Blattlaüse (*Aphis fabae* Scop. und verwandte Arten), über ihre phytopathologische Bedeutung und über die Aussichten für erfolgversprechende Bekämpfungsmassnahmen (Homoptera: Aphididae). Mitteilungen aus der Biologischen Bundesanstalt für Land-und Forstwirtschaft 131: 5-34.

12. Iglisch I (1970) Zur Aufstellung eines Verwandtschaftsbildes der "Schwarzen Blattlaüse " , *Aphis fabae* Scop. und verwandte Arten, nach Biologischen Merkmalen (Homoptera: Aphididae). Zeitschrift für angewandte Zoologie 65: 304-308.

13. Janisch R (1926) Lebensweise und Systematik der "Scwarzen Blattläuse". Arbeiten aus der Biologischen Reichsanstalt für Land-und Forstwirtschaft 14: 291-366.

14. Jorg E, Lampel G (1995) Morphological studies on the *Aphis fabae* group (Homoptera, Aphididae). Mitteilungen der Schweizerischen Entomologischen Gesellschaft 68: 387-412.

15. Jorg E, Lampel G (1996) Enzyme electrophoretic studies on the *Aphis fabae* group (Hom., Aphididae). Journal of Applied Entomology 120: 7-18.

16. Lee W, Kim H, Lim J, Choi HR, Kim Y, *et al.* (2011) Barcoding aphids (Hemiptera: Aphididae) of the Korean Peninsula: updating the global data set. Molecular Ecology Resources 11: 32-37.

17. Müller FP (1987) Faunistisch-ökologische Untersuchungen über Aphiden im westlichen Erzgebirge und Vogtland (Insecta, Homoptera, Aphidina). Faunistische Abhandlungen 14: 105-129.

18. Barbagallo S, Stroyan HLG (1982) Osservazioni biologiche, ecologiche e tassinomiche sull'afidofauna delle Sicilia. Frustula Entomologica 3: 1-182.

19. Holman J (1990) One new and one little known European *Aphis* species (Homoptera, Aphididae). Acta Entomologica Bohemoslovaca 87: 122-127.

20. Börner C (1952) Europae centralis Aphides. Die Blattläusse Mitteleuropas. Namen, Synonyme, Wirtspflanzen, Generationszyklen. Mitteilungen der Thüringischen Botanischen Gesellschaft 3: 1-488.

21. Remaudière G, Remaudière M (1997) Catalogue des Aphididae du monde - Catalogue of the world's Aphididae [Homoptera, Aphidoidea]. Paris: INRA 376 p.

22. Heie OE (1992) The Aphidoidea (Hemiptera) of Fennoscandia and Denmark. IV. Family Aphididae: Part 1 of tribe Macrosiphini of subfamily Aphidinae. Fauna Entomologica Scandinavica 25 (31): 1-188.

23. Burger HC (1975) Key to the European species of *Brachycaudus*, subgenus *Acaudus* (Homoptera: Aphidoidea) with redescriptions and note on *B. persicae*. Tijdschrift voor Entomologie 118: 99-116.

24. Müller FP, Horatschek A (1979) *Brachycaudus cardui lateralis* (Walker, 1848), Erstfund in Osterreich und Generationenfolge (Homoptera, Aphididae). Mitteilungen des Naturwissenschaftlicher Vereines für Steiermark 109: 309-316.

25. Andreev AV (2004) The subgeneric classification of *Brachycaudus* van der Goot. In: Simon JC, Dedryver CA, Rispe C, Hulle M, editors. Aphids in a new millennium: Proceedings of the Sixth International Symposium on Aphids, September, 2001. Rennes (France): INRA editions. pp. 111-117.

26. Shaposhnikov GK (1964) The aphid pests of the cherry plum and cherries. Entomological Review 43: 73-76.

27. Eastop VF, Hille Ris Lambers D (1976) Survey of the world's aphids. The Hague: Junk, W. 573 p.

28. Cocuzza G, Cavalieri V, Jousselin E, Coeur d'acier A, Barbagallo S (2007) Morphological and molecular analysis of *Brachycaudus*, subgenus *Appelia* complex (Rhynchota, Aphididae). Redia 90: 33-49.

29. Börner C (1930) Beiträge zu einem neuen System der Blattläuse. Archiv für Klassifikatorische und Phylogenetische Entomologie 1: 115-194.

30. Börner C (1931) Mitteilungen über Blattläuse. Anzeiger für Schädlingskund 7: 8-11.

31. Börner C (1951) Welche Pflanzen besiedelt die Schwarzgefleckte Pfirsichlaus *Appelia schwartzi* Börner ? Nachrichtenblatt für den Deutschen Pflanzenschutzdienst 31: 148-151.

32. Hille Ris Lambers D (1947) On some mainly western European aphids. Zoologische Mededeelingen 28: 291-333.

33. Thomas KH (1962) Die Blattläuse des Formenkreises *Brachycaudus prunicola* (Kalt.). Wissenschaftliche Zeitschrift der Universität Rostock 11: 325-342.

34. Blackman RL (2010) Aphids-Aphidinae (Macrosiphini). Handbooks for the Identification of British Insects 2 (7): 1-419.

35. Stroyan HLG (1985) Recent developments in the taxonomic study of the genus *Dysaphis* Börner. In: Szelegiewicz H, editor. Evolution and biosystematics of aphids: Proceedings of the International Aphidological Symposium at Jablona, 5-11 April, 1981 Jablona: Polska Akademia Nauk, Instytut Zoologii. pp. 347-391.

36. Börner C (1950) Neue europäische Blattlausarten. Naumburg: Privately published 19 p.

37. Stroyan HLG (1958) A contribution to the taxonomy of some British species of *Sappaphis* Matsumura, 1918 (Homoptera, Aphidoidea). The Journal of Linnean Society of London 43: 643-713.

38. Stroyan HLG (1963) A revision of the Bristish species of *Dysaphis* Börner : Part.II - The Subgenus *Dysaphis sensu stricto*. London: Her Majesty's Stationery Office. 119 p.

39. Blackman RL, Eastop VF (1994) Aphids on the world's trees: an identification and information guide. Wallingford,Oxon: C.A.B. International. 987 p.

40. Müller FP (1985) Biotype formation and sympatric speciation in aphids (Homoptera: Aphidinea). Entomologia Generalis 10: 161-181.

41. Möller FW (1972) Überwinterung des Artenkomplexes der Grünstreifigen Kartoffelblattlaus *Macrosiphum euphorbiae* (Thomas) und Polyözie der Fundatrizen. Archiv Pflanzenschutz 8: 305-312.

42. Hille Ris Lambers D (1939) Contributions to a monograph of the Aphididae of Europe. II. Genera *Dactynotus*, *Staticobium*, *Macrosiphum*, *Nasonaphis*, *Pharalis*. Temminckia 4: 1-134.

43. Möller FW (1971) *Macrosiphum stellariae* (Theobald) - eine bisher nicht von der Grünstreifigen Kartoffelblattlaus *Macrosiphum euphorbiae* (Thomas) abgegrenzte Art (Homoptera : Aphididae). Deutsche Entomologische Zeitschrift 18: 207-215.

44. Möller FW (1971) Bastardierungen innerhalb des Artenkomplexes um die Grünstreifige Kartoffelblattlaus *Macrosiphum euphorbiae* (Thomas). Beiträge zur Entomologie, Berlin 21: 531-537.

45. Möller FW (1970) Die erste gelungene bisexuelle fortpflanzung mit europäischen herkünften von *Macrosiphum euphorbiae* (Thomas) (Homptera: Aphididae). Zoologischer Anzeiger 184: 107-119.
