# Supplementary material for: DNA Barcoding and the Associated PhylAphidB@se Website for the Identification of European Aphids (Insecta: Hemiptera: Aphididae)
Source: PLoS One. 2014 Jun 4;9(6):e97620. doi: 10.1371/journal.pone.0097620 (PMC4045754; doi:10.1371/journal.pone.0097620)
Supplement: Figure S1 — Neighbor-joining tree. Neighbor-joining tree (K2P model) obtained from the analysis of the 1020 COI sequences from the 274 species of European aphid included in the study. Bootstrap values >50 are indicated at nodes. (PDF) [file pone.0097620.s001.pdf]

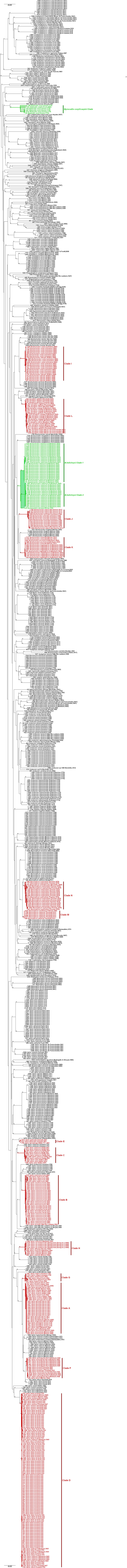
0.03
Myzocallis coryli/carpini Clade
Clade I
Clade L
B.helichrysi Clade 1
B.helichrysi Clade 2
Clade J
Clade K
Clade N
Clade M
Clade E
Clade C
Clade B
Clade H
Clade G
Clade A
Clade F
Clade D
0.03
